# Perfluorooctanesulfonate detection and removal with simple imidazolium macrocycles

**DOI:** 10.1039/d6sc05991k

**Published:** 2026-10-27

**Authors:** Arman C. Garcia, Ria Lian, Suranjan K. Paul, Xingchen Jin, Anika Seth, Sruthi Venkatesan, Hanfang Zhang, Yu-Dong Yang, Momena Awan, Paula Sharon Antony Benedict, Varshitha Rajasekaran, Veronica Carta, Jonathan L. Sessler, J. Ilja Siepmann, Richard J. Hooley

**Affiliations:** a Department of Chemistry, University of California–Riverside Riverside CA 92521 USA richard.hooley@ucr.edu; b Department of Chemistry and Chemical Theory Center, University of Minnesota–Twin Cities Minneapolis Minnesota 55455 USA siepmann@umn.edu; c Department of Chemistry, The University of Texas at Austin Texas 78712 USA sessler@cm.utexas.edu

## Abstract

Simple polycationic macrocycles act as effective receptors for the binding and precipitation-based removal of PFAS “forever chemicals” from aqueous solution. Recognition of perfluoroalkylsulfonates, such as perfluorooctanosulfonate (PFOS), is seen. This occurs even in the presence of inorganic salts and in biofluids, with the binding event “masking” the sulfonate headgroup in PFOS. This triggers self-aggregation of the macrocycle : PFOS complex and formation of disordered micron-scale aggregates that can be removed from solution through a single filtration step using sub-stoichiometric amounts of receptor. The process is highly efficient, with 99.8% PFOS removal being seen upon a single treatment with the cationic receptor. By pairing the recognition with an indicator dye, selective and sensitive PFOS detection can be achieved (LOD ≤48 nM), exploiting hydrophobic association of the dye with the receptor : PFOS aggregates. Molecular dynamics simulations show a three-step aggregation mechanism, with an initial single receptor complex forming, which triggers the formation of micelle-like species that incorporate supra-stoichiometric levels of PFOS. The detection sensitivity and selectivity can be enhanced by pairing the receptors with lipid vesicles, which associate with the receptor : PFOS : dye aggregates, but not the individual components. The net result is a highly sensitive dual-mode detection and removal strategy for a class of environmentally important targets.

## Abstract 2

Simple tetra-imidazolium macrocycles can efficiently and selectively recognize PFOS at the charged headgroup in both water and biofluids. This triggers an aggregation process, which enables extraction and detection when paired with an indicator dye.

## Graphical abstract

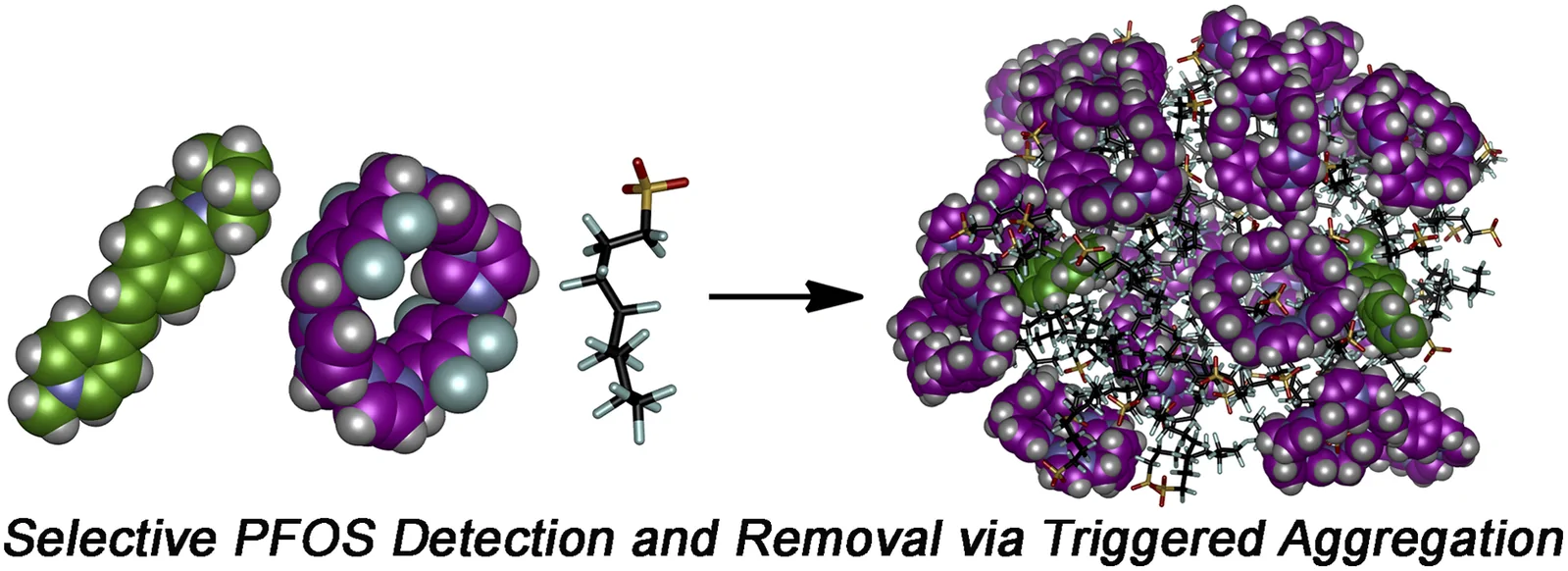

## Introduction

Per- and poly-fluoroalkyl substances (PFAS),^1–3^ often referred to as “forever chemicals”, have been widely used in consumer goods such as plastics, as well as flame retardant and non-stick coatings due to their high chemical stability and solvatophobicity, with both water and oil.^4^ Despite recent government actions to reduce and eliminate the use of PFAS, they are persistent in the environment and can be found in samples of drinking water, crops and beverages worldwide.^5–9^ Even small amounts of PFAS are deleterious to human health, with exposure linked to liver damage, cancers and a variety of fetal developmental issues.^10–13^ As such, there is a recognized need for simple PFAS detection at low concentrations in both water and biofluids. Also needed are simple-to-implement methods that permit efficient removal of PFAS from complex aqueous media.

Detection of PFAS can be achieved by engineering solutions, such as LC-MS or SERS,^14–17^ or through the use of solid-state sensors, such as MOFs,^18–20^ ZIFs,^21^ polymers^22,23^ and nanoparticles.^24^ PFAS removal can be achieved by weak-anion exchange sorbents^25–27^ or solid-phase extraction,^28,29^ as confirmed by LC-MS/MS analysis.^30^ While each of these techniques has merits, they are also subject to limitations, including technical complexity, cost, selectivity, and sensitivity. One notable challenge is the selective detection of perfluorinated alkyl sulfonates (*e.g.*, perfluorooctanesulfonate, PFOS), as opposed to the more common carboxylate derivatives (*e.g.* perfluorooctanoate, PFOA).^3^ This challenge is only exacerbated when attempting recognition and extraction from biofluids, as opposed to simple aqueous solutions.

Supramolecular recognition of PFAS has recently emerged as an effective method for the detection and capture of PFAS.^31^ Certain water-soluble macrocycles, such as pillararenes,^32^ cucurbiturils,^33^ reversibly formed disulfide^34,35^ or imine-based cyclophanes,^36,37^ and calixarenes,^38,39^ display strong affinities for various PFAS. In favorable cases, this permits extraction of the host : guest complexes from a source aqueous phase. Many examples of macrocyclic hosts reported for PFAS recognition and extraction do not use the free macrocycles, but incorporate them in polymeric networks, beads or hydrogels for solid-state extraction of PFAS.^31,40–42^ Self-assembled metal–ligand cage complexes can also bind PFAS in the internal cavity, enabling the selective recognition of certain PFAS types.^43–47^ This recognition can be coupled with an optical readout, allowing highly selective and sensitive detection of PFAS from water *via* extraction^48^ and transport^49^ by organic-soluble receptors. This progress notwithstanding, there remains a need for systems that address largely unsolved issues, such as cost-effectiveness, synthetic accessibility, scalability, selectivity, and sensitivity. In addition, recognition and detection of PFAS in impure aqueous matrices is non-trivial – environmental water samples, including drinking water and commercial beverages, as well as biological matrices, such as saliva or urine, contain a multitude of potentially competing ions and additives, often in relatively high concentrations.

An attractive strategy for recognizing PFAS in aqueous media is to take advantage of both facets of the PFAS molecule, *i.e.*, the charged headgroup and the fluorous tail. PFAS are prone to aggregation in water, driven by solvatophobic effects, far more so than the equivalent hydrocarbon equivalents. This self-aggregation can be enhanced by coordinating the charged headgroup of anionic PFAS (*e.g.*, PFOS and PFOA) and “masking” the charge, rendering these PFAS molecules more hydrophobic. This strategy has been used with cation-appended macrocycles such as pillararenes^32^ and deep cavitands^50^ to form large insoluble aggregates that can be easily extracted from water. However, these receptor systems require lengthy syntheses and are not amenable to large-scale use. Here we show that simple tetra-imidazolium macrocycles, synthesized in one step from commercially available starting materials, can recognize PFOS efficiently *via* interactions with the charged headgroup in both water and biofluids. This recognition event, in turn, triggers PFOS self-aggregation due to the self-complementary solvatophobic nature of the fluorous tails. This allows the highly efficient aggregation and filtration-based removal of PFOS from aqueous media. Moreover, the recognition can be paired with an indicator dye for an optical readout of the interaction, allowing detection of PFOS at a limit of detection (LOD) of 48 nM, 2.4 × 10^4^ ng L^−1^. The emission response can be further enhanced in the presence of lipid vesicles.

## Results and discussion

To address the dual challenge of PFAS detection and removal, we studied the suite of receptors (collectively described as XTI) shown in Fig. 1. Each receptor is readily prepared *via* simple cyclization of methyl-diimidazole and an appropriately chosen alkyl halide. Previously we have shown that PTI and CTI can recognize modified peptides in aqueous solution.^51^ As other cationic imidazolium-based macrocycles have been shown to recognize phosphate anions, such as RNA, GTP, and H_2_PO_4_^−^,^52–54^ we considered it likely that this receptor class would permit “dual-mode” recognition and removal of PFOS. To complement studies with PTI and CTI, we also synthesized the fluorinated variant FPTI, ostensibly to take advantage of fluorous-based solvatophobic interactions with the PFAS target. The bromide salts of PTI and CTI were synthesized *via* published methods,^51^ and FPTI·Br4 was obtained in 82% yield by heating methylene-diimidazole and bis (bromomethyl)-tetrafluorobenzene in CH_3_CN for 2 days, followed by rinsing the resulting white solid precipitate (Fig. S1–S8). Crystals suitable for single crystal X-ray diffraction (scXRD) analysis were grown *via* the vapor diffusion of ether into a saturated solution of FPTI·Br_4_ in CH_3_CN/H_2_O (Fig. 1e and S9). Whereas a prior scXRD analysis of PTI·Cl revealed that the host binds a single chloride anion in the cavity in the solid state (Fig. 1d),^51^ no evidence of anion binding was seen in the case of FPTI·Br_4_; rather, two molecules of CH_3_CN were found to occupy the FPTI cavity with the bromide counterions residing outside the macrocycle (Fig. 1e). Solution phase studies in water have shown that PTI is prone to anion-dependent reversible oligomerization;^51^ however, this is far less prevalent with CTI and FPTI. Quantum mechanical computations to find the minimum-energy geometry for monomeric isolated XTI yield a “book-like” arrangement for PTI and FPTI in accord with the scXRD structures. In contrast, CTI adopts a twisted geometry with two carboxylate groups pointing inward to the central macrocyclic core (Fig. S57).

**Fig. 1:**
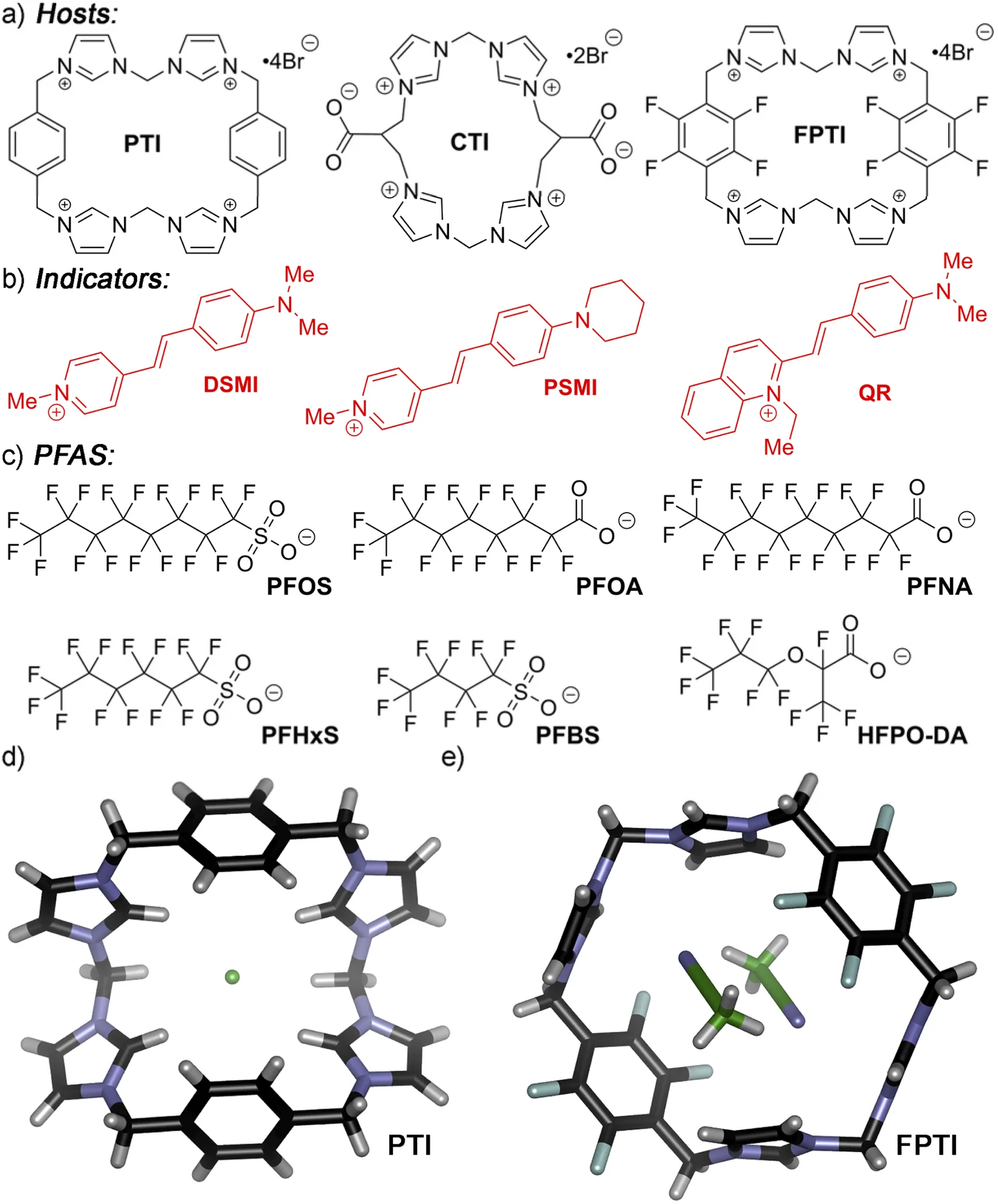
Structures of (a) imidazolium receptors, (b) indicator dyes, and (c) PFAS targets used in this study. scXRD structures of (d) PTI·Cl^51^ and (e) FPTI·Br_4_ (CCDC 2551513, counterions omitted for clarity).

The initial task was to assess whether the XTI macrocycles would indeed recognize different PFAS at low concentrations in water. Toward this end, we used an indicator displacement assay approach since it was expected to provide much lower detection limits than, for example, NMR spectroscopy. PTI has previously been shown to bind and quench the emission of cationic styrylpyridinium indicator dyes (DSMI, PSMI, QR, Fig. 1b) and do so with notably strong affinities (*K*_d_ = 90–400 nM).^51^ To determine the most effective detection system, we also tested the indicator recognition capabilities of FPTI. Surprisingly, anionic indicators such as Methyl Red, Methyl Orange and Indigo Carmine displayed no noticeable interaction (either fluorescence emission quenching or enhancement) with the tetracationic FPTI (Fig. S10). However, cationic dyes, such as DSMI and PSMI, produced detectable levels of quenching (*i.e.*, ∼20% at 10 µM of the macrocycle) when FPTI was titrated into an aqueous solution of the dye (0.5 µM, 20 mM Tris–HCl buffer, pH = 7.4, Fig. S11). The quenching was less effective than what was seen in the case of PTI, but still adequate to provide an operational indicator displacement assay. Thus, with a suite of receptors, *i.e.*, PTI, FPTI, and CTI, and several indicator dyes (DSMI, PSMI, QR) in hand, it proved possible to test as putative guests six anionic PFAS, namely HFPO-DA, PFBS, PFHxS, PFNA, PFOA, and PFOS (see Fig. 1c for structures).

The six different PFAS (0–20 µM) were titrated into mixed solutions of receptors (PTI, FPTI, or CTI, 4.0 µM) and dye (DSMI, PSMI, or QR, 0.5 µM) in 20 mM Tris–HCl buffer. The titration data is shown in Fig. 2 and S13–S15. Two conclusions can be drawn from the data. First, the host : dye pairings are sensitive detectors that show variable selectivities for different PFAS. The relative selectivity and fluorescent enhancement vary with the specific host : dye combinations; however, in each case, the detection is selective for PFOS over the other five tested PFAS. The emission enhancement is strong – addition of 10 µM PFOS to the CTI·QR pair causes a 7-fold increase in emission, for example. The greatest selectivity is seen for FPTI·DSMI, which produces up to a 6-fold enhancement in the presence of PFOS, but minimal enhancement in the presence of all other PFAS.

**Fig. 2:**
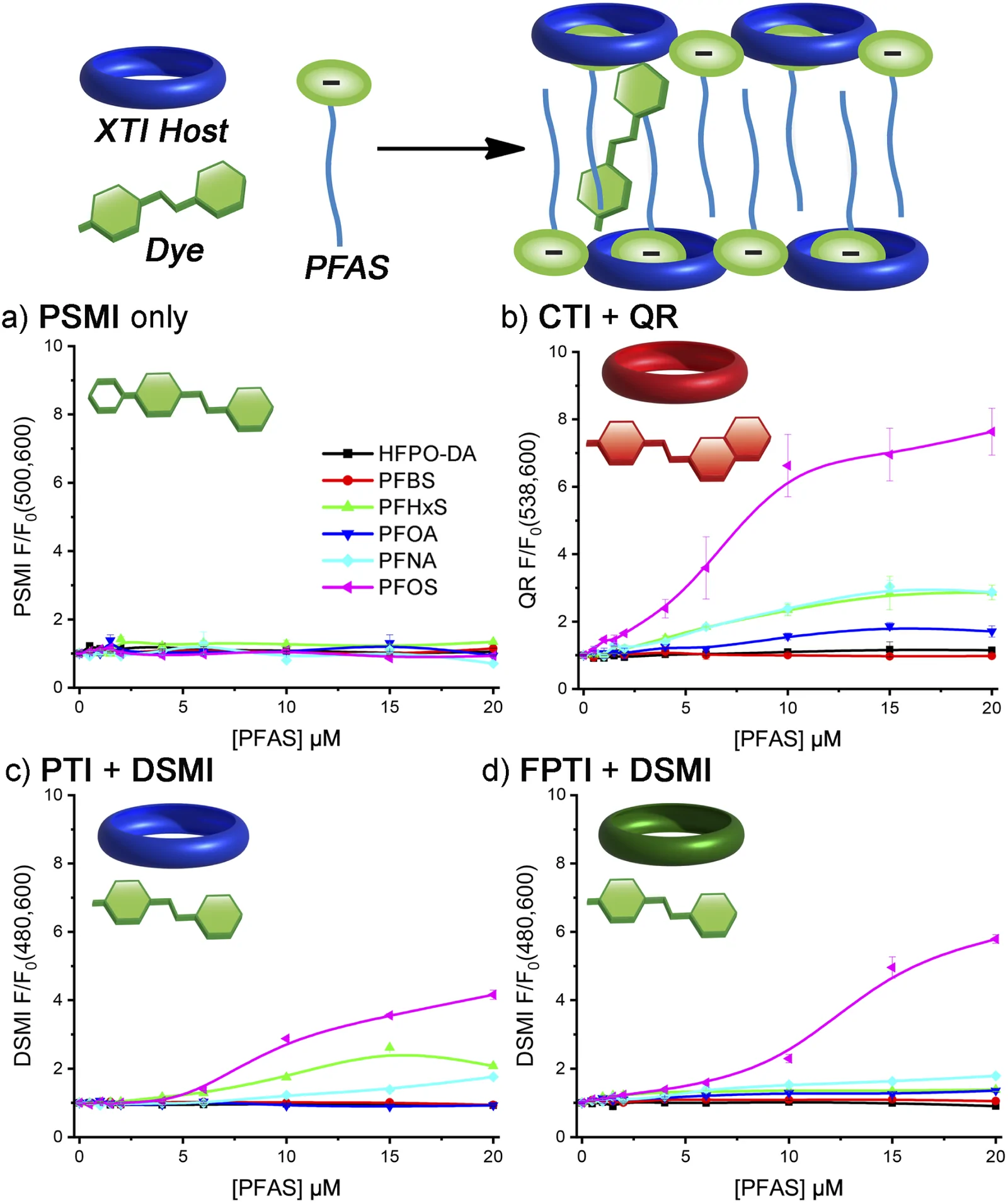
Fluorescence titrations of different PFAS (0–20 µM) into solutions of (a) PSMI dye only, (b) CTI + QR, (c) PTI + PSMI, (d) FPTI + DSMI in 20 mM Tris buffer, pH = 7.4. [XTI] = 4 µM, [dye] = 0.5 µM, *F*_0_ = emission in the absence of PFAS.

The second conclusion we draw from the data is that the detection mechanism is not a simple indicator displacement assay. This is based on the finding that the addition of PFOS to the various host : indicator combinations raises the emission of dye above that of dye alone. While DSMI is quenched by PTI, for example, addition of PFOS does not merely return the indicator emission to its native, unquenched state, it enhances it further. This would not be observed were a simple indicator displacement process at play. Additionally, CTI causes essentially zero quenching of DSMI and PSMI at pH 7.4, while the addition of 20 µM PFOS causes an 8–9-fold enhancement in the emission of the CTI·DSMI and CTI·QR pairs. It is important to underscore that the detection is quite selective for PFOS – carboxylate variants such as PFOA or HFPO-DA gave no response with any of the host : dye combinations, and shorter sulfonates such as PFBS were also not detected. The other PFAS that produced an enhanced emission were PFHxS and PFNA, and in each case the F/F_0_ was far smaller than generated by PFOS. On this basis, we suggest that two factors at minimum underscore the observed selectivity, namely the nature of the headgroup and the length of the fluorous tail. First, sulfonate-containing PFAS caused a greater emission increase than the corresponding carboxylates. Second, the emission response was found to depend on the fluorous chain length. Evidently, PFOS occupies a “sweet spot”, combining both long fluorous chain recognition and sulfonate anion complexation. However, the response is evidently complex and is not simply dependent on greater affinity of sulfonates for the macrocycle; rather, it involves multiple factors, including self-assembly and desolvation of both headgroup and fluorous tail.

To quantitate the sensitivity of the inferred PFOS recognition, the limits of detection (LOD) were determined. The LOD of PFOS by a series of XTI dye pairs were measured in Tris–HCl buffer at pH = 7.4, see Fig. S26 for full details. The most sensitive detection was seen with the CTI·PSMI combination, with the LOD for PFOS being 48 nM, 2.4 × 10^4^ ng L^−1^. This is highly competitive with the best macrocyclic PFOS detection systems – for example, Guo reported LOD (PFOS) = 21.4 nM using cationic calixarene hosts.^38^

The present detection approach is not limited to aqueous buffer; it also proved effective in other complex matrices, such as real-world water samples, beverages and biofluids. The LOD of PFOS with the CTI·DSMI pairing was determined in 10% human saliva. As might be expected given the presence of additional ions and extravascular fluids that can compete with PFOS for the receptor, a drop in sensitivity was observed. Nevertheless, a LOD for PFOS of 970 nM was still seen. The detection was also monitored in a 10% artificial urine sample, which consists of phosphates, alkali metals, and other anions at the levels found in normal human urine (see Fig. S26).^55^ The presence of these anions again lowered the detection response compared to simple buffer, but the CTI·DSMI combination was still capable of efficient detection, with PFOS LOD = 164 nM being found. The various sensor combinations were also applied to real world water samples. As PFAS are often present in a plethora of aqueous media, we tested 12 different water extracts from the Southern California food and water supply. Samples of sea, lake and drinking water were acquired from multiple sources in Southern California (see SI for details) and spiked into Tris buffer at an additive ratio of 10%. The samples were then analyzed by combinations of the three XTI receptors considered in this study in conjunction with two dyes, DSMI and PSMI (Fig. S27 and S28). The general trend was similar to that of the urine/saliva tests, in that the fluorescence emission changes were lower than in pure buffer, presumably due to the presence of interferents. Nevertheless, in each case, enhanced emission upon addition of 20 µM PFOS was seen. Some notable data points were that CTI was far less affected by interferents than the other receptors, and FPTI was the least effective receptor. PSMI responses were higher than those for DSMI in most cases. Certain combinations proved highly effective – CTI·DSMI gave the same emission response change for PFOS in spiked Lake Perris water as in pure buffer, as did PTI·PSMI for Laguna Beach sea water. The detection systems were also tested using commercial beverages – 8 different beverages were spiked into buffer using the same method as above, and the XTI dye responses tested (Fig. S27 and S28). In this case, dark beverages such as Coke, containing a high proportion of organic species, were least tolerated; however, as with the real-world water samples, the XTI dye suite was capable of detecting PFOS in all samples, despite the presumed presence of multiple different interferents. LODs were determined for the Balboa Beach water and GFuel spiked samples with the CTI·DSMI sensor. Only modest declines were seen as compared to what was seen in pure buffer (LODs = 249 nM and 117 nM respectively, Table S2). Finally, extracts from citrus leaves were tested as an interferent, as PFAS have been found to bioaccumulate in those samples.^56^ Citrus leaves were taken from Washington Navel orange trees, extracted with CH_2_Cl_2_ followed by solvent removal and dissolution of the residue into dimethoxyethane : water before the samples were spiked into Tris as before (see SI for details). The LOD for the CTI·DSMI/CTI·PSMI sensors were determined (Fig. S30). The LOD for PFOS with each sensor was slightly less favorable than in pure buffer, but still low (LODs = 176 nM and 152 nM for CTI·DSMI and CTI·PSMI, respectively; Table S2). The variability in response also illustrates the complexity of the unique assembly mechanism – detection is not *via* a simple indicator displacement process that can be interrupted by a single type of interferent. These complexities notwithstanding, the present detection protocol proved highly tolerant and could be applied to various real-world matrices, underscoring its potential utility.

To understand in greater detail the PFOS·XTI recognition mechanism, and the unusual behavior of the XTI : indicator pairings, the interactions were analyzed by ^1^H and ^19^F NMR spectroscopy. Solutions of macrocycles (PTI, FPTI or CTI, 1.0 mM in D_2_O) were titrated with increasing concentrations of PFOS (0–2 mM). Analysis of the ^1^H NMR spectra showed a loss of signal for the macrocycles upon increasing concentration of PFAS, as well as the production of a visible solid precipitate almost immediately after addition (Fig. 3a and b). No changes in the chemical shift for the macrocycle peaks were observed, simply a disappearance of peaks until the point that 2.0 mM PFOS had been added, at which point none of the ^1^H signals remain visible in the spectrum (Fig. S31–S37). To visualize the effect on PFOS, inverse titrations, wherein PFOS (1 mM) was titrated with the XTI hosts (0–1 mM), were also performed. Analysis of the ^19^F NMR signals of the test PFAS as a function of increasing XTI host concentration revealed that in the case of PFOS, the internal –CF_2_ signals are no longer visible after addition of 0.5 mM PTI. On the other hand, the signal for the terminal –CF_3_ group does not disappear; rather, it shifts from a sharp singlet at −81 ppm to a broader peak resonating at −83 ppm. The selective loss of the –CF_2_ signals, retention of the –CF_3_ signal, and the precipitation seen during these spectral titrations lead us to suggest the formation of large aggregates wherein fluorous chains are disordered. This disorder and less efficient packing near the tail ends provide the –CF_3_ groups with a relatively greater degree of freedom, allowing for a well-defined ^19^F NMR signal. PFOA was also tested for its ability to undergo aggregation at the millimolar concentrations used for the NMR spectral analyses (Fig. S38–S44). Results similar to those obtained with PFOS were observed. Evidently, the XTI hosts can precipitate multiple PFAS at the relatively high concentrations required for the NMR spectral analyses. On the other hand, the selectivity for PFOS over the other PFAS is enhanced at the micromolar concentrations relevant for the fluorescence-based assays, since under these conditions the relative solubility effects are magnified.

**Fig. 3:**
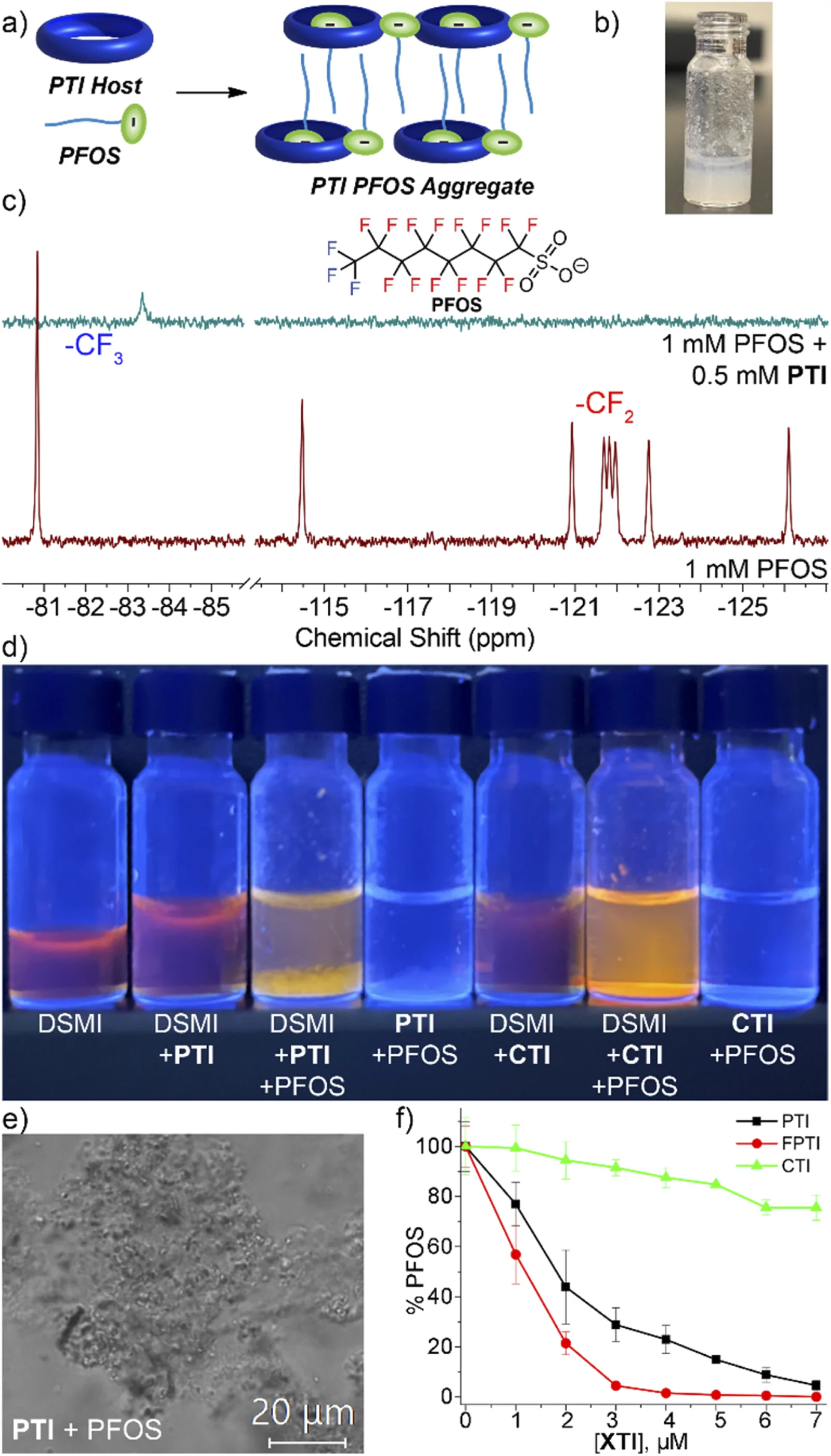
(a) Illustration of XTI·PFOS aggregation; (b) image of precipitate formed after 2 mM PFOS was added to 1 mM PTI in H_2_O; (c) ^19^F NMR spectra of 1 mM PFOS and a mixture of 1 mM PFOS + 0.5 mM PTI. (d) Images of host (4.0 mM)/dye (0.5 mM)/PFOS (8 mM) solutions in H_2_O under a 365 nm lamp; (e) optical microscope image of the PTI·PFOS precipitate. (f) Relative concentration data (determined *via* LC-MS analysis of various aliquots), showing removal of PFOS from a 10 µM solution in H_2_O *via* the addition of various concentrations of an XTI-type host.

The XTI·PFOS aggregates were further studied by microscopy and dynamic light scattering (DLS). Optical microscopy images (Fig. 3e) revealed the formation of large micron-sized aggregates that do not possess a defined structure. The results of the DLS analyses proved concordant with the microscopy images and supported the difference in behavior seen for the three different macrocycles (for DLS data, see Fig. S50–S54 and Table S3). The PTI·PFOS aggregates were found to average 1000 nm in diameter. On the other hand, the CTI·PFOS aggregates proved to be much smaller, with an average diameter of 28 nm. The FPTI·PFOS aggregates were found to lie between, with an average diameter of 170 nm being seen. These data support the inference drawn above, namely that the differences in the fluorescence response ascribed to aggregation are not simply driven by the PFAS molecule, but a subtle interplay between the receptor and the aggregate controls the size of the aggregate formed upon complexation. These experiments are thus consistent with the postulate that the XTI receptors bind the headgroup of the PFAS, which triggers aggregation in water, which is observed as precipitation at millimolar concentrations. Left undetermined by these studies is why an additional enhancement of the dye emission is seen in the PFOS·XTI ensembles.

To determine where the dye locates upon aggregation, PFOS (2.0 mM) was added to a mixture of PTI (1.0 mM) and DSMI (0.5 mM) in D_2_O (Fig. S45 and S48). In this case, an orange precipitate was formed. This precipitate proved emissive when placed under a UV lamp (Fig. 3d). This finding is taken as evidence that the DSMI dye is located within the aggregate. ^1^H and ^19^F NMR spectral analysis of the solution revealed no detectable signals for PFOS, PTI or DSMI in the aqueous phase, leading us to infer that all the dye was extracted into the aggregate, at least to the limits of NMR spectral detection. This observation is consistent with the fluorescence results – the styrylpyridinium dyes are solvatochromic^57^ and show enhanced emission in hydrophobic environments. Upon formation of the host : PFOS aggregate, the dyes are likely displaced from the XTI macrocycle cavity by the PFOS but are then captured within the fluorous phase of the aggregate. This gives rise to an enhanced emission relative to what is seen for the dye alone in aqueous media.

The visible and rapid aggregation between the XTI macrocycles and PFOS (occurring almost immediately after mixing) led us to consider that this system would prove effective for the removal of PFOS from water. To test this possibility, aqueous solutions of PFOS (10 µM) were treated with increasing concentrations (0–7 µM) of PTI, FPTI, and CTI. The resulting mixtures were filtered through a 0.22 µm MCE filter to remove the resulting insoluble aggregate. The remaining PFOS was then quantified *via* LC-MS analyses (Fig. 3f). Both FPTI and PTI proved to be effective at removing the PFOS *via* this precipitation and filtration approach. Moreover, the PFOS removal proved to be submolar in macrocycle. For instance, the addition of only 1 µM PTI to a 10 µM PFOS solution allowed 50% of the original PFOS to be removed, yielding a final [PFOS] = 4 µM. Evidently the tetracationic macrocycle coordinates multiple mono-anionic PFOS molecules within each aggregate. This simple experiment should not be interpreted as implying a specific PTI : PFOS stoichiometry, simply that the aggregate contains more molecules of PFOS than receptor. The most efficient removal occurred when the added macrocycle concentration reached 7 µM, 3.5 × 10^6^ ng L^−1^, and after a 30 min delay to allow full precipitation (Fig. 3f, Tables S5 and S6). For instance, a single mix-and-filter experiment with PTI removed 95% of the PFOS originally present in the solution. The fluorinated congener FPTI proved even more efficient, effecting 99.8% PFOS removal after a single treatment. The efficiency of PFOS removal by FPTI is competitive with other solution-phase macrocycle-mediated PFOS extraction methods.^50^ While there are other, putatively more effective extraction systems reported (*e.g.* Zuilhof's pillararenes^32^ or Guo's calixarenes^38^), they are all solid state extractants, and require covalent immobilization on a solid support to enable PFAS removal. In contrast, the present system achieves 99.8% PFOS removal in a simple mix/filter experiment. As such, it does not require organic solvents as typical for liquid : liquid extraction protocols. It is based on a water-soluble macrocycle with no need for derivatization or immobilization on a solid support. We believe that the present approach thus embodies an attractive mix of synthetic simplicity and facile extraction chemistry while permitting the rapid and efficient removal of PFOS from various aqueous source phases.

In contrast to PTI and FPTI, CTI proved relatively less effective as a precipitation-based PFOS removal agent. In the case of this macrocycle, the filtration-based removal efficiency plateaus rapidly with <25% PFOS being effectively removed. This finding is rationalized in terms of the DLS results, which revealed that CTI forms smaller aggregates than PTI or FPTI (*vide supra*). In the case of CTI, aggregation still occurs; however, the aggregates are not efficiently removed with a 0.22 µm filter, as on average they are smaller than the aperture. Considered in concert, these results support the conclusion that appropriately chosen cationic macrocycles, such as PTI and FPTI, will form large, disordered host : PFOS aggregates in aqueous media. These aggregates can incorporate hydrophobic dyes thus enhancing their emission and allowing for PFOS detection. The aggregates can be removed from the aqueous medium through simple filtration, allowing for the effective removal of the PFOS.

To understand in greater detail the nature of the assembly process and the aggregate structures, we turned to computational methods. Analysis of the molecular electrostatic potential of the three XTI polycations shows that the charge deficiency (*i.e.*, repulsive interaction for a positive probe charge) is delocalized fairly well over the entire macrocycle surface (see Fig. S61), indicating the absence of a well-defined binding pocket. A similar analysis reveals that the negative charge on the sulfonate headgroup is more delocalized than on the carboxylate headgroup. A series of 18 host : PFAS : dye combinations (see Table S10) were probed through molecular dynamics simulations in the isothermal–isobaric ensemble (*T* = 300 K and *P* = 1 bar). For each combination, three independent trajectories were run. In most cases, the simulated systems contained 56 000 water molecules, 16 XTI cations, and 64 PFOS or PFBS anions (16 and 64 mM), *i.e.*, concentrations about one order of magnitude larger than in the aggregation experiments. This allows for manageable aggregate sizes of a few nm instead of the micron-sized aggregates found in the DLS measurements. Additional simulations were carried out for PTI : (PFOS or PFBS) to explore the effects of concentration, system size, and non-stoichiometric ratios. The time required to yield converged nano-aggregate size distributions depends on the degree of super-saturation, the system size (a longer diffusion path is required for larger systems), and the solute species, with increased charge of the macrocycle and longer fluorous PFAS tail increasing the driving force. For the majority of systems with 56 000 water molecules (box length of 12 nm), a 100-ns simulation period (requiring about 40 hours of wall-clock time using 8 CPU cores and one GPU) is sufficient to yield equilibrium structures (see Fig. S62–S67). However, for the CTI : PFOS, PTI : PFBS, and FPTI : PFBS systems, 300 ns were found to be insufficient to reach a converged aggregate size distribution.

Radial distribution functions calculated only over the last 20 ns of the trajectories (see Fig. S68) allow us to find suitable site–site distance criteria to define a pair of sites to belong to the same aggregate. Here, we use 12 Å as an upper bound for the sulfur–sulfur distance for a pair of PFAS anions (while there is a well-defined peak for CF_3_–CF_3_ contacts in large aggregate, using the corresponding first minimum does not work well to describe the initial aggregation process) and 10 Å as an upper bound for the S-center-of-mass distance between a PFAS anion and an XTI macrocycle. (This distance criterion holds for both the tetra- and dicationic receptors of this study.) The center-of-mass radial distribution function for a pair of macrocycles does not lend itself to a well-defined cluster criterion, but using a “neighbors-of-neighbors” search algorithm allowed aggregates containing multiple macrocycles to be readily defined.

Snapshots of the final configuration observed for PTI : PFOS : PSMI, PTI : PFBS, and NaPFOS are shown in Fig. 4. A single, “football”-shaped aggregate is found for PTI : PFOS : PSMI (Fig. 4a) and only intermittently does a PTI or PFOS ion leave the aggregate. In this aggregate, the PTI cations are found on the surface, the PFOS headgroups are in contact with PTI or water, and the PFOS tails form a disordered fluorous core. The non-polar end of the dye is preferentially located in the core of the aggregate (as indicated by its proximity to terminal CF_3_ units), and its pyridinium ring is found closer to the PFOS sulfonate groups and, to a lesser extent, water molecules (see Fig. S69–S71). When PTI is replaced by FPTI, or the dye molecules are omitted, the simulations yield similar outcomes as for PTI : PFOS : PSMI (see Fig. 4d and S72–S74). Interestingly, the final outcome (*i.e.*, a single “football”-shape aggregate containing essentially all PTI and PFOS ions) is not affected when the PTI and PFOS concentrations are increased from 8 and 32 mM to 16 and 64 mM, nor when the system size is doubled (see Fig. S72 and S75). However, when PFOS is replaced by the less hydrophobic PFBS, the aggregation behavior is concentration dependent. For the PTI : PFBS system at the lower concentration (8 and 32 mM, respectively), a large number of small aggregates is formed, each containing on one or two PTI cations and up to six PFBS anions, as well as many non-aggregated PFBS anions (Fig. 4b). When the concentration is doubled, then one larger aggregate is formed that, on average, contains at least half of the PFBS anions, with the remaining PFBS anions found in smaller aggregates or free in solution (Fig. S76). The simulation length of 300 ns is not sufficient to establish whether these smaller aggregates will merge with the larger aggregate to form a single aggregate as for PTI : PFOS systems. Nevertheless, the significant fraction of non-aggregated PFBS anions and the longer induction periods for aggregate formation (despite the faster diffusion of PFBS) are taken as evidence that aggregation is not solely driven by electrostatic attraction to the tetracationic PTI, but that solvatophobic effects involving the PFOS fluorous tails help drive aggregation. Similarly, for NaPFOS, the simulation results in multiple smaller aggregates containing 10 to 16 PFOS anions (Fig. 4c), whereas the largest aggregate for NaPFBS averages 3 PFBS anions (Fig. S77). However, when the tetracationic receptors PTI and FPTI are replaced by CTI, a dicationic congener, a single large aggregate is formed after 150 ns in all three simulations carried out for the system. This aggregate contains 16 mM CTI and 32 mM PFOS in a defined stoichiometric ratio (Fig. S78). The longer induction period concords with the DLS finding that much larger aggregates form with PTI than with CTI.

**Fig. 4:**
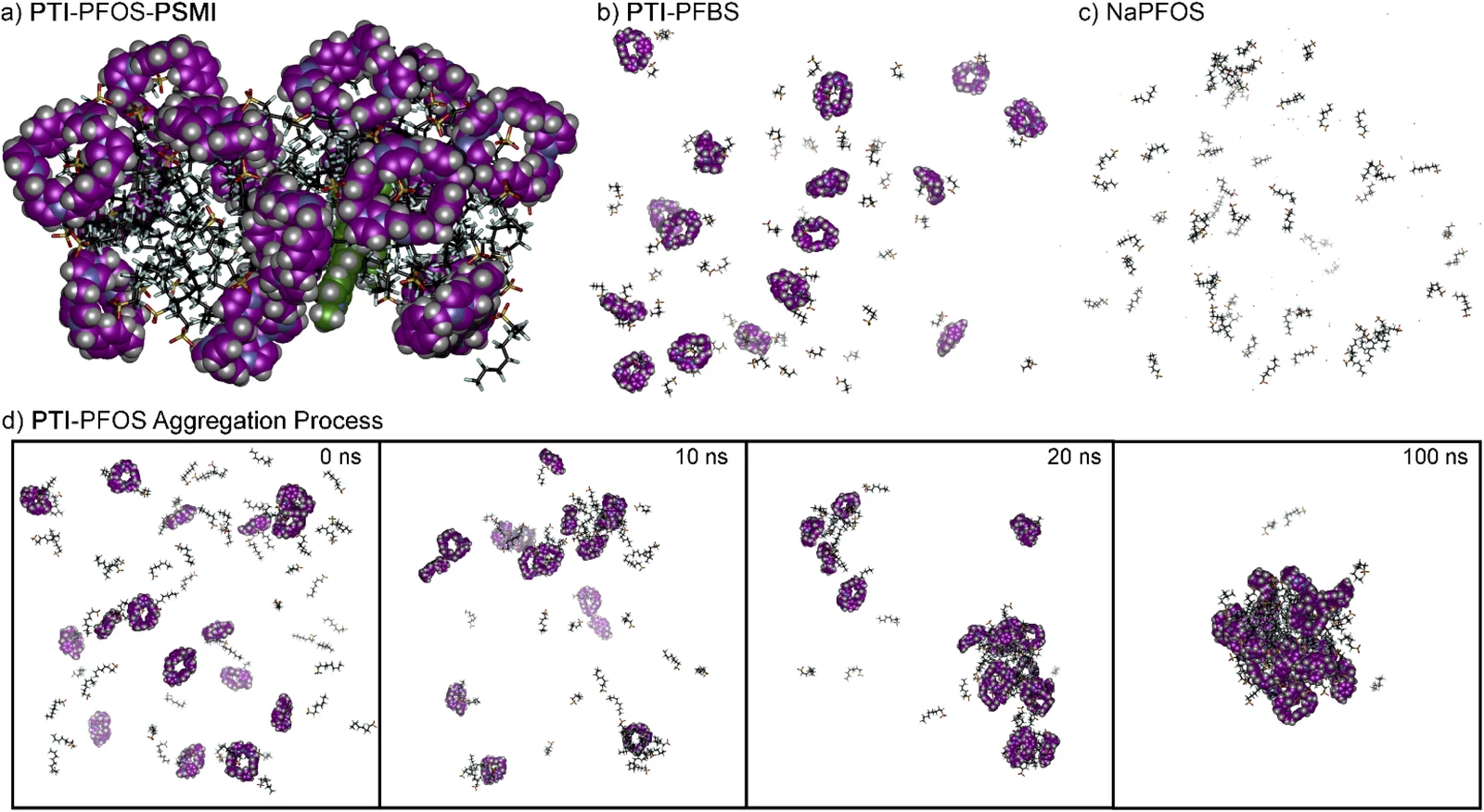
Snapshots of the final configuration found in simulations for (a) PTI : PFOS : PSMI (16 : 64 : 2), (b) PTI : PFBS (8 : 32), and (c) NaPFOS (64). All concentrations are given in units of mM (corresponding to the same number of molecules for simulations with 56k water molecules). (d) Time series illustration of the aggregation process for the PTI : PFOS system (8 : 32) obtained over the first 100 ns of the NPT-trajectory after a 2-ns pre-equilibration period. PTI macrocycles and dye molecules are shown as spheres (using purple and green for their C atoms), while PFOS anions are shown as wireframes (using yellow and red for S and O atoms, respectively). Water molecules are omitted for clarity; images only show the part of the simulation box that contains PTI or PFOS molecules.

Analysis of the simulation trajectories also provides molecular-level insights into a plausible aggregation mechanism at concentrations accessible to the simulations. Fig. 4d illustrates the aggregation process for the PTI : PFOS system without dye molecules. Even during the 2-ns pre-equilibration period, the strong electrostatic attraction between the tetracationic receptor and PFOS leads to sub-stochiometric aggregates containing a single receptor and one or two PFOS anions. At the beginning of the 100-ns simulation trajectory (*t* = 0 ns), only about one-third of the PTI cations remain isolated, and the others are, with about equal probability, associated with one or two PFOS anions (Fig. S62). The weaker electrostatic repulsion between these pre-aggregates (as PFOS neutralizes part of the PTI charge) can then be surmounted by solvatophobic driving forces between PFOS tails, leading to the formation of small and rather disordered micelle-like objects containing a few PTI macrocycles (*t* = 10 ns). Over this 10-ns period, the fraction of PTI cations with one or two PFOS within the aggregation criterion decreases, while the fraction of PTI associated with three PFOS rises sharply, and that with four PFOS increases more slowly. Over the next 10 ns, the micelles continue to grow and assume a well-defined core–shell morphology with the PTI occupying the shell and the fluorous tails the core region. At *t* = 20 ns, about 90% of the PTI are found in these micelle-like objects, with some PFOS anions not being part of the aggregates. In related work,^58^ MD simulations probing the adsorption of PFOA in a cationic covalent organic frame COF, it was likewise found that rapid binding to the COF surface is followed by PFOA aggregation through solvatophobic interactions. PFOA molecules were also found to rapidly bind to preformed micellar aggregates of cationic fluorinated polymers and subsequently form the core of swollen aggregates.^59^

As the micelle-like objects grow (mostly through micelle–micelle fusion and not through transfer of aggregates containing only one macrocycle), the volume-to-area ratio increases. This allows for the incorporation of additional PFOS molecules until a stoichiometric ratio is reached. At this point, about 80% of the PTI cations in the large aggregate are associated with four PFOS anions. For the CTI : PFOS combination, simulations were also carried out with 16 CTI and 64 PFOS molecules (*i.e.*, twice the stoichiometric ratio but the same PFOS concentration as for the tetracationic receptors, Fig. S79 and S80). Even in this case, the fraction of unassociated PFOS drops to less than 10% with the majority of the simulations resulting in the formation of two aggregates. It is inferred that the dense core of the larger micelles provides a favorable environment for PFOS beyond the stoichiometric ratio (see also Fig. S81 for supra-stochiometric aggregation of PFOS with PTI). Notably, in these supra-stoichiometric aggregates, the charge is compensated by the presence of Na^+^ cations in the vicinity of the aggregate surface.

As the driving force for the assembly involves formation of a hydrophobic aggregate, we next sought to explore how hydrocarbon lipids might be affected by the present series of XTI hosts. Accordingly, sodium octanoate and sodium dodecyl sulfate (0–20 mM) were titrated into a solution of FPTI (1 mM). No obvious precipitation was observed and only minimal peak shifts in the ^1^H NMR spectrum were seen (Fig. S46 and S47). This lack of formation of an insoluble aggregate is ascribed to the fact that hydrocarbon lipids are more soluble than PFAS. For example, the solubility of PFOS (680 mg L^−1^ at 25 °C) is far lower than SDS (200 g L^−1^ at 25 °C).^60^

We then tested the system on pre-aggregated lipids (Fig. 5). In this study, extruded unilamellar vesicles/liposomes of palmitoyl-oleylphosphocholine (POPC) were formed *via* a literature procedure.^61^ These vesicles were added to solutions of either DSMI or PSMI, as well as dye : host mixtures (Fig. 5a and b). Minimal changes in emission, and even slight quenching were seen in these cases. This was taken as evidence that the charged XTI hosts and dyes are not sufficiently hydrophobic to be well-incorporated into the vesicles. In contrast, when the various PFAS were titrated into dye : host : POPC vesicle mixtures, significant enhancement of the dye emission signal was seen, relative to that seen for the corresponding dye : host mixtures in aqueous buffer. For example, the maximal fluorescence emission enhancement for PFOS by CTI·DSMI is *F*/*F*_0_ = 8.2 upon addition of 20 µM PFOS (Fig. 5c). However, when the same experiment is performed in the presence of 132 µM POPC vesicles, the *F*/*F*_0_ maximum increases to 21.9 at 20 µM CTI (Fig. 5d). The presence of lipid vesicles does not disrupt the hydrophobic assembly formation; rather, it favors it and enhances the emission response. This enhancement occurs with no loss in the PFOS selectivity. In fact, the enhancement in emission produced by PFOS in the CTI·DSMI : POPC mixture is greater than that produced by PFHxS and PFNA. The FPTI·DSMI pair demonstrated an increased fluorescence response for PFOS (10-fold), double that seen in the absence of lipids (Fig. S19). Overall, introduction of low concentrations of lipid vesicles increased the detection response of the XTI dye systems, with no loss in PFAS selectivity.

**Fig. 5:**
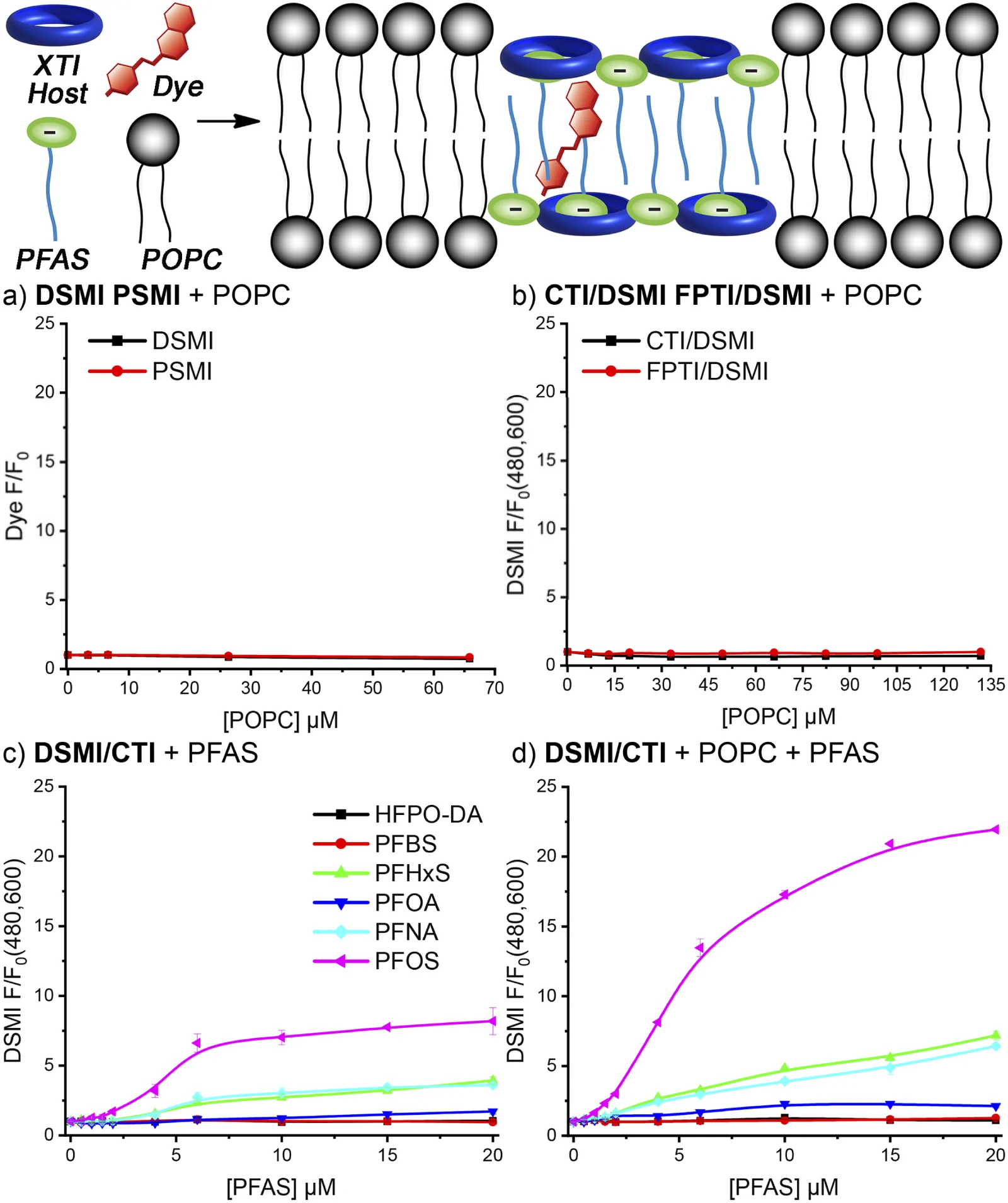
Fluorescence titration plots showing the effect of POPC lipids on PFAS detection. (a) Dyes alone; DSMI/PSMI + POPC, (b) dye/macrocycle complexes; CTI/DSMI + POPC, FPTI/DSMI + POPC; (c) dye/macrocycle/PFAS complex in the absence of lipids; CTI/DSMI + PFAS; (d) dye/macrocycle/PFAS complex + lipids; CTI/DSMI + POPC + PFAS. 20 mM Tris–HCl buffer, pH = 7.4, [XTI] = 4 µM, [dye] = 0.5 µM, [POPC] = 132 µM, *F*_0_ = emission in the absence of PFAS.

## Conclusions

In this study, we have shown that a simple set of cationic macrocycles can recognize PFAS in aqueous solution. The recognition is driven in large measure by interactions between the cationic macrocycles and the headgroups of the PFAS, which “masks” the anionic charge. This leads to aggregation, and precipitation at higher concentrations. The PFAS selectivity in optical detection is controlled by a complex combination of macrocycle–PFAS headgroup interactions, fluorous tail size and desolvation upon aggregation. PFOS, containing sulfonate headgroups and a long fluorous tail, appears to lie at a “sweet spot” amongst the common PFAS where these two recognition effects synergize most effectively at the low concentrations used for the optical experiments. When indicator dyes are added to the host : PFOS mixture, they are incorporated into the hydrophobic aggregate, and the solvatochromic response enables detection of PFOS at LOD = 48 nM in buffered solution. The detection is functional in a variety of aqueous media, including human saliva, artificial urine, drinking water, commercial beverages and citrus extracts, and can be enhanced by pairing the receptors with hydrophobic POPC lipid vesicles. The aggregation process enables the removal of PFOS from water *via* a simple filtration process. Using the cationic PTI host, 95% removal of PFOS is possible from an initial 10 µM aqueous solution with a single treatment and at a substoichiometric concentration of the host. Changing the nature of the host changes the size of the XTI·PFOS aggregates, which affects the filtration-based purification efficacy. For instance, the zwitterionic CTI forms much smaller aggregates, reducing the effectiveness of the filtration step. In contrast, use of the fluorous FPTI host increased the efficacy of the removal to 99.8% under otherwise identical conditions. The aggregation process was analyzed through molecular dynamics simulations (at higher concentrations) and found to progress *via* a three-step mechanism involving (1) very rapid electrostatically-driven formation of small ensembles containing one macrocycle and one or two PFOS molecules, followed by (2) formation of disordered assemblies containing multiple macrocycles, and (3) formation of well-structured core–shell micelles that grow to larger aggregates.

Overall, these receptor systems provide a simple and efficient approach to the recognition, optical detection, and removal of PFOS from complex aqueous media. Combining anion recognition in water with the solvatophobic properties of fluorocarbon chains is key to the efficacy and simplicity of the process.

## Author contributions

Conceptualization: A. C. Garcia, R. Lian, S. K. Paul, J. L. Sessler, J. I. Siepmann, R. J. Hooley. Investigation: A. C. Garcia, R. Lian, S. K. Paul, X. Jin, A. Seth, S. Venkatesan, H. Zhang, Y.-D. Yang, M. Awan, P. S. A. Benedict, V. Rajasekaran, V. Carta, J. L. Sessler, J. I. Siepmann, R. J. Hooley. Writing – original draft: A. C. Garcia, R. Lian, S. K. Paul, X. Jin, J. L. Sessler, J. I. Siepmann, R. J. Hooley. Writing – review and editing: A. C. Garcia, R. Lian, S. K. Paul, X. Jin, A. Seth, S. Venkatesan, H. Zhang, Y.-D. Yang, M. Awan, P. S. A. Benedict, V. Rajasekaran, V. Carta, J. L. Sessler, J. I. Siepmann, R. J. Hooley. Methodology: A. C. Garcia, R. Lian, S. K. Paul, X. Jin, A. Seth, S. Venkatesan, H. Zhang, Y.-D. Yang, M. Awan, P. S. A. Benedict, V. Rajasekaran, V. Carta, J. L. Sessler, J. I. Siepmann, R. J. Hooley. Validation: A. C. Garcia, R. Lian, S. K. Paul, X. Jin, A. Seth, S. Venkatesan, H. Zhang, Y.-D. Yang, M. Awan, P. S. A. Benedict, V. Rajasekaran, V. Carta, J. L. Sessler, J. I. Siepmann, R. J. Hooley. Supervision: J. L. Sessler, J. I. Siepmann, R. J. Hooley. funding acquisition: J. L. Sessler, J. I. Siepmann, R. J. Hooley. Project administration: J. L. Sessler, J. I. Siepmann, R. J. Hooley.

## Conflicts of interest

There are no conflicts to declare.

## Supplementary Material

SC-OLF-D6SC05991K-s001

SC-OLF-D6SC05991K-s002

## Data Availability

CCDC 2551513 contains the supplementary crystallographic data for this paper.^62^ The data supporting this article have been included as part of the supplementary information (SI). Supplementary information: new molecule synthesis and characterization, fluorescence data, molecular dynamics analysis, and statistical methods. See DOI: https://doi.org/10.1039/d6sc05991k.
