# Supplementary material for: Perfluorooctanesulfonate detection and removal with simple imidazolium macrocycles
Source: Chem Sci. 2026 Oct 27. Online ahead of print. doi: 10.1039/d6sc05991k (PMC13725869; doi:10.1039/d6sc05991k)
Supplement: Supplementary file 1 [file SC-OLF-D6SC05991K-s001.pdf]

Supplementary Information (SI) for Chemical Science.
This journal is © The Royal Society of Chemistry

Selective Perfluorooctanesulfonate Detection and Removal with Simple Imidazolium Macrocycles

Arman C. Garcia[1], Ria Lian[1], Suranjan K. Paul[2], Xingchen Jin[3], Anika Seth[1], Sruthi Venkatesan[1], Hanfang Zhang,[3] Yu-Dong Yang,[3] Momena Awan,[1] Paula Sharon Antony Benedict,[1] Varshitha Rajasekaran,[1] Veronica Carta[1], Jonathan L. Sessler[3]*, J. Ilja Siepmann[2]*, Richard J. Hooley[1]*

[1]Department of Chemistry; University of California-Riverside, Riverside, CA 92521, U.S.A.
[2]Department of Chemistry and Chemical Theory Center, University of Minnesota–Twin Cities, Minneapolis, Minnesota 55455, United States
[3]Department of Chemistry, The University of Texas at Austin, Texas 78712, United States

E-mail: richard.hooley@ucr.edu; siepmann@umn.edu; sessler@cm.utexas.edu

Electronic Supplementary Information

Table of Contents

1. General Information……………………………………………………………….......S-3
1.1 Chemical and Reagents……………………………….....................................…....S-3
1.2 Experimental Methods ....……………………………………….........................S-6
1.3 Chemical Synthesis and Characterization……………………………….…….S-11
2. Characterization Data…………………………………………………………….............S-12
2.1 NMR Spectral Characterization of New Molecules......………………….....…....S-12
3. Fluorescence Spectral Titrations..........................………………………………….......S-17
3.1 FPTI-Dye Titrations...........................……………………………………………S-17
3.2 XTI-Dye + PFAS Titrations...................…........................……………………...S-19
3.3 Dye, XTI-Dye + POPC Titrations.....…......................……………………….........S-22
3.4 XTI-Dye + Biofluid Titrations.....….......................………………………................S-24
3.5 XTI-Dye + PFAS Fluorescence Response Bar Plot Responses.......….........…....S-25
4. ^{1}H and ^{19}F NMR Spectral Titrations ……………………………………….............…S-29
4.1 ^{1}H NMR Spectral Titrations of PTI, FPTI, and CTI with PFOA or PFOS.......S-29
4.2 ^{19}F NMR Spectral Titrations of PTI, FPTI, and CTI with PFOA or PFOS......S-33
5. XTI Aggregate Characterization …………………………………………….............S-39
5.1 Microscopy...S-39

5.2 Dynamic Light Scattering (DLS) Data……………………………………………………S-40
6. PFOS Removal Studies………………………………………………………………………S-44
6.1 LC-MS Data………………………………………………………………………………S-44
7. Computational Details……………………………………………………………………S-46
7.1 Molecular Models…………………………………………………………………………S-46
7.2 Molecular Dynamics Details……………………………………………………………S-55
7.3 System Set-up and Simulation Details…………………………………………………S-56
7.4 Snapshots………………………………………………………………………………S-66
8. References…………………………………………………………………………………S-81

1. General Information

1.1 Chemicals and Reagents

Fluorophores **DSMI**[1] and **PSMI**[2] were synthesized and characterized according to literature procedures. Fluorophores **Quinaldine Red** (**QR**), **Methyl Orange**, **Methyl Red**, **and Indigo Carmine** were purchased commercially from Sigma and used as received. Methylene-diimidazole (MDI) was prepared according to literature procedures.[3] 1,4-Bis(bromomethyl)-2,3,5,6-tetrafluorobenzene was synthesized according to literature procedure.[4] **PTI** and **CTI** were synthesized according to literature procedures.[5] Human saliva was purchased from Lee Biosolution.

Sodium chloride, sodium sulfate, potassium chloride, potassium oxalate, ammonium chloride, magnesium sulfate, and disodium monohydrogen phosphate were purchased from Fisher Scientific. Uric acid and calcium chloride were purchased from Mallinckrodt Chemicals Works. Urea was purchased from Sigma. Trisodium citrate was purchased from Alfa Aesar. Sodium dihydrogen phosphate was purchased from EMD Millipore.

The following perfluoroalkyl substances (PFAS) were purchased from Synquest Labs: Perfluorooctanesulfonic acid (PFOS), perfluorononanoic acid (PFNA), perfluorohexanesulfonic acid (PFHxS), perfluorooctanoic acid (PFOA), nonafluorobutane-1-sulfonic acid (PFBS), and undecafluoro-2-methyl-3 oxahexanoic acid (HFPO-DA).

Artificial urine was prepared according to a literature procedure – sodium sulfate (170 mg), uric acid (25 mg), trisodium citrate (0.72 mg), urea (1.5 g), potassium chloride (230 mg), sodium chloride (175 mg), calcium chloride (19 mg), ammonium chloride (126 mg), potassium oxalate (3.5 mg), magnesium sulfate (108 mg), sodium dihydrogen phosphate (291 mg), and disodium monohydrogen phosphate (83 mg) were dissolved in 100 mL of Ultrapure water. The mixture was stirred and heated at 35°C overnight and filtered through a 0.22 µm PVDF membrane filter.[6]

A liposome extruder set was obtained from Avanti. A Nucleopore Track-Etch Membrane (0.1 µm, 19 mm) and a polyethylene drain disc were both purchased from Cytiva. POPC (16:0/18:1 PC) was purchased from Echelon Bioscience and used as received.

The pyrogen-, nuclease- and bacteria-free ultrapure (Type 1) water was generated using a Direct-Q 3 UV water purification system with a Biopak polisher (Catalog Number CDUFBI001) and used

in the fluorescence emission studies. The pH values of buffers were measured using a Fisher Scientific Accumet Excel XL50 Dual Channel pH/Ion/Conductivity Meter with Fisherbrand Accumet glass body standard size combination electrode, which was pre-calibrated using RICCA Chemical pH buffer reference standard solutions of pH 4.00, 7.00, and 10.00. Solvents were dried through a commercial solvent purification system (Pure Process Technologies, Inc.).

"Real-World" Water Samples: 12 samples were collected in total, and can be separated into 3 broad groupings: **a) Stream/lake/external water sources: Channel water**, **residential water**, and **Big Bear Lake water** were collected in the Big Bear community. Channel water was collected from a drainage easement in the Big Bear community. Residential water was collected in a water drainage system located in the Big Bear suburbs. **Big Bear Lake water** was collected directly from Big Bear Lake. **Brackish water** was collected from water run off alongside the Bolsa Chica Wetlands West Trailhead in Huntington Beach, CA. **Aliso Creek stream** and **Aliso Creek pond water** were collected from a small water runoff in the English Canyon in Lake Forest, CA. Lake Perris water was collected alongside the shoreline of Lake Perris State Recreation Area in Riverside CA. **b) Sea Water: Balboa Beach Pier water** was from the shoreline of Balboa Beach in Newport Beach, CA. **Laguna Beach water** was collected along the shoreline from Laguna Beach, CA. **Scripps water** was collected in the marshland near the UCSD Scripps Pier in La Jolla. **c) Drinking Water: Tap water** and **fountain water** were collected in the Chemical Science Building at UC Riverside. All water samples were collected in a 50 mL Falcon tube and used as collected in analytical studies.

Beverage Samples: 8 commercial beverages were sampled: Caffeine Free Coca-Cola, International Breweries Company (IBC) Root Beer, White Rockstar (Silver Ice, Pure Zero), Red Rockstar (Fruit Punch), G Fuel (Blue Bomber Slushee), Prime (Ice Pop), Mott's Apple Juice, and White Monster were purchased at a local grocery stores and used as received. Each beverage was collected into 50 mL Falcon tubes, degassed via sonication, and allowed to sit overnight prior to use. Each sample was kept refrigerated prior to analytical studies.

Citrus Extracts: Fresh leaves of the Washington Navel Orange variant were randomly collected from plants located outside the Highlander Union Building (HUB) at the University of California, Riverside on July 8, 2026. The leaves were then extracted immediately after collection. Prior to

extraction, the leaves were washed with water to remove dust and debris and then dried with paper towels.

Extraction procedure: the leaves were cut into smaller pieces and transferred to a 500 mL beaker. Dichloromethane (CH_2Cl_2) was added to completely submerge all of the leaves. A magnetic stir bar was then added, and the suspension was stirred at ambient temperature for 24 h. The resulting suspension was subjected to vacuum filtration to remove the leaves. The leftover residue was rinsed with additional CH_2Cl_2 and the combined filtrates were transferred to a 250 mL Erlenmeyer flask. The filtrates were subjected to a series of aqueous washes. The organic phase was washed twice with water, followed by washing with saturated $NaHCO_3$ solution and then saturated NaCl (brine) solution. The organic layer was separated and dried over anhydrous Na_2SO_4. The drying agent was removed by gravity filtration and the filtrate was concentrated under reduced pressure to remove the remaining CH_2Cl_2.

The resulting crude extract was redissolved in 1,2-dimethoxyethane (DME; Oakwood Chemical, catalog no. 098861) to give a stock solution with a final concentration of 158 mg/mL. The resulting stock solution was further diluted to 0.6 mg/mL for all of the titration experiments performed in citrus leaves extract.

^{1}H, ^{19}F and ^{13}C NMR spectra were recorded on Bruker Avance NEO 400 MHz and 600 MHz NMR spectrometers. The spectrometers were automatically tuned and matched to the correct operating frequencies. Proton (^{1}H) and carbon (^{13}C) chemical shifts are reported in parts per million (δ) with respect to tetramethylsilane (TMS, $\delta = 0$) and referenced internally with respect to the protio solvent impurity. Fluorine (^{19}F) chemical shifts are reported in parts per million (δ) and referenced internally to the standard hexafluorobenzene shift ($\delta = -164.9$). Deuterated NMR solvents were obtained from Cambridge Isotope Laboratories, Inc., Andover, MA, and used without purification. High-resolution accurate mass spectral data were obtained from the Analytical Chemistry Instrumentation Facility at the University of California, Riverside as recorded on an Agilent 6545 Q-TOF LC/MS instrument. LC-MS measurements for the PFOS removal experiments were performed on an Agilent Technologies LC/MSD iQ system.

1.2 Experimental Methods

Fluorescence measurements

Fluorescence measurements were performed on an Agilent BioTek Synergy H1 MultiMode Reader, both operated in the Fluorescence Endpoint mode. Excitation/emission wavelength settings were as follows: 480/600 nm (**DSMI**), 500/600 nm (**PSMI**), 528/630 nm (**QR**), 462/528 nm (**Methyl Orange**), 520/620 nm (**Methyl Red)**, and 600/630 nm (**Indigo Carmine**). The Agilent BioTek Synergy Reader gain was optimized for each dye: 126 (**DSMI**), 119 (**PSMI**), 140 (**QR**), 120 (**Methyl Orange**), 150 (**Methyl Red**) 150 (**Indigo Carmine**). Fluorescence data were analyzed using Gen5 software (v3.16). A blank sample was also prepared to calculate the normalized fluorescence response (F/F_0). The F/F_0 ratio was calculated by dividing the measured fluorescence intensity (F) by the fluorescence of the blank (F_0).

XTI:Dye Titrations

The dye stock solutions were prepared in dimethyl sulfoxide (DMSO) purchased from Fisher Chemicals at a concentration of 20 mM and later diluted with water for use in experiments. The **FPTI** stock solutions (320 µM) were also prepared in water and later diluted with water for use in experiments. The fluorescence titration curves were generated by using 0.5 µM **dye** (**DSMI**, **PSMI**, **QR**, **Methyl Orange**, **Methyl Red**, and **Indigo Carmine**) with increasing **FPTI** concentrations from 0-20 µM in 20 mM Tris-HCl buffer pH 7.4.

XTI:Dye + PFAS Titration

The PFAS titration experiments were conducted using 8 receptor:dye complexes, 0.5 µM **dye** (**DSMI**, **PSMI**, or **QR**) and 4 µM **XTI** (**PTI**, **FPTI**, and **CTI**) in 20 mM Tris-HCl buffer at pH 7.4. A blank sample was also prepared to calculate the normalized fluorescence response (F/F_0). The sample mixtures were incubated for 20 min and then 100 µL of each mixture was transferred to a 96-well plate. Each PFAS target was pipetted into separate wells in triplicate.

Fluorescence Response Plots of XTI/Dye and PFAS

The data for fluorescence response plots were obtained using 8 receptor:dye complexes, consisting of 0.5 µM **dye** (**DSMI**, **PSMI**, and **QR**) and 4 µM **XTI** (**PTI**, **FPTI**, and **CTI**) in 20 mM Tris-HCl buffer at pH 7.4. “Real-World” water samples and beverages were added at 10% volume to

solution mixture of 0.5 μM dye, 4 μM **XTI**, 20 mM Tris-HCl buffer, and 20 μM PFOS. A blank sample was also prepared to calculate the normalized fluorescence response (F/F_0). The sample mixtures were incubated for 20 min and then 100 μL of each mixture was transferred to a 96-well plate. For each of the six PFAS targets, five replicate measurements were pipetted into separate wells.

Liposome Preparation

A stock solution of POPC lipid was dissolved in 1:9 $CH_3OH/CHCl_3$ (5 mg/mL) and retained in a freezer at -80°C. Liposomes were prepared via transfer of the POPC stock to a glass vial and dried under brief vacuum. The lipid film was maintained in a desiccator overnight. The dried lipid bilayer was then resuspended in water to achieve a final concentration of 1 mg/mL. The resulting cloudy mixture was vortexed to ensure full solvation of the lipid and sonicated for 20 minutes. The liposome mixture was then extruded via 100 nm membranes to produce unilamellar liposomes.

Limit of Detection for PFOS

To measure the limit of detection (LOD) of PFOS the linear portion of the fluorescence titrations was used. The LOD was calculated via the following formula: LOD = (3 × SD)/Slope. The standard deviation (SD) is representative of the blank measurements and slope is derived from the linear region of the fluorescence titration curve. The LOD measurements were calculated for PFOS in three different buffered conditions: Tris-HCl pH 7.4, Tris-HCl pH 7.4 with 10% human saliva, and Tris-HCl pH 7.4 with 10% artificial urine. The LOD measurements were calculated for PFOS in two “Real-World” water samples: Tris-HCl pH 7.4 with 10% Balboa Beach Water and Tris-HCl pH 7.4 with 10% Gfuel. And the LOD measurements were extended to citrus leaves extract spiked with 100 μM PFOS. The spiked samples were diluted to obtain PFOS concentrations ranging from 0 to 20 μM with corresponding citrus leaves extract concentrations ranging from 0 to 0.12 mg/mL.

LC-MS

LC-MS analyses were conducted to evaluate the removal capabilities of **PTI**, **FPTI,** and **CTI** following filtration. This was done by monitoring the decrease in the PFOS peak area. A decrease in the peak area indicates that the macrocycles are capable of forming a complex with PFOS and generating aggregates (see main text), thereby lowering their concentration in the solution. 1.0 mL

deionized water samples containing PFOS (10.0 μM) and **PTI**, **FPTI**, or **CTI** (0.00–7.00 μM) were allowed to stand on the bench for 0.5 h and then passed through a 0.22 μm MCE filter. The filtrates were analyzed using an Agilent Technologies LC/MSD iQ system. Data are presented as mean ± SD (n = 3). PFOS concentrations were determined using a calibration curve constructed from PFOS standards at 0.00, 0.01, 0.05, 0.10, 0.50, 1.00, 5.00, and 10.0 μM. The estimated limit of detection (LOD) was 0.02 μM, based on the low-concentration (0.00-1.00 μM) calibration curve.

Dynamic Light Scattering (DLS) Measurements

DLS measurements for size and zeta potential analysis were performed using Zetasizer Nano ZS. For each measurement, 1 mL of sample was prepared at the appropriate concentration in 20 mM Tris-HCl buffer at pH 7.4. Samples were transferred to clean disposable cuvettes before measurement. In the case of all samples, the measurements were carried out in triplicate, with each triplicate consisting of 10 runs and a 10 s delay. Data were analyzed using Zetasizer software version 7.02, and all graphs were manually generated using Graphpad Prism 10.6.

Optical Microscopy

The aggregate images were acquired using two different microscopes: a Keyence BZ-X Series all-in-one fluorescence microscope and BIM-800FLW inverted epi-fluorescent biological microscope. The sample was prepared by mixing 4 mM **PTI** and 8 mM PFOS in water. For imaging with the Keyence microscope, 20 μL of the prepared sample was transferred to a clean disposable 18-chambered cover glass purchased from Cellvis. For analysis with the BIM-800FLW microscope, 100 μL of the sample was transferred to a clean microscope slide and allowed to dry for 1 hour. The sample was visualized under brightfield illumination using 20x, 50x, and 100x objectives on the BIM-800FLW microscope and a 40x objective on the Keyence microscope. Images were captured using BZ-X1000 Viewer and analyzed using BZ-X1000 Analyzer for the Keyence microscope and Qcam 2.0.8 software for the BIM-800FLW microscope.

Single Crystal X-ray Diffraction

FPTI•Br$_4$ (10 mg, 0.01 mmol)) was dissolved into 20 mL CH_3CN. The solution was sonicated and heated to dissolve the receptor, after which 500 μL of water was added, resulting in a clear solution. The vial was placed into a jar and prepared for vapor diffusion with ether. After six days crystals

suitable for X-ray analysis were acquired. A colorless crystal (plate, approximate dimensions 0.77 × 0.11 × 0.06 mm^3) was placed onto the tip of a MiTeGen pin and mounted on a Bruker Venture D8 diffractometer equipped with a PhotonIII detector at 180.00 K. The data collection was carried out using Mo Kα radiation (l = 0.71073 Å, ImS micro-source) with a frame time of 3 seconds and a detector distance of 40 mm. A collection strategy was calculated and complete data to a resolution of 0.75 Å were collected. The frames were integrated with the Bruker SAINT[7] software package using a narrow-frame algorithm to 0.80 Å resolution. Data were corrected for absorption effects using the multi-scan method (SADABS).[8] Please refer to Table S-1 for additional crystal and refinement information. The data deposited as CCDC number 2551513 also contain additional details.

Structure Solution and Refinement

The space group *P-1* was determined based on intensity statistics and systematic absences. The structure was solved using the SHELX suite of programs[9,10] and refined using full-matrix least-squares on F^2 within the OLEX2 suite.[11] An intrinsic phasing solution was calculated, which provided most non-hydrogen atoms from the E-map. Full-matrix least squares / difference Fourier cycles were performed, which located the remaining non-hydrogen atoms. All non-hydrogen atoms were refined with anisotropic displacement parameters. The hydrogen atoms were placed in ideal positions and refined as riding atoms with relative isotropic displacement parameters. The final full matrix least squares refinement converged to $R1$ = 0.0397 and $wR2$ = 0.1052 (F^2, all data). The goodness-of-fit was 1.025. Based on the final model, the calculated density was 1.775 g/cm^3 and $F(000)$, 516 e-. Two C-level alerts were generated by the IUCr Checkcif validation report. One highlights a difference in U(eq) for C17 compared to the average Ueq) of the atoms bonded to it. C17 is part of an acetonitrile molecule and close to the terminal nitrogen, which has a large ADP, and causes the alert. The second alert warns about missing reflections (1 -4 1, -1 3 3). These two reflections were collected but were rejected during the scaling by SADABS.

Table S-1. Crystal data and structure refinement for [$C_{34}H_{30}N_{10}F_8$][Br_4], CCDC 2551513

Formula	$C_{34}H_{30}N_{10}F_8Br_4$
Formula weight	1050.32
Crystal color, shape, size, mm^3	Colorless plate 0.77 × 0.11 × 0.06
Temperature	180.0 K
Wavelength, Å	0.71073
Crystal system	Triclinic
space group	*P-1*
a, Å	8.1309(14)
b, Å	11.2970(16)
c, Å	12.4276(18)
α, °	113.142(6)°
β, °	108.688(5)°
γ, °	90.490(5)°
Volume	982.4(3) $Å^3$
Z,	1
Calculated density, mg/m^3	1.775
Absorption coefficient, mm^{-1}	4.175
F(000)	516
**Data Collection**	
Diffractometer	Bruker D8 Venture
Theta range for data collection, deg	1.904 to 26.371
Limiting indices	$-10<=h<=10$, $-14<=k<=14$, $-15<=l<=15$
Reflections collected / unique	25458, 4021 [R_{int} = 0.0968]
Observed reflections	3393
Completeness to θ = 25.242°	99.9%
**Solution and Refinement**	
Absorption correction	Semi-empirical from equivalents
Max. and Min. Transmission	0.74557 and 0.4978
Solution	Intrinsic methods
Refinement method	Full-matrix least-squares on F^2
Weighting scheme	$w = [\sigma^2 F_o^2 + AP^2 + BP]^{-1}$, with $P = (F_o^2 + 2F_c^2)/3$, $A = 0.045$, $B = 1.35$
Data / restraints / parameters	4021 / 9/ 254
Goodness-of-fit on F^2	1.025
Final *R* indices [$I > 2\sigma(I)$]	$R1$ =0.0397, $wR2$ = 0.0989
R indices (all data)	$R1$ =0.0495, $wR2$ = 0.1052
Largest diff. Peak and hole, $eÅ^3$	1.306, -0.764

1.3 Chemical Synthesis and Characterization

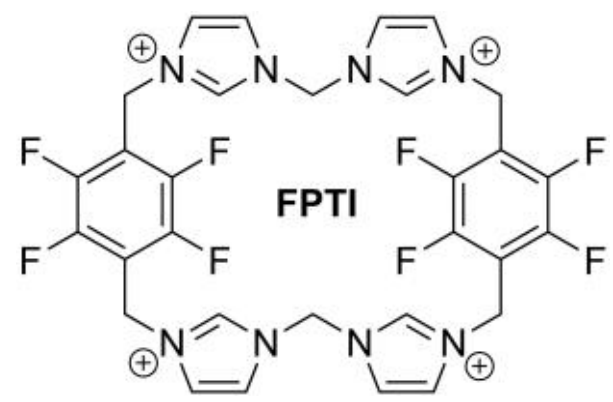

Synthesis of FPTI

A one-necked round-bottomed flask was equipped with a stir bar and di(1H-imidazol-1-yl)methane (100 mg, 0.67 mmol) and 100 mL of acetonitrile were then added. 1,4-Bis(bromomethyl)-2,3,5,6-tetrafluorobenzene (248 mg, 0.74 mmol) was dissolved in 50 mL of acetonitrile and added dropwise to this flask over a 4-hour period via syringe pump. Once the addition was complete, the reaction mixture was heated at reflux for 48 h. After heating, the reaction mixture was allowed to cool at ambient conditions and briefly sonicated. The reaction mixture was concentrated under reduced pressure to remove the volatiles and resuspended in 100 mL of ether and then filtered via vacuum filtration. The resulting solid precipitate was rinsed briefly with 100 mL of acetonitrile and dried under high vacuum to give a white powder (264 mg, 82%). ^{1}H NMR (600 MHz, CD_3CN) δ 10.21 (s, 4H), 8.31 (s, 4H), 7.71 (s, 4H), 6.82 (s, 4H), 5.53 (s, 8H) (600 MHz, DMSO-d_6) δ 9.84 (s, 4H), 8.17 (s, 4H), 7.71 (s, 4H), 6.63 (s, 4H), 5.71 (s, 8H) (600 MHz, D_2O) δ 8.93 (s)*, 7.81 (s, 4H), 7.64 (s, 4H), 6.67 (s, 4H), 5.66 (s, 8H) *Denotes proton that undergoes H-D exchange. ^{13}C {^{1}H} NMR (101 MHz, D_2O δ 146.4, 143.9, 137.4, 137.1, 124.0, 122.8, 113.1, 59.0, 58.7, 41.0 ^{19}F NMR (D_2O, 564 MHz, referenced to C_6F_6) -141.06 (HRMS) *m/z* $[M]^{+}$*calcd* for $C_{30}H_{24}F_8N_8Br_3$ 886.9521 found 886.9520.

2. Characterization Data

2.1 NMR Spectral Characterization of New Molecules

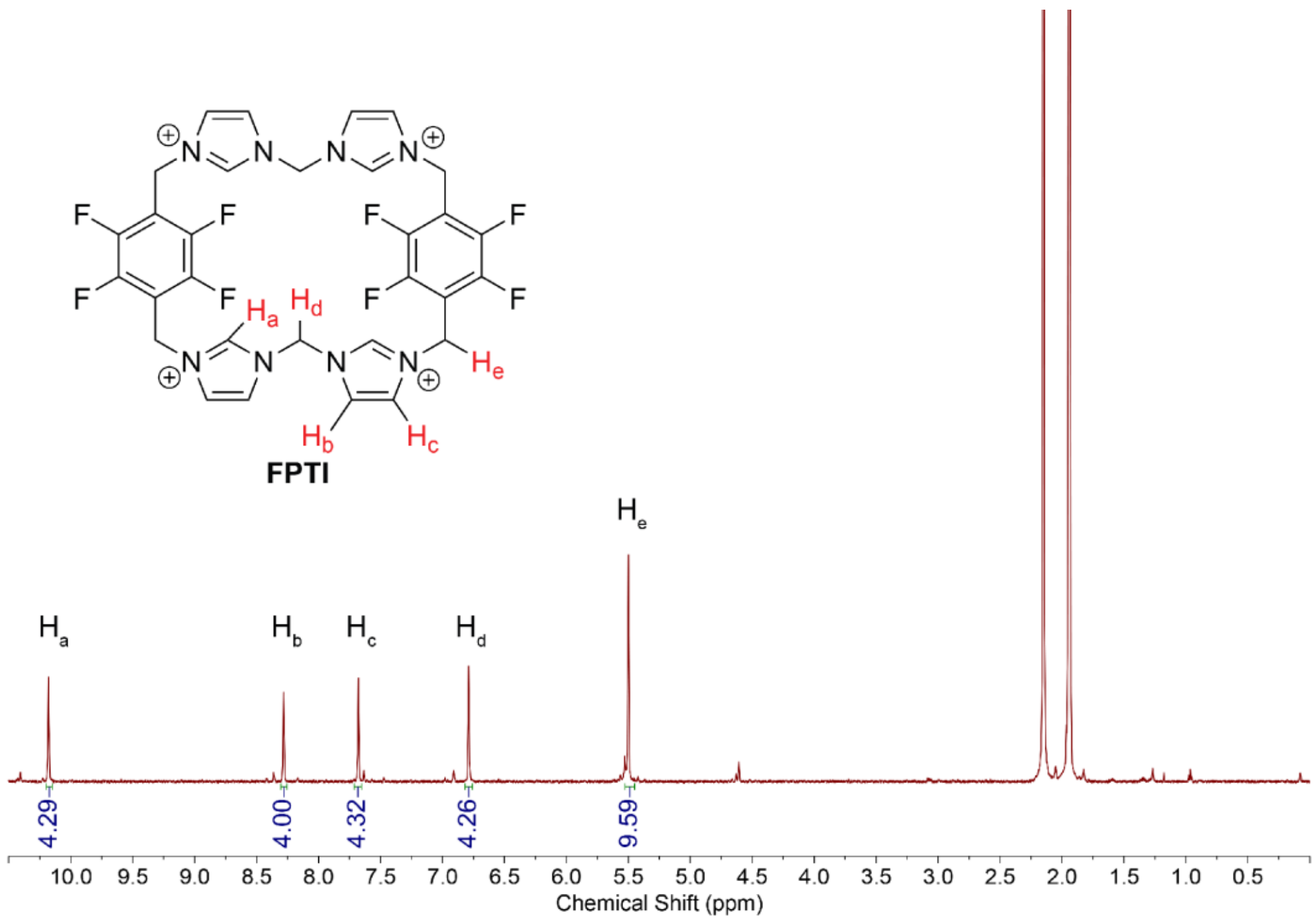

Figure S-1. ^{1}H NMR spectrum of **FPTI** (CD_3CN, 600 MHz, 298K).

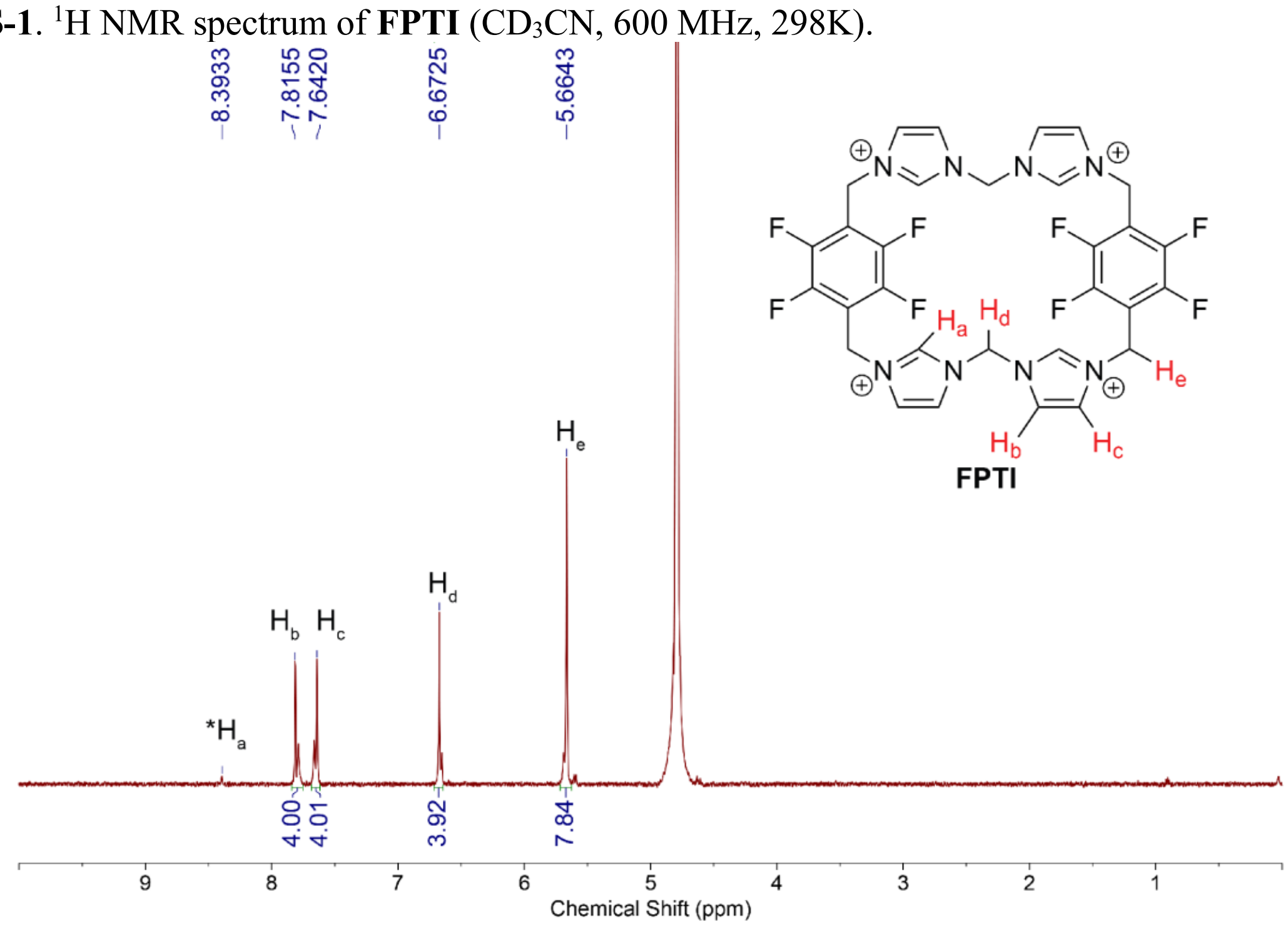

Figure S-2. ^{1}H NMR spectrum of **FPTI** (D_2O, 600 MHz, 298K). * Denotes proton that undergoes H-D exchange.

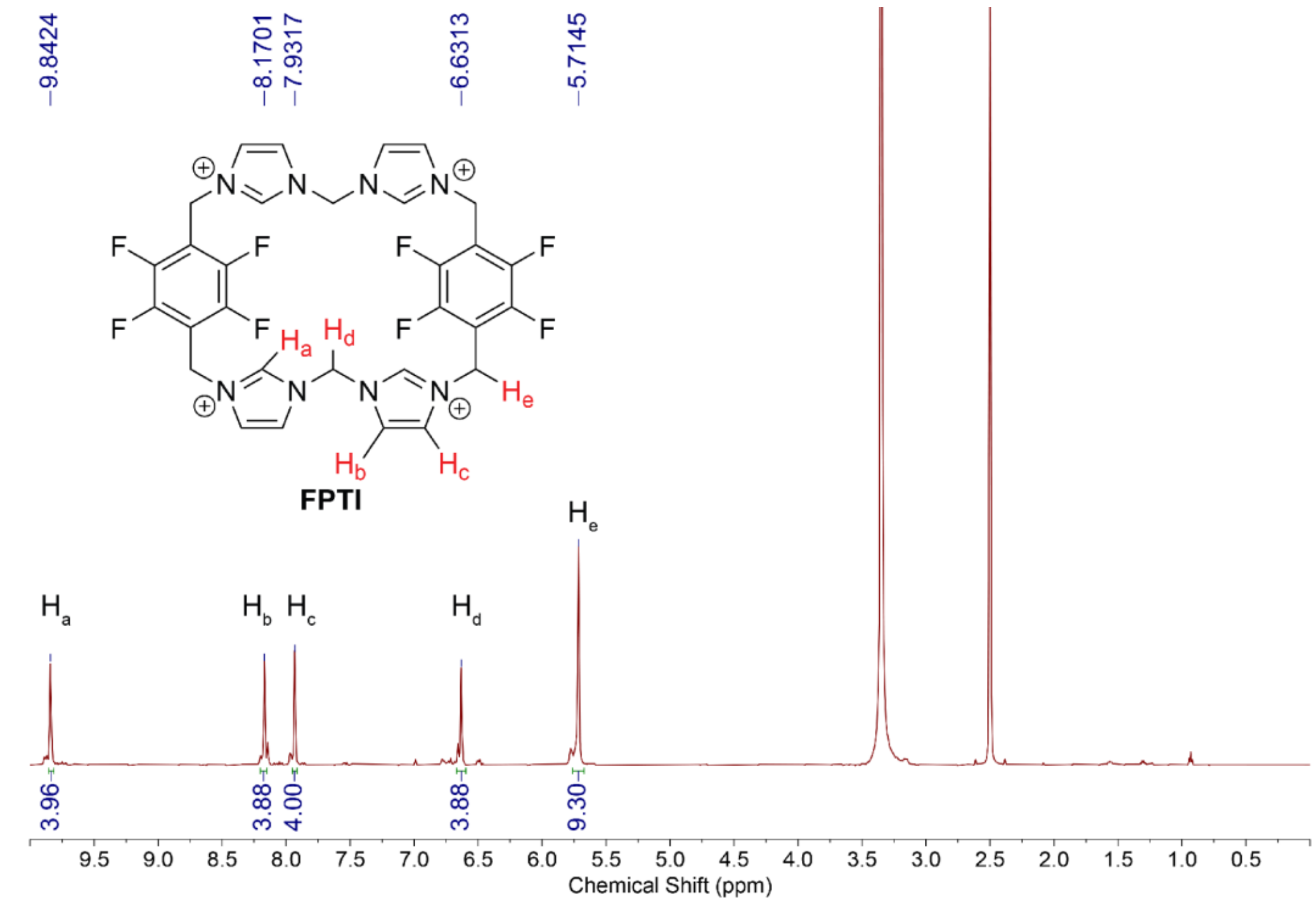

Figure S-3. ^{1}H NMR spectrum of **FPTI** (DMSO-d_6, 600 MHz, 298K).

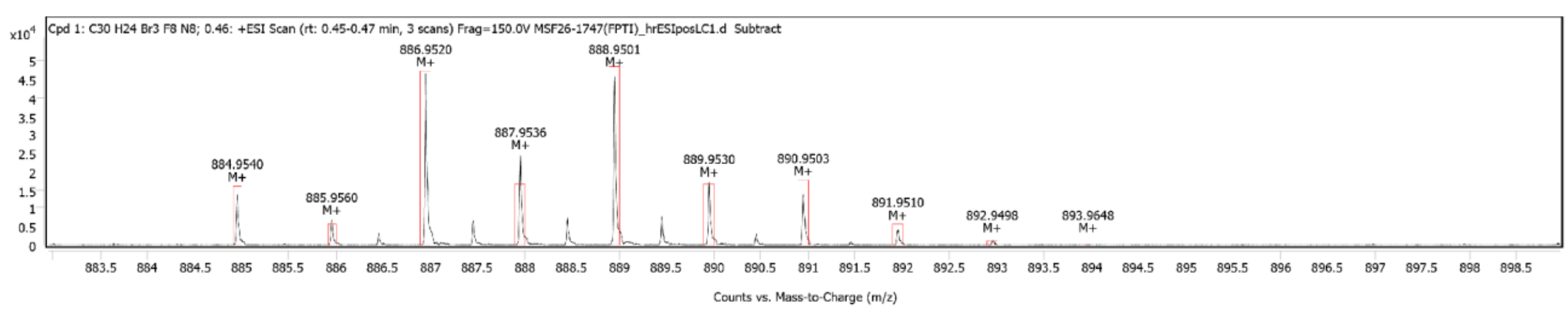

Figure S-4. High-resolution mass spectrometry spectrum of **FPTI**. Red boxes represent theoretical *m/z* of **FPTI•Br**$^{+}$ cation and black peak represent experimental *m/z* peaks.

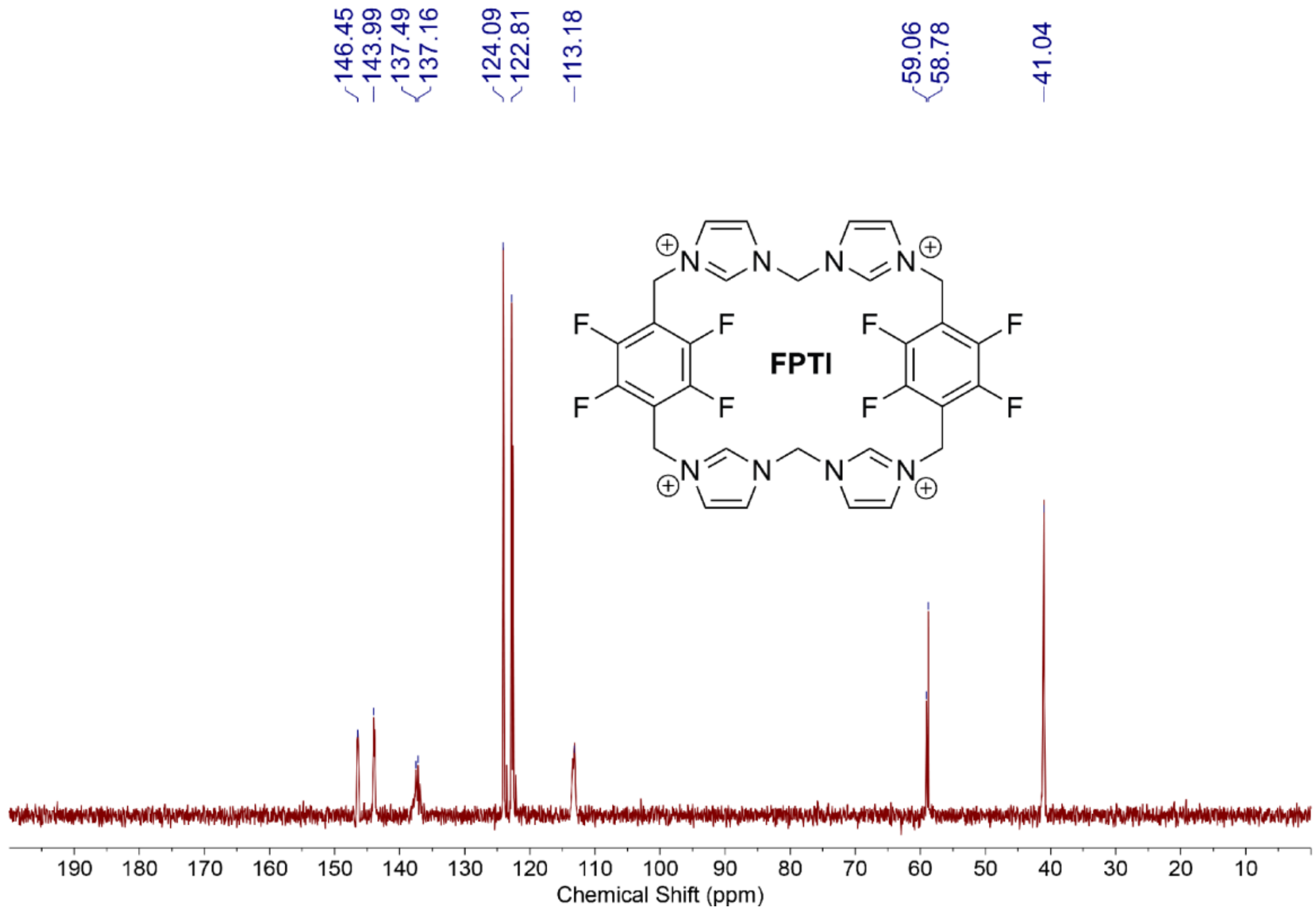

Figure S-5. $^{13}C\{^{1}H\}$ NMR spectrum of **FPTI** (D_2O, 101 MHz, 298K).

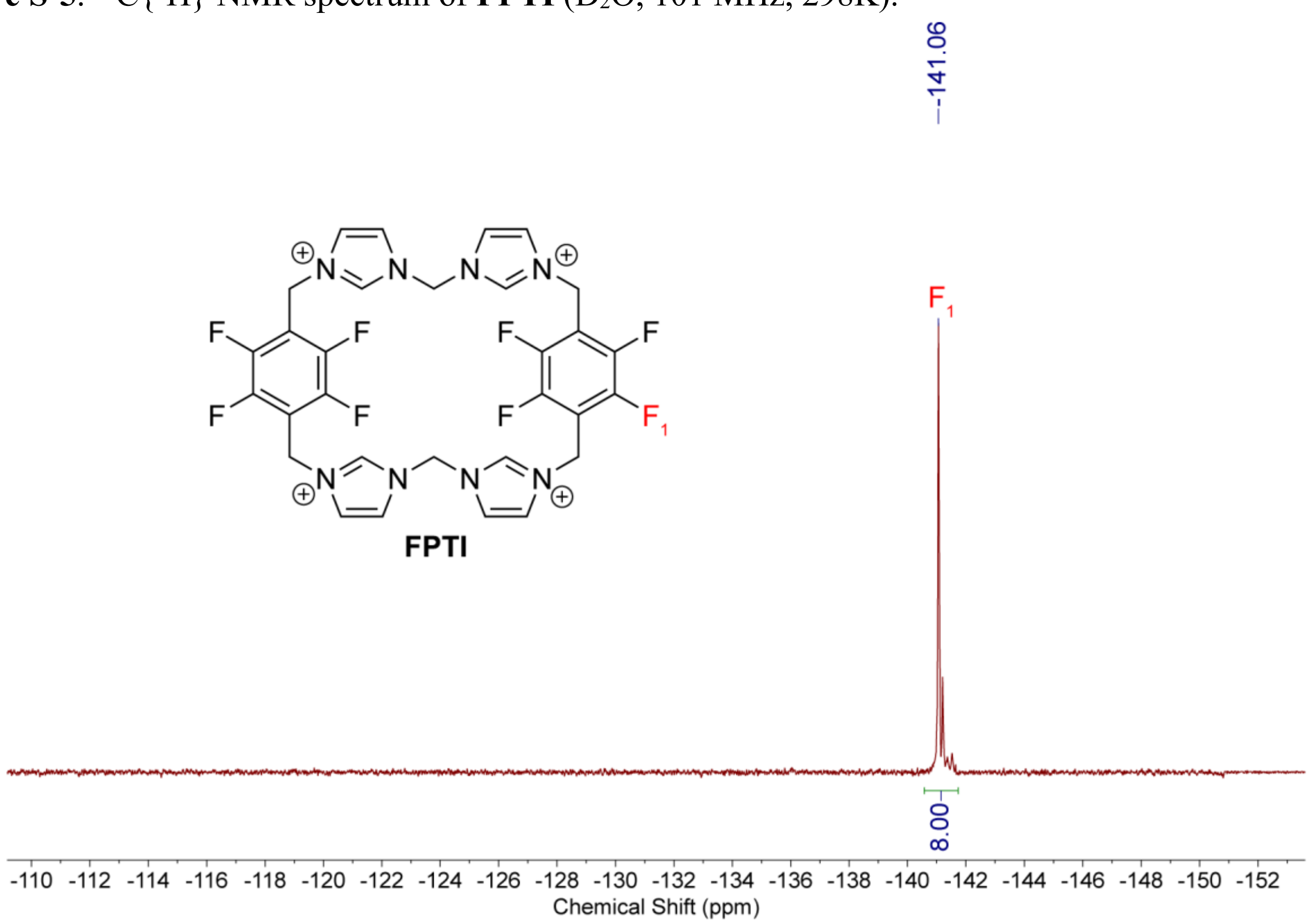

Figure S-6. ^{19}F NMR spectrum of **FPTI** (D_2O, 564 MHz, 298K).

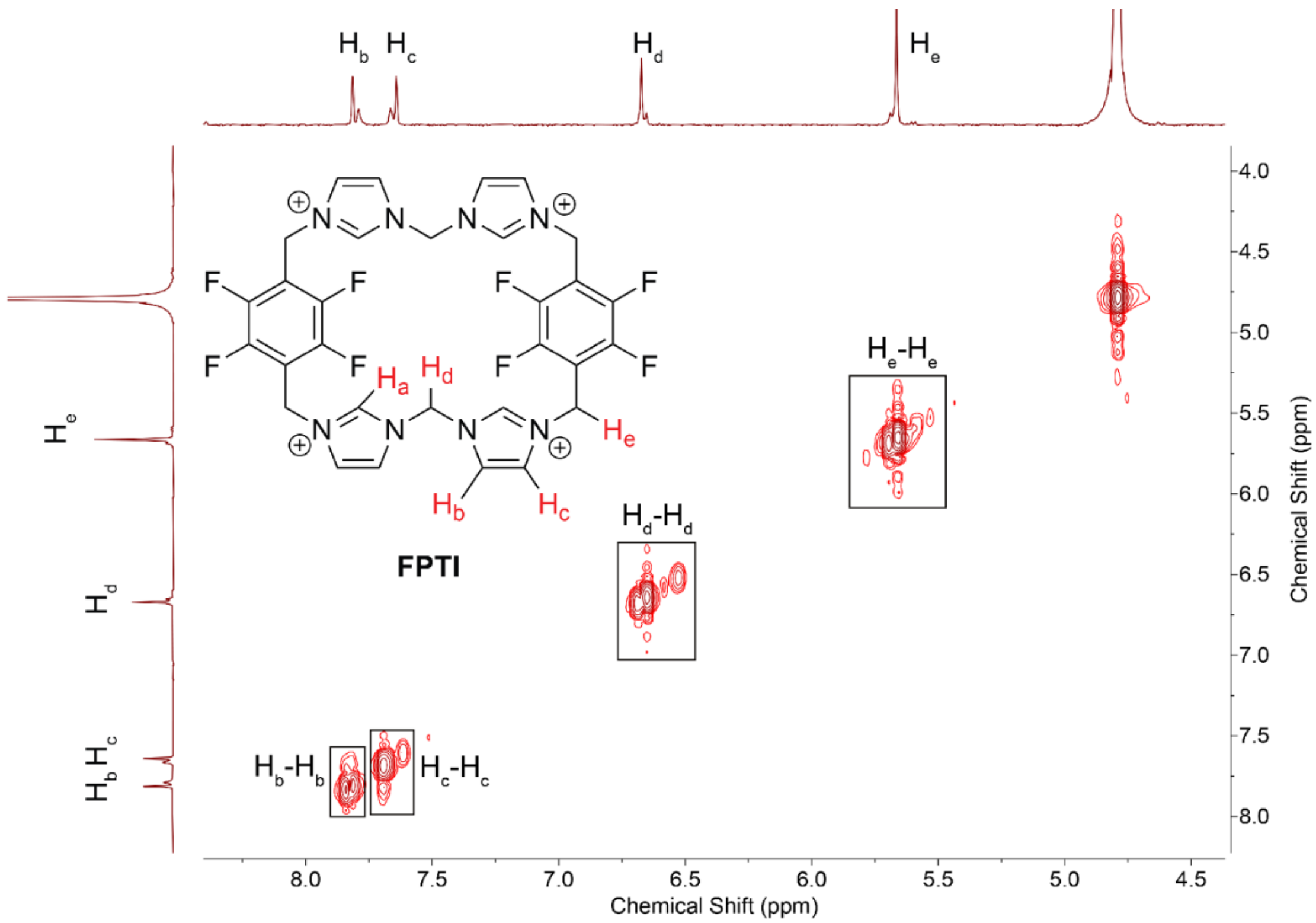

Figure S-7. COSY NMR spectrum of **FPTI** (D_2O, 600 MHz, 298K).

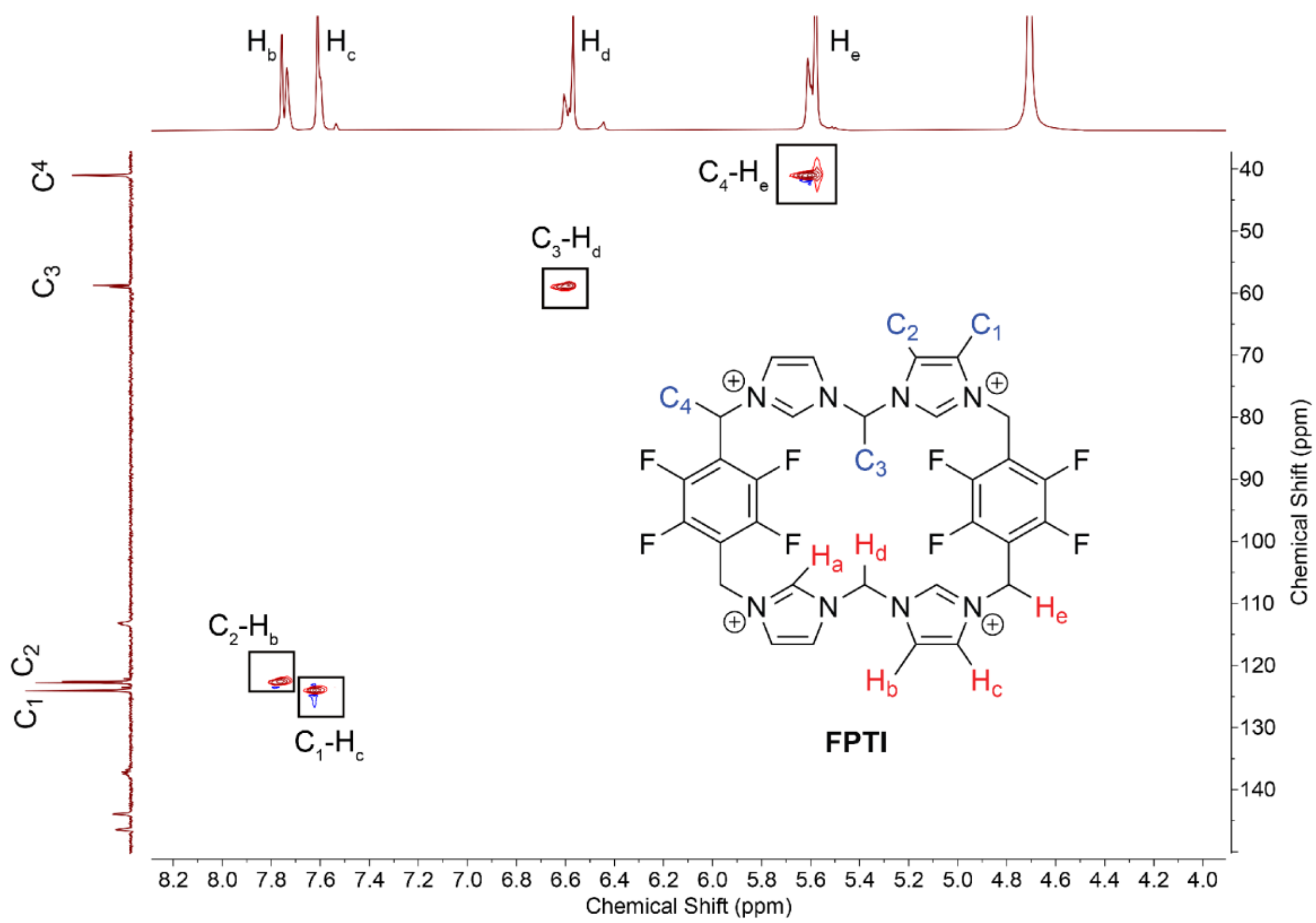

Figure S-8. HSQC NMR spectrum of **FPTI** (D_2O, 600 MHz, 298K).

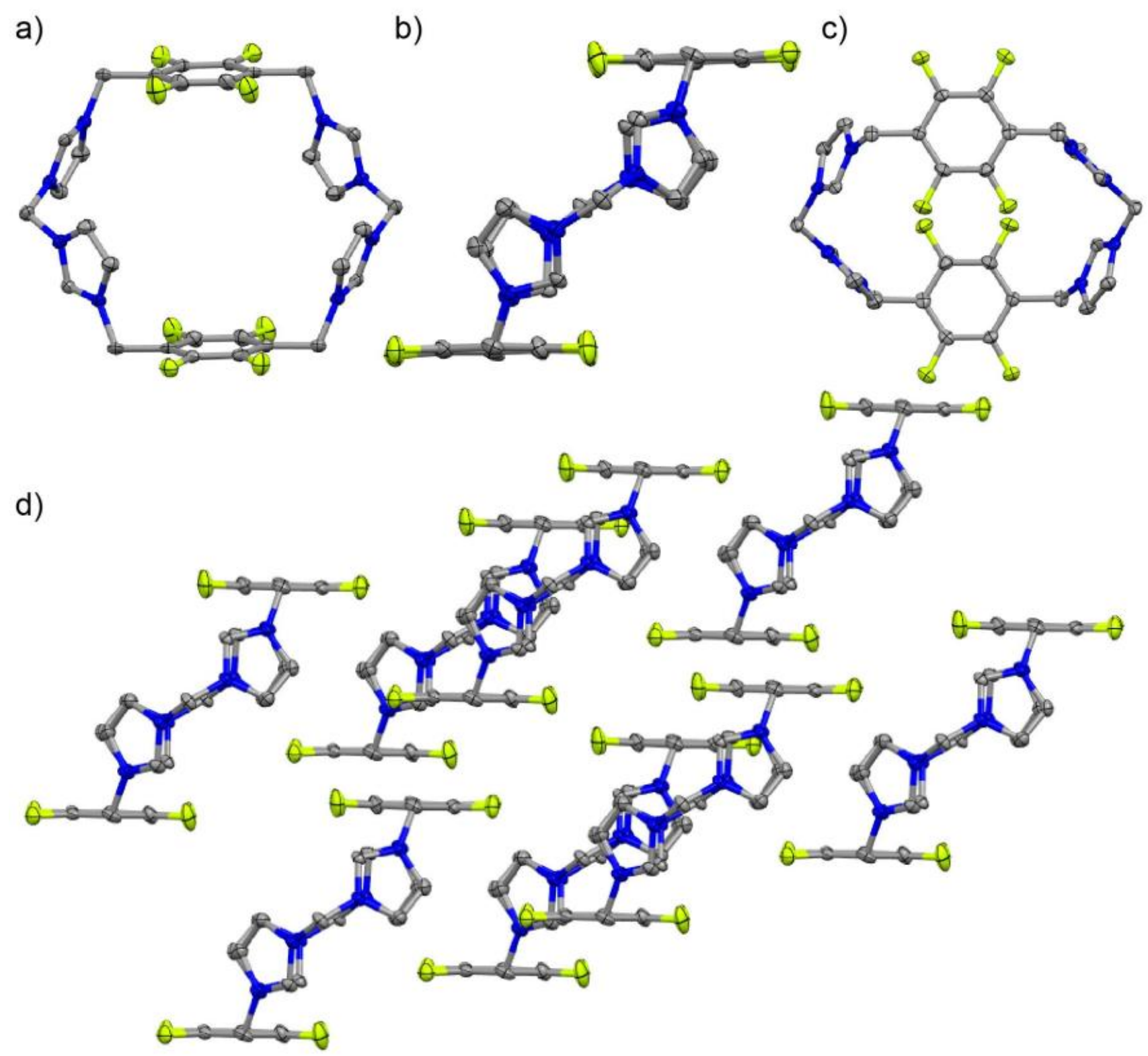

Figure S-9. scXRD structure of $[C_{34}H_{30}N_{10}F_8][Br_4]$, CCDC 2551513. a) Front view; b) side view; c) top view; d) packing unit. Grey, blue, and yellow-green ellipsoids represent carbon, nitrogen, and fluorine. Ellipsoids are shown at 50% probability levels. Protons, solvent (CH_3CN), and counter anions (Br^-) are removed for clarity.

3. Fluorescence Spectral Titrations

3.1 FPTI-Dye Titrations

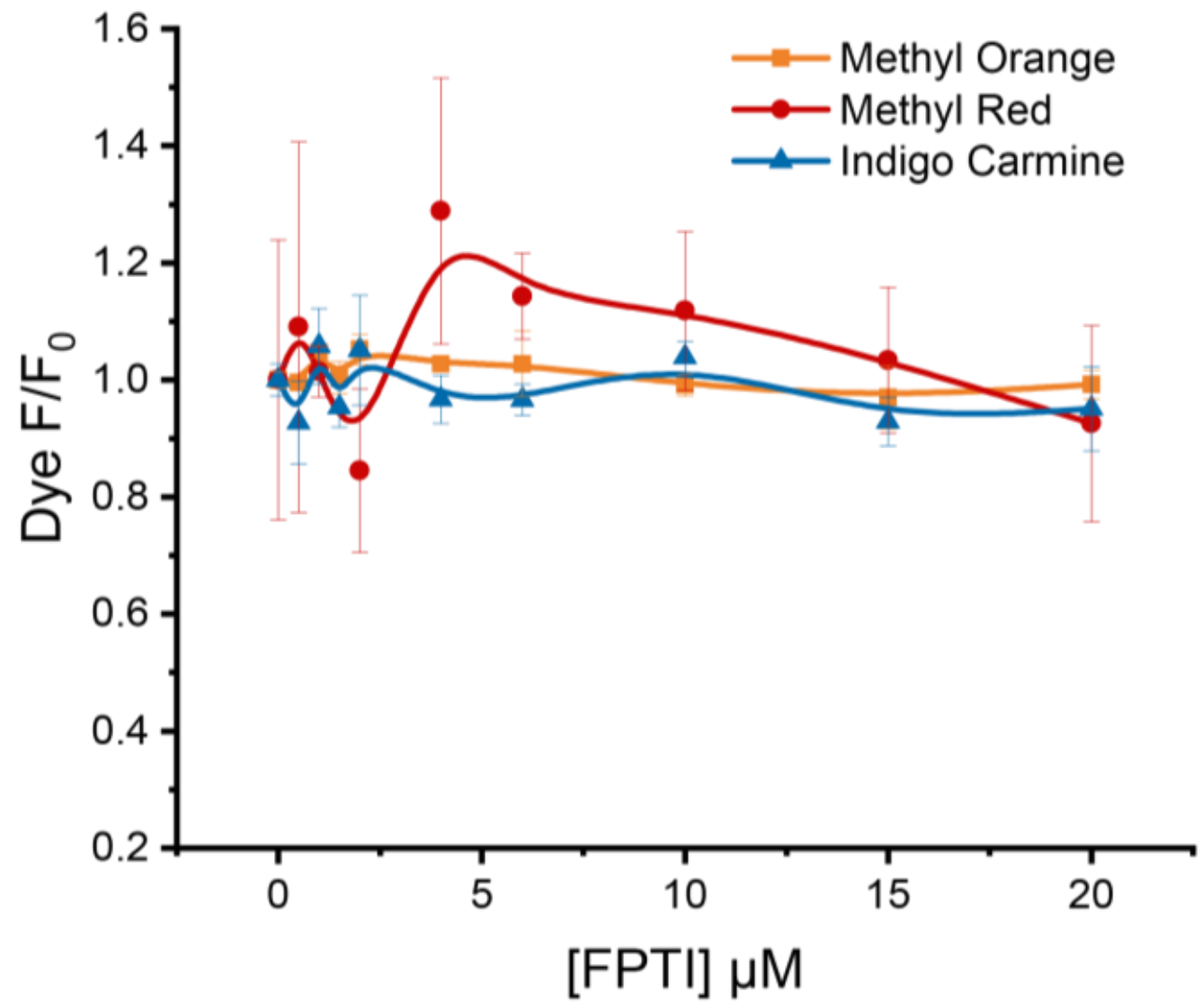

Figure S-10. Normalized fluorescence emission (F/F_0) titration curves of 0.5 µM dye (Methyl Orange, Methyl Red, and Indigo Carmine) and increasing concentration of **FPTI** (0-20 µM) in 20 mM Tris-HCl buffer at pH 7.4.

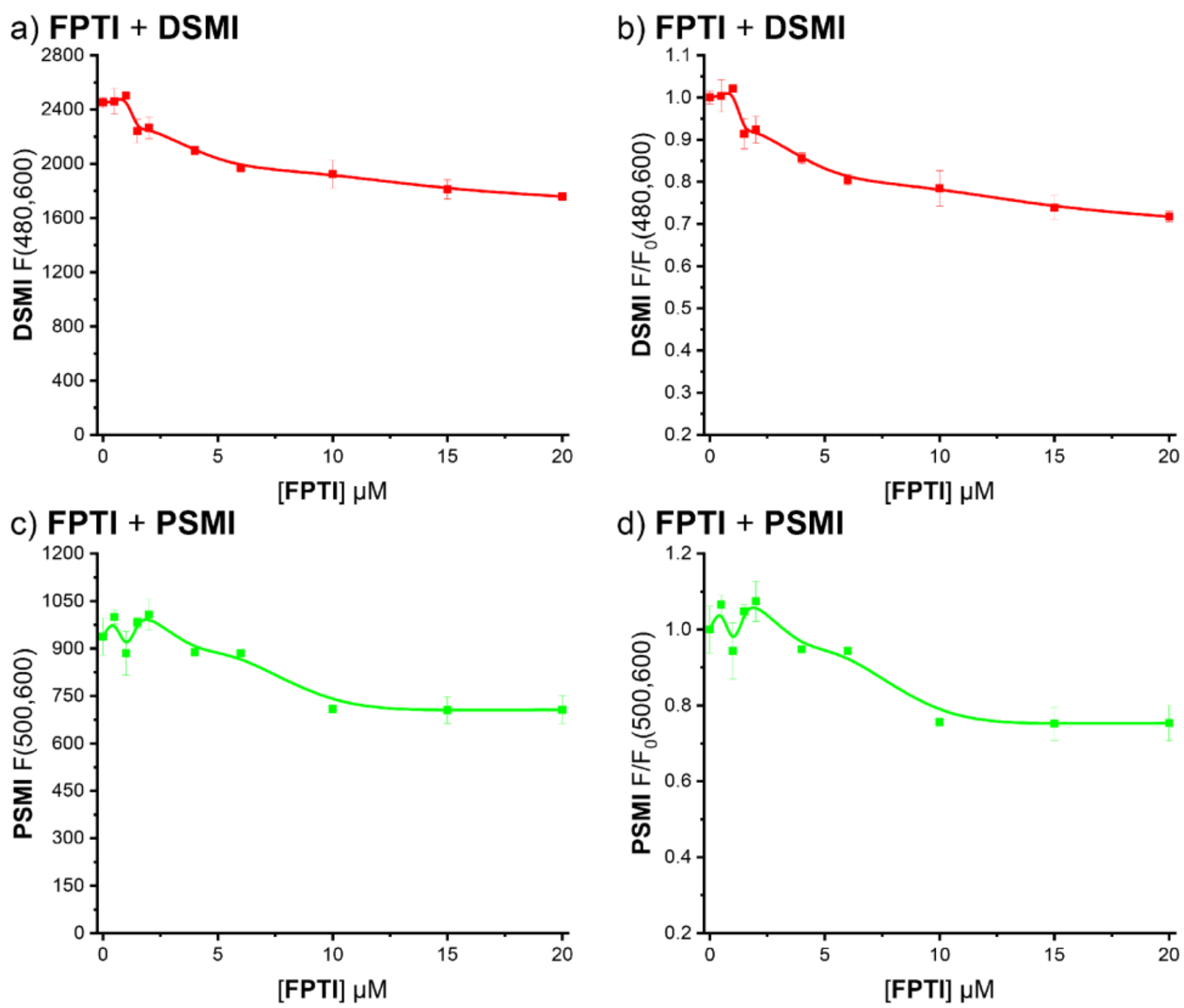

Figure S-11. Fluorescence emission titration curves of 0.5 µM dye and increasing concentration of **FPTI** (0-20 µM) in 20 mM Tris-HCl buffer at pH: 7.4 a) **DSMI** F; b) **DSMI** F/F_0; c) **PSMI** F; d) **PSMI** F/F_0.

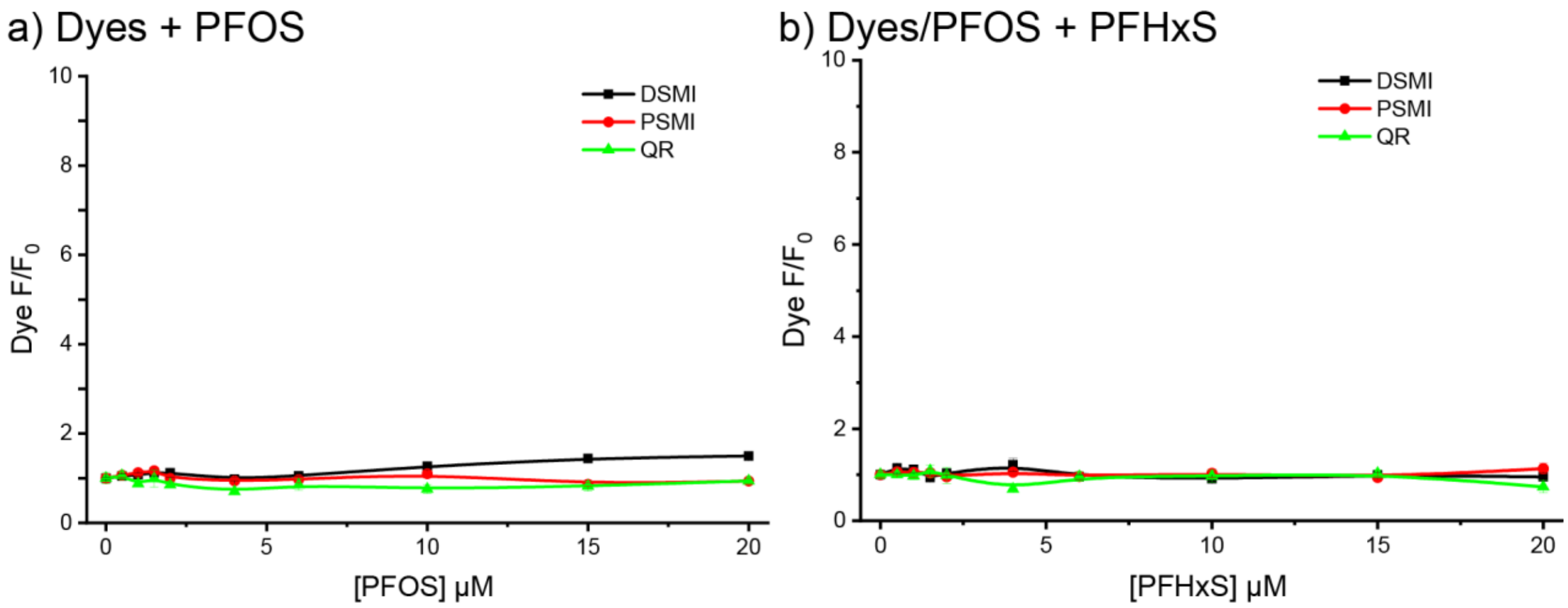

Figure S-12. Fluorescence emission titration curve of 0.5 μM dye (**DSMI**, **PSMI**, **QR**) and increasing concentration of PFAS: a) PFOS (0-20 μM); b) 20 μM PFOS + PFHxS (0-20 μM) in 20 mM Tris-HCl buffer.

3.2 XTI-Dye + PFAS Titrations

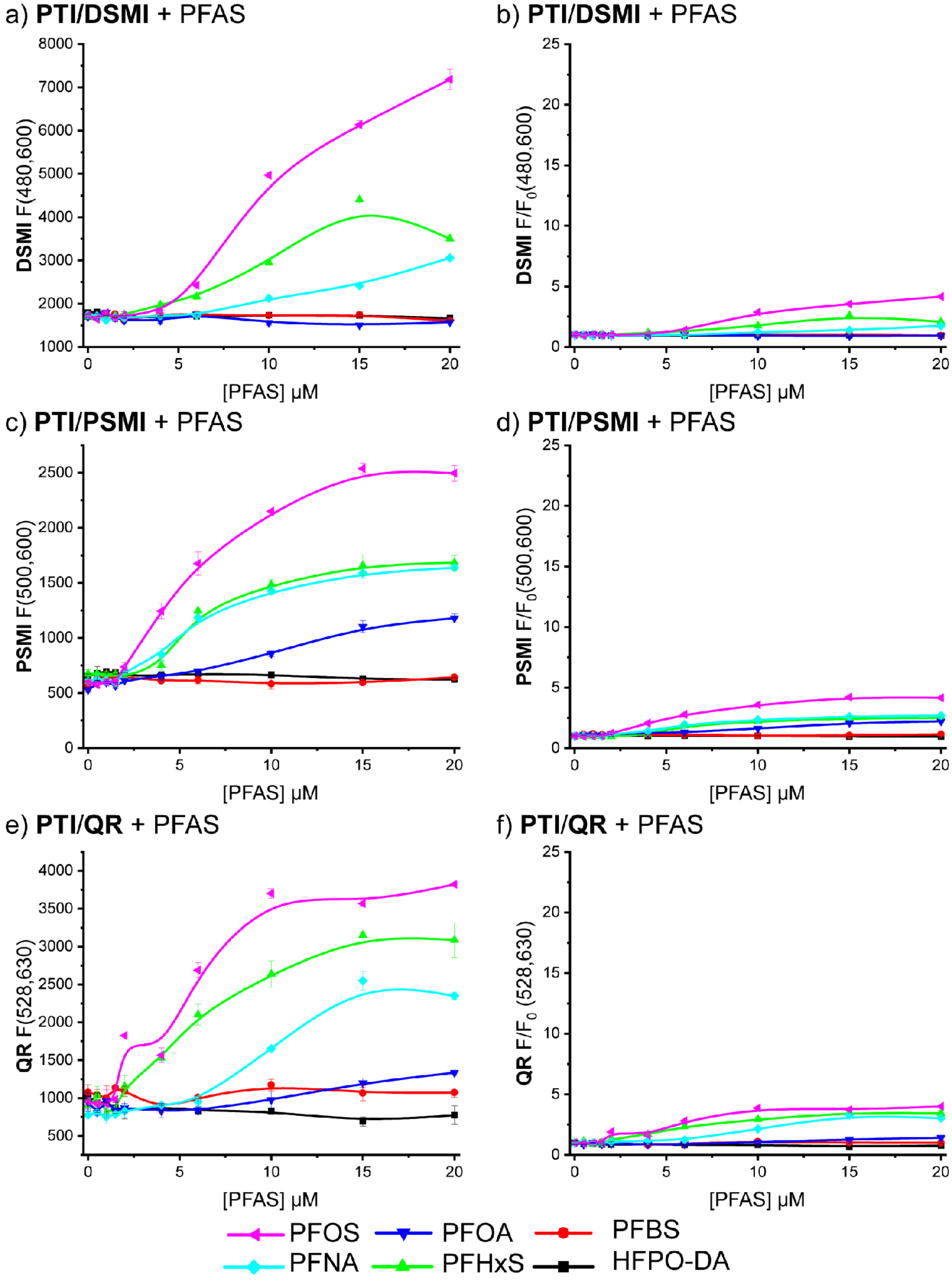

Figure S-13. Fluorescence emission (F) (left) and normalized fluorescence emission (F/F_0) (right) titration curves of 0.5 μM dye and 4.0 μM **PTI** and increasing concentration of PFAS (PFOS, PFOA, PFNA, PFHxS, PFBS, and HFPO-DA) at 0-20 μM in 20 mM Tris-HCl buffer at pH 7.4: a) **PTI•DSMI** F; b) **PTI•DSMI** F/F_0; c) **PTI•PSMI** F; d) **PTI•PSMI** F/F_0; e) **PTI•QR** F; f) **PTI•QR** F/F_0.

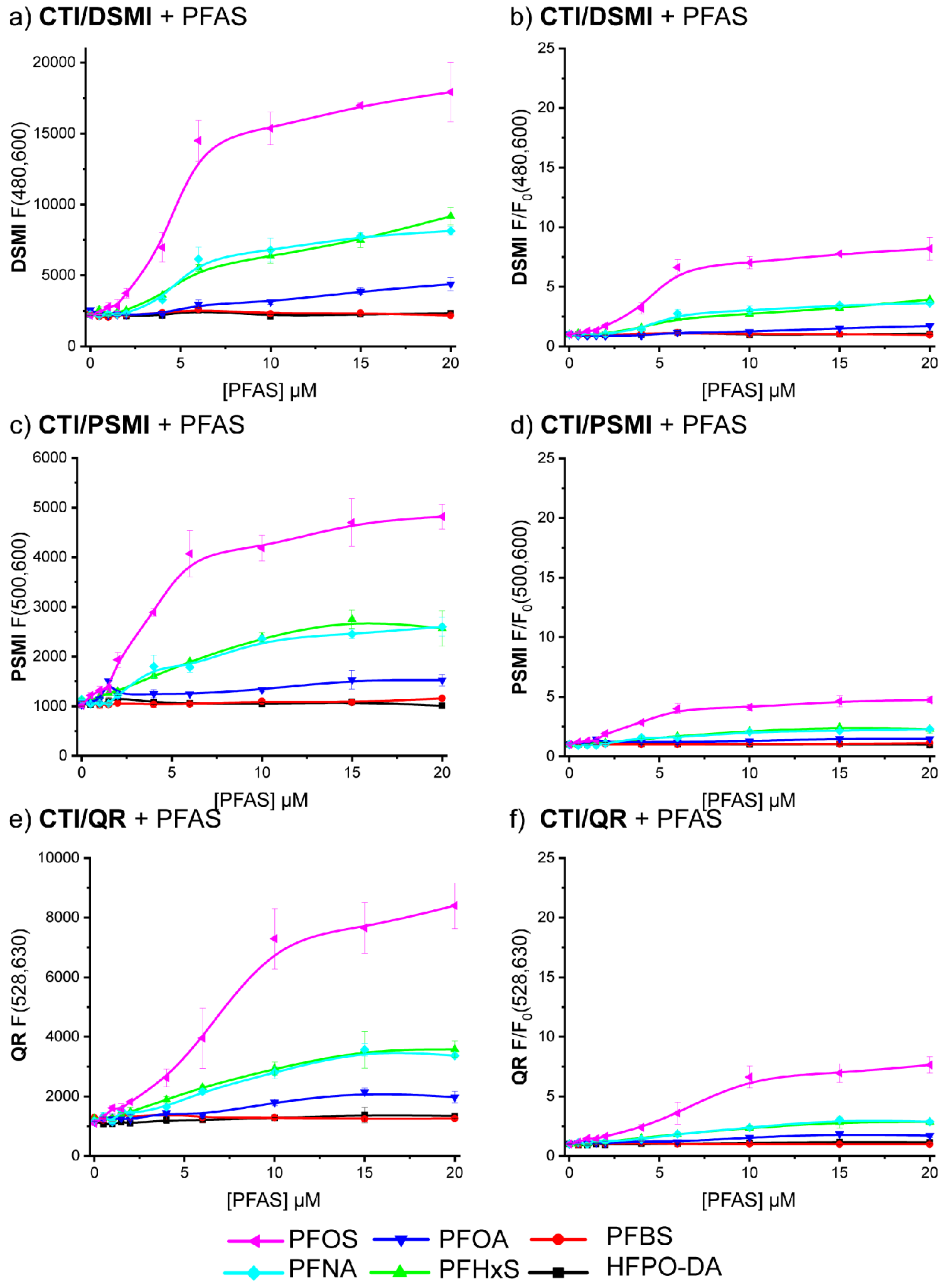

Figure S-14. Fluorescence emission (F) (left) and normalized fluorescence emission (F/F_0) (right) titration curves of 0.5 μM dye and 4.0 μM **CTI** and increasing concentration of PFAS (PFOS, PFOA, PFNA, PFHxS, PFBS, and HFPO-DA) at 0-20 μM in 20 mM Tris-HCl buffer at pH 7.4: a) **CTI•DSMI** F; b) **CTI•DSMI** F/F_0; c) **CTI•PSMI** F; d) **CTI•PSMI** F/F_0; e) **CTI•QR** F; f) **CTI•QR** F/F_0.

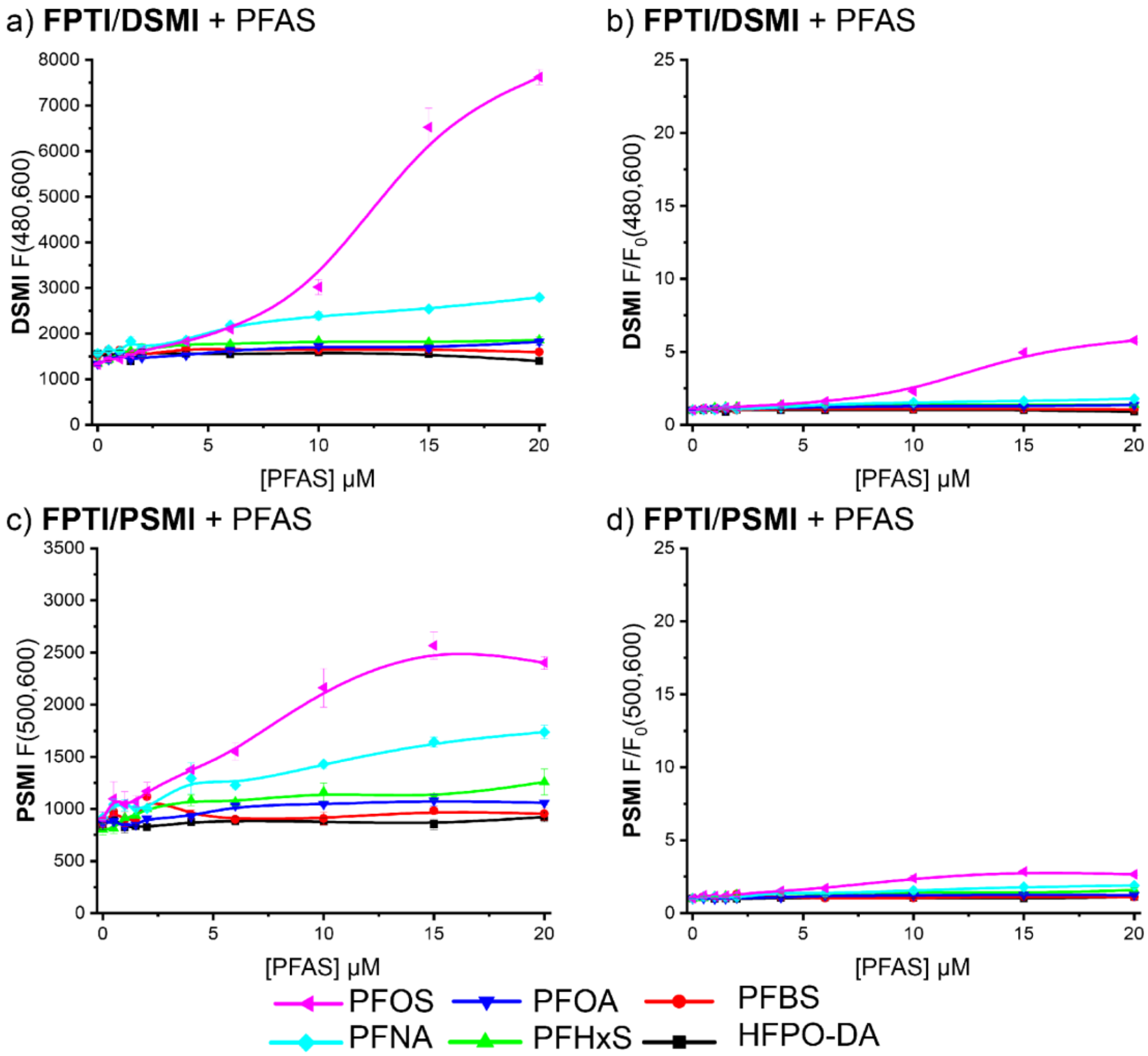

Figure S-15. Fluorescence emission (F) (left) and normalized fluorescence emission (F/F_0) (right) titration curves of 0.5 µM dye and 4.0 µM **FPTI** and increasing concentration of PFAS (PFOS, PFOA, PFNA, PFHxS, PFBS, and HFPO-DA) at 0-20 µM in 20 mM Tris-HCl buffer at pH 7.4: a) **FPTI•DSMI** F; b) **FPTI•DSMI** F/F_0; c) **FPTI•PSMI** F; d) **FPTI•PSMI** F/F_0.

3.3 Dye, XTI-Dye + POPC Titrations

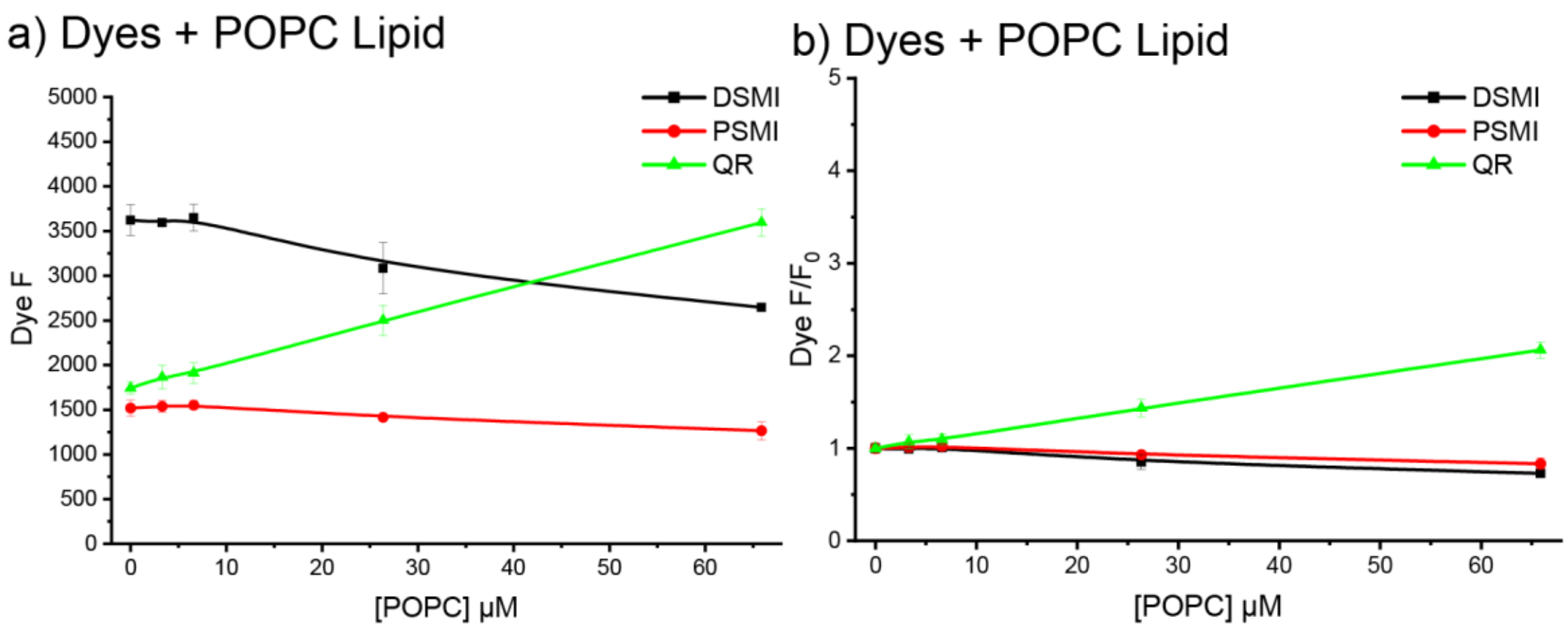

Figure S-16. a) Fluorescence emission (F); b) normalized fluorescence emission (F/F_0) titration curves of 0.5 μM dye (**DSMI**, **DTMI**, **PSMI**) and POPC (0-65 μM) in 20 mM Tris-HCl buffer at pH 7.4.

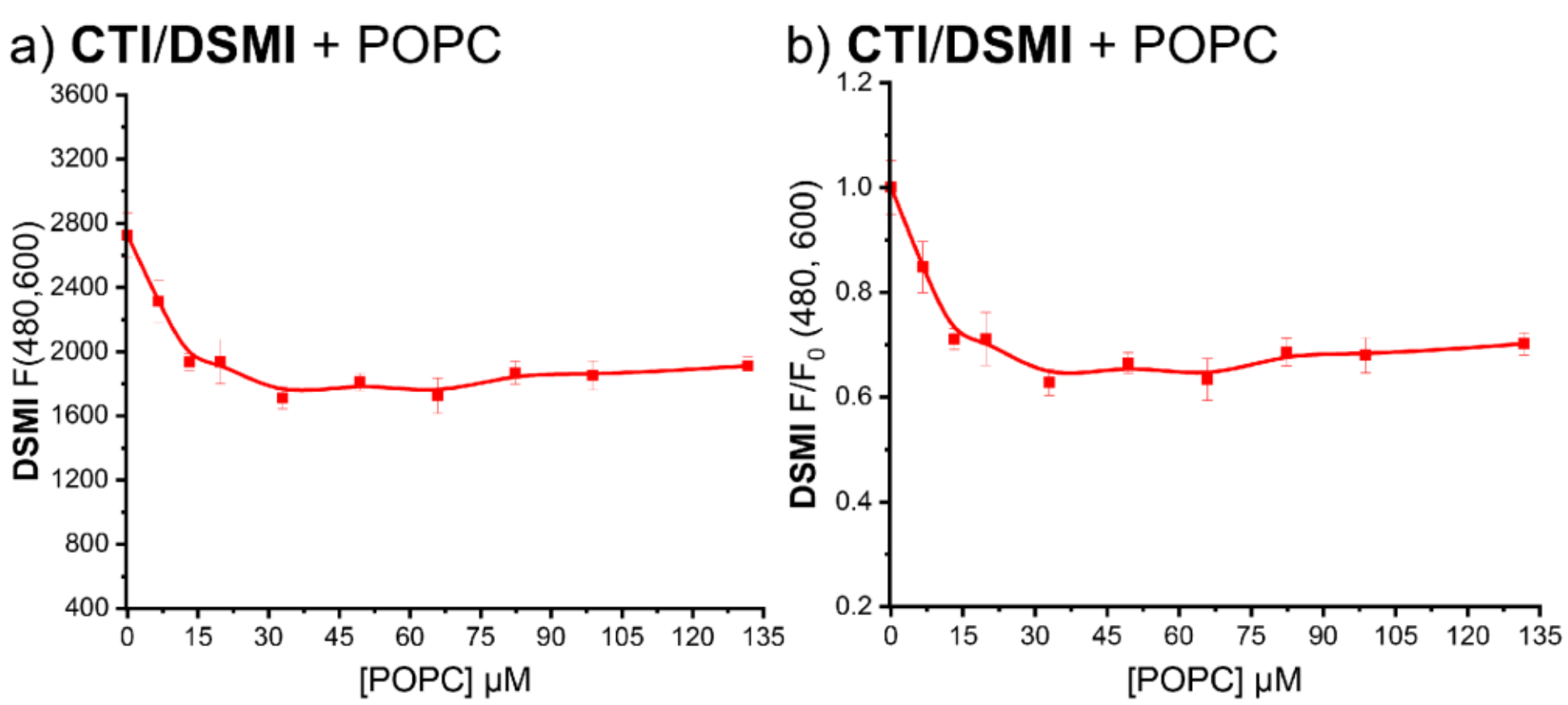

Figure S-17. a) Fluorescence emission (F); b) normalized fluorescence emission (F/F_0) titration curves of 0.5 μM **DSMI** and 0.5 μM **CTI** and POPC (0-132 μM) in 20 mM Tris-HCl buffer at pH 7.4.

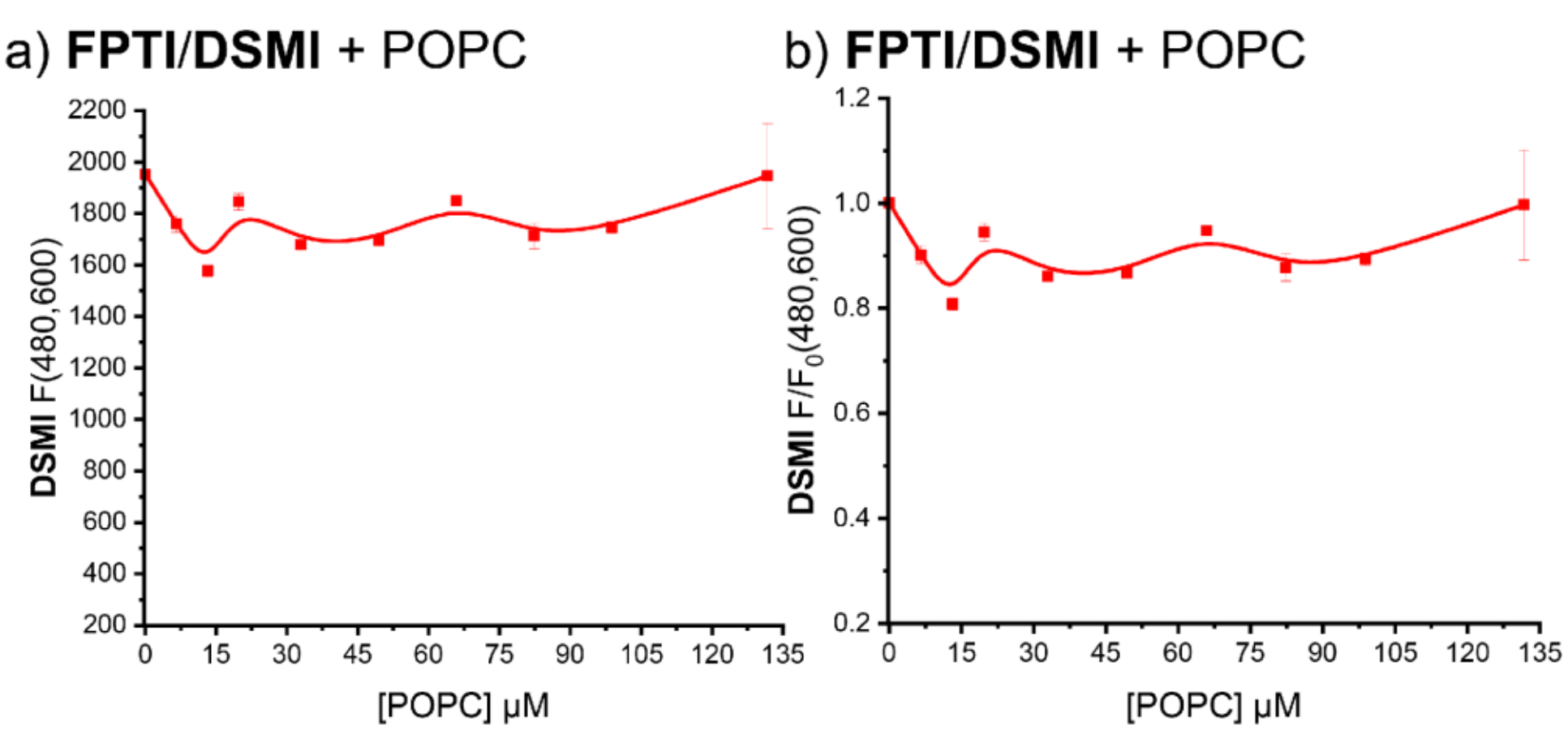

Figure S-18. a) Fluorescence emission (F); b) normalized fluorescence emission (F/F_0) titration curves of 0.5 μM **DSMI** and 0.5 μM **FPTI** and POPC (1 mg/mL) in 20 mM Tris-HCl buffer at pH 7.4.

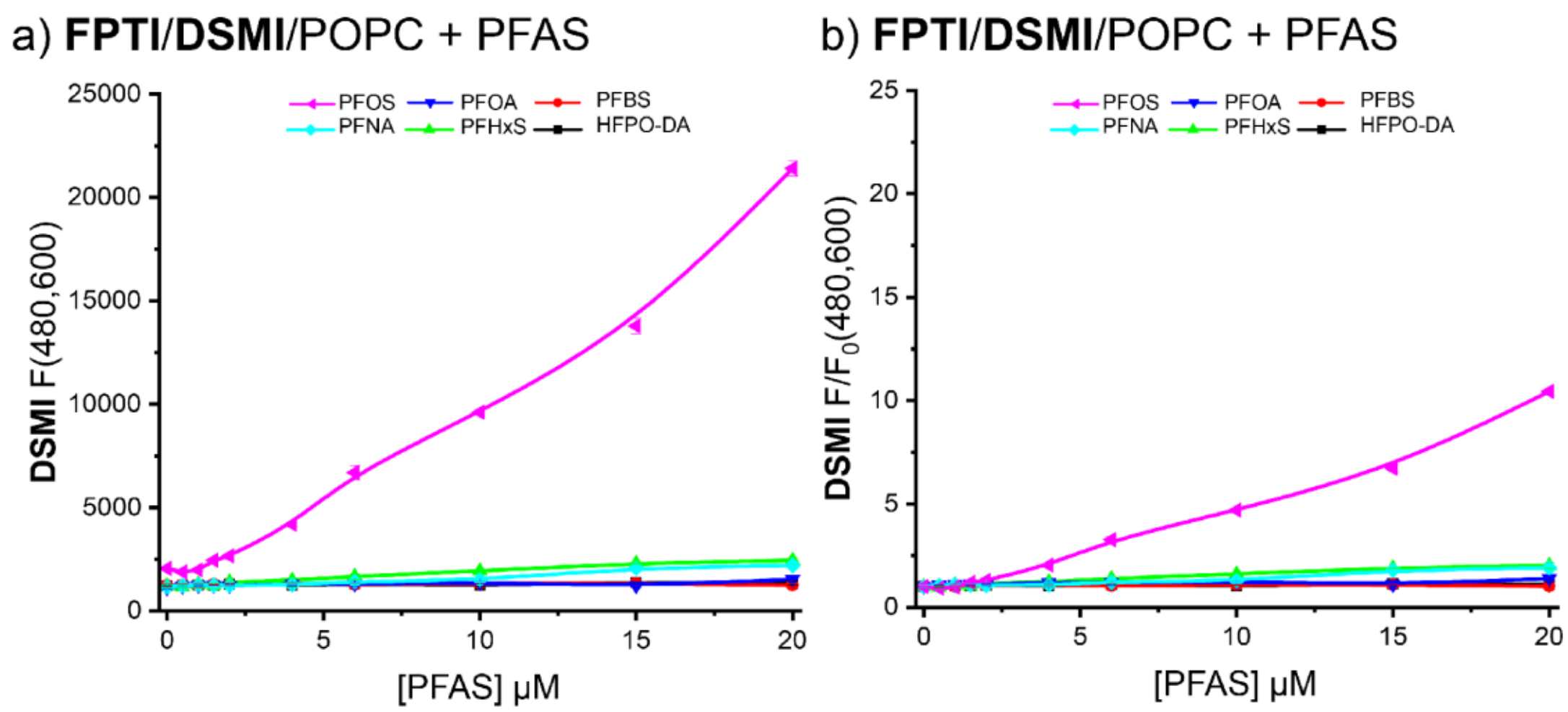

Figure S-19. a) Fluorescence emission (F); b) normalized fluorescence emission titration (F/F_0) curves of 0.5 μM **DSMI**, 4.0 μM **FPTI**, 132 μM POPC, and increasing concentrations of PFAS (PFOS, PFOA, PFNA, PFHxS, PFBS, and HFPO-DA) at 0-20 μM in 20 mM Tris-HCl buffer at pH 7.4.

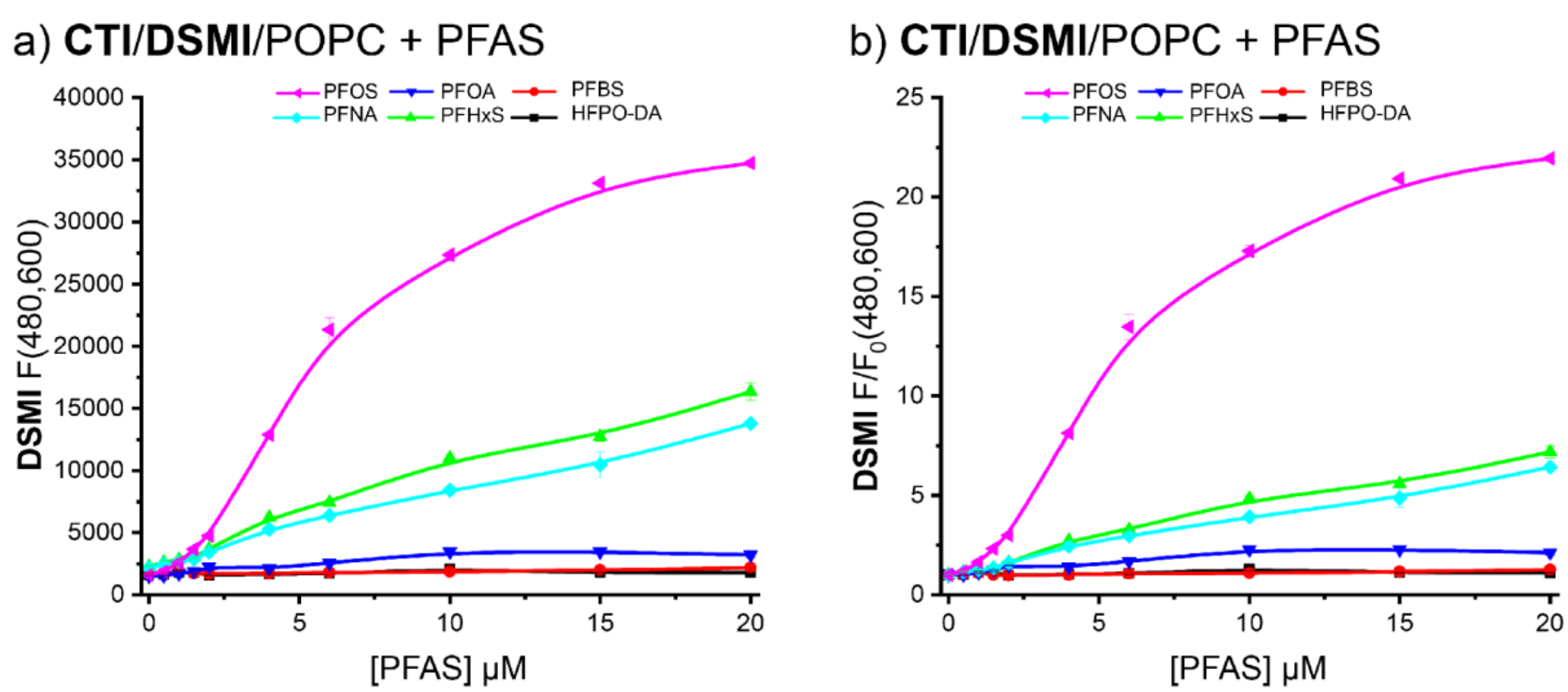

Figure S-20. a) Fluorescence emission (F); b) and normalized fluorescence emission titration (F/F_0) curves of 0.5 μM **DSMI**, 4.0 μM **CTI•Br**, 132 μM POPC, and increasing concentrations of PFAS (PFOS, PFOA, PFNA, PFHxS, PFBS, and HFPO-DA) at 0-20 μM in 20 mM Tris-HCl buffer at pH 7.4.

3.4 XTI-Dye + Biofluid Titrations

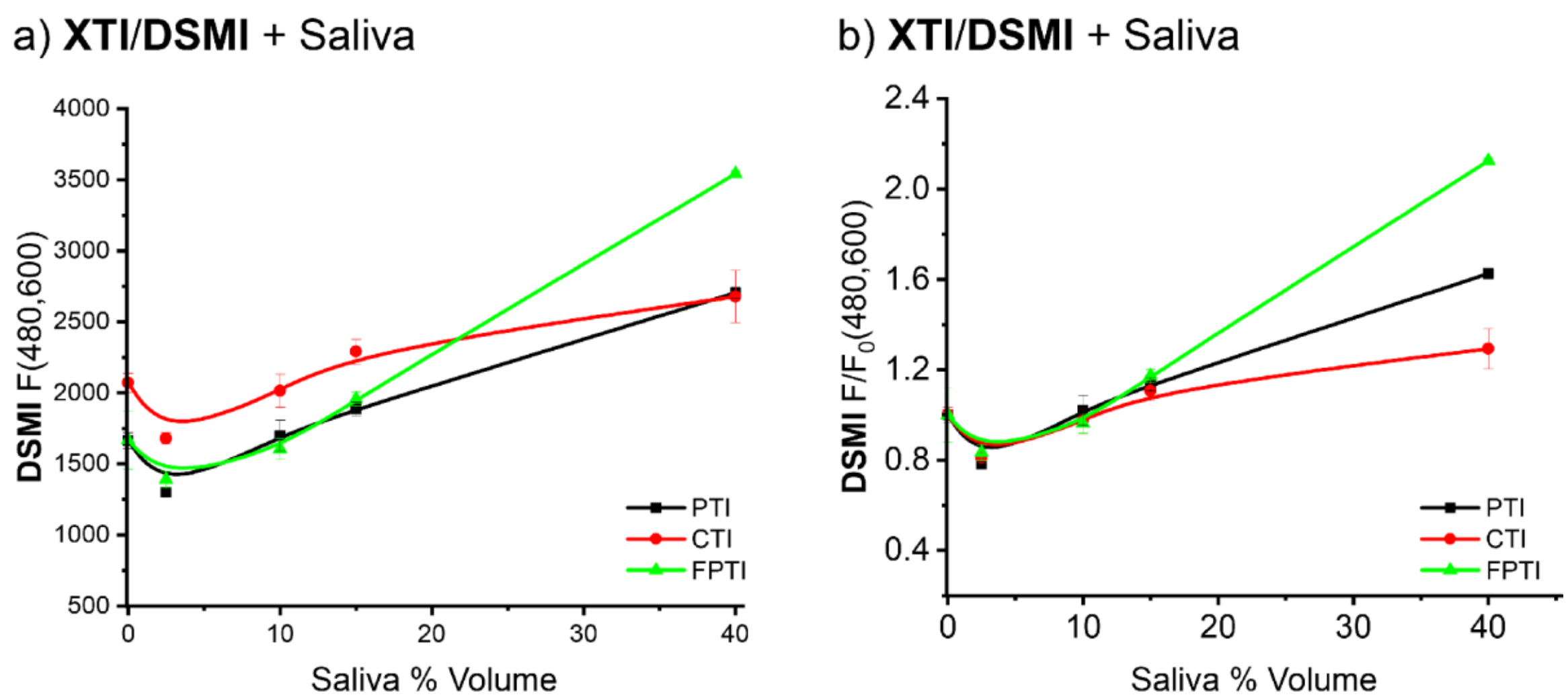

Figure S-21. Fluorescence emission titration plot of 0.5 μM **DSMI** and 4.0 μM **XTI** (**PTI**, **CTI**, **FPTI**) with increasing % volume of human saliva: a) F; b) F/F_0.

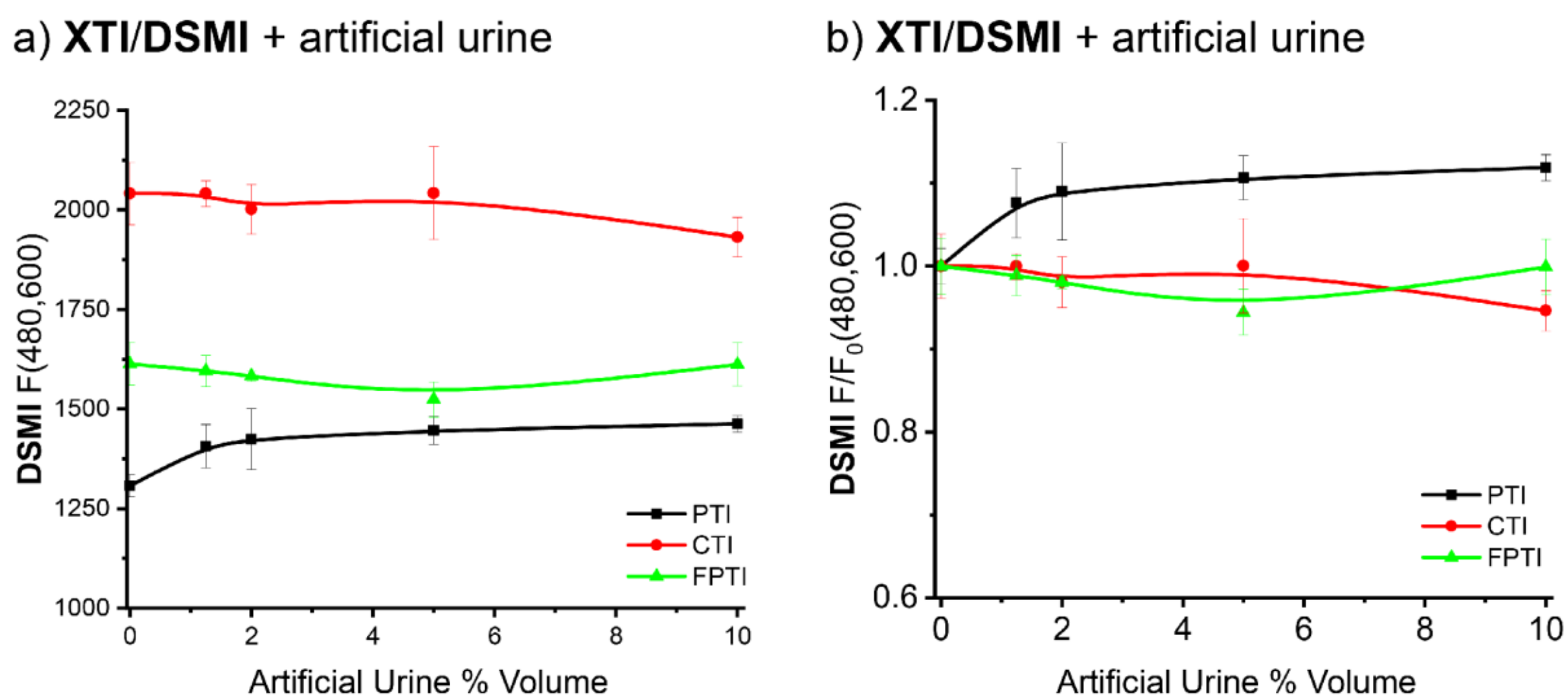

Figure S-22. Fluorescence emission titration plot of 0.5 μM **DSMI** and 4.0 μM **XTI** (**PTI**, **CTI**, **FPTI**) with increasing % volume of artificial urine: a) F; b) F/F_0.

3.5 XTI-Dye + PFAS Fluorescence Response Bar Plots

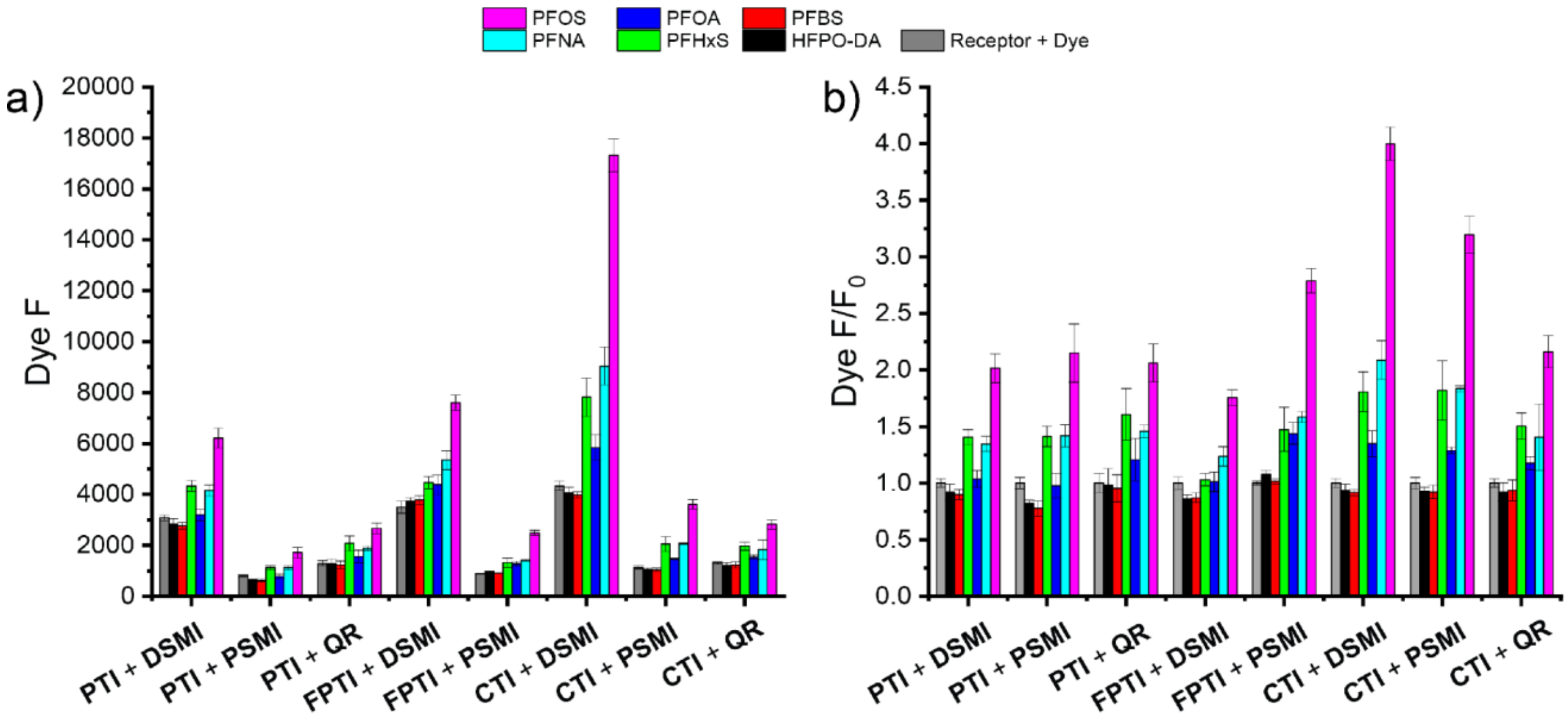

Figure S-23. Bar plots a) F; b) F/F_0 of 0.5 µM dye (**DSMI**, **PSMI**, **QR**) and 4.0 µM **XTI** (**PTI**, **FPTI**, **CTI**) and 10 µM PFAS (PFOS, PFOA, PFNA, PFHxS, PFBS, and HFPO-DA) in 20 mM Tris-HCl buffer at pH 7.4.

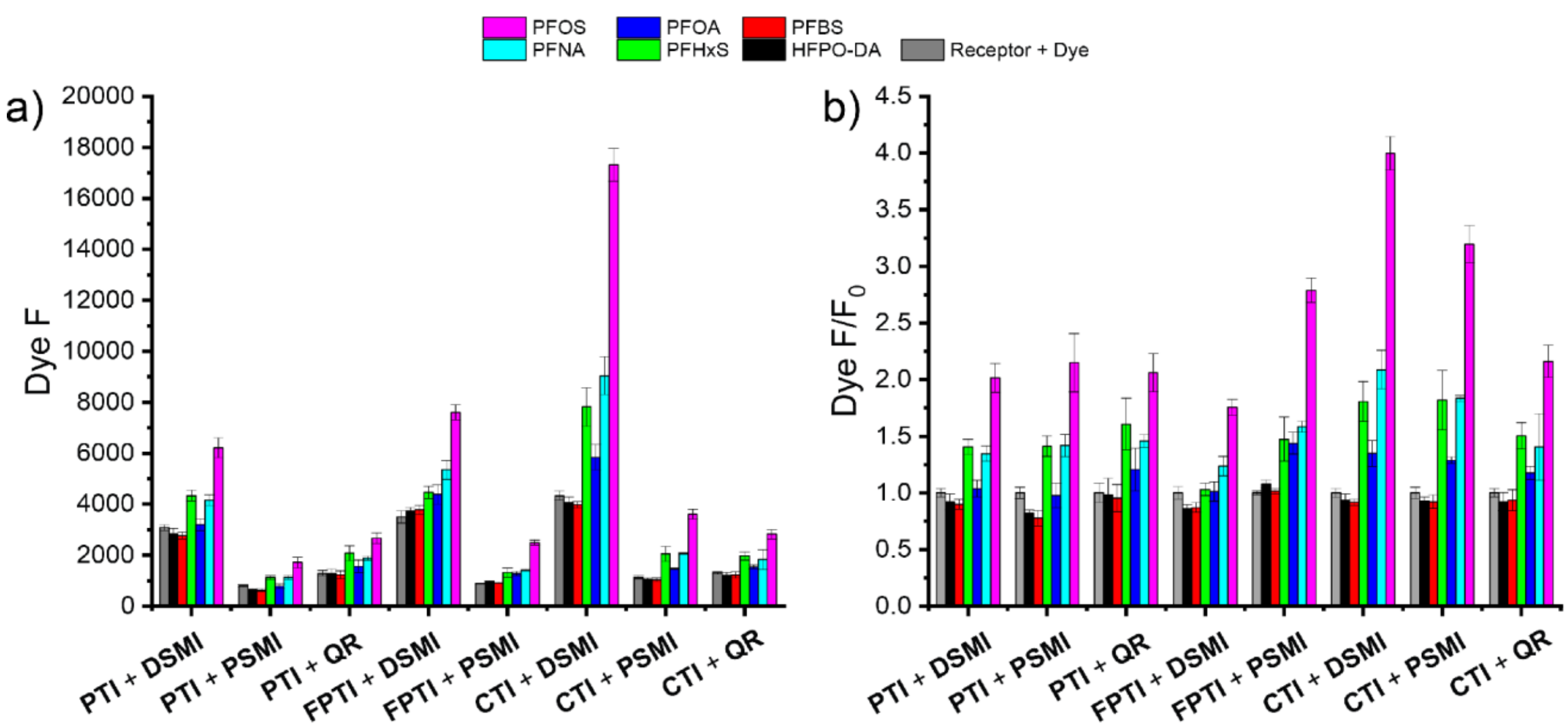

Figure S-24. Bar plots a) F; b) F/F_0 of 0.5 µM dye (**DSMI**, **PSMI**, **QR**) and 4.0 µM **XTI** (**PTI**, **FPTI**, **CTI**) and 10 µM PFAS (PFOS, PFOA, PFNA, PFHxS, PFBS, and HFPO-DA) in 20 mM Tris-HCl buffer at pH 7.4 with 10% human saliva.

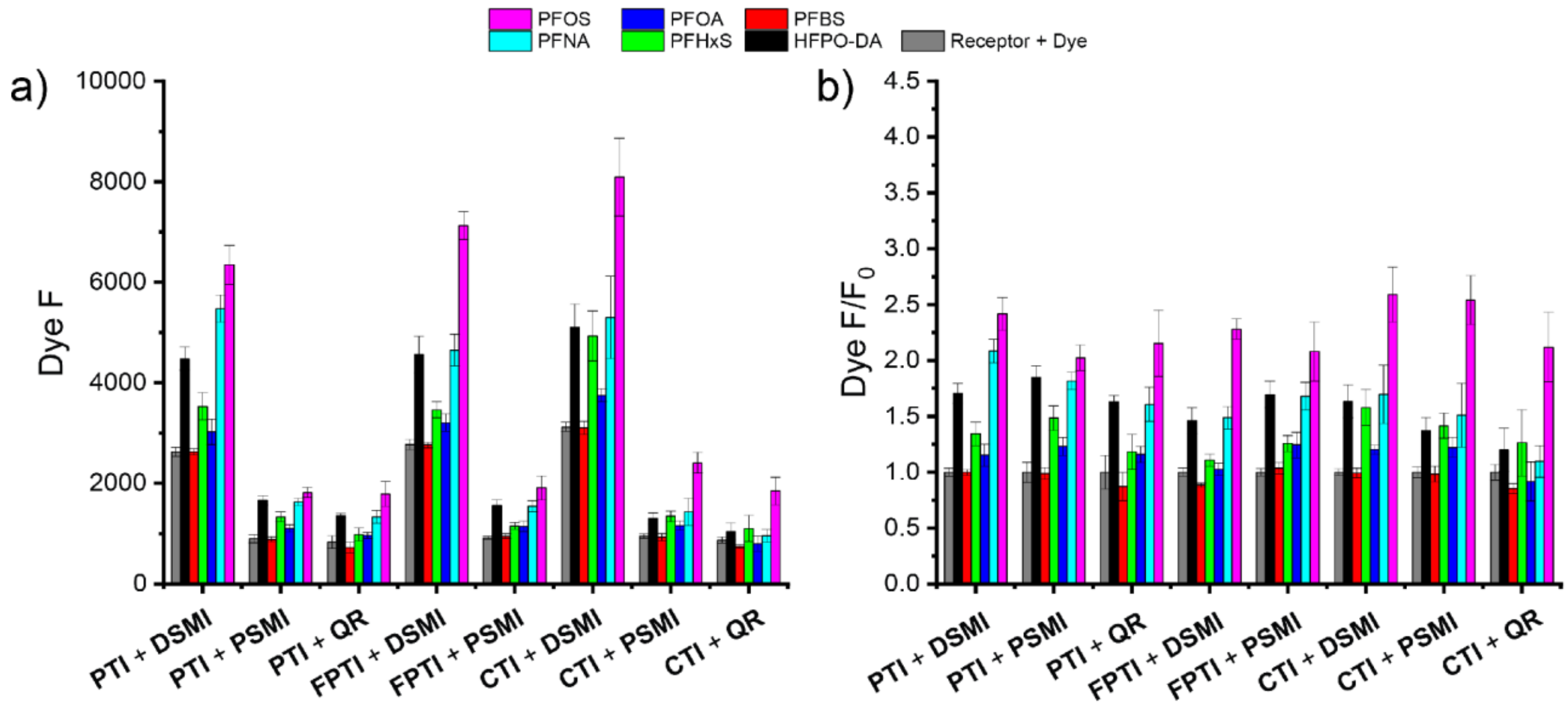

Figure S-25. Bar plots showing a) F; b) F/F$_0$ of dye 0.5 µM dye (**DSMI**, **PSMI**, **QR**) and 4.0 µM **XTI** (**PTI**, **FPTI**, **CTI**) and 10 µM PFAS (PFOS, PFOA, PFNA, PFHxS, PFBS, and HFPO-DA) in 20 mM Tris-HCl buffer at pH 7.4 with 10% artificial urine.

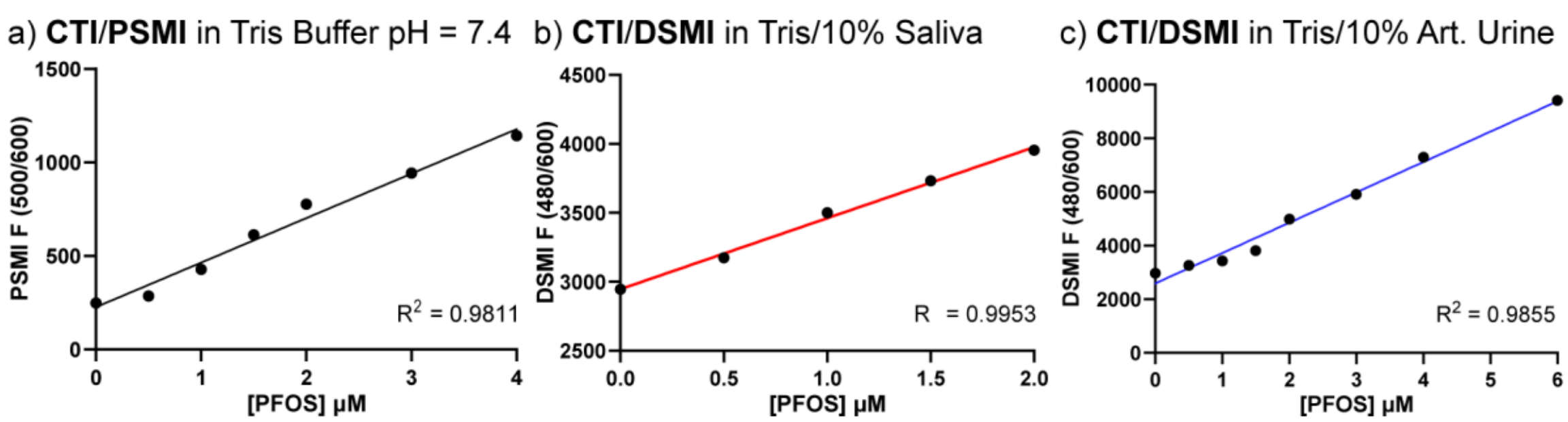

Figure S-26. Linear regression fits to determine slope and goodness of fit for the LOD of PFOS: a) **CTI**/**PSMI** in Tris-HCl Buffer at pH 7.4; b) **CTI**/**DSMI** in Tris Buffer at pH 7.4/10% human saliva; c) **CTI**/**DSMI** in Tris-HCl buffer at pH 7.4/10% artificial urine. [**CTI**] = 4 µM, [**DSMI** or **PSMI**] 0.5 µM, [PFOS] = 0-2, 4, or 6 µM, respectively.

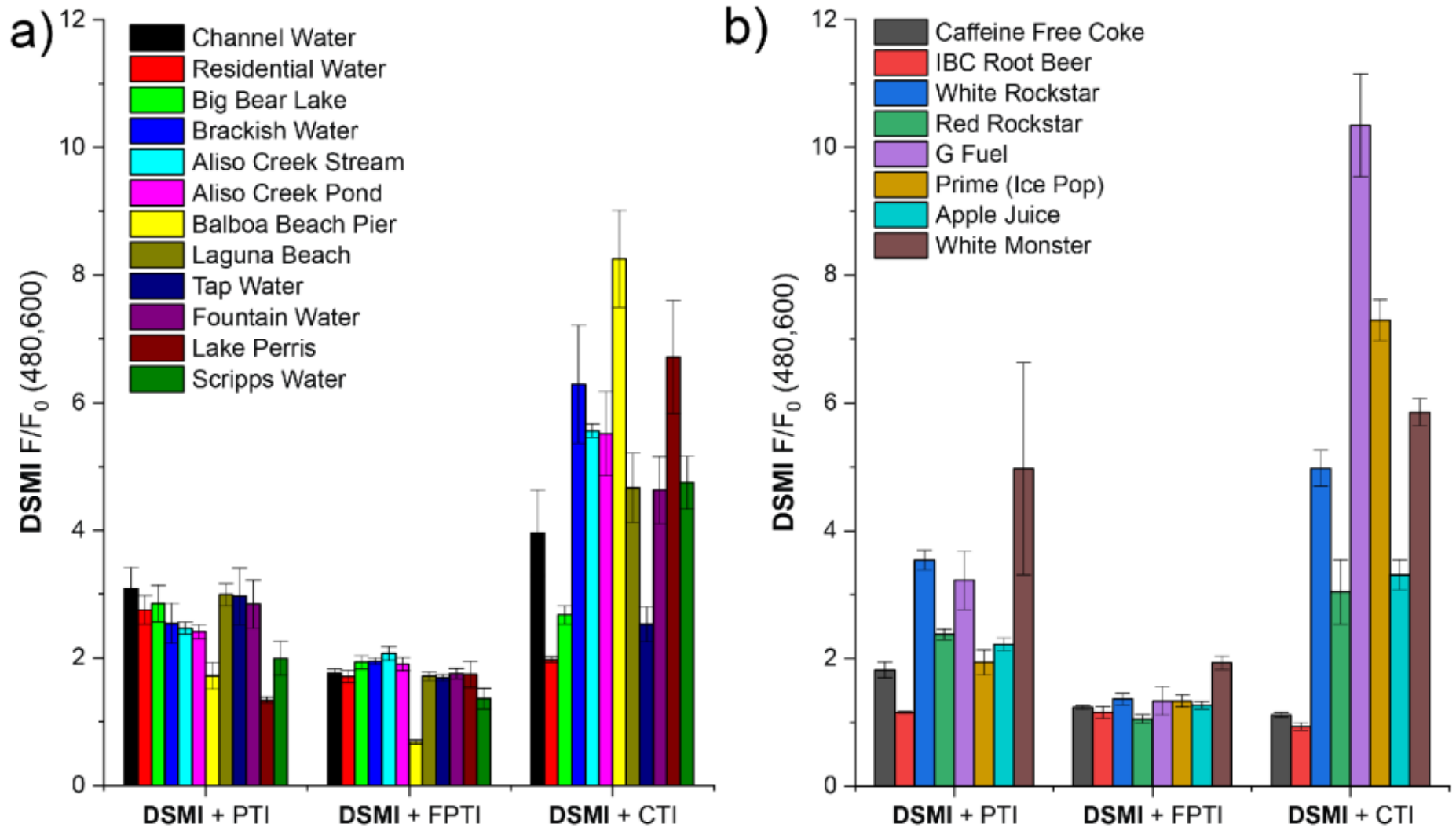

Figure S-27. Bar plots showing 0.5 µM **DSMI** and 4.0 µM **XTI** (**PTI**, **FPTI**, **CTI**) and 20 µM PFOS in 10% aqueous medium in 20 mM Tris-HCl: a) 10% local body of water; b) 10% commercial beverages.

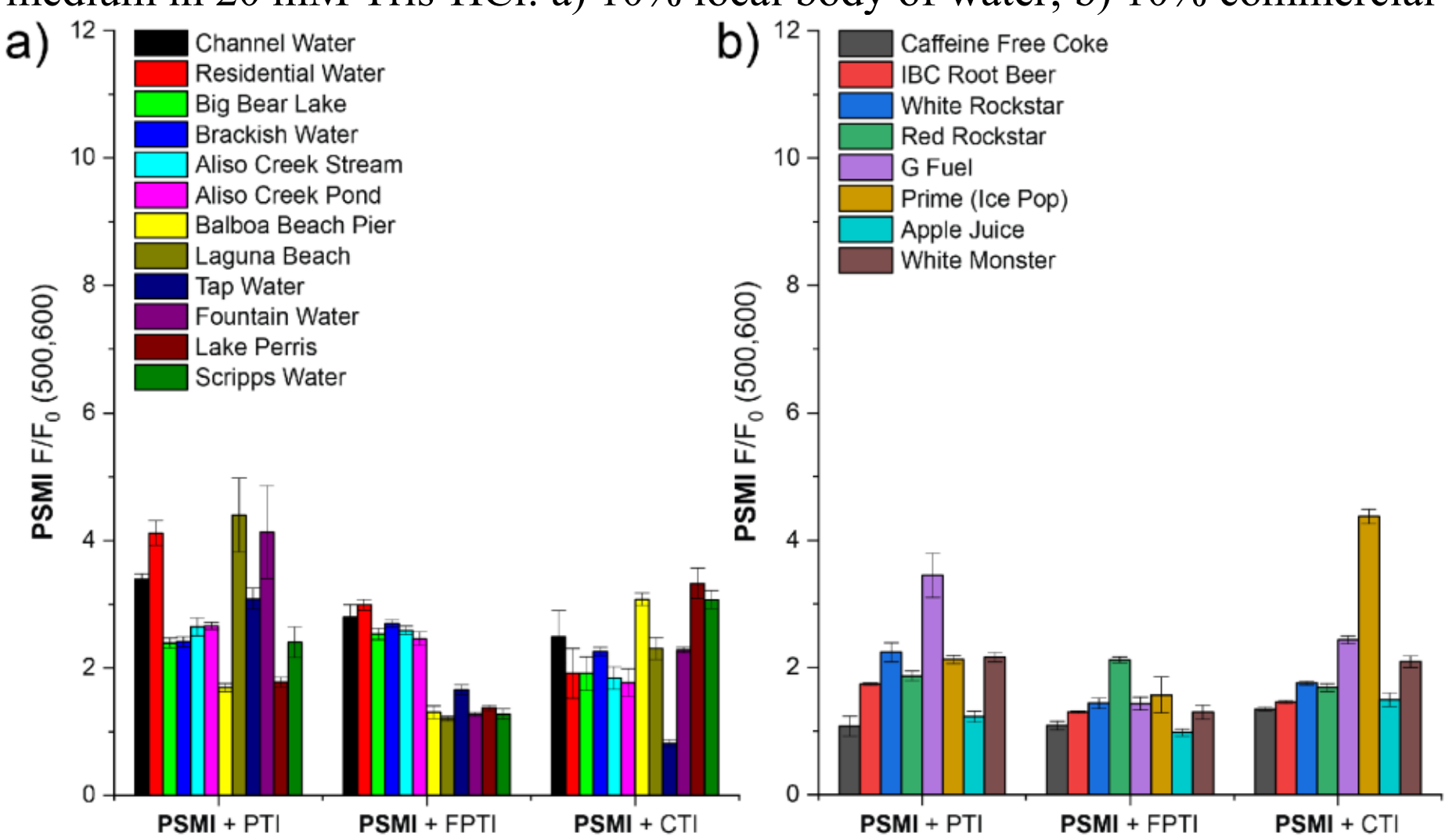

Figure S-28. Bar plots showing 0.5 µM **PSMI** and 4.0 µM **XTI** (**PTI**, **FPTI**, **CTI**) and 20 µM PFOS in 10% aqueous medium in 20 mM Tris-HCl: a) 10% local body of water; b) 10% commercial beverages.

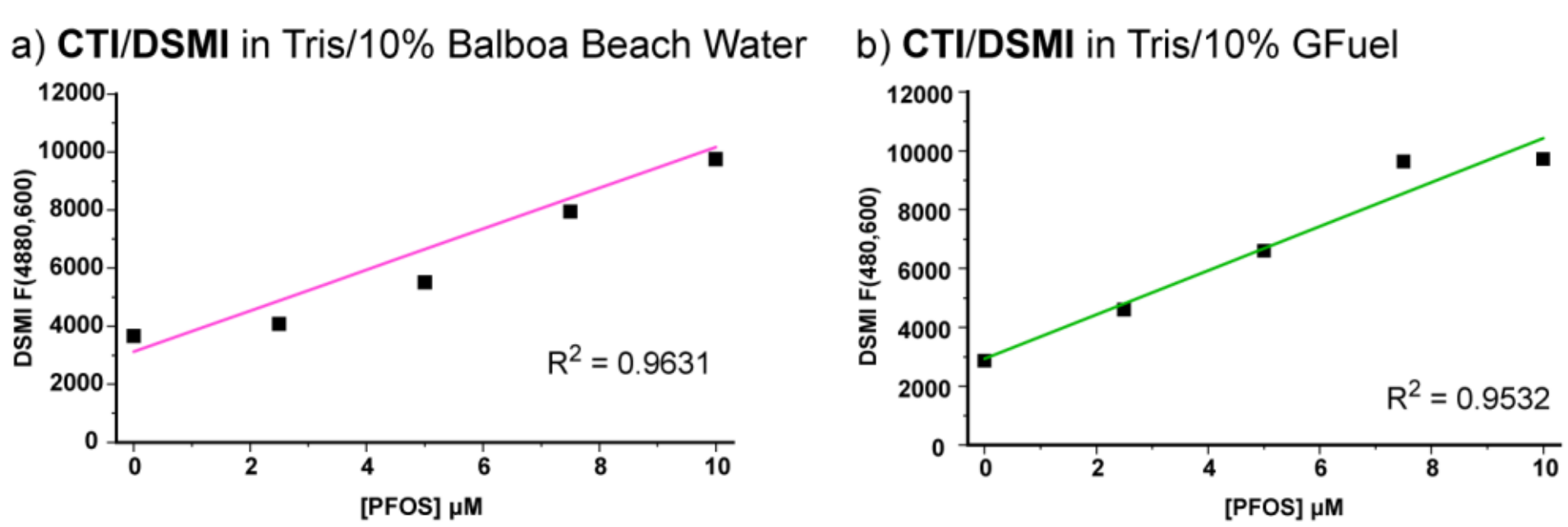

Figure S-29. Linear regression fits were used to determine the slope and goodness of fit for the LOD of PFOS: a) **CTI**/**DSMI** in Tris-HCl Buffer at pH 7.4/10% Balboa Beach water; b) **CTI**/**DSMI** in Tris Buffer at pH 7.4/10% GFuel. [**CTI**] = 4 µM, [**DSMI**] 0.5 µM, [PFOS] = 0-20 µM, respectively.

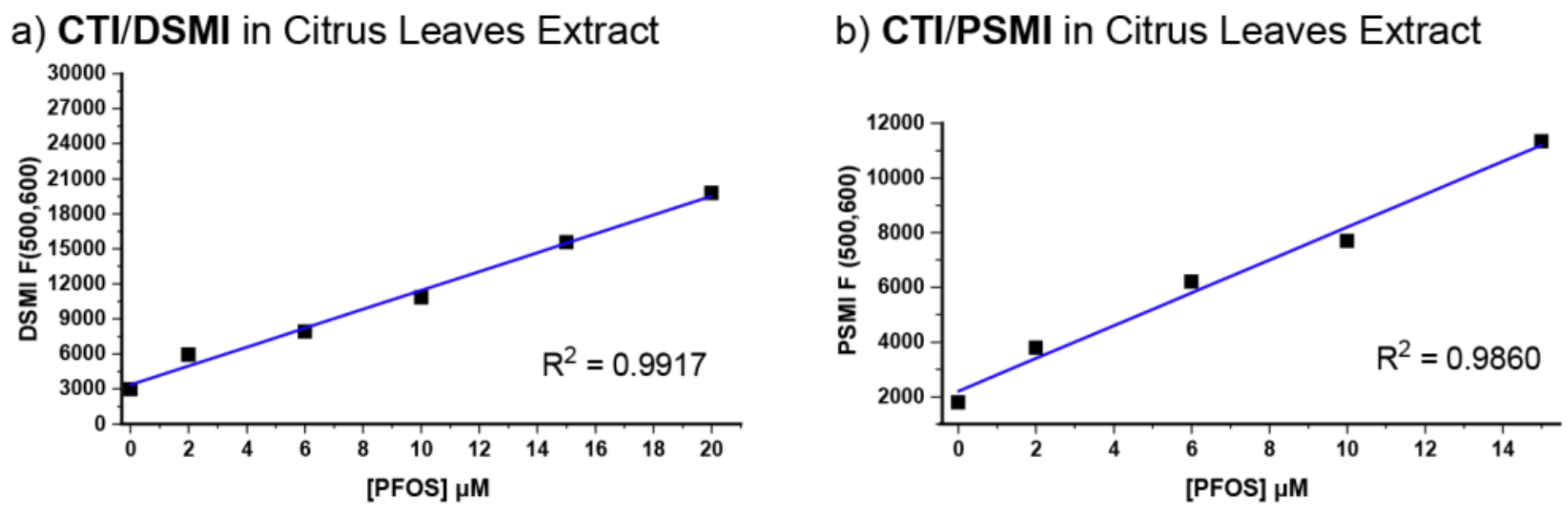

Figure S-30. Linear regression fits were used to determine the slope and goodness of fit for the LOD of PFOS in extracts of citrus leaves. The citrus leaves were taken from Washington navel orange tree on the University of California, Riverside campus: a) **CTI/DSMI**; b) **CTI/PSMI** in 0-0.12 mg/mL of extracts of citrus leaves [**CTI**] = 4 µM, [**DSMI**] 0.5 µM, [PFOS] = 0-20 µM, respectively.

Table S-2. Compiled Limit of Detection (LOD) of PFOS from the dye•**CTI** system in Tris Buffer (pH = 7.4) and various aqueous media (local sources of water and beverages).

Water Conditions	Receptor•Dye	LOD (nM)
Tris Buffer pH=7.4	**CTI•PSMI**	48
Tris/10% Human Saliva	**CTI•DSMI**	970
Tris/10% Artificial Urine	**CTI•DSMI**	164
Tris/10% Balboa Beach Water	**CTI•DSMI**	249
Tris/10% Gfuel	**CTI•DSMI**	117
Citrus leaves extract	**CTI•DSMI**	176
Citrus leaves extract	**CTI•PSMI**	152

4. ^{1}H and ^{19}F NMR Spectral Titrations

4.1 ^{1}H NMR Spectral Titrations of PTI, FPTI, and CTI with PFOA or PFOS

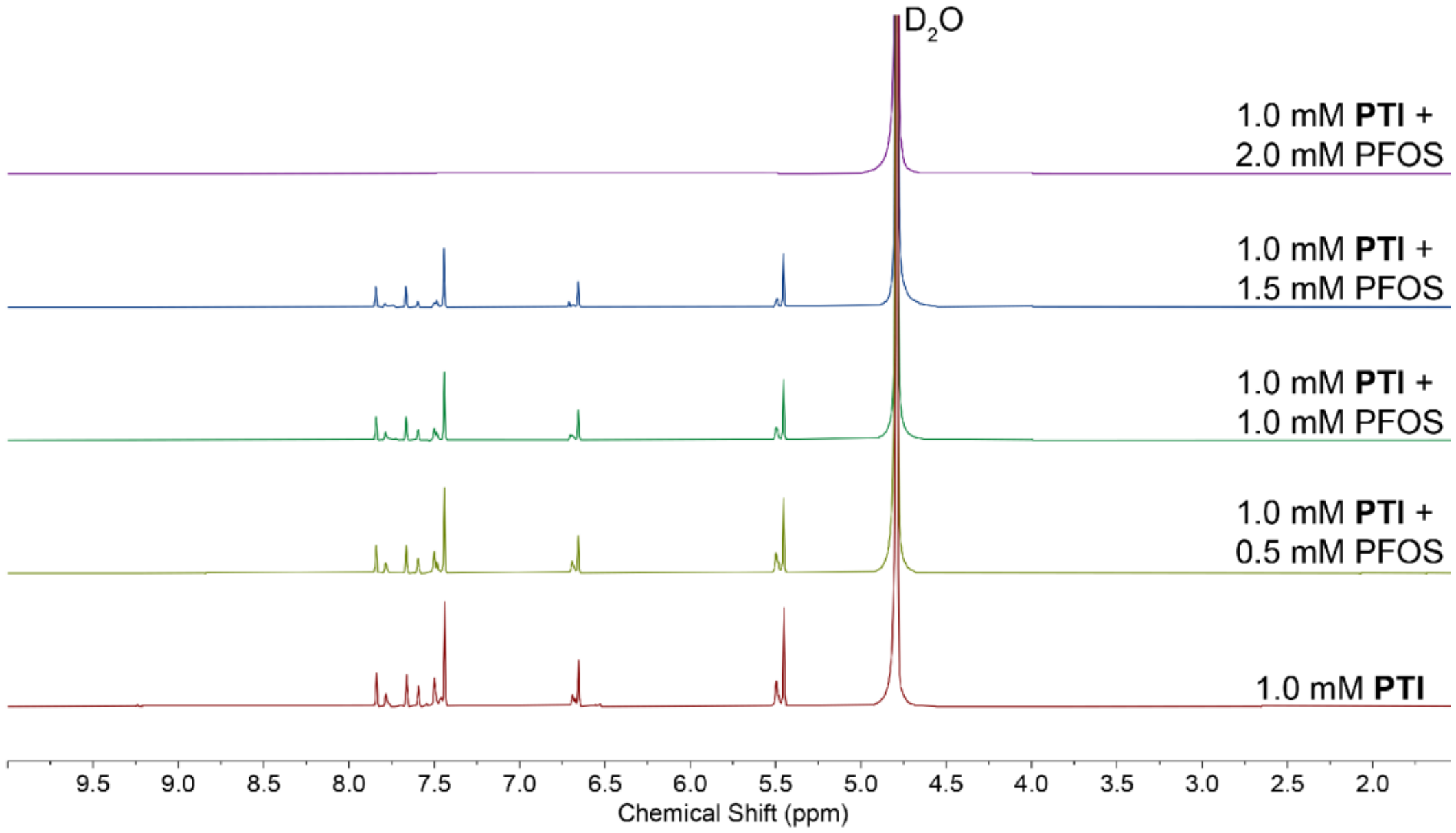

Figure S-31. Full ^{1}H NMR spectral titration of **PTI** (1 mM) and PFOS (0-2 mM) in D_2O (600 MHz, 298K). PFOS added in aliquots of 0.5 molar equivalents each.

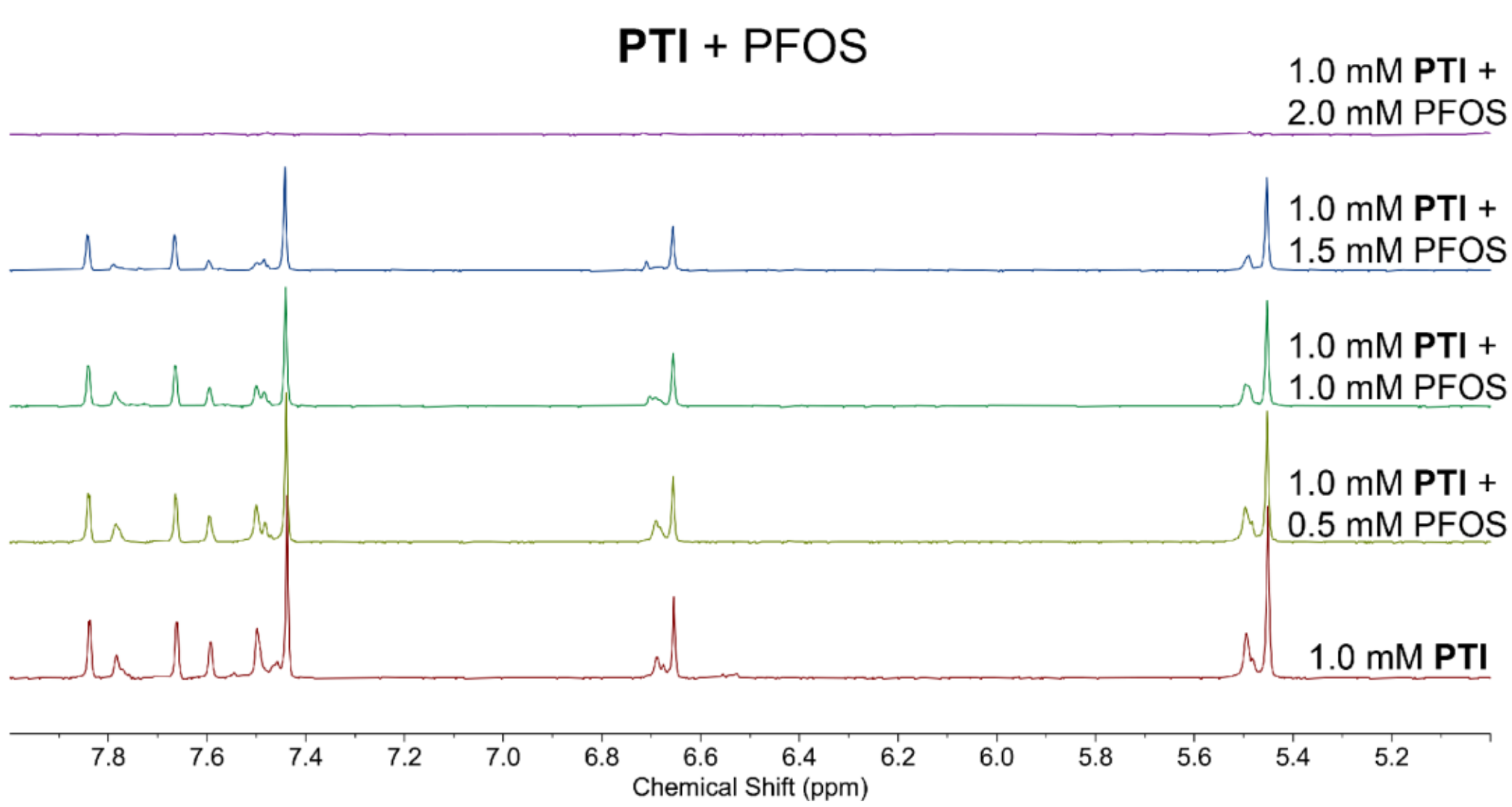

Figure S-32. ^{1}H NMR spectral titration of **PTI** (1 mM) and PFOS (0-2 mM) in D_2O (600 MHz, 298K). PFOS added in aliquots of 0.5 molar equivalents each. Only visible changes are shown, titrations referenced to the HOD peak at 4.79 ppm.

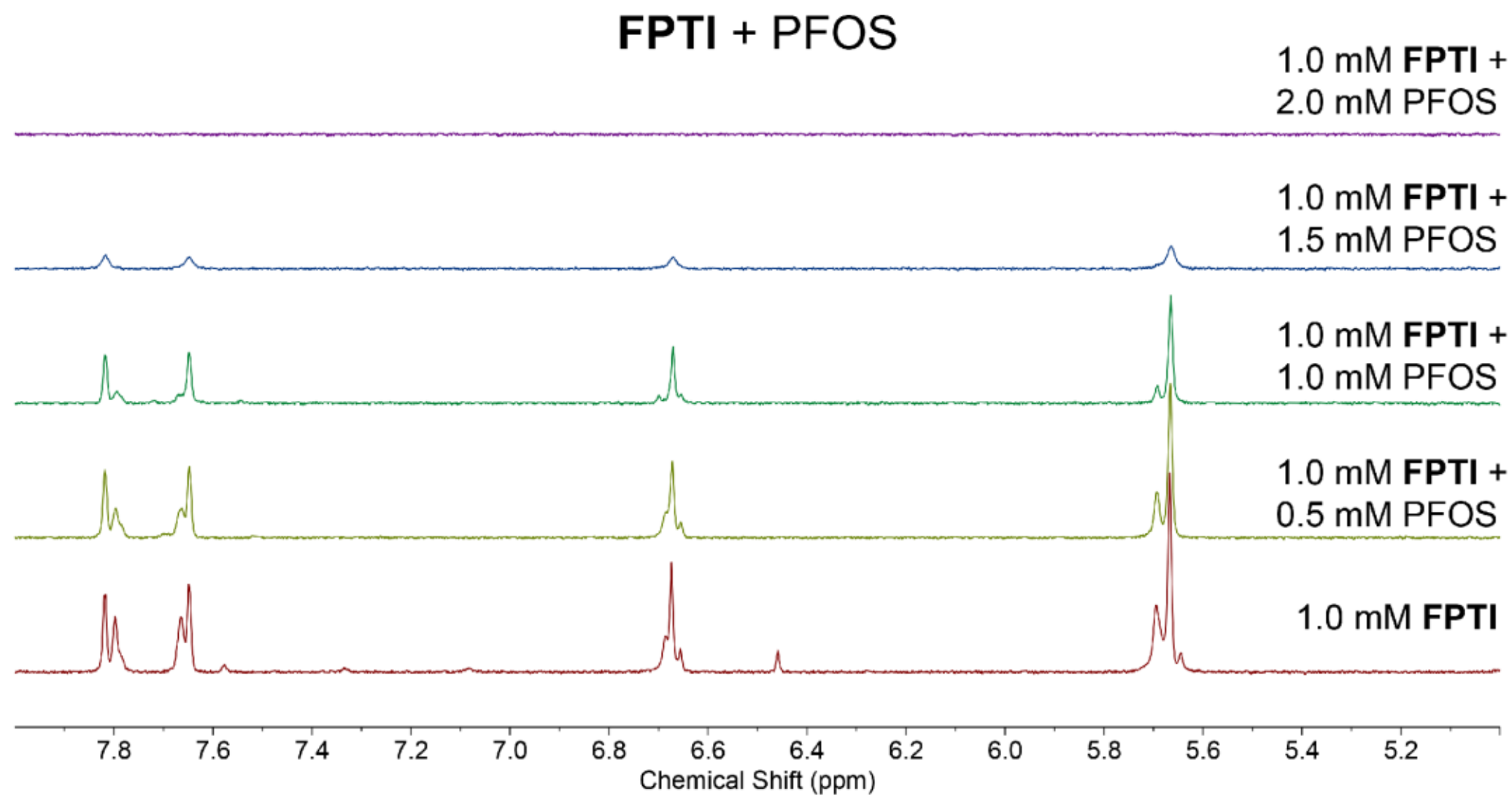

Figure S-33. ^{1}H NMR spectral titration of **FPTI** (1 mM) and PFOS (0-2 mM) in D_2O (600 MHz, 298K). PFOS added in aliquots of 0.5 molar equivalents each. Only visible changes are shown; titrations referenced to the HOD peak at 4.79 ppm.

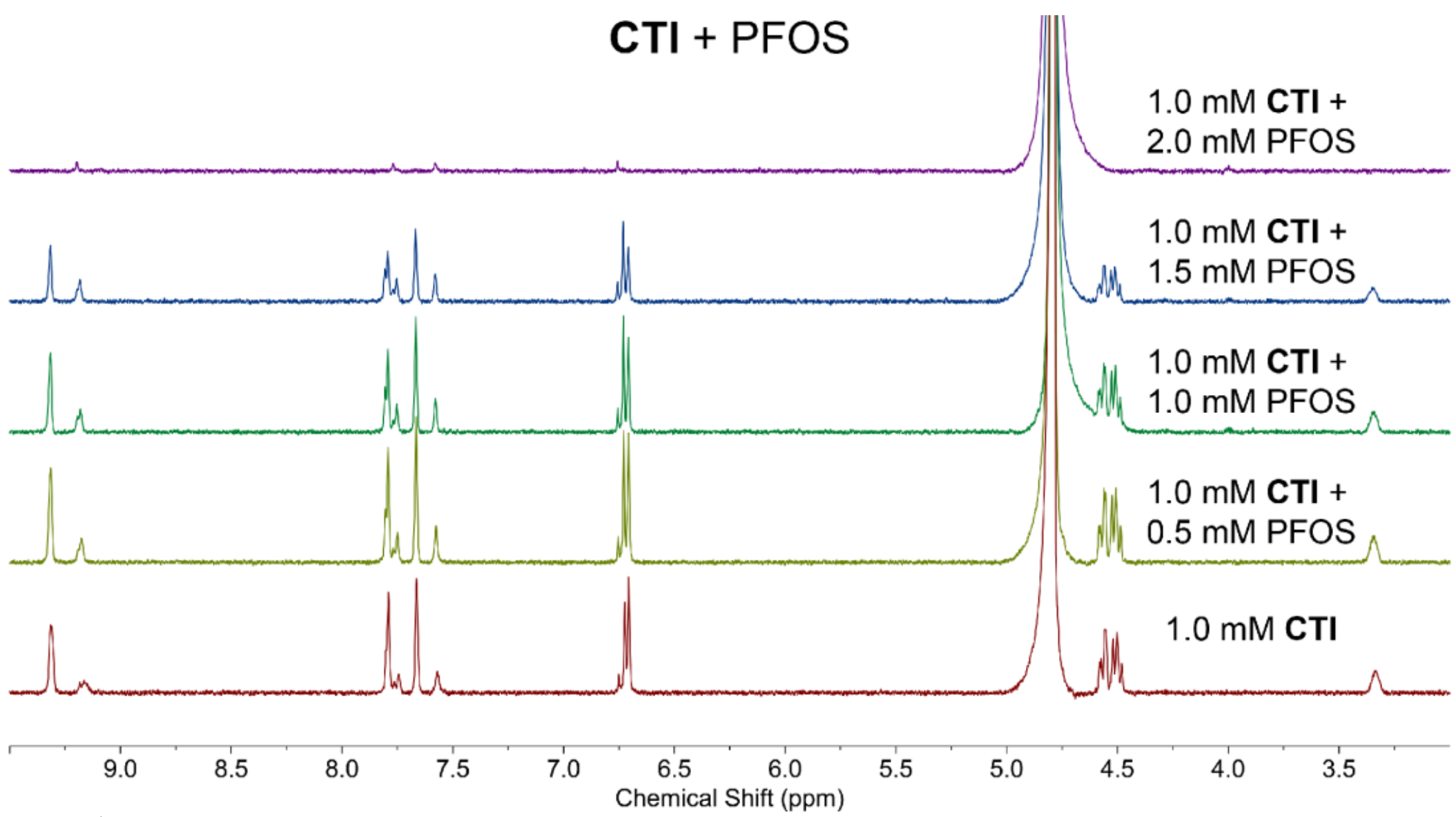

Figure S-34. ^{1}H NMR spectral titration of **CTI** (1 mM) and PFOS (0-2 mM) in D_2O (600 MHz, 298K). PFOS added in aliquots of 0.5 molar equivalents each. Only visible changes are shown; titrations referenced to the HOD peak at 4.79 ppm.

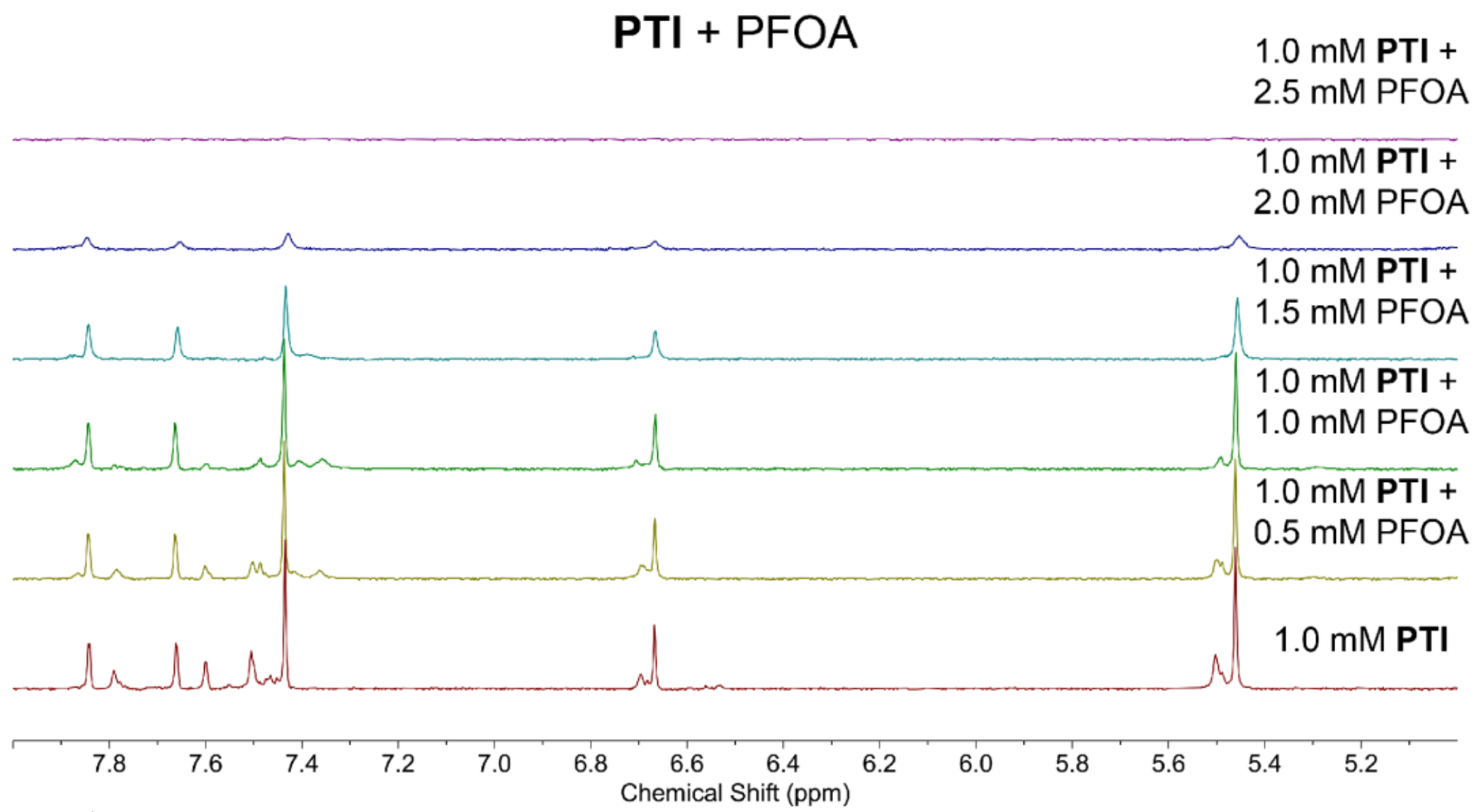

Figure S-35. ^{1}H NMR spectral titration of **PTI** (1mM) and PFOA (0-2 mM) in D_2O (600 MHz, 298K). PFOA added in aliquots of 0.5 molar equivalents each. Only visible changes are shown; titrations referenced to the HOD peak at 4.79 ppm.

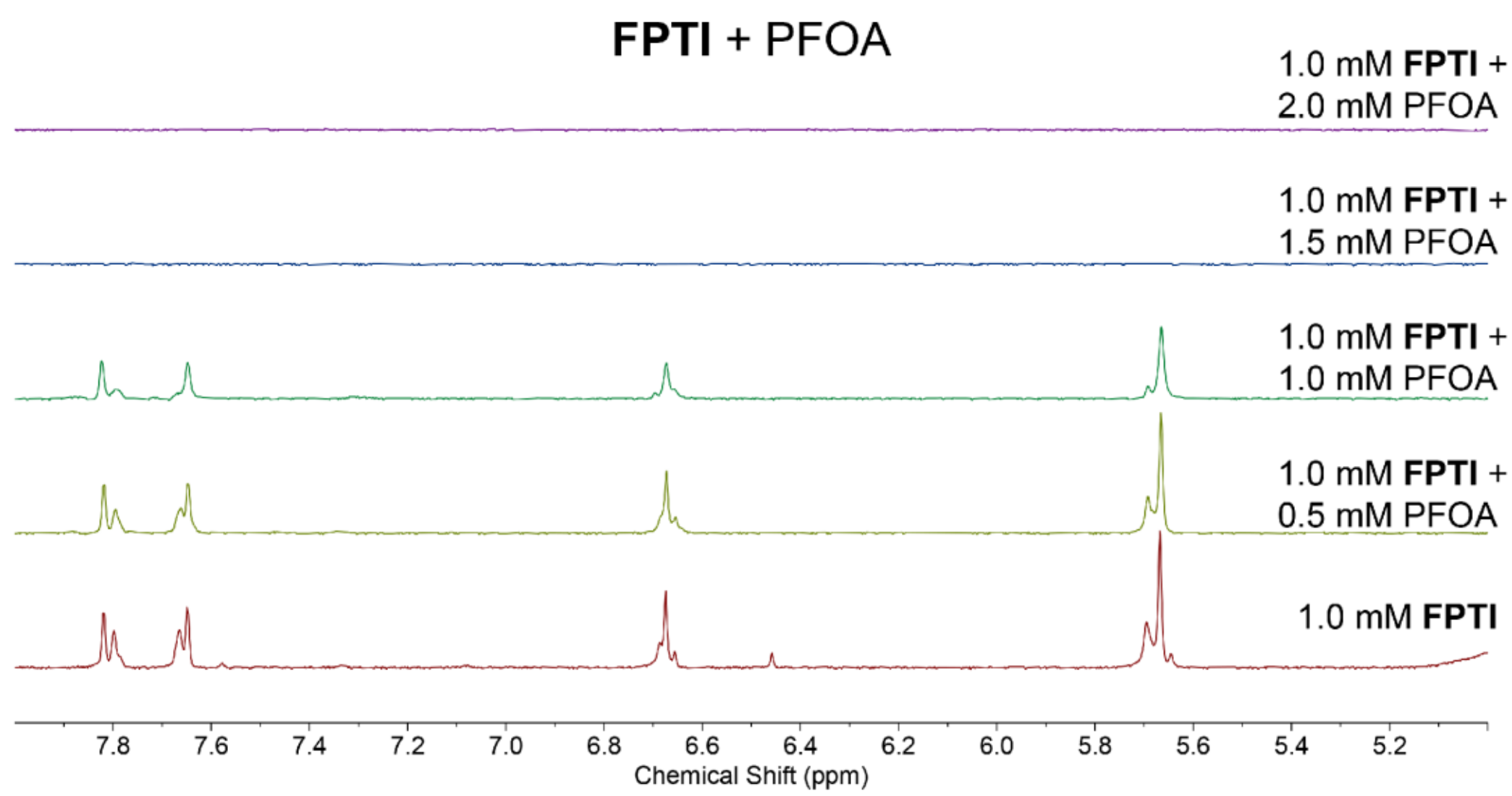

Figure S-36. ^{1}H NMR spectral titration of **FPTI** (1 mM) and PFOA (0-2 mM) in D_2O (600 MHz, 298K). PFOA added in aliquots of 0.5 molar equivalents each. Only visible changes are shown; titrations referenced to the HOD peak at 4.79 ppm.

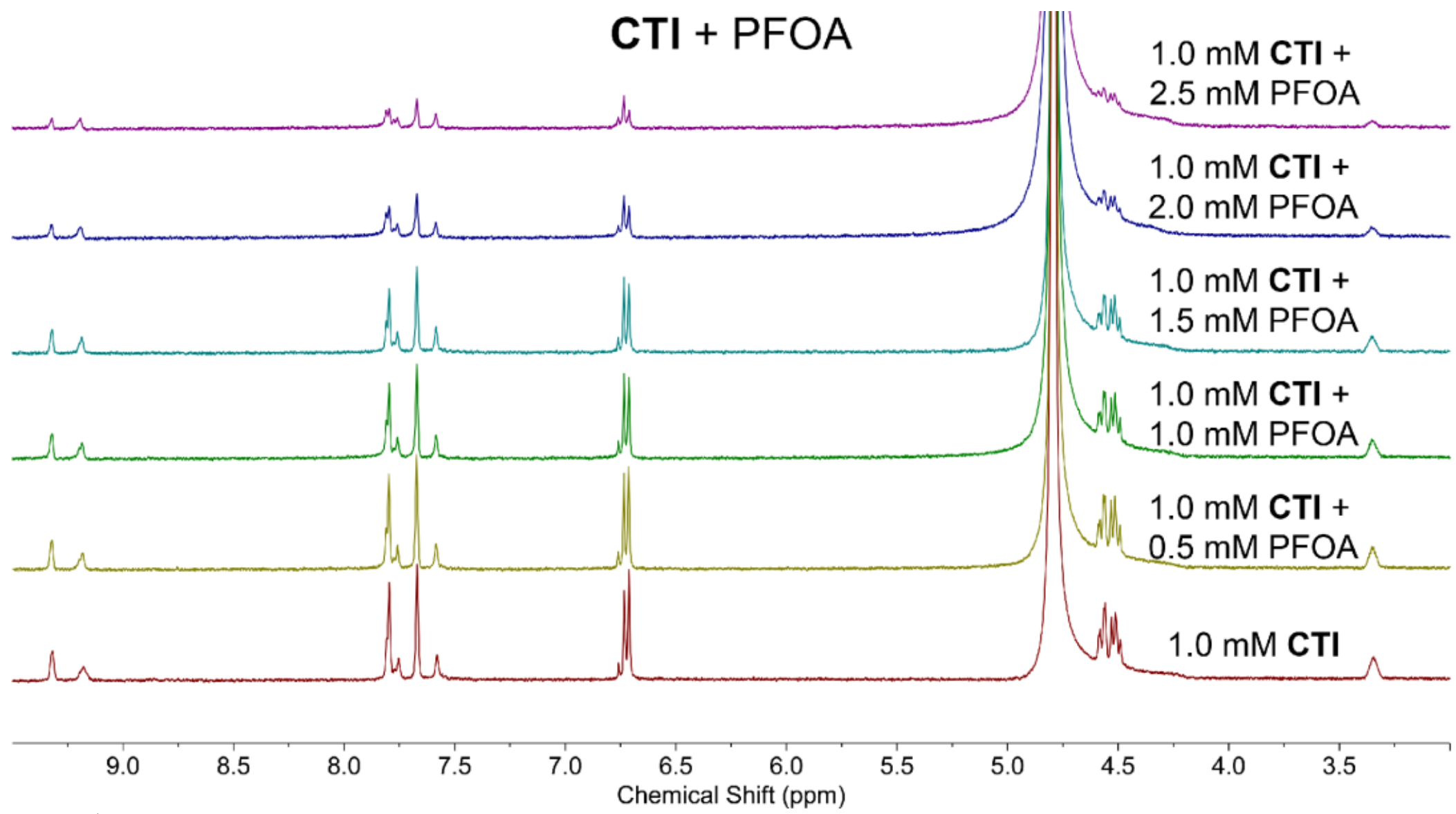

Figure S-37. ^{1}H NMR spectral titration of **CTI** (1 mM) and PFOA (0-2 mM) in D_2O (600 MHz, 298K). PFOA added in aliquots of 0.5 molar equivalents each. Only visible changes are shown; titrations referenced to the HOD peak at 4.79 ppm.

4.2 ^{19}F NMR Spectral Titrations of PTI, FPTI, and CTI with PFOA or PFOS

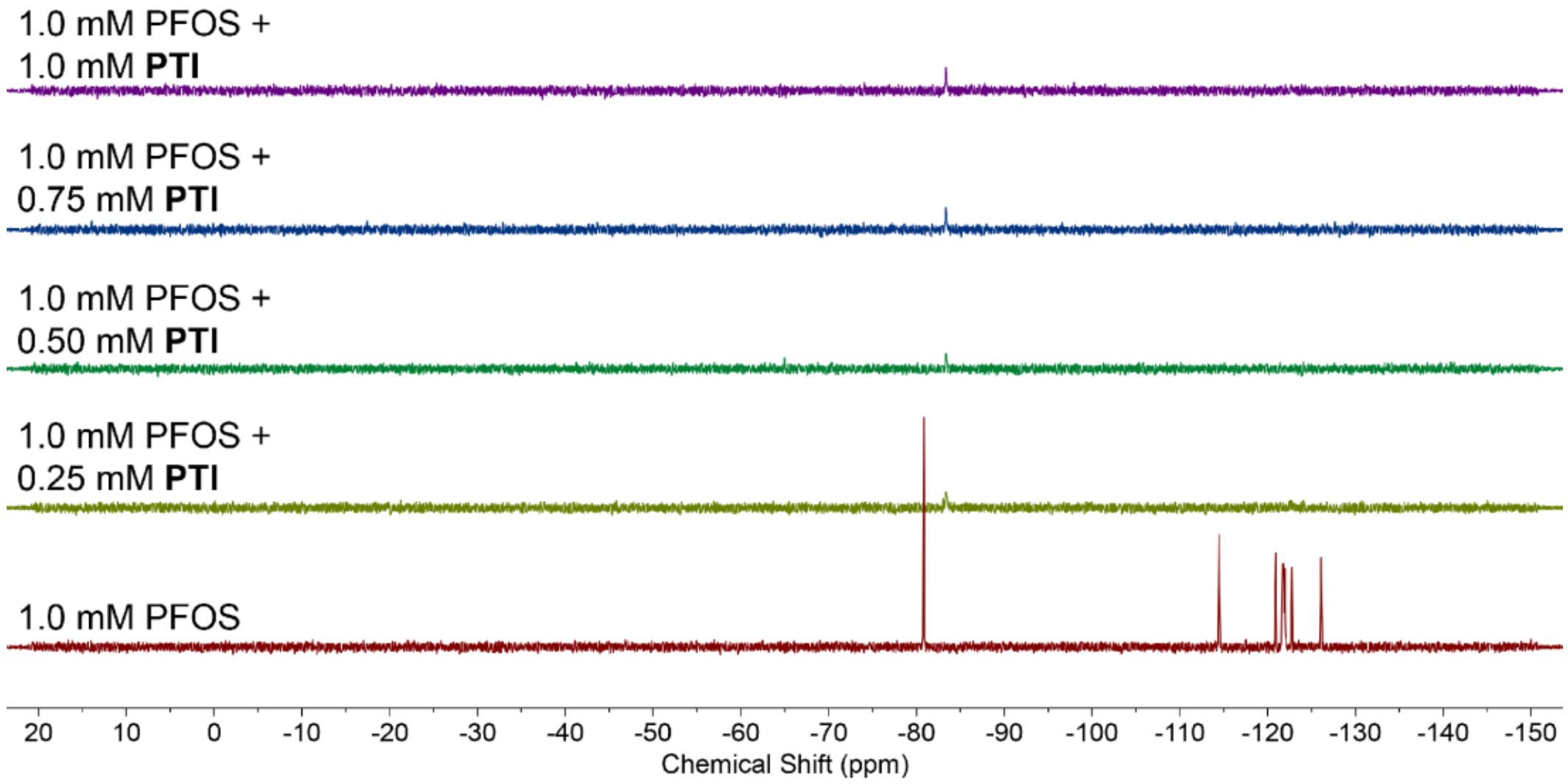

Figure S-38. Full ^{19}F NMR spectral titration of PFOS (1 mM) and **PTI** (0-1.0 mM) in D_2O (564 MHz, 298K). **PTI** added in aliquots of 0.25 molar equivalents each.

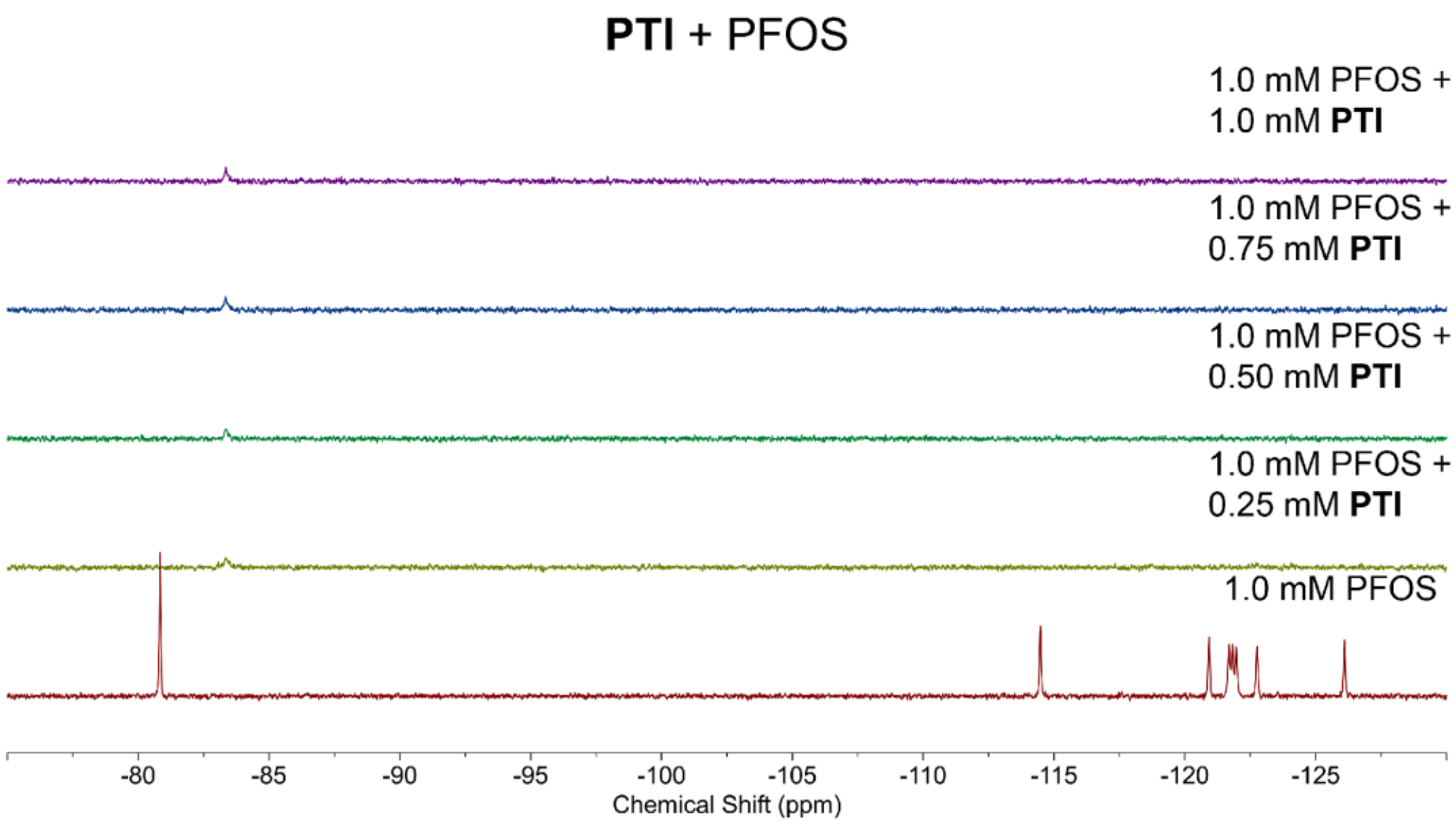

Figure S-39. ^{19}F NMR spectral titration of PFOS (1 mM) and **PTI** (0-1.0 mM) in D_2O (564 MHz, 298K). **PTI** added in aliquots of 0.25 molar equivalents each. Only visible changes are shown.

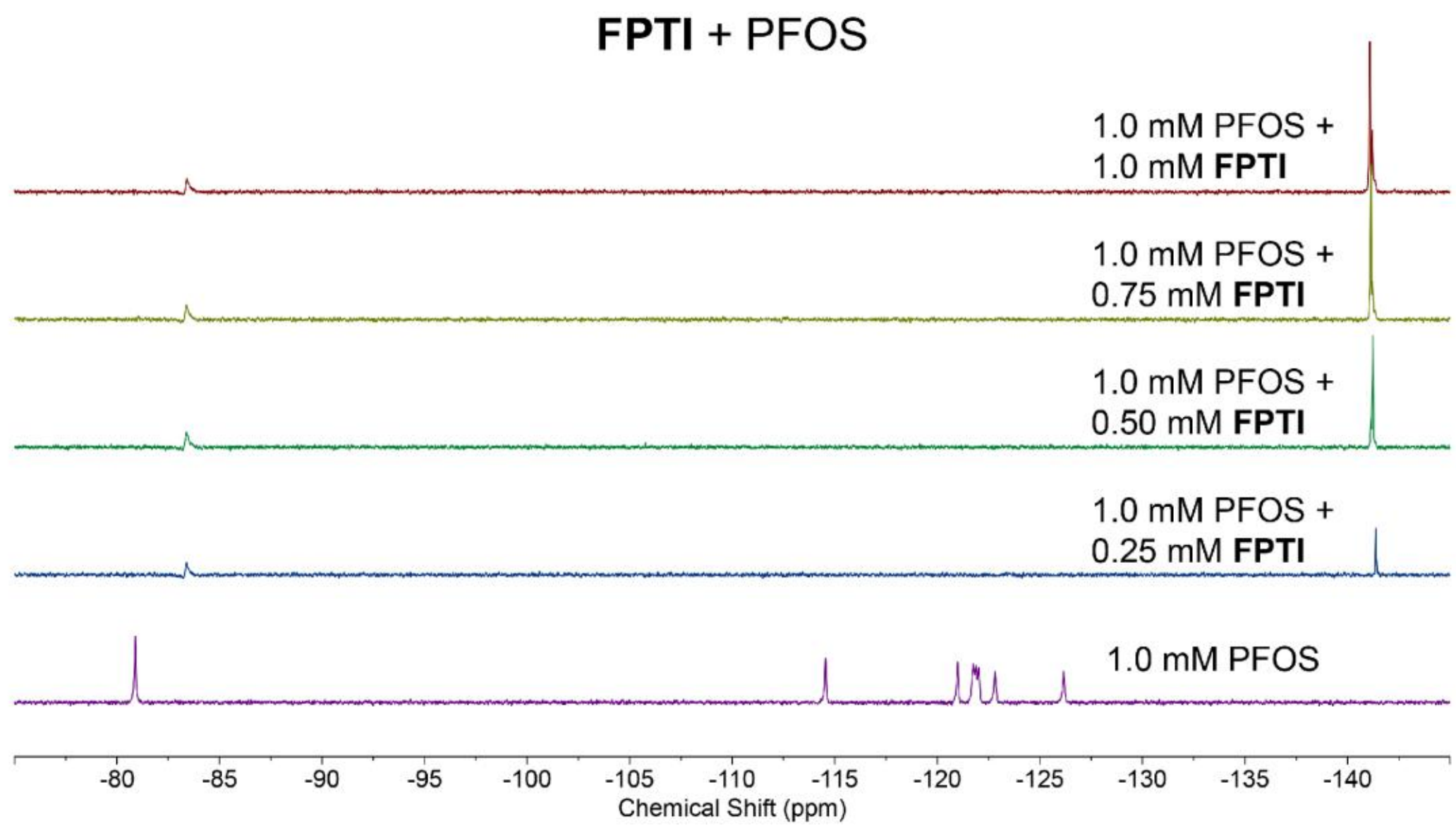

Figure S-40. ^{19}F NMR spectral titration of PFOS (1 mM) and **FPTI** (0-1.0 mM) in D_2O (564 MHz, 298K). **FPTI** added in aliquots of 0.25 molar equivalents each. Only visible changes are shown.

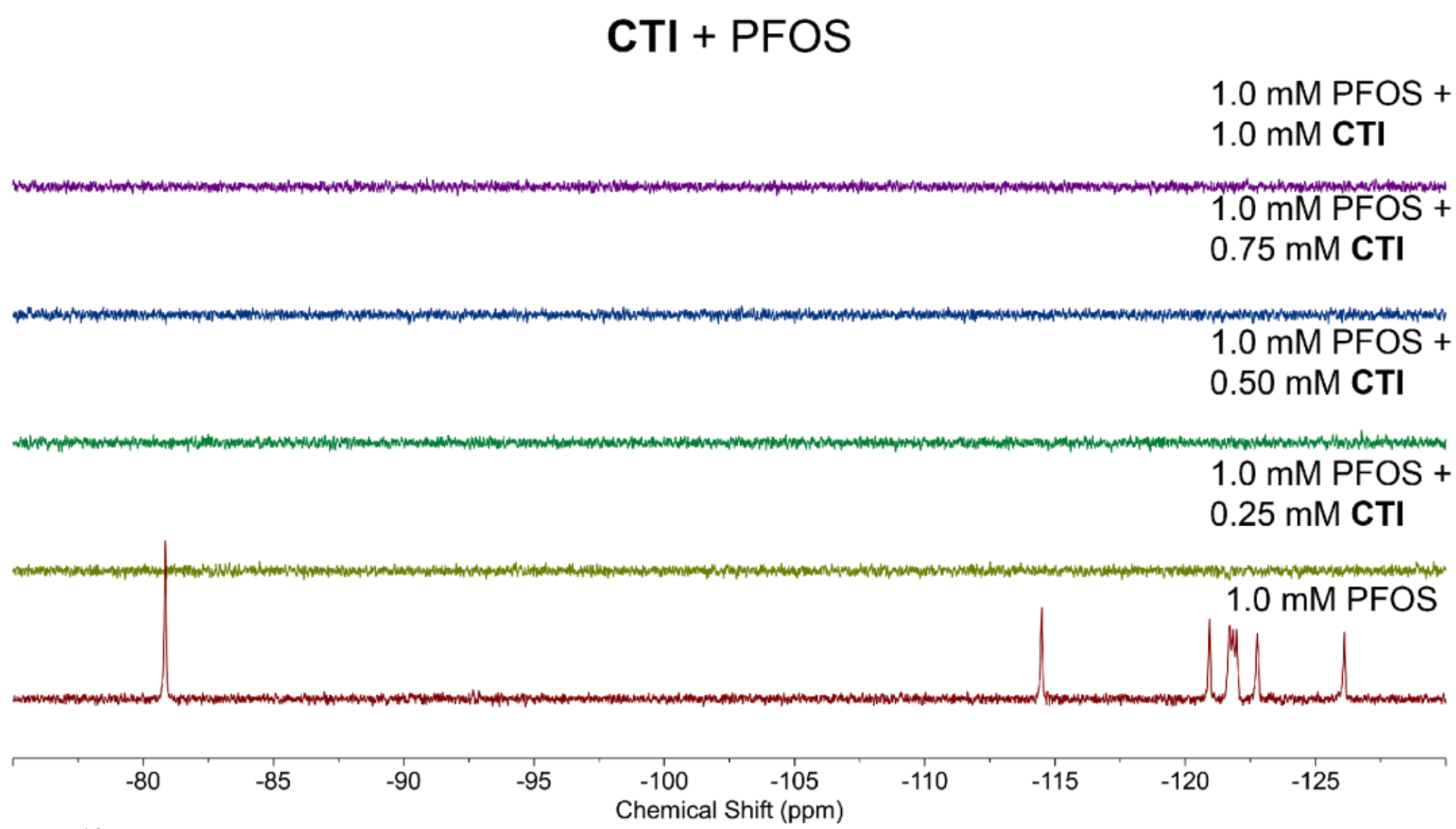

Figure S-41. ^{19}F NMR spectral titration of PFOS (1 mM) and **CTI** (0-1.0 mM) in D_2O (564 MHz, 298K). **CTI** added in aliquots of 0.25 molar equivalents each. Only visible changes are shown.

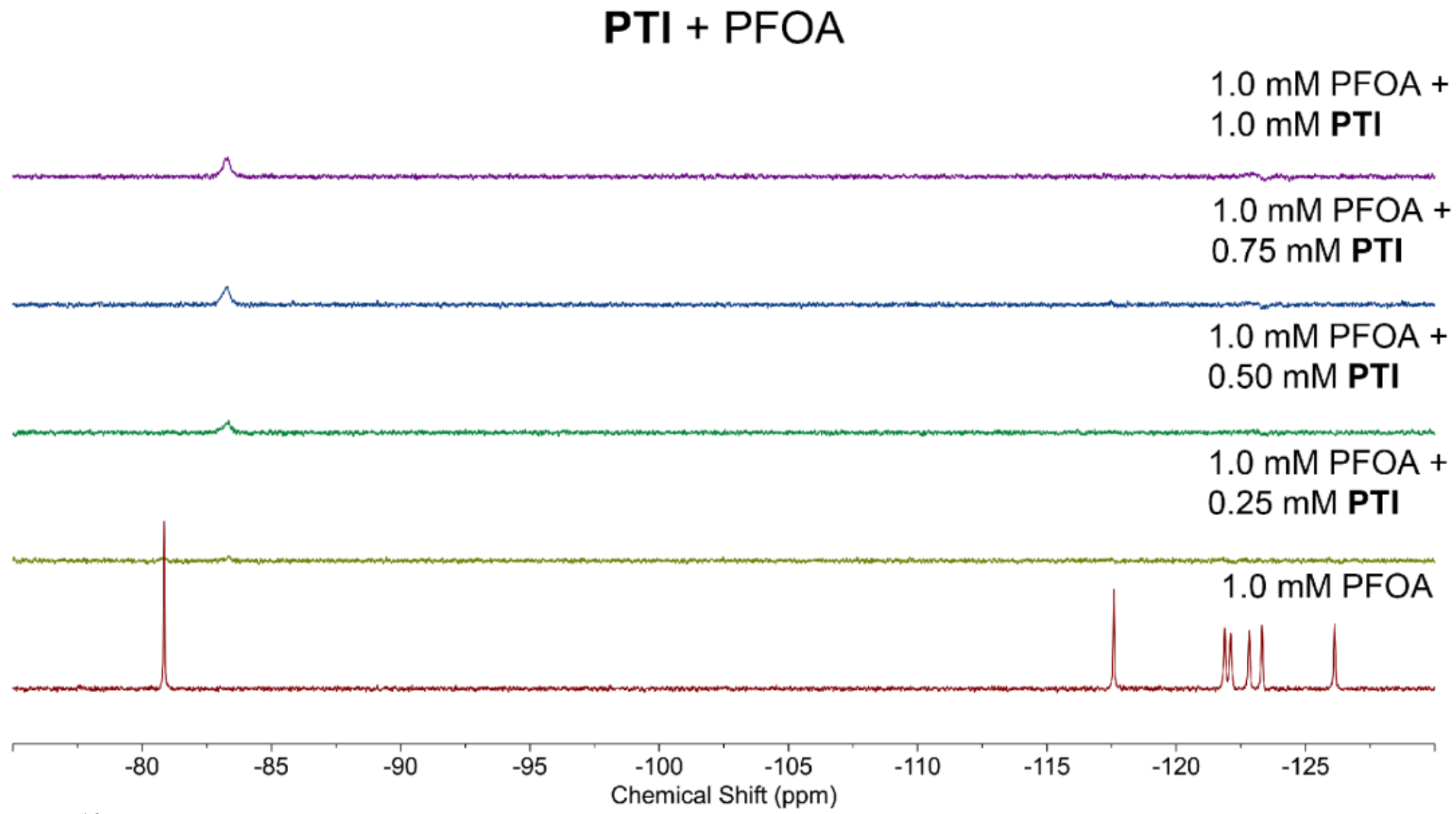

Figure S-42. ^{19}F NMR spectral titration of PFOA (1 mM) and **PTI** (0-1.0 mM) in D_2O (564 MHz, 298K). **PTI** added in aliquots of 0.25 molar equivalents each. Only visible changes are shown.

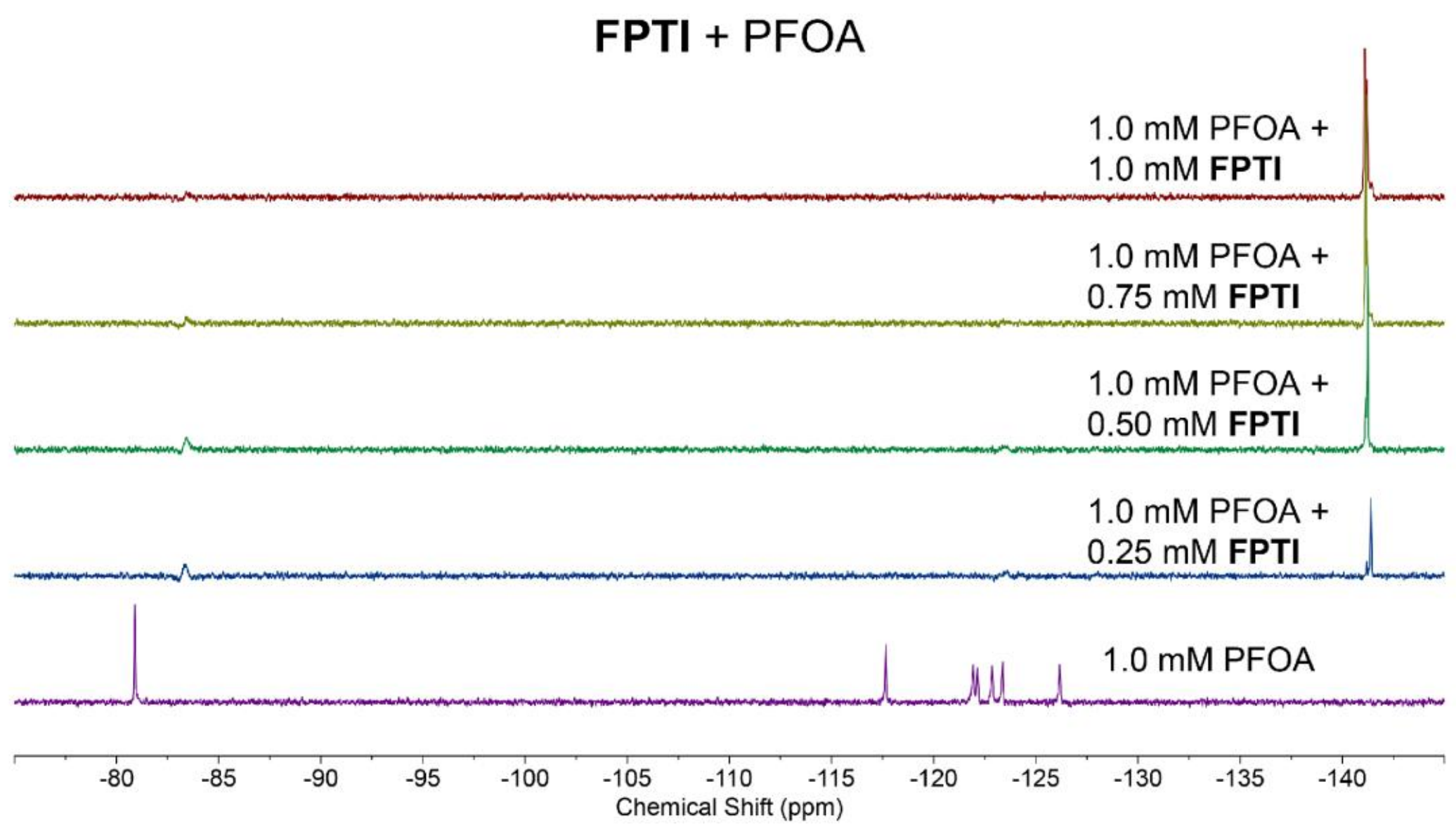

Figure S-43. ^{19}F NMR spectral titration of PFOA (1 mM) and **FPTI** (0-1.0 mM) in D_2O (564 MHz, 298K). **FPTI** added in aliquots of 0.25 molar equivalents each. Only visible changes are shown.

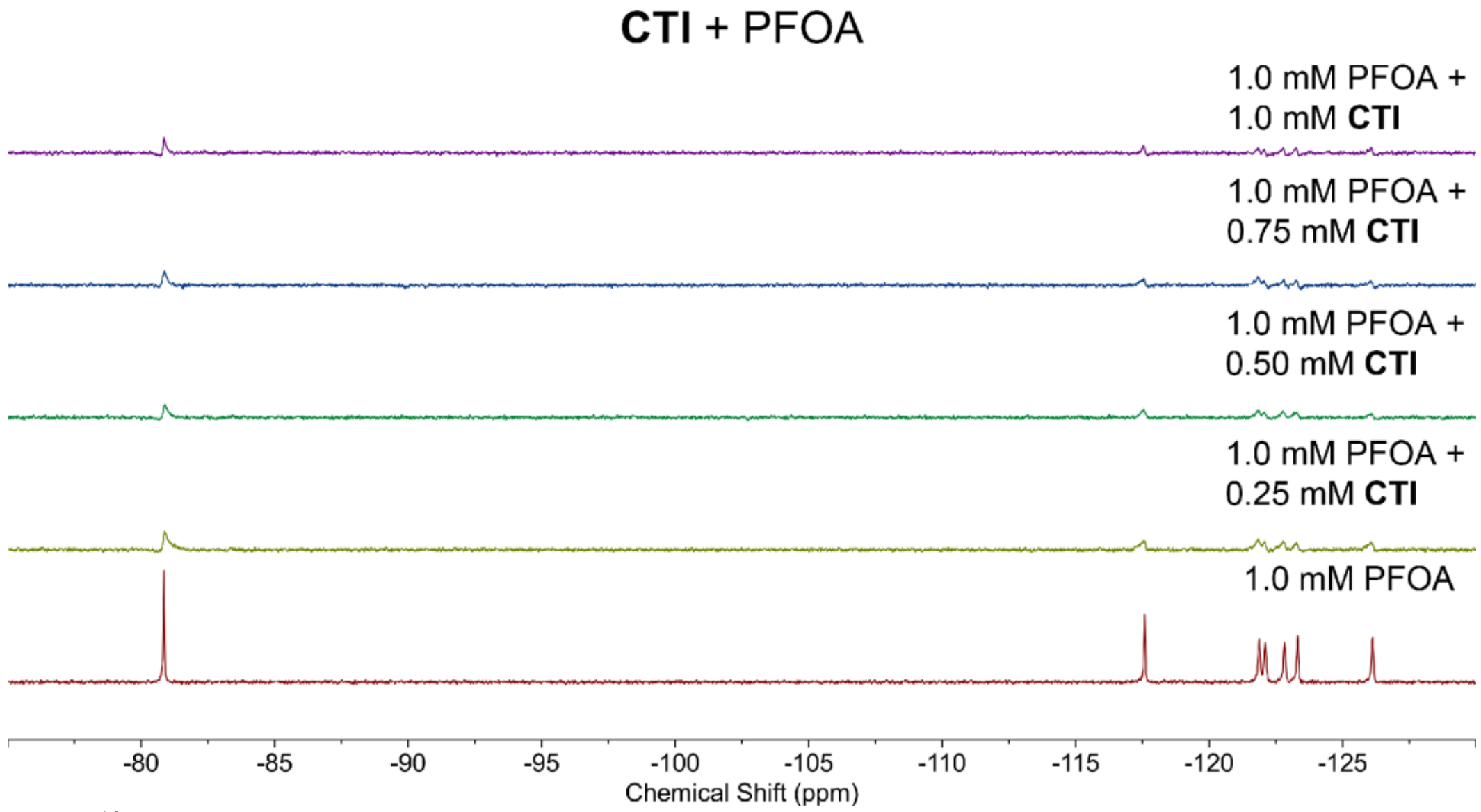

Figure S-44. ^{19}F NMR spectral titration of PFOA (1 mM) and **CTI** (0-1.0 mM) in D_2O (564 MHz, 298K). **CTI** added in aliquots of 0.25 molar equivalents each. Only visible changes are shown.

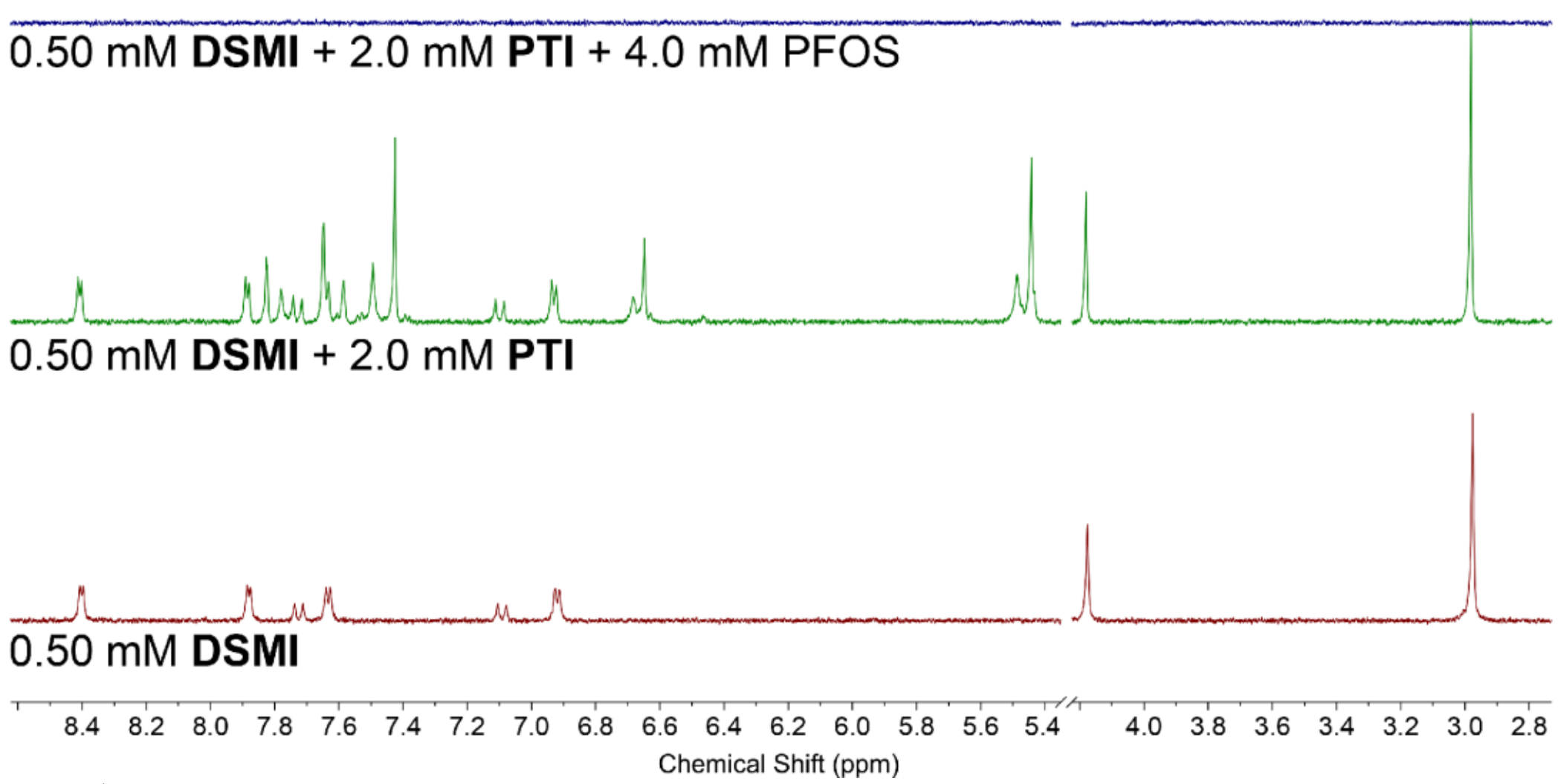

Figure S-45. ^{1}H NMR spectral titration of **DSMI** (0.5 mM), **DSMI** + **PTI** (2 mM), and **DSMI** + **PTI** + **PFOS** (4 mM) D_2O (600 MHz, 298K).

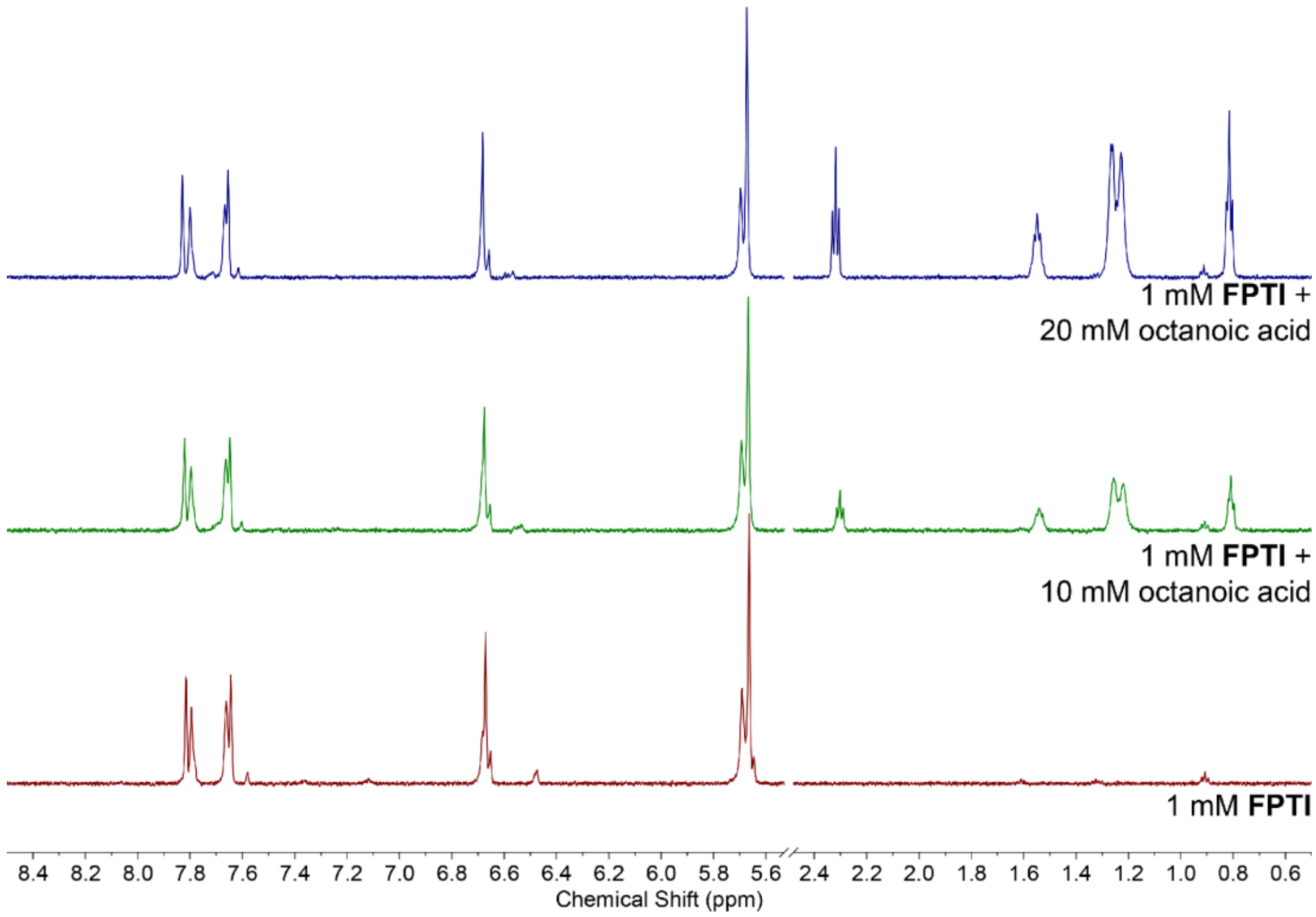

Figure S-46. ^{1}H NMR spectrum of **FPTI** (1 mM) and octanoic acid (0-20 mM) in D_2O (600 MHz, 298K). Only visible changes are shown; titrations referenced to the HOD peak at 4.79 ppm.

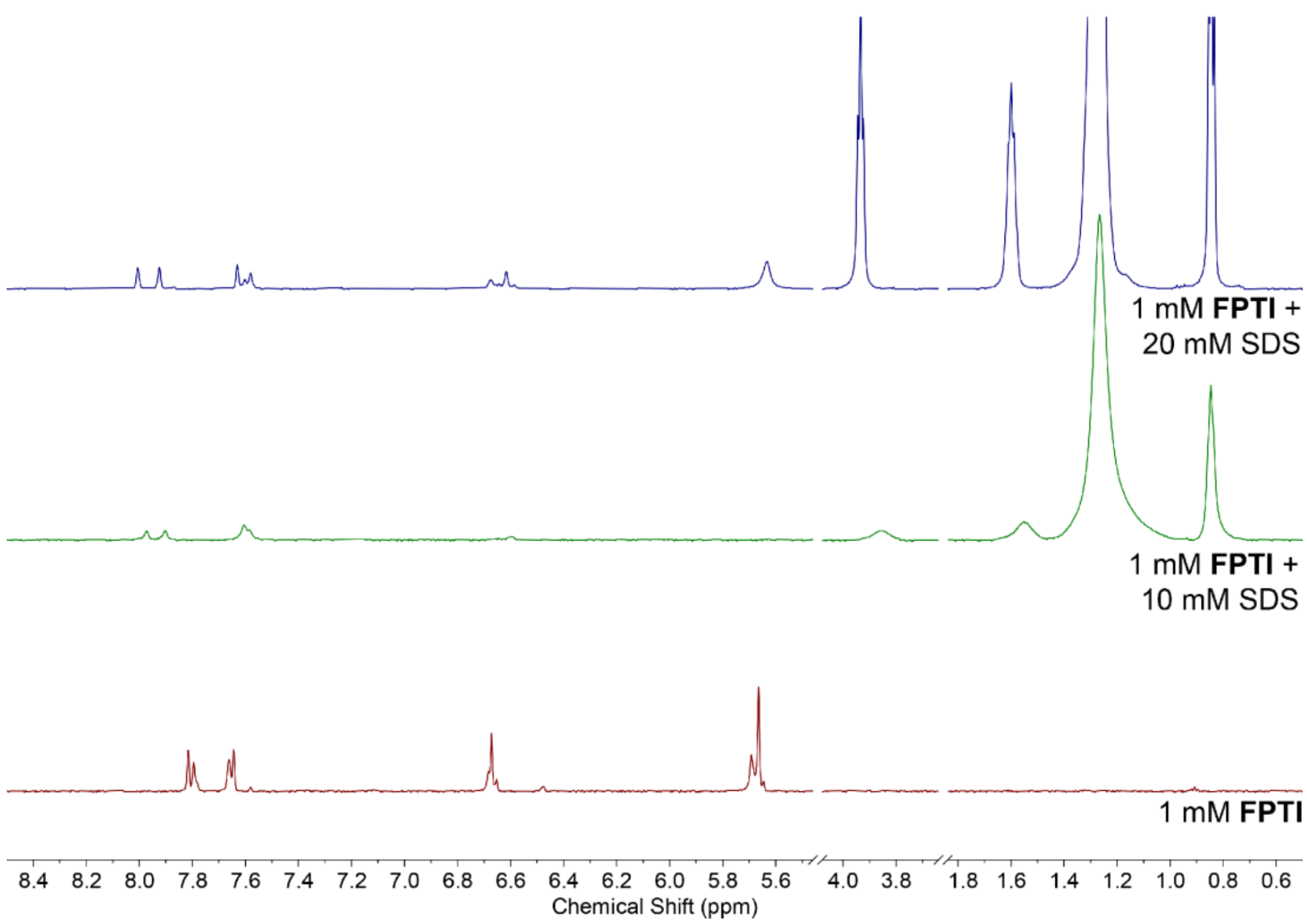

Figure S-47. ^{1}H NMR spectrum of **FPTI** (1 mM) and sodium dodecyl sulfate (SDS) (0-20 mM) in D_2O (600 MHz, 298K). Only visible changes are shown; titrations referenced to the HOD peak at 4.79 ppm.

a) Photos of **DSMI**, **DSMI/XTI**, **XTI**/PFOS, and **DSMI/XTI** + PFOS

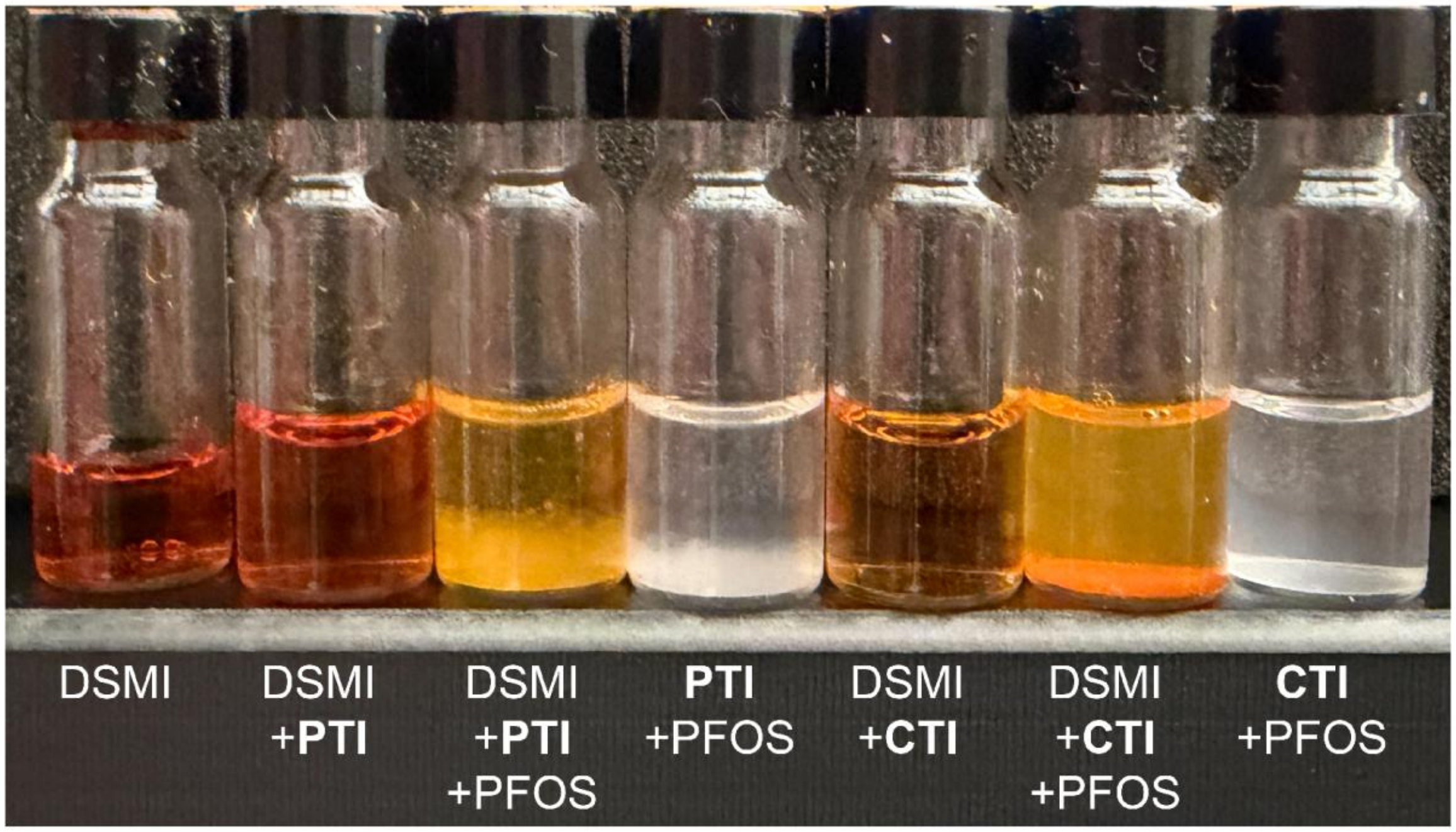

b) Photos of **DSMI**, **DSMI/XTI**, **XTI**/PFOS, and **DSMI/XTI** + PFOS under UV light

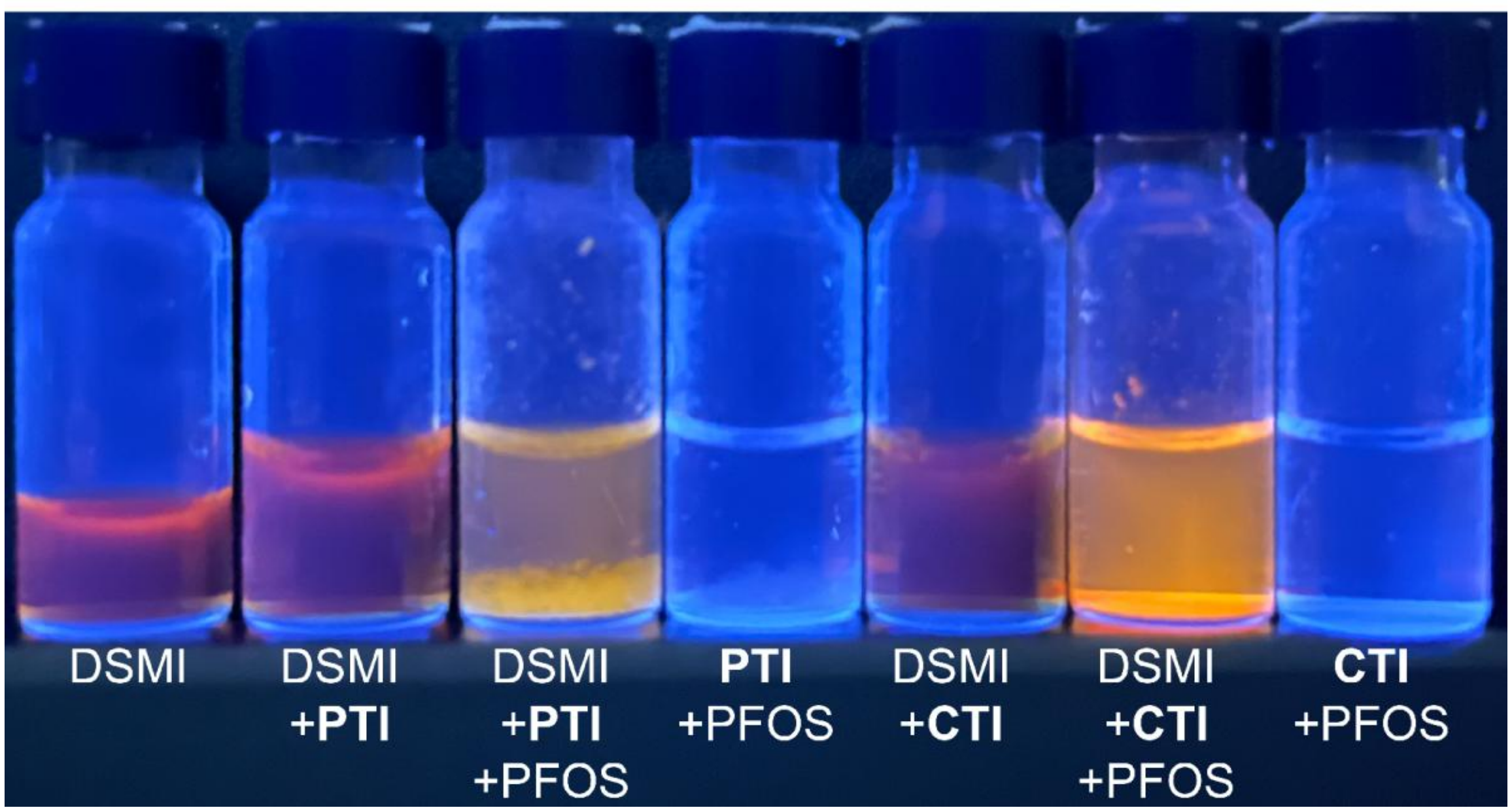

Figure S-48. Photos of **DSMI**, **XTI**, and PFOS mixtures under a) ambient light; b) UV light. [**DSMI**] = 0.5 μM, [**XTI**, **PTI** or **CTI**] = 4 μM, [PFOS] = 8 μM.

5. XTI Aggregate Characterization

5.1 Microscopy

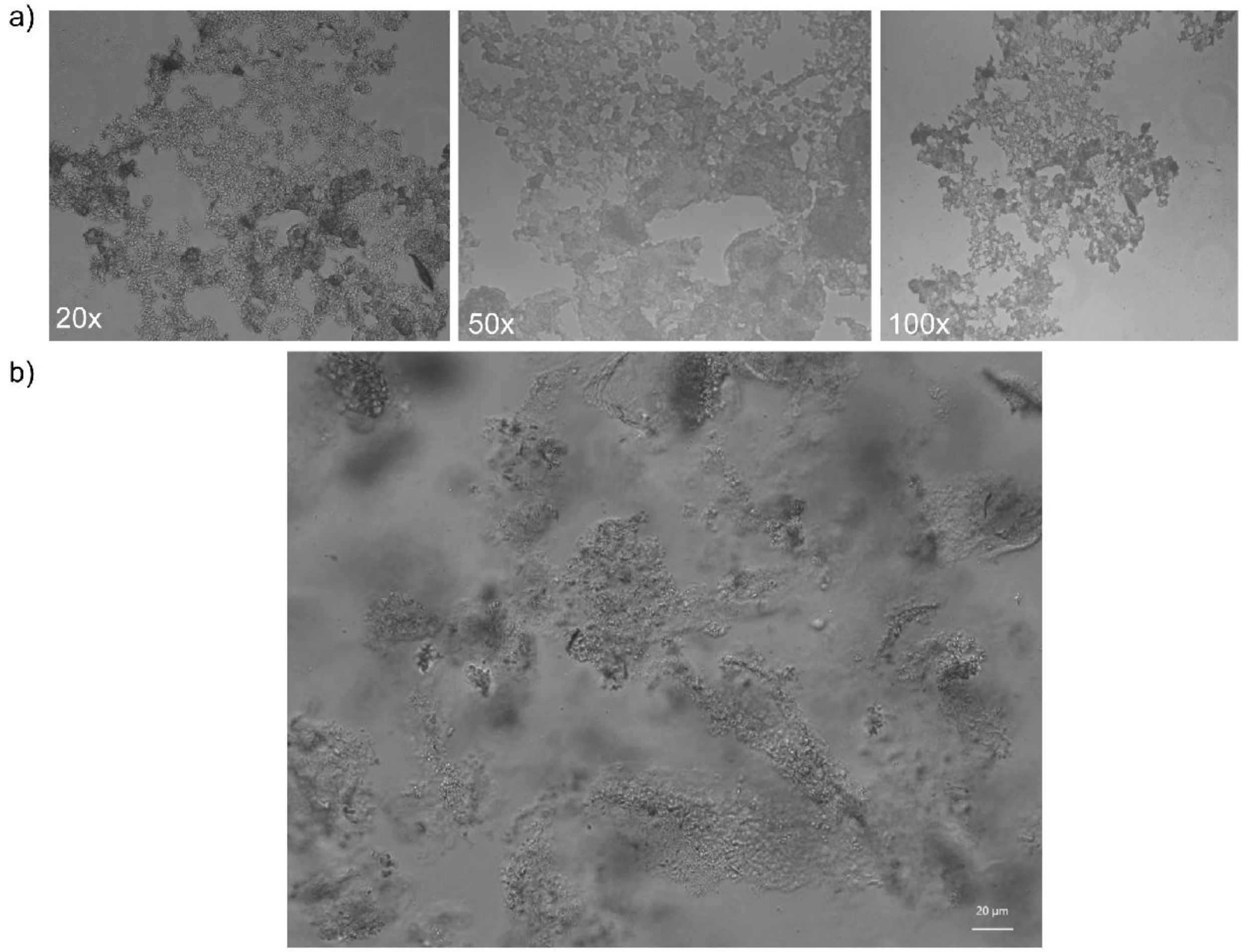

Figure S-49. Alternate views at various magnifications of the optical microscope images of the **PTI**•PFOS precipitate described in Figure 3e of the main text: a) acquired using BIM-800FLW microscope with 20x, 50x, and 100x objectives; b) acquired using a Keyence BZ-X Series all-in-one fluorescence microscope with a 40x objective.

5.2 Dynamic Light Scattering (DLS) Data

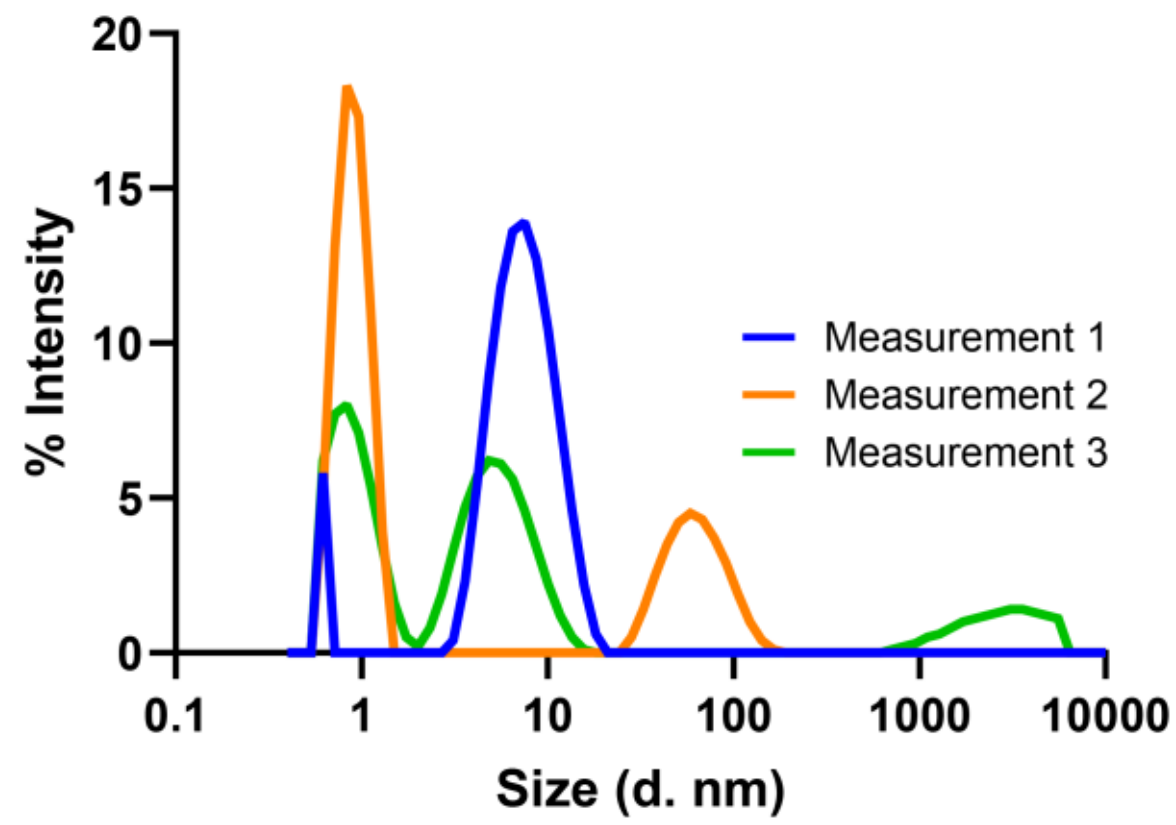

Figure S-50. Dynamic light scattering (DLS) size measurement of PFOS (0.4 mM) in 20 mM Tris-HCl buffer at pH 7.4 taken in 3 measurements.

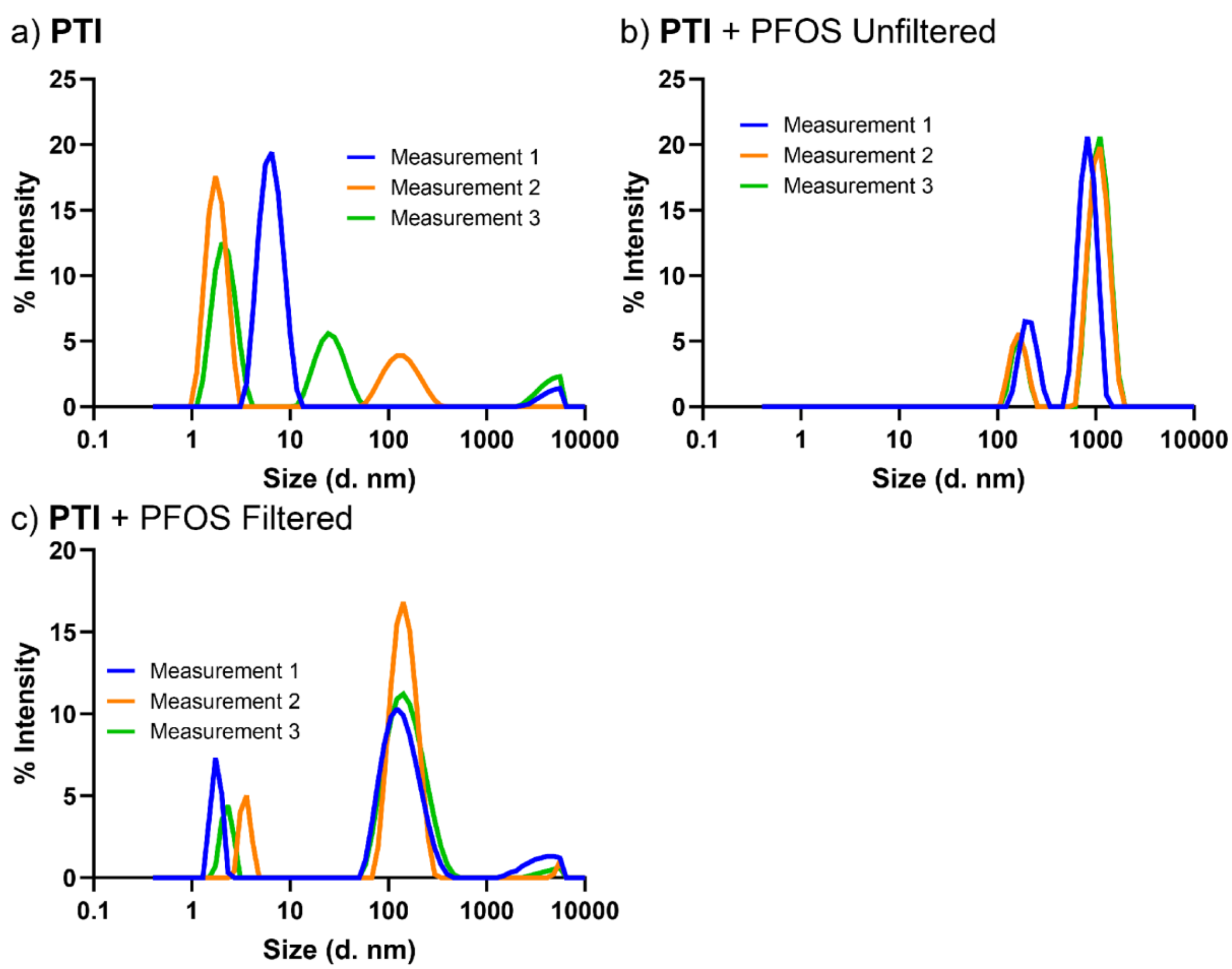

Figure S-51. Dynamic light scattering (DLS) size measurement of: a) **PTI** (0.1 mM); b) unfiltered solution of **PTI** (0.1 mM) and PFOS (0.4 mM); c) filtered solution of **PTI** (0.1 mM) and PFOS (0.4 mM).

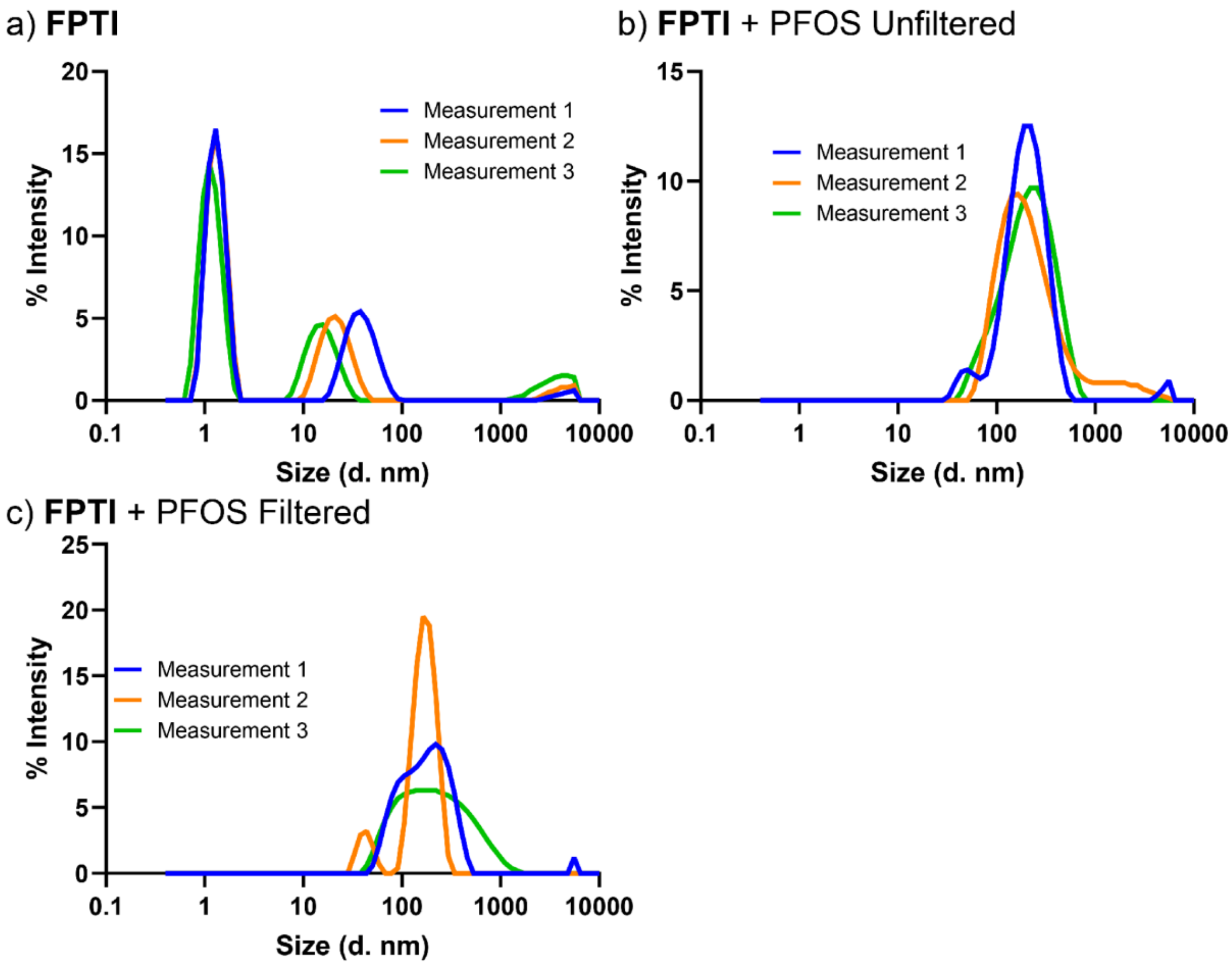

Figure S-52. Dynamic light scattering (DLS) size measurement of: a) **FPTI** (0.1 mM); b) unfiltered solution of **FPTI •Br** (0.1 mM) and PFOS (0.4 mM); c) filtered solution of **FPTI** (0.1 mM) and PFOS (0.4 mM).

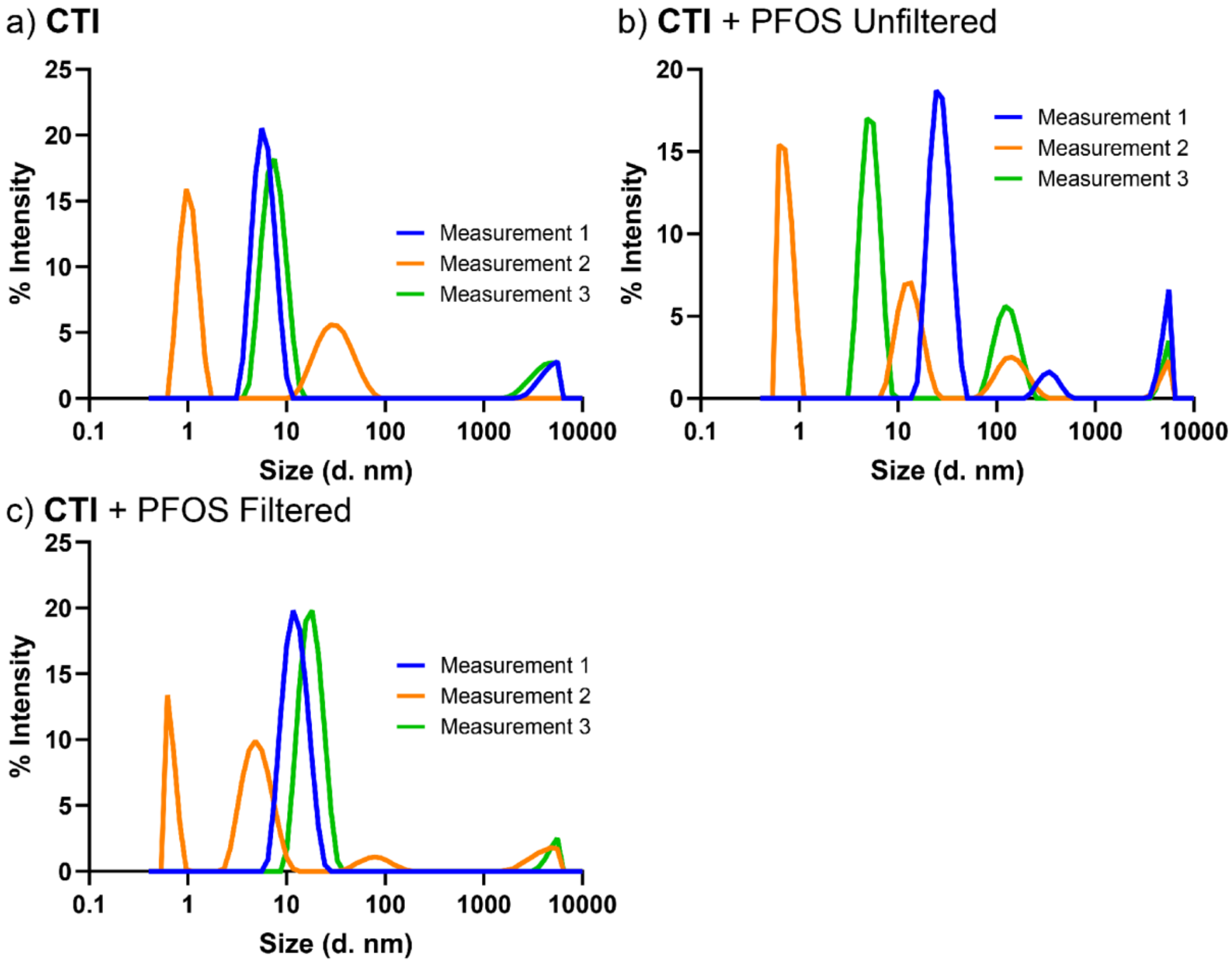

Figure S-53. Dynamic light scattering (DLS) size measurement of: a) **CTI** (0.1 mM); b) unfiltered solution of **CTI** (0.1 mM) and PFOS (0.4 mM); c) filtered solution of **CTI** (0.1 mM) and PFOS (0.4 mM).

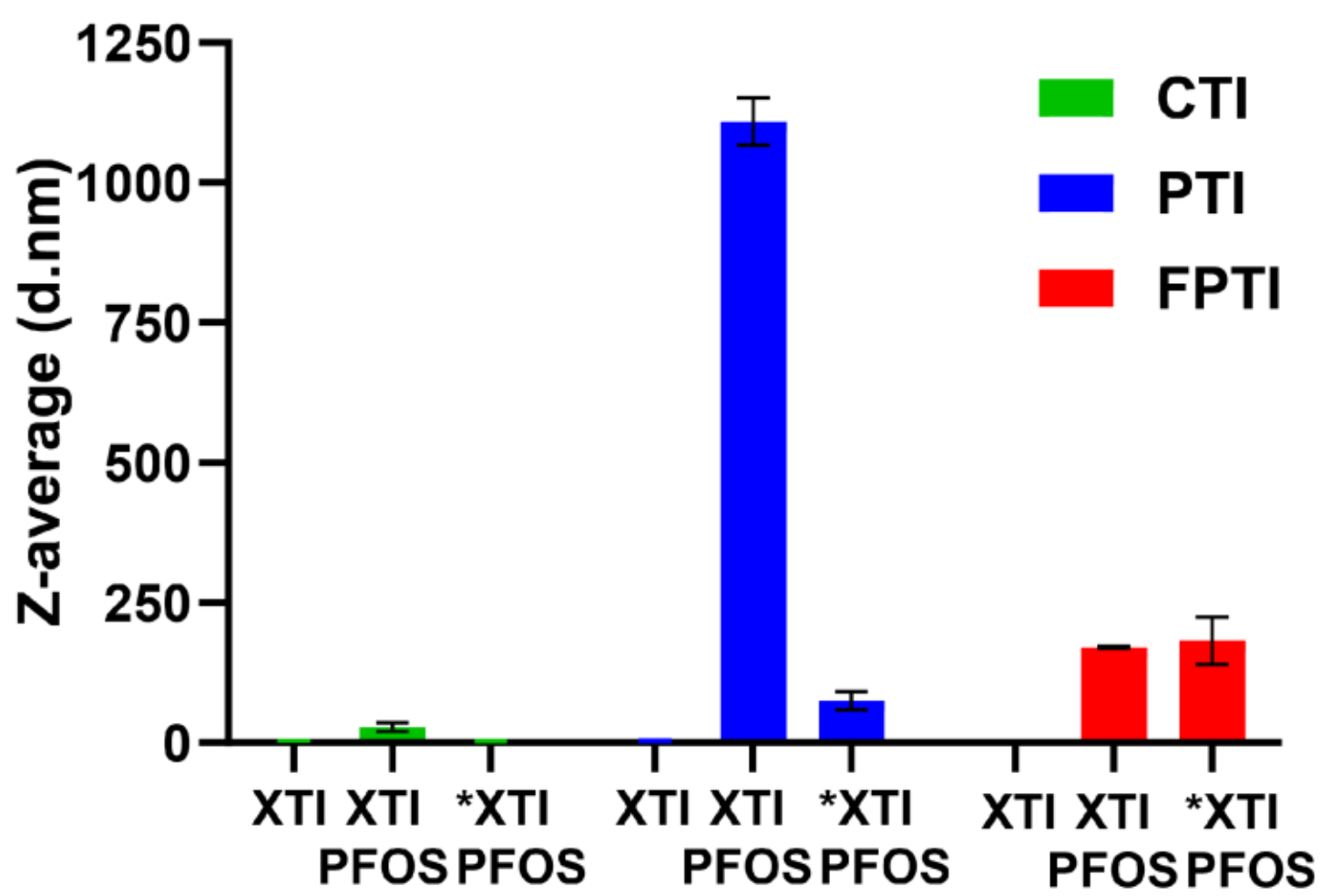

Figure S-54. Summarized DLS data. Average size of **XTI** (**CTI**, **PTI**, **FPTI**) (0.1 mM), **XTI** (0.1 mM) and PFOS (0.4 mM), and (*) filtered **XTI** and PFOS mixture.

Table S-3. Tabulated DLS Z-average data of receptor (**CTI**, **PTI**, and **FPTI**) aggregates with PFOS, from **Figures S-50 – S-54**.

		**CTI**			**PTI**			**FPTI**		
Sample	0.4 mM PFOS	0.1 mM **CTI**	1:4 **CTI**/PFOS	Filtered 1:4 **CTI**/PFOS	0.1 mM **PTI**	1:4 **PTI**/PFOS	Filtered 1:4 **PTI**/PFOS	0.1 mM **FPTI**	1:4 **FPTI**/PFOS	Filtered 1:4 **FPTI**/PFOS
Measurement 1	2.55	3.20	35.36	3.81	5.08	1089	56.54	2.69	172.50	176.90
Measurement 2	2.28	3.10	19.41	2.66	2.78	1158	86.88	2.66	169.60	227.10
Measurement 3	2.68	2.84	29.31	2.11	2.55	1081	82.17	2.75	168.90	143.10
Z-average (d. nm)	2.50	3.05	28.02	2.86	3.47	1109	75.19	2.70	170.33	182.37
SD	0.20	0.18	8.06	0.86	1.39	42.33	16.32	0.045	1.91	42.27

Table S-4. Tabulated DLS Polydispersity Index (PdI) data of receptor (**CTI**, **PTI**, and **FPTI**) aggregates with PFOS, from **Figures S-50 – S-54**.

		**CTI**			**PTI**			**FPTI**		
Sample	0.4 mM PFOS	0.1 mM **CTI**	1:4 **CTI**/PFOS	Filtered 1:4 **CTI**/PFOS	0.1 mM **PTI**	1:4 **PTI**/PFOS	Filtered 1:4 **PTI**/PFOS	0.1 mM **FPTI**	1:4 **FPTI**/PFOS	Filtered 1:4 **FPTI**/PFOS
Measurement 1	0.357	0.328	0.155	0.363	0.370	1	1	0.363	0.273	0.380
Measurement 2	0.331	0.332	0.146	0.361	0.343	0.898	0.554	0.327	0.293	0.491
Measurement 3	0.343	0.336	0.154	0.260	0.328	0.802	0.693	0.353	0.266	0.451
PdI average	0.344	0.332	0.152	0.328	0.347	0.900	0.749	0.348	0.277	0.441
SD	0.013	0.004	0.005	0.059	0.021	0.099	0.228	0.019	0.014	0.056

6. PFOS Removal Studies

6.1 LC-MS Data

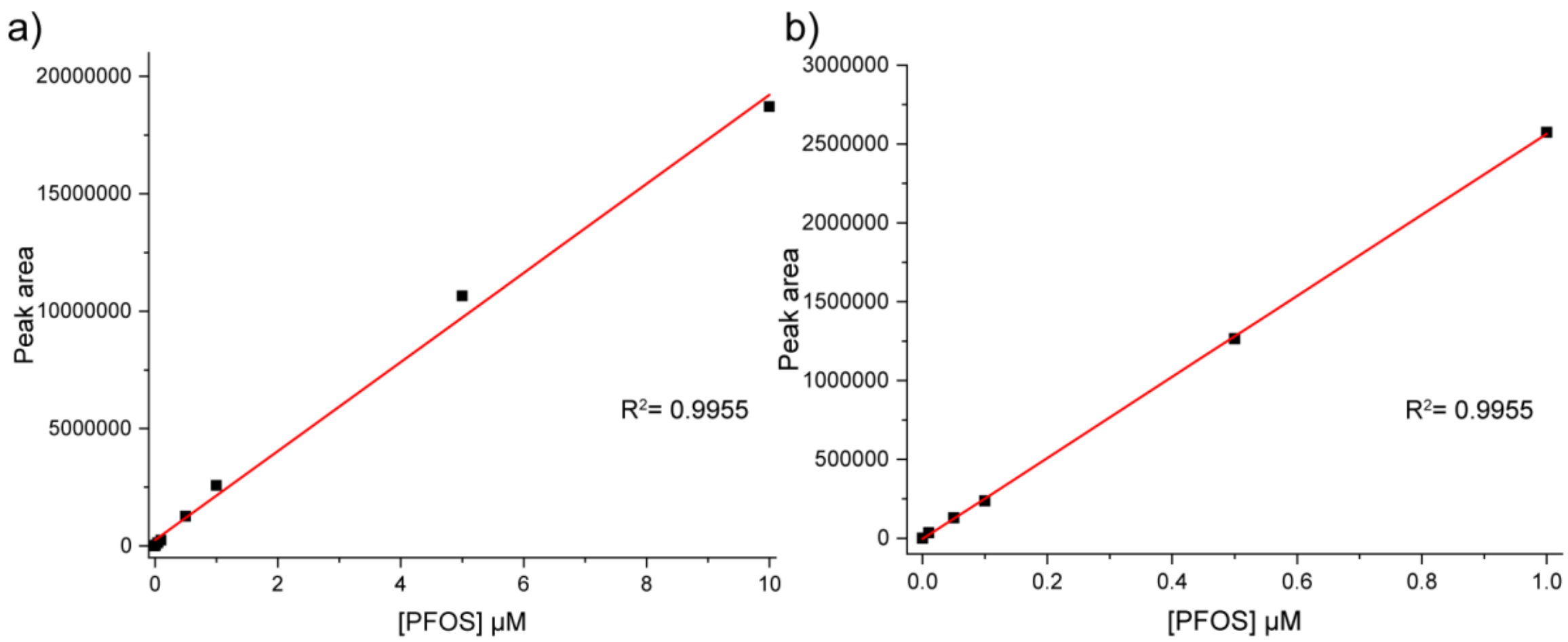

Figure S-55. LC-MS calibration curve of PFOS with concentration ranges of a) 0.01-10 µM PFOS b) 0.01-1.0 µM PFOS. Retention time = 10.3 minutes.

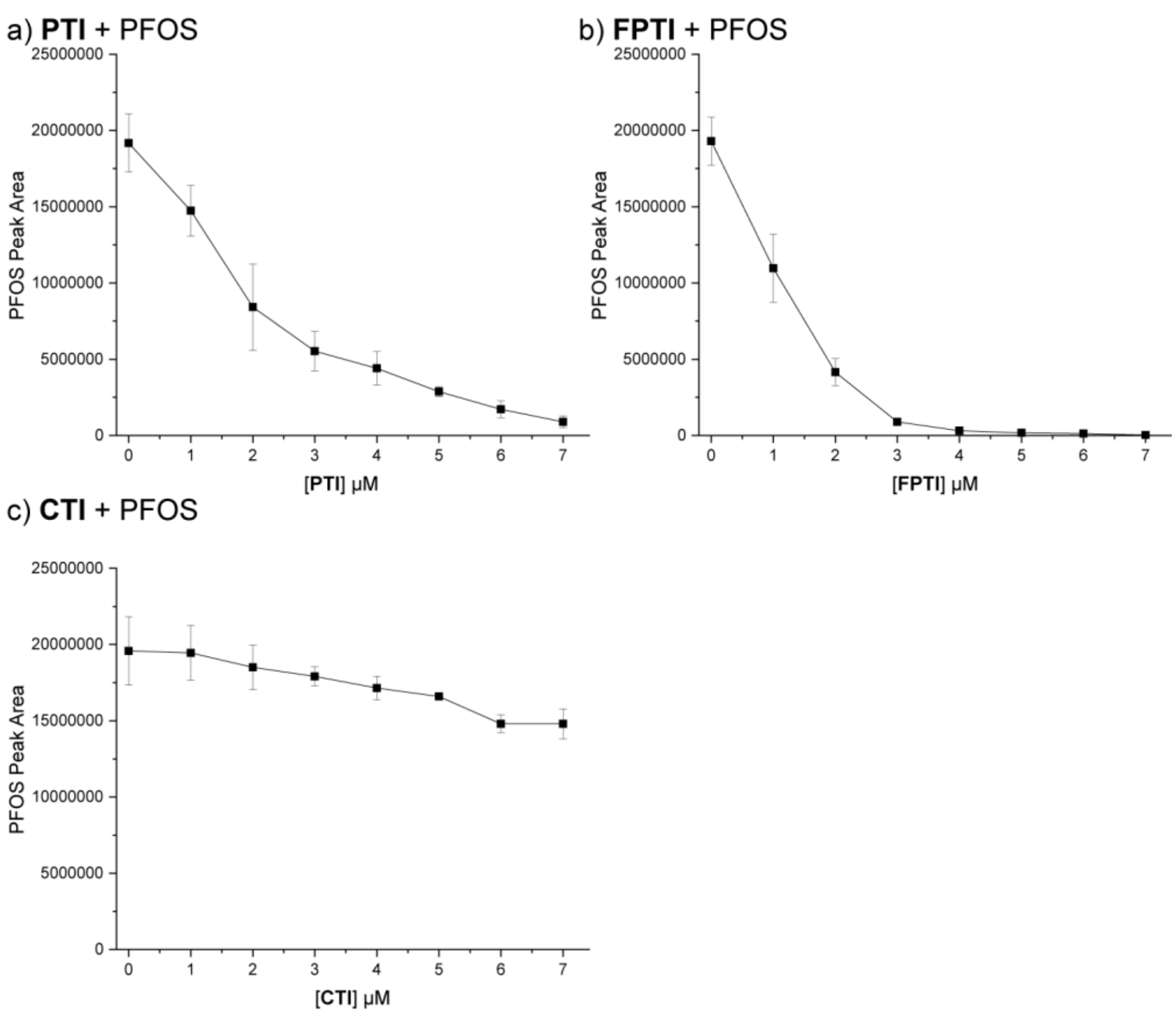

Figure S-56. LC-MS analysis of 10 µM PFOS (5000 ppb) in water with increasing concentration of **XTI** (0-7 µM) added to a) **PTI**; b) **FPTI**; c) **CTI**. The **XTI** + PFOS mixtures were filtered through a 0.22 µm methylcellulose (MCE) membrane prior to measurement.

Table S-5. Tabulated LC-MS average peak area for each receptor of remaining PFOS.

[Receptor, µM]	PTI	FPTI	CTI
0	19,174,966±1,904,464	19,295,200±1,583,777	19,584,966±2,235,048
1	14,747,900±1,670,929	10,966,526±2,243,413	19,453,066±1,793,393
2	8,419,066±2,830,606	4,148,020±885,711	18,501,733±1,460,406
3	5,536,256±1,291,904	887,367±161,344	17,915,533±627,574
4	4,405,733±1,083,214	312,376±228,945	17,148,033±776,413
5	2,868,143±310,547	167,809±124,589	16,596,666±101,883
6	1,709,070±568,570	122,498±97,411	14,806,100±586,211
7	894,257±379,141	21,237±3,750	14,801,033±990,769

Table S-6. Tabulated percentage of PFOS remaining by each receptor.

[Receptor, µM]	PTI	FPTI	CTI
0	100±9.93	100±8.21	100±11.41
1	76.91±8.71	56.83±11.63	99.33±9.16
2	43.91±14.76	21.50±4.59	94.47±7.46
3	28.87±6.74	4.60±0.84	91.48±3.20
4	22.98±5.65	1.62±1.19	87.56±3.96
5	14.96±1.62	0.87±0.65	84.74±0.52
6	8.91±2.96	0.63±0.50	75.60±2.99
7	4.66±1.98	0.11±0.019	75.57±5.06

7. Computational Details

7.1 Molecular Models

As a first step, the geometries of the **PTI**, **FPTI**, and **CTI** receptors were optimized (Figure S-57) using Kohn-Sham density functional theory (DFT)[12] with the M06−2X exchange correlation functional[13] and def2-TZVP basis set[14] and embedded in a continuum aqueous solvent using C-PCM.[15] The **PTI** and **FPTI** receptors adopt a characteristic 'book-like' conformation exhibiting C_s symmetry, where the molecular folding is localized at the methylene (CH_2) linkages connecting the imidazole moieties. In contrast, the **CTI** receptor exhibits a twisted geometry with the carboxylate groups pointing inward to the central macrocyclic core.

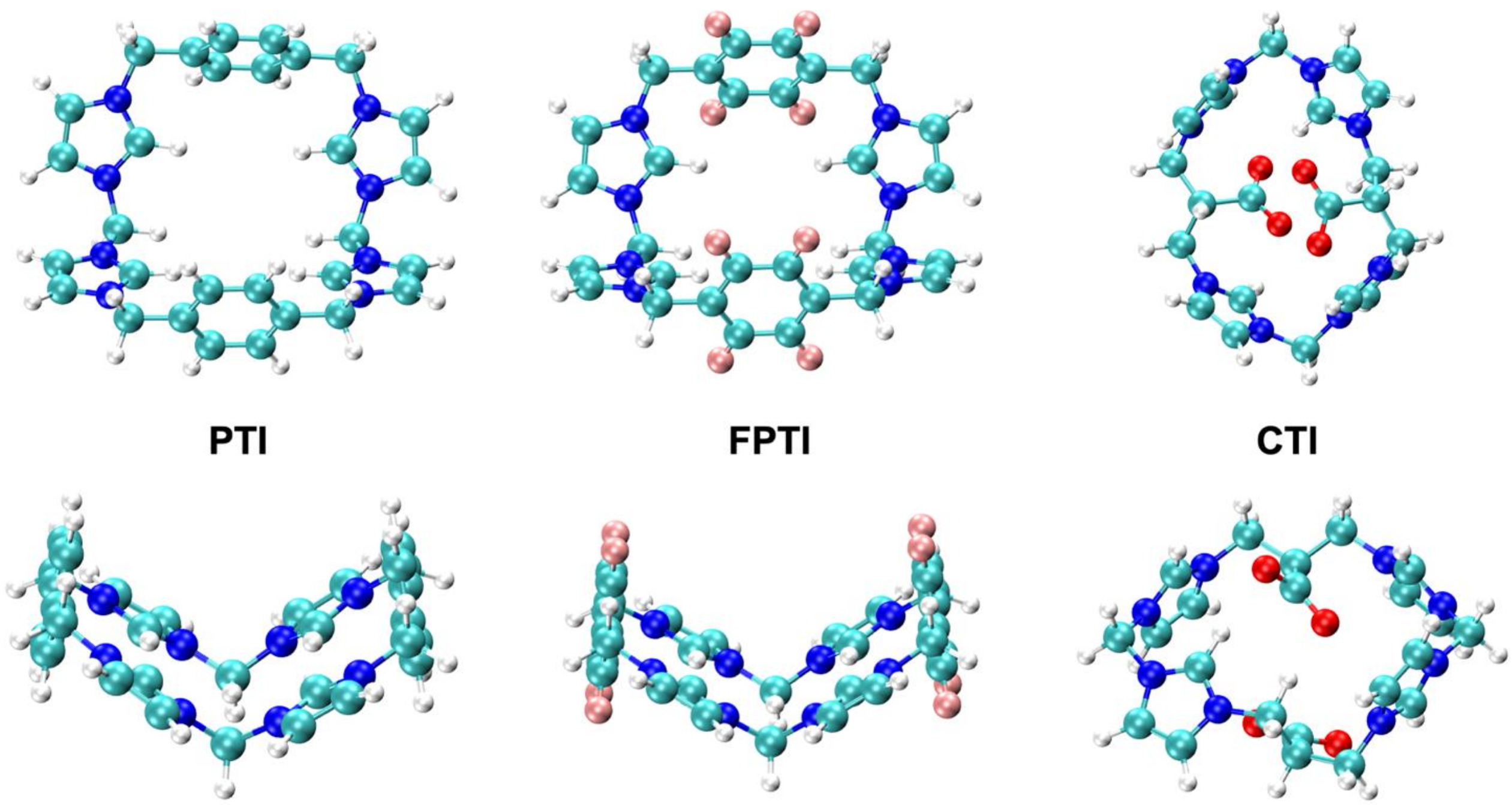

Figure S-57. Optimized structures of **PTI** (left), **FPTI** (middle), and **CTI** (right) viewed from two orientations.

The optimized structures were used to generate General Amber Force Field (GAFF) parameters.[16] Initial coordinates were processed through the Antechamber module of AmberTools,[17] with atom types assigned according to the GAFF2 classification system. To ensure force field completeness, the parmchk2 utility[18] was used to identify and supplement any missing valence parameters (bond, angle, or dihedral terms). System topologies and coordinate files were subsequently generated using TLeap[18] and the ParmEd library,[19] which defined the bonded and non-bonded interactions, including 1-4 and 1-5 pair-type terms. To accurately represent the charge distribution of the receptors, partial atomic charges were derived using the Charge Model 5 (CM5)[20] in octanol continuum solvent[15] (which reflects an intermediate medium) and

mapped onto the corresponding atoms. To observe the effect of polarization due to the presence of an anion, we performed calculations for both the bare receptor cation and its complex with a single chloride anion. For **PTI** and **FPTI**, the chloride anion sits in the central location of the symmetric receptor structures, but sits somewhat off-center for **CTI** (Figures S-58 to S-60). The CM5 charge model allows for charge transfer from between macrocycle and anion in the complex resulting in partial charges between −0.727 and −0.809 |e| for the chloride anion. To yield a macrocycle with integer charge, the partial charges of the imidazolium H(N) atoms were adjusted to yield a charge of +4 |e| for **PTI** and **FPTI** and +2 |e| for **CTI**. Partial charges for the receptor obtained for bare receptor and adjusted receptor−chloride complex were then averaged for each atom. As the last step, partial charges for atoms in equivalent formula positions (e.g., the carbon atoms bonded to two nitrogen atoms in each of the four imidazole rings) were averaged and rounded to four significant figures, but constrained to yield integer charge. Tables S-7 to S-9 list the partial charges for **PTI**, **FPTI**, and **CTI** encountered during this multi-step procedure. Analysis of the molecular electrostatic potential of the three **XTI** polycations shows that the charge deficiency (i.e., repulsive interaction for a positive probe charge) is delocalized fairly well over the entire macrocycles (Figure S-58). The **DSMI** and **PSMI** dye molecules were parametrized using the protocol described above.

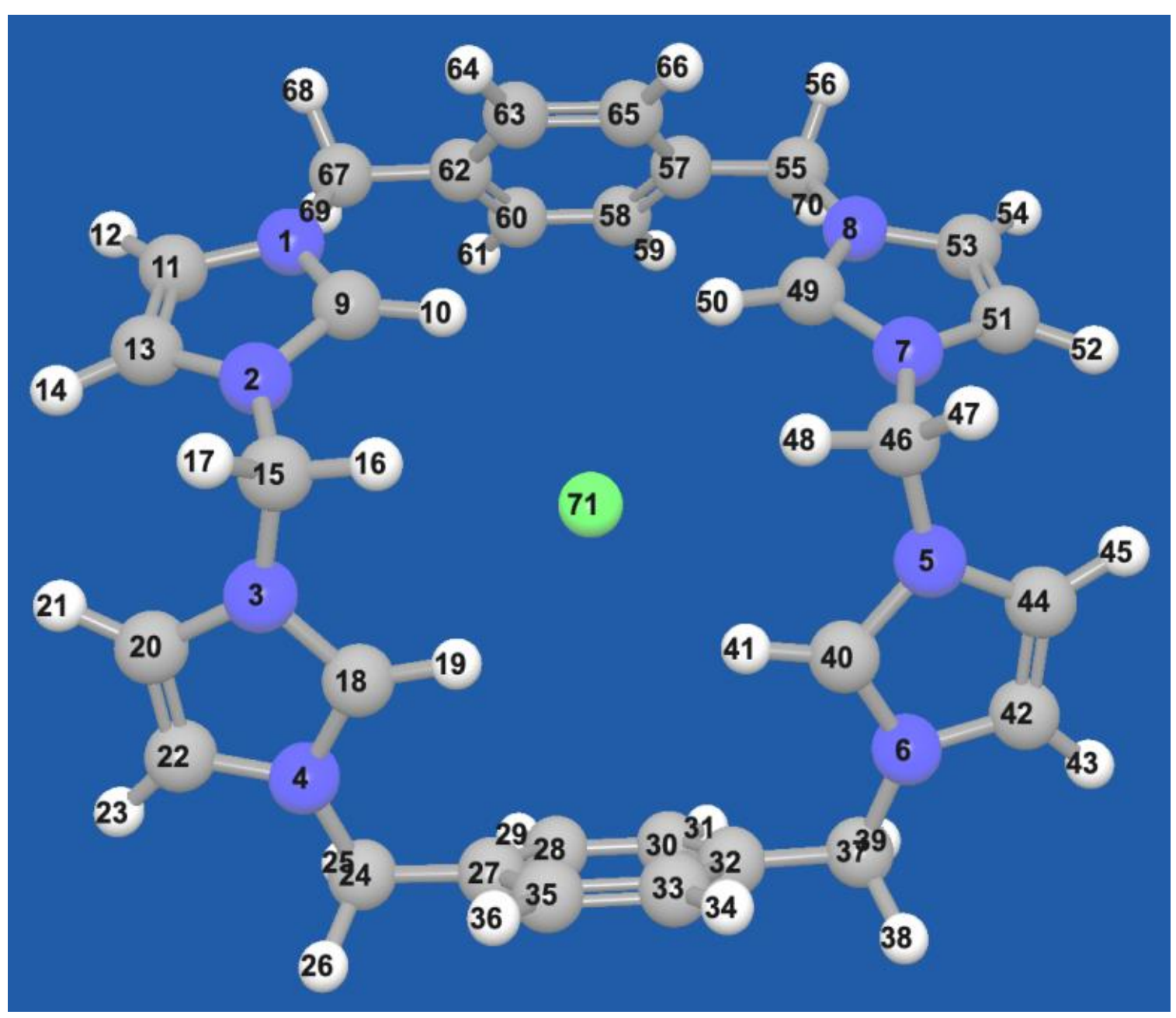

Figure S-58. Calculated structure of the **PTI**-Cl complex with atom indices. The same atom indices are used for the bare **PTI** macrocycle.

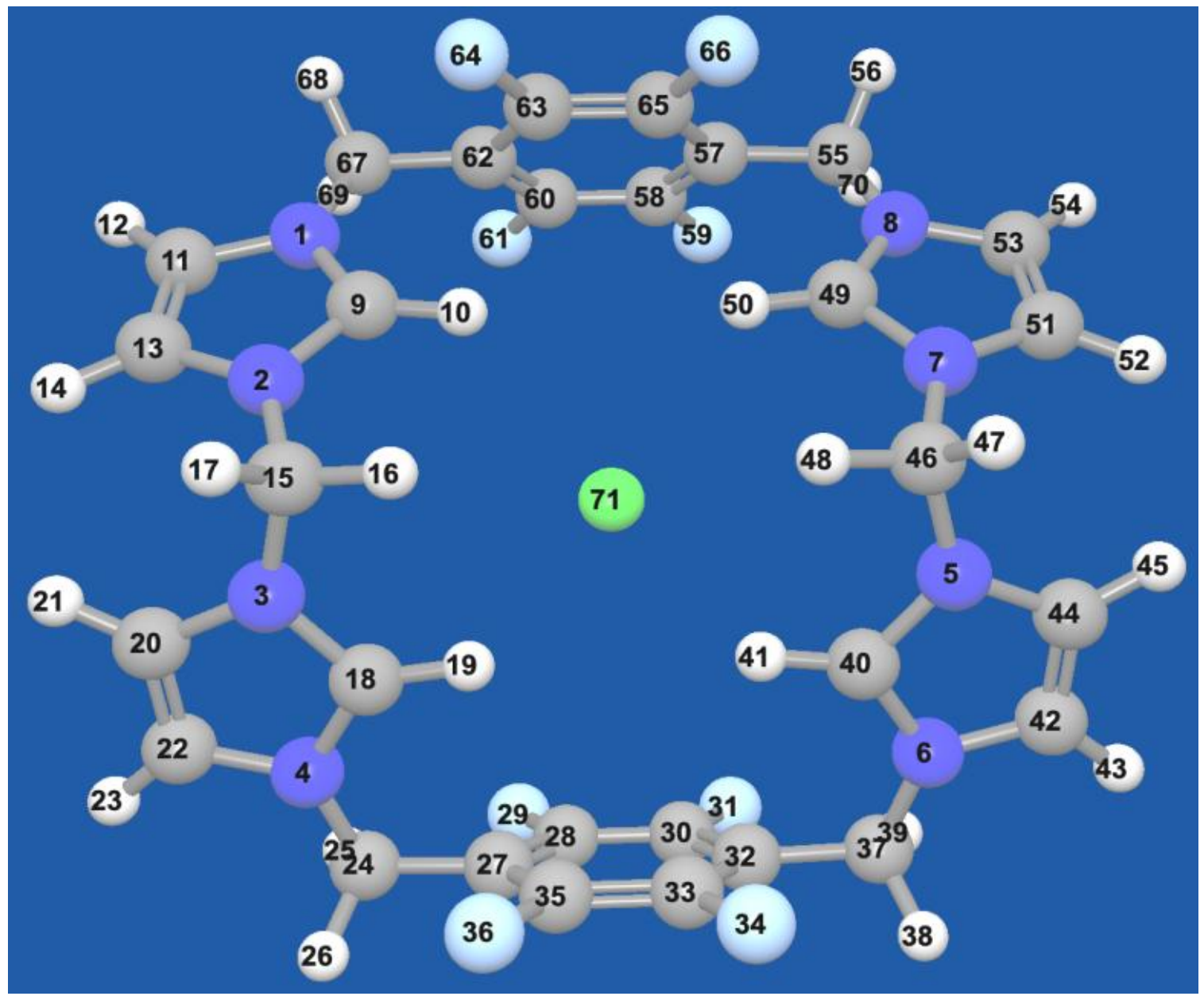

Figure S-59. Calculated structure of the **FPTI**-Cl complex with atom indices. The same atom indices are used for the bare **FPTI** macrocycle.

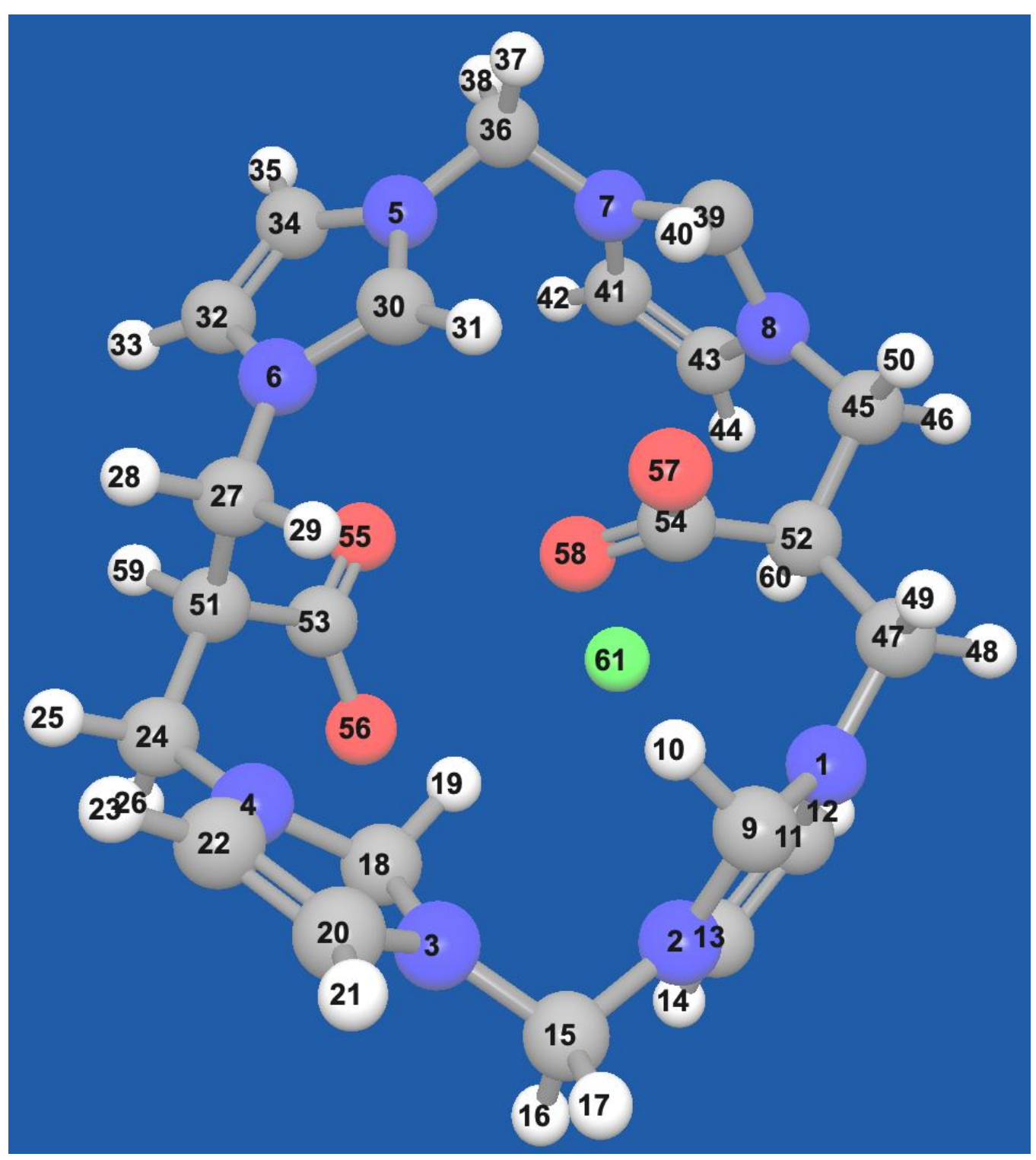

Figure S-60. Calculated structure of the **CTI**-Cl complex with atom indices. The same atom indices are used for the bare **CTI** macrocycle.

Table S7. Partial charges encountered during the multi-step process as used in this work to obtain partial charges for the **PTI** macrocycle.

Index	Atom	Raw CM5 charges		Difference	ImidH	Average	Equivalent
		bare PTI	PTI-Cl	bare − complex	adjusted	0.5*(bare PTI + ImidH-adj)	position average
1	N	-0.211643	-0.193076	-0.018567	-0.193076	-0.2023595	-0.2024
2	N	-0.227746	-0.21191	-0.015836	-0.21191	-0.219828	-0.2198
3	N	-0.22708	-0.212258	-0.014822	-0.212258	-0.219669	-0.2198
4	N	-0.211596	-0.193415	-0.018181	-0.193415	-0.2025055	-0.2024
5	N	-0.227769	-0.211914	-0.015855	-0.211914	-0.2198415	-0.2198
6	N	-0.211549	-0.193077	-0.018472	-0.193077	-0.202313	-0.2024
7	N	-0.227166	-0.212265	-0.014901	-0.212265	-0.2197155	-0.2198
8	N	-0.211597	-0.193426	-0.018171	-0.193426	-0.2025115	-0.2024
9	C	0.210376	0.195847	0.014529	0.195847	0.2031115	0.2029
**10**	**H**	**0.166838**	**0.157419**	0.009419	**0.1801895**	**0.17351375**	0.1735
11	C	0.064028	0.051535	0.012493	0.051535	0.0577815	0.0576
**12**	**H**	**0.171478**	**0.17039**	0.001088	**0.1931605**	**0.18231925**	0.1822
13	C	0.052007	0.040867	0.01114	0.040867	0.046437	0.0460
**14**	**H**	**0.165455**	**0.164018**	0.001437	**0.1867885**	**0.17612175**	0.1755
15	C	0.139505	0.12053	0.018975	0.12053	0.1300175	0.1300
16	H	0.173458	0.164713	0.008745	0.164713	0.1690855	0.1715
17	H	0.179228	0.168659	0.010569	0.168659	0.1739435	0.1715
18	C	0.209784	0.195521	0.014263	0.195521	0.2026525	0.2029
**19**	**H**	**0.166498**	**0.157463**	0.009035	**0.1802335**	**0.17336575**	0.1735
20	C	0.051454	0.039682	0.011772	0.039682	0.045568	0.0460
**21**	**H**	**0.163928**	**0.162915**	0.001013	**0.1856855**	**0.17480675**	0.1755
22	C	0.063658	0.051009	0.012649	0.051009	0.0573335	0.0576
**23**	**H**	**0.1714**	**0.17016**	0.00124	**0.1929305**	**0.18216525**	0.1822
24	C	0.003463	-0.004407	0.00787	-0.004407	-0.000472	-0.0005
25	H	0.142244	0.13894	0.003304	0.13894	0.140592	0.1405
26	H	0.144938	0.1382	0.006738	0.1382	0.141569	0.1416
27	C	-0.010304	-0.012111	0.001807	-0.012111	-0.0112075	-0.0114
28	C	-0.076607	-0.079503	0.002896	-0.079503	-0.078055	-0.0779
29	H	0.122665	0.120321	0.002344	0.120321	0.121493	0.1215
30	C	-0.076195	-0.079435	0.00324	-0.079435	-0.077815	-0.0779
31	H	0.122696	0.12032	0.002376	0.12032	0.121508	0.1215
32	C	-0.010634	-0.012365	0.001731	-0.012365	-0.0114995	-0.0114
33	C	-0.081766	-0.085908	0.004142	-0.085908	-0.083837	-0.0839
34	H	0.120562	0.116311	0.004251	0.116311	0.1184365	0.1182

35	C	-0.082203	-0.085942	0.003739	-0.085942	-0.0840725	-0.0839
36	H	0.119903	0.116067	0.003836	0.116067	0.117985	0.1182
37	C	0.003433	-0.0044	0.007833	-0.0044	-0.0004835	-0.0005
38	H	0.144918	0.13822	0.006698	0.13822	0.141569	0.1416
39	H	0.141744	0.138999	0.002745	0.138999	0.1403715	0.1405
40	C	0.210393	0.195909	0.014484	0.195909	0.203151	0.2029
**41**	**H**	**0.166821**	**0.157448**	0.009373	**0.1802185**	**0.17351975**	0.1735
42	C	0.064066	0.051515	0.012551	0.051515	0.0577905	0.0576
**43**	**H**	**0.171548**	**0.170386**	0.001162	**0.1931565**	**0.18235225**	0.1822
44	C	0.051757	0.040837	0.01092	0.040837	0.046297	0.0460
**45**	**H**	**0.165362**	**0.164011**	0.001351	**0.1867815**	**0.17607175**	0.1755
46	C	0.139519	0.12054	0.018979	0.12054	0.1300295	0.1300
47	H	0.179268	0.168662	0.010606	0.168662	0.173965	0.1715
48	H	0.173384	0.164707	0.008677	0.164707	0.1690455	0.1715
49	C	0.209738	0.195554	0.014184	0.195554	0.202646	0.2029
**50**	**H**	**0.166601**	**0.157449**	0.009152	**0.1802195**	**0.17341025**	0.1735
51	C	0.051392	0.039673	0.011719	0.039673	0.0455325	0.0460
**52**	**H**	**0.16412**	**0.16292**	0.0012	**0.1856905**	**0.17490525**	0.1755
53	C	0.063735	0.051003	0.012732	0.051003	0.057369	0.0576
**54**	**H**	**0.171346**	**0.17016**	0.001186	**0.1929305**	**0.18213825**	0.1822
55	C	0.003509	-0.004407	0.007916	-0.004407	-0.000449	-0.0005
56	H	0.144854	0.138198	0.006656	0.138198	0.141526	0.1416
57	C	-0.010375	-0.01212	0.001745	-0.01212	-0.0112475	-0.0114
58	C	-0.076524	-0.079517	0.002993	-0.079517	-0.0780205	-0.0779
59	H	0.12251	0.120308	0.002202	0.120308	0.121409	0.1215
60	C	-0.0763	-0.079449	0.003149	-0.079449	-0.0778745	-0.0779
61	H	0.122621	0.120306	0.002315	0.120306	0.1214635	0.1215
62	C	-0.010555	-0.012376	0.001821	-0.012376	-0.0114655	-0.0114
63	C	-0.081678	-0.085905	0.004227	-0.085905	-0.0837915	-0.0839
64	H	0.120689	0.116309	0.00438	0.116309	0.118499	0.1182
65	C	-0.082089	-0.085942	0.003853	-0.085942	-0.0840155	-0.0839
66	H	0.120078	0.11607	0.004008	0.11607	0.118074	0.1182
67	C	0.003506	-0.004406	0.007912	-0.004406	-0.00045	-0.0005
68	H	0.144953	0.138215	0.006738	0.138215	0.141584	0.1416
69	H	0.141689	0.138999	0.00269	0.138999	0.140344	0.1405
70	H	0.142193	0.13894	0.003253	0.13894	0.1405665	0.1405
71	Cl	-	-0.726786				
**Total charge**		3.999937	3.726691	0.273246	3.999937	3.999937	4.0000

Table S8. Partial charges encountered during the multi-step process as used in this work to obtain partial charges for the **FPTI** macrocycle.

Index	Atom	Raw CM5 charges		Difference	ImidH	Average	Equivalent position average
		bare FPTI	FPTI-Cl	bare − complex	adjusted	0.5*(bare FPTI + ImidH-adj)	
1	N	-0.213754	-0.194311	-0.019443	-0.194311	-0.2040325	-0.2041
2	N	-0.226572	-0.208325	-0.018247	-0.208325	-0.2174485	-0.2174
3	N	-0.226168	-0.208434	-0.017734	-0.208434	-0.217301	-0.2174
4	N	-0.213815	-0.194509	-0.019306	-0.194509	-0.204162	-0.2041
5	N	-0.226635	-0.208322	-0.018313	-0.208322	-0.2174785	-0.2174
6	N	-0.213714	-0.194309	-0.019405	-0.194309	-0.2040115	-0.2041
7	N	-0.226465	-0.208436	-0.018029	-0.208436	-0.2174505	-0.2174
8	N	-0.213937	-0.19451	-0.019427	-0.19451	-0.2042235	-0.2041
9	C	0.207646	0.194826	0.01282	0.194826	0.201236	0.2010
**10**	**H**	**0.165383**	**0.158741**	0.006642	**0.180648667**	**0.173015833**	0.1730
11	C	0.064211	0.056878	0.007333	0.056878	0.0605445	0.0605
**12**	**H**	**0.17167**	**0.173689**	-0.002019	**0.195596667**	**0.183633333**	0.1836
13	C	0.05207	0.045337	0.006733	0.045337	0.0487035	0.0486
**14**	**H**	**0.164721**	**0.166193**	-0.001472	**0.188100667**	**0.176410833**	0.1763
15	C	0.139418	0.12272	0.016698	0.12272	0.131069	0.1311
16	H	0.173544	0.166151	0.007393	0.166151	0.1698475	0.1726
17	H	0.178972	0.171724	0.007248	0.171724	0.175348	0.1726
18	C	0.206913	0.19464	0.012273	0.19464	0.2007765	0.2010
**19**	**H**	**0.165592**	**0.158593**	0.006999	**0.180500667**	**0.173046333**	0.1730
20	C	0.051847	0.045014	0.006833	0.045014	0.0484305	0.0486
**21**	**H**	**0.164452**	**0.165864**	-0.001412	**0.187771667**	**0.176111833**	0.1763
22	C	0.064184	0.056684	0.0075	0.056684	0.060434	0.0605
**23**	**H**	**0.171625**	**0.173599**	-0.001974	**0.195506667**	**0.183565833**	0.1836
24	C	-0.001112	0.003537	-0.004649	0.003537	0.0012125	0.0012
25	H	0.141805	0.14536	-0.003555	0.14536	0.1435825	0.1436
26	H	0.143865	0.148406	-0.004541	0.148406	0.1461355	0.1461
27	C	-0.070995	-0.007698	-0.063297	-0.007698	-0.0393465	-0.0394
28	C	0.112002	0.125541	-0.013539	0.125541	0.1187715	0.1188
29	F	-0.026302	-0.10692	0.080618	-0.10692	-0.066611	-0.0666
30	C	0.11209	0.125648	-0.013558	0.125648	0.118869	0.1188
31	F	-0.026226	-0.106863	0.080637	-0.106863	-0.0665445	-0.0666
32	C	-0.071144	-0.007844	-0.0633	-0.007844	-0.039494	-0.0394
33	C	0.1021	0.115883	-0.013783	0.115883	0.1089915	0.1090
34	F	-0.03208	-0.112408	0.080328	-0.112408	-0.072244	-0.0722

35	C	0.101905	0.115885	-0.01398	0.115885	0.108895	0.1090
36	F	-0.032182	-0.112338	0.080156	-0.112338	-0.07226	-0.0722
37	C	-0.001116	0.003541	-0.004657	0.003541	0.0012125	0.0012
38	H	0.143745	0.148352	-0.004607	0.148352	0.1460485	0.1461
39	H	0.14173	0.145412	-0.003682	0.145412	0.143571	0.1436
40	C	0.207389	0.194832	0.012557	0.194832	0.2011105	0.2010
**41**	**H**	**0.165319**	**0.158758**	0.006561	**0.180665667**	**0.172992333**	0.1730
42	C	0.064266	0.05688	0.007386	0.05688	0.060573	0.0605
**43**	**H**	**0.171662**	**0.17369**	-0.002028	**0.195597667**	**0.183629833**	0.1836
44	C	0.052235	0.045339	0.006896	0.045339	0.048787	0.0486
**45**	**H**	**0.164965**	**0.166194**	-0.001229	**0.188101667**	**0.176533333**	0.1763
46	C	0.139355	0.12272	0.016635	0.12272	0.1310375	0.1311
47	H	0.179003	0.171725	0.007278	0.171725	0.175364	0.1726
48	H	0.173371	0.166151	0.00722	0.166151	0.169761	0.1726
49	C	0.206986	0.194639	0.012347	0.194639	0.2008125	0.2010
**50**	**H**	**0.165553**	**0.158578**	0.006975	**0.180485667**	**0.173019333**	0.1730
51	C	0.05207	0.045012	0.007058	0.045012	0.048541	0.0486
**52**	**H**	**0.164509**	**0.165863**	-0.001354	**0.187770667**	**0.176139833**	0.1763
53	C	0.064206	0.056682	0.007524	0.056682	0.060444	0.0605
**54**	**H**	**0.171614**	**0.173598**	-0.001984	**0.195505667**	**0.183559833**	0.1836
55	C	-0.001081	0.003536	-0.004617	0.003536	0.0012275	0.0012
56	H	0.143882	0.148404	-0.004522	0.148404	0.146143	0.1461
57	C	-0.0709	-0.007702	-0.063198	-0.007702	-0.039301	-0.0394
58	C	0.111929	0.125533	-0.013604	0.125533	0.118731	0.1188
59	F	-0.026361	-0.106924	0.080563	-0.106924	-0.0666425	-0.0666
60	C	0.111989	0.125639	-0.01365	0.125639	0.118814	0.1188
61	F	-0.026303	-0.106867	0.080564	-0.106867	-0.066585	-0.0666
62	C	-0.071238	-0.007847	-0.063391	-0.007847	-0.0395425	-0.0394
63	C	0.102227	0.115882	-0.013655	0.115882	0.1090545	0.1090
64	F	-0.03196	-0.112411	0.080451	-0.112411	-0.0721855	-0.0722
65	C	0.102102	0.115884	-0.013782	0.115884	0.108993	0.1090
66	F	-0.032123	-0.11234	0.080217	-0.11234	-0.0722315	-0.0722
67	C	-0.001128	0.003539	-0.004667	0.003539	0.0012055	0.0012
68	H	0.143791	0.14835	-0.004559	0.14835	0.1460705	0.1461
69	H	0.141589	0.14541	-0.003821	0.14541	0.1434995	0.1436
70	H	0.141798	0.145359	-0.003561	0.145359	0.1435785	0.1436
71	Cl	-	-0.737019				
**Total charge**		3.999989	3.737097	0.262892	3.999989	3.999989	4.0000

Table S9. Partial charges encountered during the multi-step process as sused in this work to obtain partial charges for the **CTI** macrocycle.

Index	Atom	Raw CM5 charges		Difference	ImidH	Average	Equivalent
		bare CTI	CTI-Cl	bare − complex	adjusted	0.5*(bare CTI + ImidH-adj)	position average
1	N	-0.280218	-0.194945	-0.085273	-0.194945	-0.2375815	-0.2231
2	N	-0.275373	-0.206609	-0.068764	-0.206609	-0.240991	-0.2316
3	N	-0.239308	-0.207733	-0.031575	-0.207733	-0.2235205	-0.2316
4	N	-0.237169	-0.188049	-0.04912	-0.188049	-0.212609	-0.2231
5	N	-0.257621	-0.215491	-0.04213	-0.215491	-0.236556	-0.2316
6	N	-0.264695	-0.192657	-0.072038	-0.192657	-0.228676	-0.2231
7	N	-0.240908	-0.209701	-0.031207	-0.209701	-0.2253045	-0.2316
8	N	-0.235352	-0.192049	-0.043303	-0.192049	-0.2137005	-0.2231
9	C	0.249216	0.134598	0.114618	0.134598	0.191907	0.1889
**10**	**H**	**0.170211**	**0.168314**	0.001897	**0.18427133**	**0.177241167**	0.1735
11	C	0.025954	0.065376	-0.039422	0.065376	0.045665	0.0461
**12**	**H**	**0.154413**	**0.147819**	0.006594	**0.16377633**	**0.159094667**	0.1645
13	C	0.013495	0.06414	-0.050645	0.06414	0.0388175	0.0444
**14**	**H**	**0.152881**	**0.164767**	-0.011886	**0.18072433**	**0.166802667**	0.1699
15	C	0.119469	0.118422	0.001047	0.118422	0.1189455	0.1206
16	H	0.161256	0.164871	-0.003615	0.164871	0.1630635	0.1631
17	H	0.165133	0.169512	-0.004379	0.169512	0.1673225	0.1673
18	C	0.214386	0.134338	0.080048	0.134338	0.174362	0.1889
**19**	**H**	**0.161805**	**0.156595**	0.00521	**0.17255233**	**0.167178667**	0.1735
20	C	0.041739	0.059806	-0.018067	0.059806	0.0507725	0.0444
**21**	**H**	**0.1612**	**0.168107**	-0.006907	**0.18406433**	**0.172632167**	0.1699
22	C	0.03936	0.056264	-0.016904	0.056264	0.047812	0.0461
**23**	**H**	**0.157989**	**0.166416**	-0.008427	**0.18237333**	**0.170181167**	0.1645
24	C	-0.016678	-0.014898	-0.00178	-0.014898	-0.015788	-0.0141
25	H	0.131696	0.125815	0.005881	0.125815	0.1287555	0.1295
26	H	0.133924	0.129463	0.004461	0.129463	0.1316935	0.1295
27	C	-0.020905	-0.017796	-0.003109	-0.017796	-0.0193505	-0.0153
28	H	0.130846	0.123403	0.007443	0.123403	0.1271245	0.1295
29	H	0.122112	0.116151	0.005961	0.116151	0.1191315	0.1295
30	C	0.229252	0.196767	0.032485	0.196767	0.2130095	0.1889
**31**	**H**	**0.156773**	**0.164986**	-0.008213	**0.18094333**	**0.168858167**	0.1735
32	C	0.026451	0.042745	-0.016294	0.042745	0.034598	0.0461
**33**	**H**	**0.154677**	**0.161556**	-0.006879	**0.17751333**	**0.166095167**	0.1645
34	C	0.024019	0.04049	-0.016471	0.04049	0.0322545	0.0444

**35**	**H**	**0.155037**	**0.163493**	-0.008456	**0.17945033**	**0.167243667**	0.1699
36	C	0.124026	0.120442	0.003584	0.120442	0.122234	0.1206
37	H	0.161294	0.163716	-0.002422	0.163716	0.162505	0.1625
38	H	0.169456	0.172527	-0.003071	0.172527	0.1709915	0.1710
39	C	0.222523	0.129901	0.092622	0.129901	0.176212	0.1889
**40**	**H**	**0.178832**	**0.166361**	0.012471	**0.18231833**	**0.180575167**	0.1735
41	C	0.043001	0.06847	-0.025469	0.06847	0.0557355	0.0444
**42**	**H**	**0.159628**	**0.17041**	-0.010782	**0.18636733**	**0.172997667**	0.1699
43	C	0.046173	0.066842	-0.020669	0.066842	0.0565075	0.0461
**44**	**H**	**0.159985**	**0.149037**	0.010948	**0.16499433**	**0.162489667**	0.1645
45	C	-0.012602	-0.010055	-0.002547	-0.010055	-0.0113285	-0.0153
46	H	0.133408	0.135306	-0.001898	0.135306	0.134357	0.1295
47	C	-0.016391	-0.008471	-0.00792	-0.008471	-0.012431	-0.0141
48	H	0.133765	0.134384	-0.000619	0.134384	0.1340745	0.1295
49	H	0.129772	0.129718	5.4E-05	0.129718	0.129745	0.1295
50	H	0.133474	0.129125	0.004349	0.129125	0.1312995	0.1295
51	C	-0.080977	-0.067587	-0.01339	-0.067587	-0.074282	-0.0761
52	C	-0.079024	-0.076919	-0.002105	-0.076919	-0.0779715	-0.0761
53	C	0.217812	0.172602	0.04521	0.172602	0.195207	0.1991
54	C	0.232212	0.173571	0.058641	0.173571	0.2028915	0.1991
55	O	-0.365514	-0.443935	0.078421	-0.443935	-0.4047245	-0.4194
56	O	-0.392066	-0.52279	0.130724	-0.52279	-0.457428	-0.4194
57	O	-0.377007	-0.448955	0.071948	-0.448955	-0.412981	-0.4194
58	O	-0.331006	-0.474131	0.143125	-0.474131	-0.4025685	-0.4194
59	H	0.115623	0.120279	-0.004656	0.120279	0.117951	0.1097
60	H	0.108463	0.094305	0.014158	0.094305	0.101384	0.1097
61	Cl		-0.808624				
**Total charge**		1.999927	1.808439	0.191488	1.999927	1.999927	2.0000

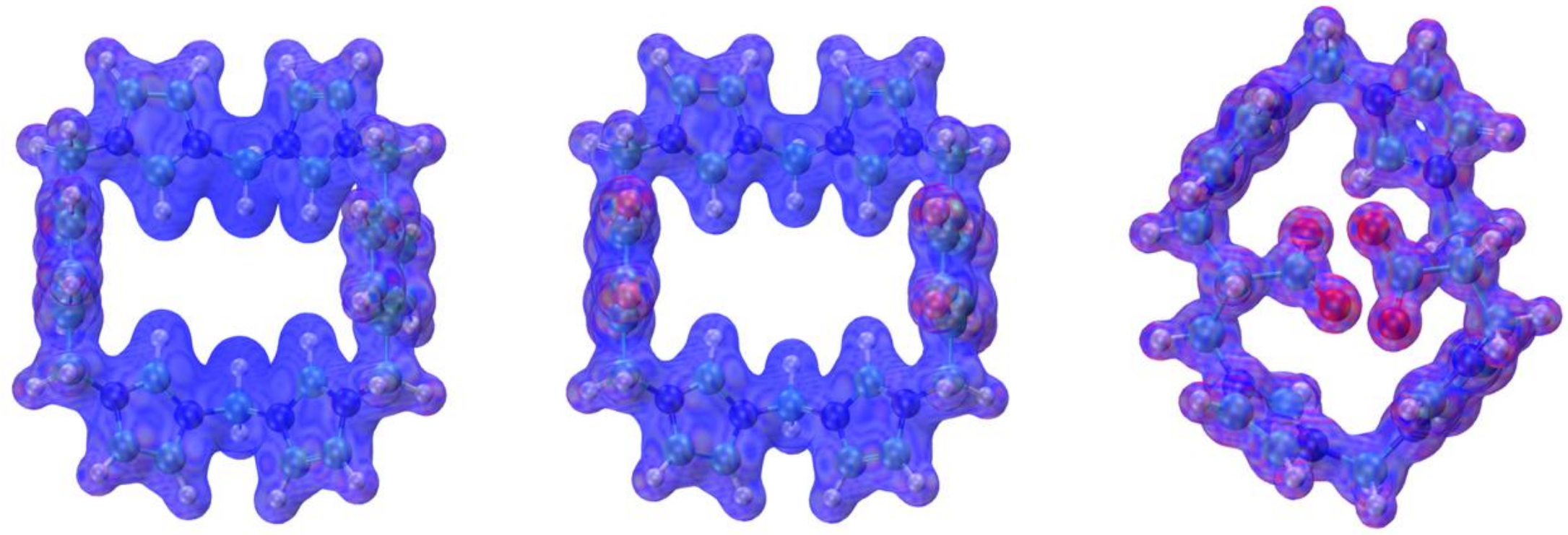

Figure S-61. Molecular electrostatic potentials for **PTI** (left), **FPTI** (middle), and **CTI** (right) (isovalue = 0.6). Red and blue colors indicate surface regions with negative and positive charge density, respectively (attractive or repulsive, respectively, force for a positive probe charge).

The force field parameters for PFOS and PFBS were adapted from previous literature.[21] That is, we retained all bonded parameter (bond stretch, angle bend, and dihedral rotation), all Lennard-Jones parameters, and the partial charges for the sulfonate headgroup. However, the model by Barbosa et al.[21] utilized site-specific partial charges on the tail group (e.g., the three partial charges on the F atoms of the terminal CF_3 group are not identical). Here, CM5 charge calculations were used as a guide to update the partial charges on the fluorous tail, selecting the same partial charge for all fluorine atoms irrespective of position, setting the partial charge for all but the α-carbon to be the negative of the sum of its bonded F atoms, and, finally, setting the partial charge on the α-carbon to yield a model with a net charge of -1 |e|. The TIP3P water model[22] and the LJ parameters for chloride anion[23] were selected because they are consistent with the GAFF.[16] Lennard-Jones (LJ) parameters were obtained from the GAFF database using the Amber TLeap tool.[18] Force field parameters for **PTI**, **FPTI**, **CTI**, **PSMI**, **DSMI**, PFOS, and PFBS are provided as GROMACS[25,26] compatible .itp files as Supporting Information (zipped folder).

Long-range electrostatic interactions were evaluated with the particle-mesh Ewald (PME) method,[24] employing a 12 Å real-space cutoff, fourth-order spline interpolation, and a Fourier grid spacing of 1.6 Å. Short-range van der Waals interactions were truncated at 12 Å.

7.2 Molecular Dynamics Details

Molecular dynamics (MD) simulations in the isobaric−isothermal ensemble (*NPT*) ensemble were conducted using the GROMACS software package, version 2022.[25,26] The leap-frog integrator was used for all simulations. All covalent bonds involving hydrogen atoms were constrained with the LINCS algorithm,[27] which enabled use of a 2-fs time step without compromising numerical stability. Periodic

boundary conditions were imposed in all three dimensions. Neighbor lists were constructed with the Verlet cutoff scheme using a grid-based search.[28] The system temperature was maintained at 300 K using the velocity-rescaling thermostat (V-rescale)[29] with a coupling time constant of 0.1 ps. The applied pressure was set to 1 bar using the Parrinello-Rahman barostat[30] with a pressure relaxation time constant of 5.0 ps. Trajectory coordinates were saved every 1 ps, and energies and log data were written every 10 ps.

7.3 System Set-up and Simulation details

MD simulations were performed for aqueous solutions containing about 56000 or 111000 solvent molecules and combinations of **PTI**, **FPTI**, or **CTI** receptors, PFOS or PFBS anions, **PSMI** or **DSMI** dye molecules, and, if needed, sodium or chloride counterions. Table S-4 provides information on the specific combinations of non-solvent species investigated here. For systems with 56000 water molecules, a system with 16 receptors corresponds to a 16 mM concentration. An initial value of the system volume was estimated using the number density of water at ambient conditions. The system was assembled using the "gmx editconf" program and first subjected to energy minimization to remove steric clashes. This was followed by two 1-ns pre-equilibration periods: first in the canonical (*NVT*) ensemble at 300 K, then in the isothermal–isobaric (*NPT*) ensemble at 300 K and 1 bar. Following the pre-equilibration periods, trajectories ranging from 200 to 300 ns were performed in the *NPT* ensemble where three independent runs were carried out for each system by reassigning the initial velocities.

For the systems with 56000 water molecules (box length of 12 nm), a 100-ns simulation period requires about 40 hours of wall-clock time using 8 CPU cores and one GPU. Decreasing the **XTI** concentration to from 16 mM to 1 mM (as used in the experiments), increasing the number of **XTI** macrocycles and PFAS anions by 10 (to allow for formation of larger aggregates), and increasing the simulation lengths to 1 μs (accounting for longer diffusion pathways in larger systems and a smaller thermodynamic driving force for aggregation yields an estimate that demonstrates that getting close to the experimental conditions is not feasible with current computational resources.

Table S-10. Number of species (receptor, PFAS, dye, water, and counter ions) in each system. The last part of the system name indicates deviations from 16 mM **XTI**, stoichiometric ratio, or number of water molecules. The last column reports the average box length computed over the final 20 ns (within 95% confidence intervals).

System	**XTI**	PFAS	Dye	Water	Na^+	Cl^-	L (Å)	Snapshot Figure
**PTI**/PFBS	16	64	0	56000	0	0	119.970 ± 0.002	66
**PTI**/PFBS-low	8	32	0	56000	0	0	119.676 ± 0.001	4b
**PTI**/PFOS	16	64	0	56000	0	0	120.225 ± 0.003	62
**PTI**/PFOS-low	8	32	0	56000	0	0	119.806 ± 0.002	4d
**PTI**/PFOS-large	16	64	0	111000	0	0	150.501 ± 0.002	65
**PTI**/PFOS-sub	16	32	0	55968	0	32	119.905 ± 0.001	72
**PTI**/FPOS-supra	8	64	0	55968	32	0	120.079 ± 0.003	71
**PTI**/PFOS/**PSMI**	16	64	2	55598	0	2	120.244 ± 0.002	4a
**FPTI**/PFBS	16	64	0	56000	0	0	119.991 ± 0.004	74
**FPTI**/PFOS	16	64	0	56000	0	0	120.247 ±0.002	64
**FPTI**/PFOS-sub	16	32	0	55568	0	32	119.928 ± 0.003	73
**FPTI**/PFOS/**PSMI**	16	64	2	55598	0	2	120.265 ± 0.001	63
**CTI**/PFBS-supra	16	64	0	55568	32	0	119.903 ± 0.001	75
**CTI**/PFOS	16	32	0	56000	0	0	119.884 ± 0.002	68
**CTI**/PFOS-supra	16	64	0	55968	32	0	120.160 ± 0.001	69
**CTI**/PFOS/**DSMI**	16	64	2	55970	30	0	120.178 ± 0.001	70
NaPFOS	0	64	0	55936	64	0	119.938 ± 0.001	4c
NaPFBS	0	64	0	55936	64	0	119.677 ± 0.002	67

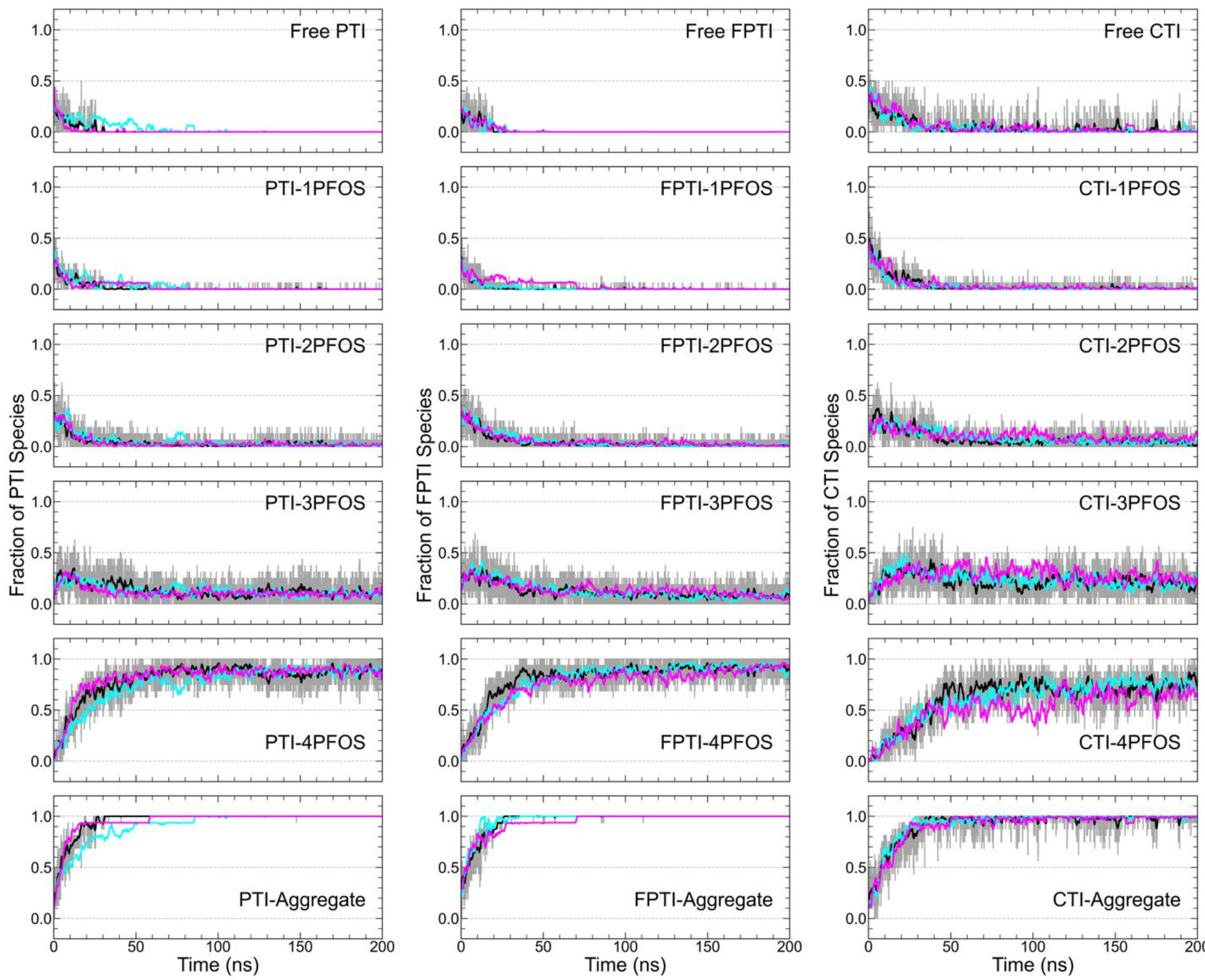

Figure S-62. Time evolution of receptor:PFOS association and multireceptor aggregation for **PTI**:PFOS (left), **FPTI**:PFOS (middle), **CTI**:PFOS (right) systems with a concentration ratio of 16 mM:64 mM (i.e., the **CTI**:PFOS ratio is not stochiometric) and without dye molecules. The top row shows the fraction of **XTI** receptors that are not associated with any PFOS molecule. The next four rows represent the fractions of receptor being associated directly with 1 to 4 PFOS molecules. The bottom row shows the fraction of receptors involved in aggregates containing multiple receptors (determined using the "neighbors-of-neighbors" search algorithm). The grey lines indicate values found in a given snapshot for run-1; the black, cyan, and magenta lines show the moving average using a window size of 0.5 ns for run-1, run-2, and run-3, respectively.

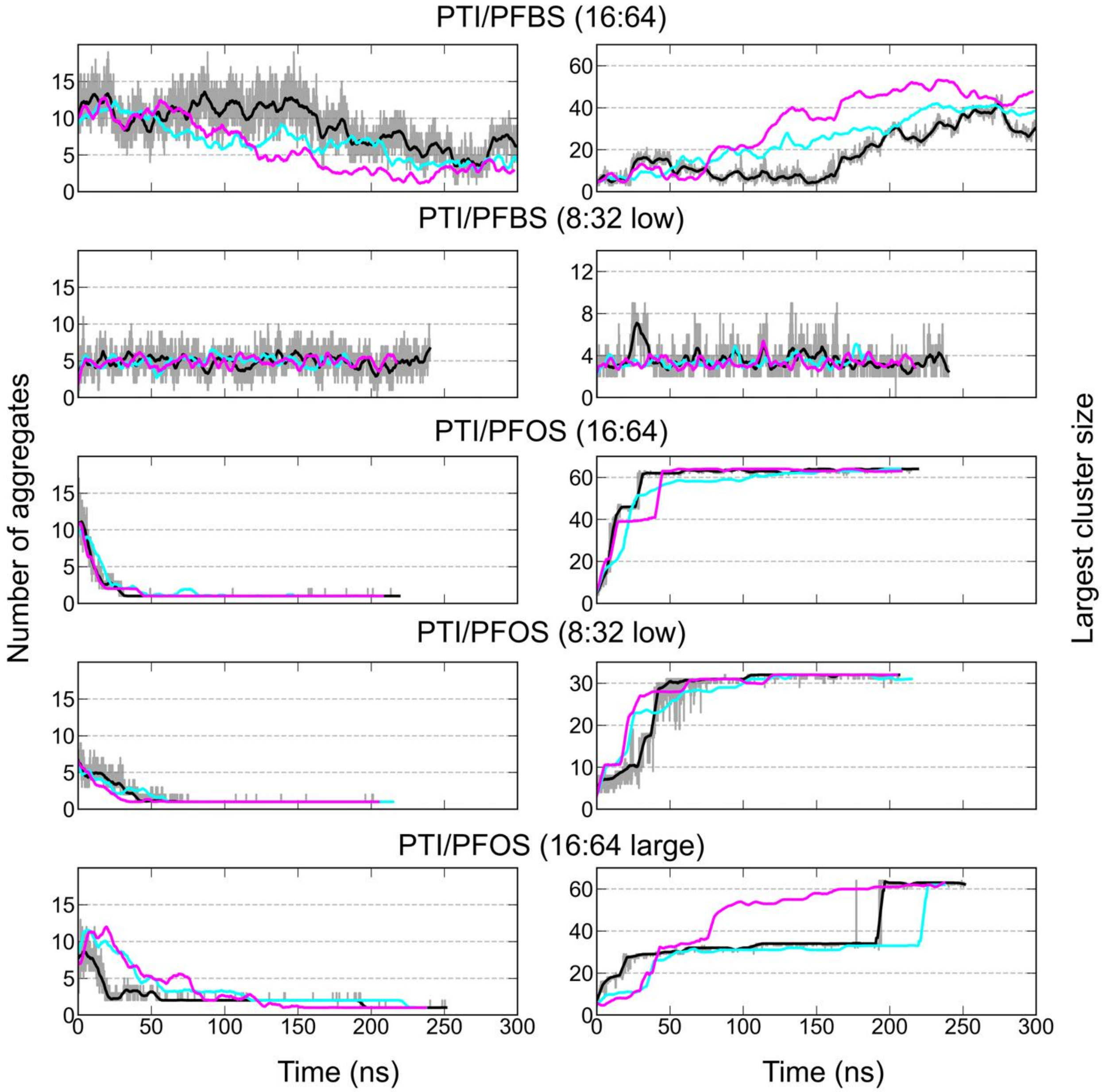

Figure S-63. Time evolution of the number of PFAS containing aggregates (left column) and of the largest aggregate in terms of the number of its PFAS anions (right column) for **PTI**:PFAS systems with stoichiometric ratio. The name of each system (see Table S-10) and the concentrations (in mM) of **PTI** and PFAS are given on top of each row. The grey lines indicate values found in a given snapshot for run-1; the black, cyan, and magenta lines show the moving average using a window size of 5 ns for run-1, run-2, and run-3, respectively.

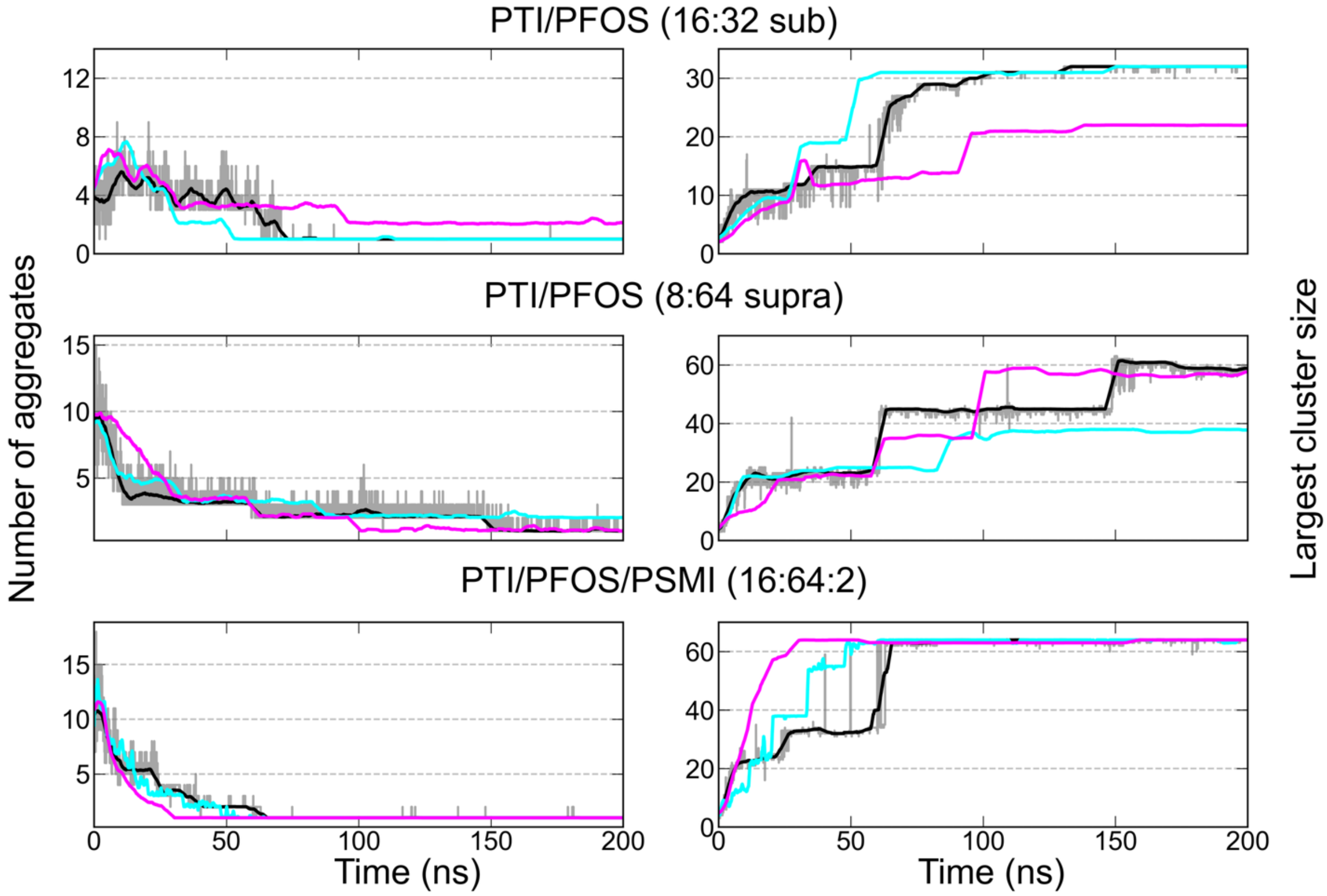

Figure S-64. Time evolution of the number of PFAS containing aggregates (left column) and of the largest aggregate in terms of the number of its PFAS anions (right column) for **PTI**:PFAS systems with sub- and supra-stoichiometric ratios or containing **PSMI**. The name of each system (see Table S-10) and the concentrations (in mM) of **PTI** and PFAS are given on top of each row. The grey lines indicate values found in a given snapshot for run-1; the black, cyan, and magenta lines show the moving average using a window size of 5 ns for run-1, run-2, and run-3, respectively.

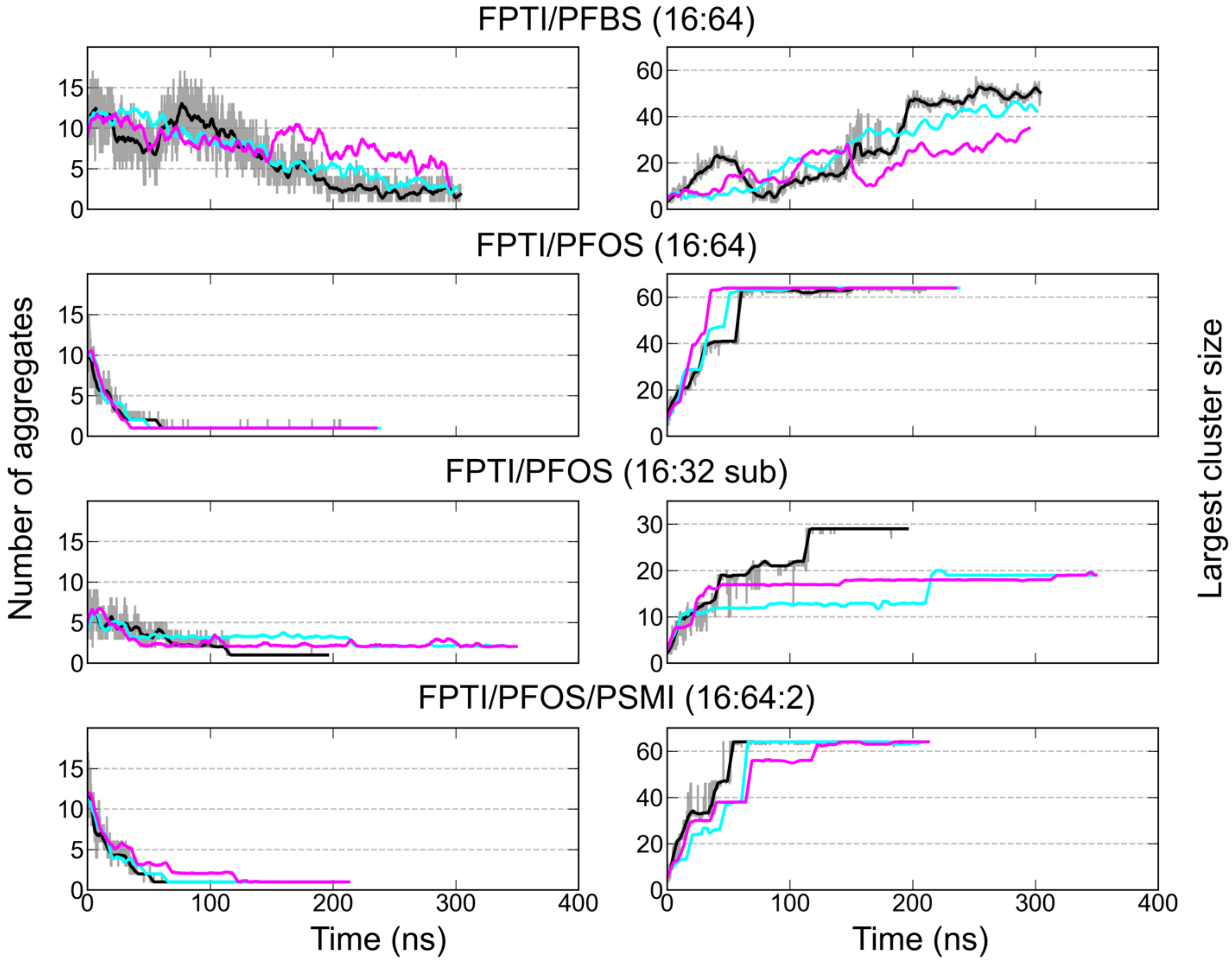

Figure S-65. Time evolution of the number of PFAS containing aggregates (left column) and of the largest aggregate in terms of the number of its PFAS anions (right column) for **FPTI**:PFAS systems. The name of each system (see Table S-10) and the concentrations (in mM) of **FPTI** and PFAS are given on top of each row. The grey lines indicate values found in a given snapshot for run-1; the black, cyan, and magenta lines show the moving average using a window size of 5 ns for run-1, run-2, and run-3, respectively.

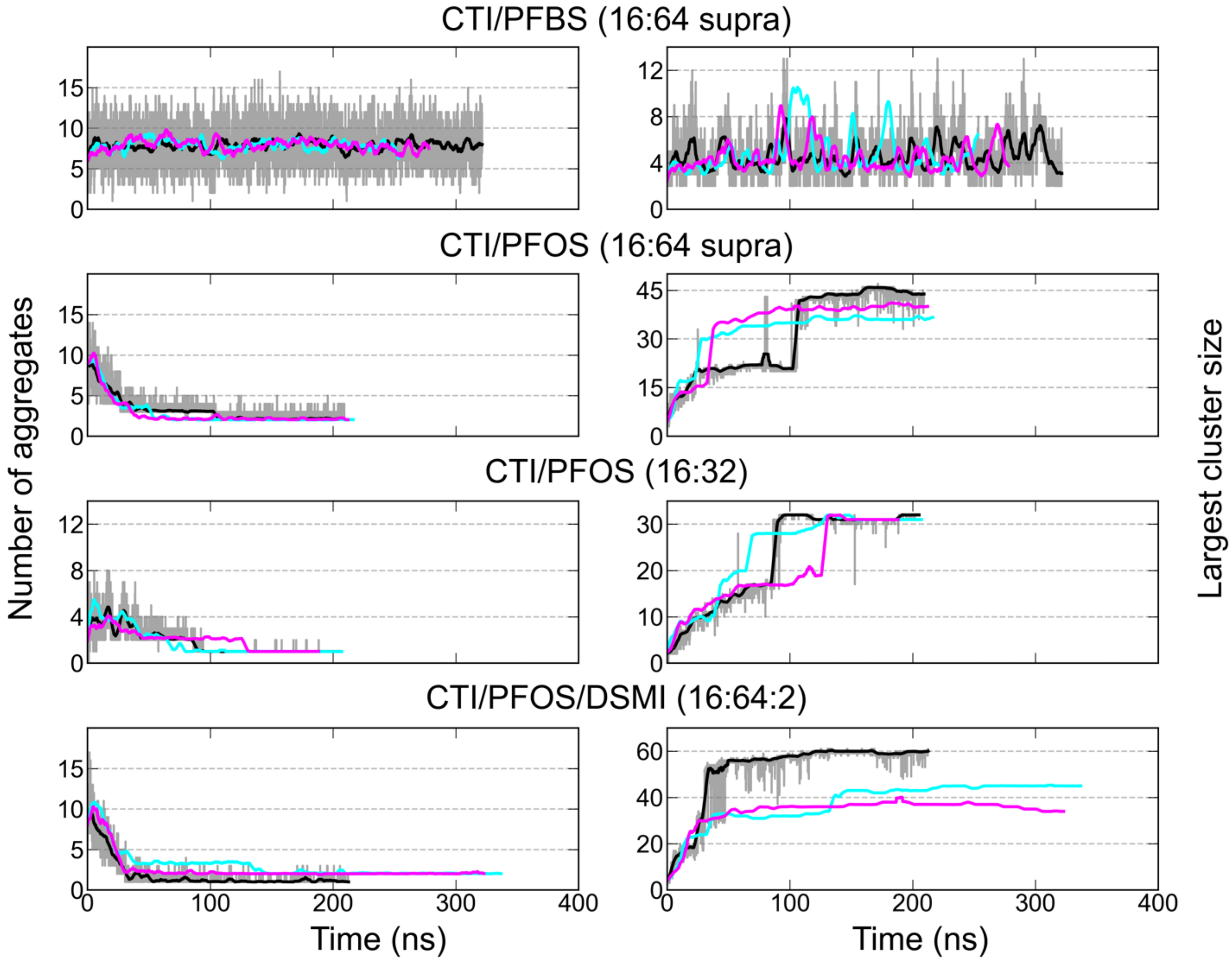

Figure S-66. Time evolution of the number of PFAS containing aggregates (left column) and of the largest aggregate in terms of the number of its PFAS anions (right column) for **CTI**:PFAS systems without or with **DSMI**. The name of each system (see Table S-10) and the concentrations (in mM) of **CTI**, PFAS, and **DSMI** are given on top of each row. The grey lines indicate values found in a given snapshot for run-1; the black, cyan, and magenta lines show the moving average using a window size of 5 ns for run-1, run-2, and run-3, respectively.

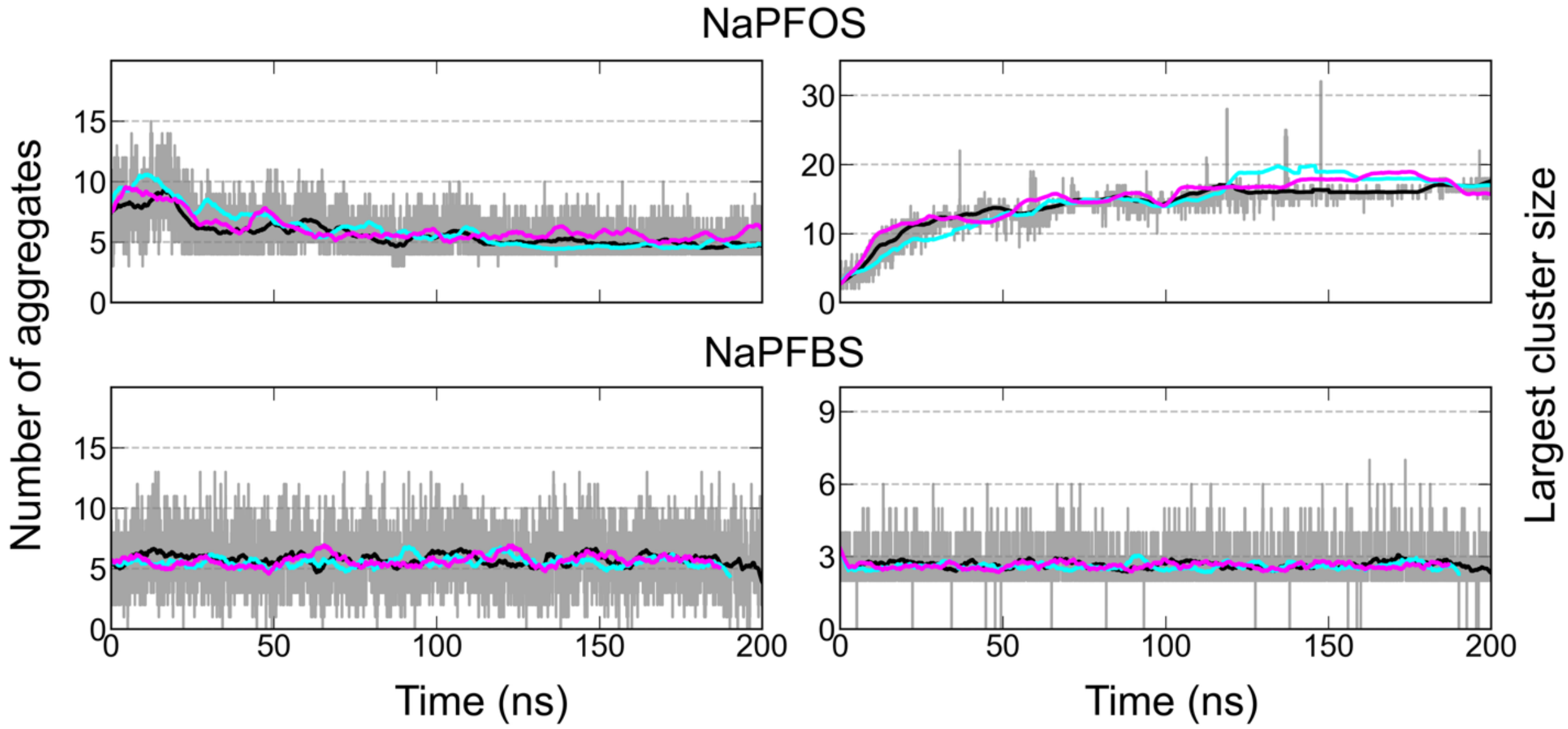

Figure S-67. Time evolution of the number of PFAS containing aggregates (left column) and of the largest aggregate in terms of the number of its PFAS anions (right column) for NaPFAS systems without **XTI** and dye. The name of each system (see Table S-10) and the concentrations (in mM) of PFAS are given on top of each row. The grey lines indicate values found in a given snapshot for run-1; the black, cyan, and magenta lines show the moving average using a window size of 5 ns for run-1, run-2, and run-3, respectively.

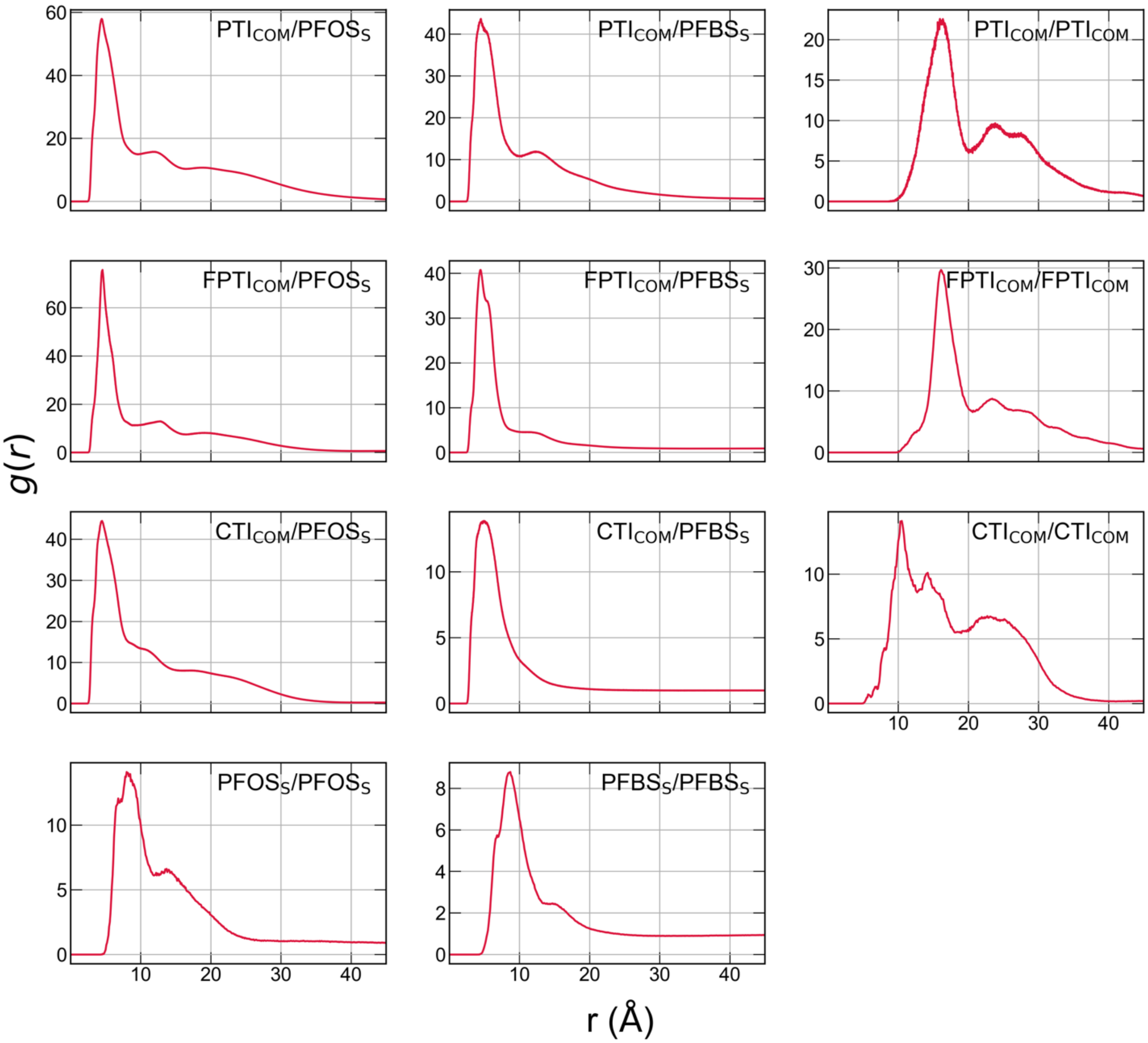

Figure S-68. Radial distribution functions (RDFs) evaluated to select the distance criteria used to determine whether a pair of sites belong to the same aggregate. All RDFs correspond to systems with 16 mM **XTI** macrocycles and 64 mM PFOS or PFBS anions, and a bin width of 0.05 Å is used. For the COM−S distance between **XTI** macrocycles and PFAS anions, the upper bound is set at 10 Å, and this criterion holds for both tetra- and dicationic receptors. For the S−S distance of two PFAS anions, the upper bound is set at 12 Å. The RDFs for two macrocycles do not lend themselves to a well-defined cluster criterion because of the shoulder at shorter distances and the large distance (r = 20 Å) found for the first minimum in the RDFs for **PTI** and **FPTI**.

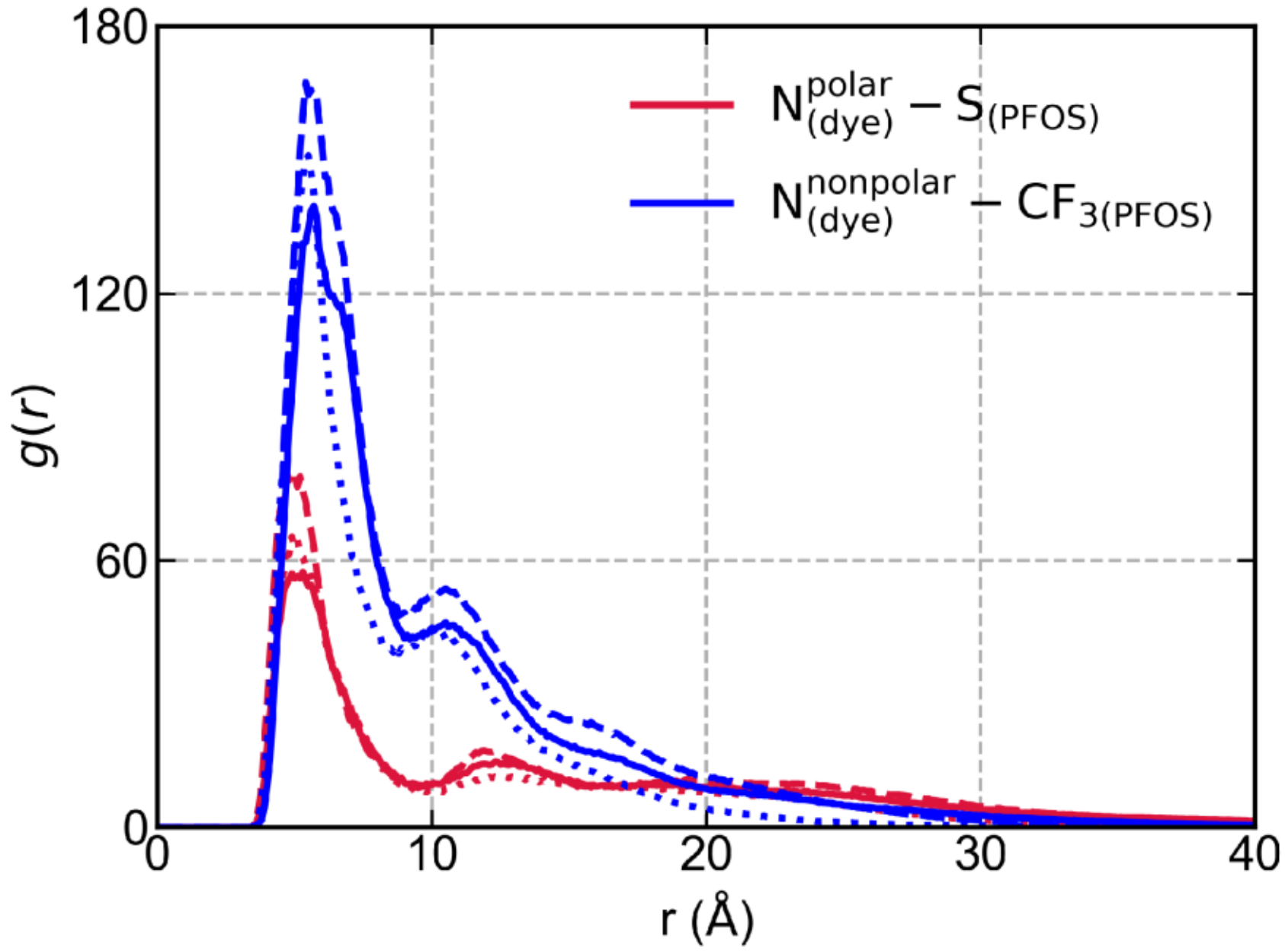

Figure S-69. Radial distribution functions (RDFs) between polar-polar (red lines) and nonpolar-nonpolar descriptors (blue lines) of the dye cation and PFOS anion. Solid and dashed lines represent **PSMI**/PFOS in the **PTI** and **FPTI** systems, respectively, and dotted lines represent **DSMI**/PFOS in the **CTI** system. RDFs were computed from simulations performed at a **PTI**:PFOS:dye ratio of 16:64:2.

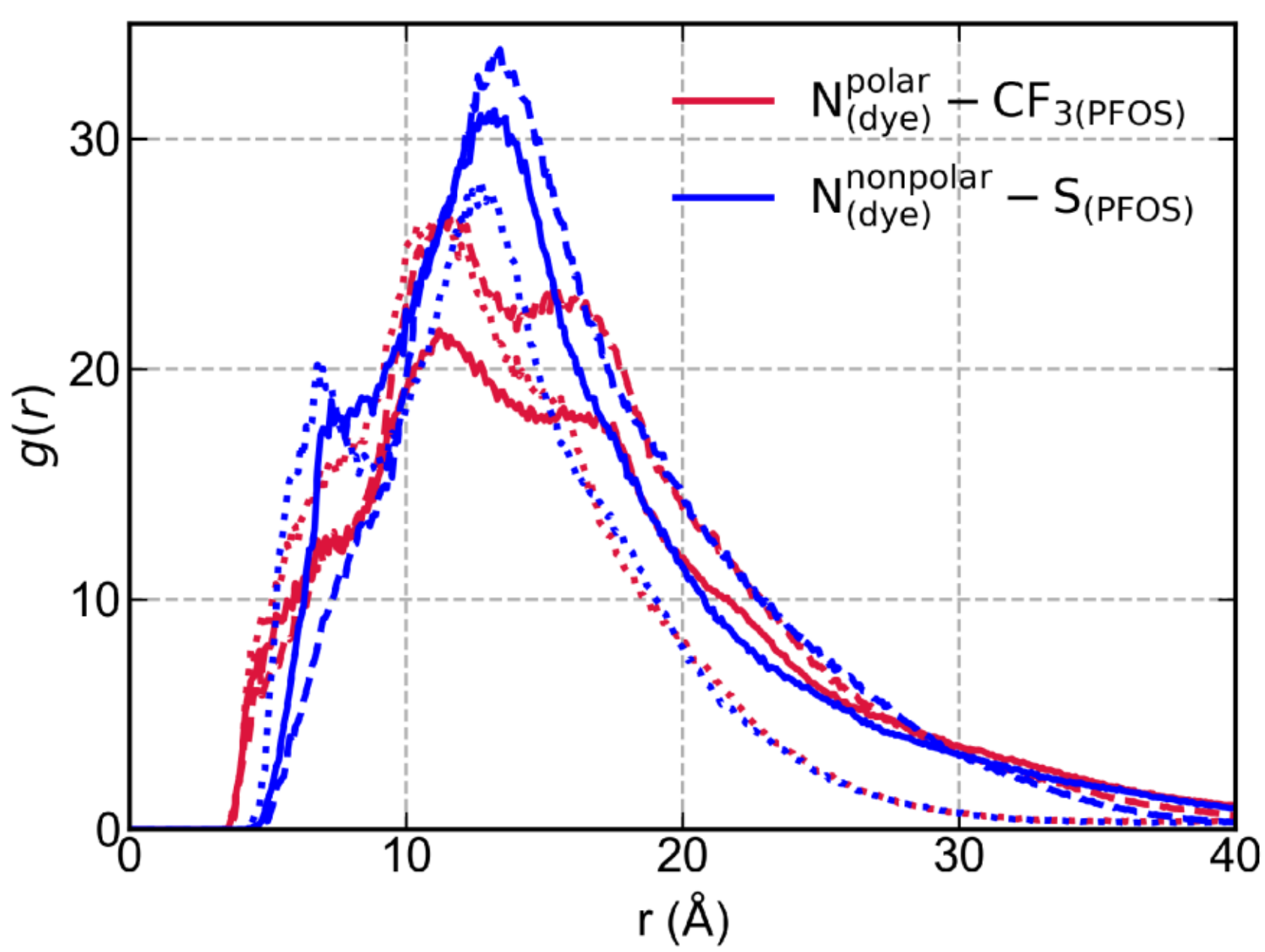

Figure S-70. Radial distribution functions (RDFs) between polar and nonpolar descriptors of the dye cation and PFOS anion. Red lines show the RDFs between polar end of the dye with nonpolar site of PFOS. Blue lines show the RDF between nonpolar site of the dye and polar site of PFOS. Solid and dashed lines represent **PSMI**/PFOS in the **PTI** and **FPTI** systems, respectively, and dotted lines represent **DSMI**/PFOS in the **CTI** system. RDFs were computed from simulations performed at a **PTI**:PFOS:dye ratio of 16:64:2.

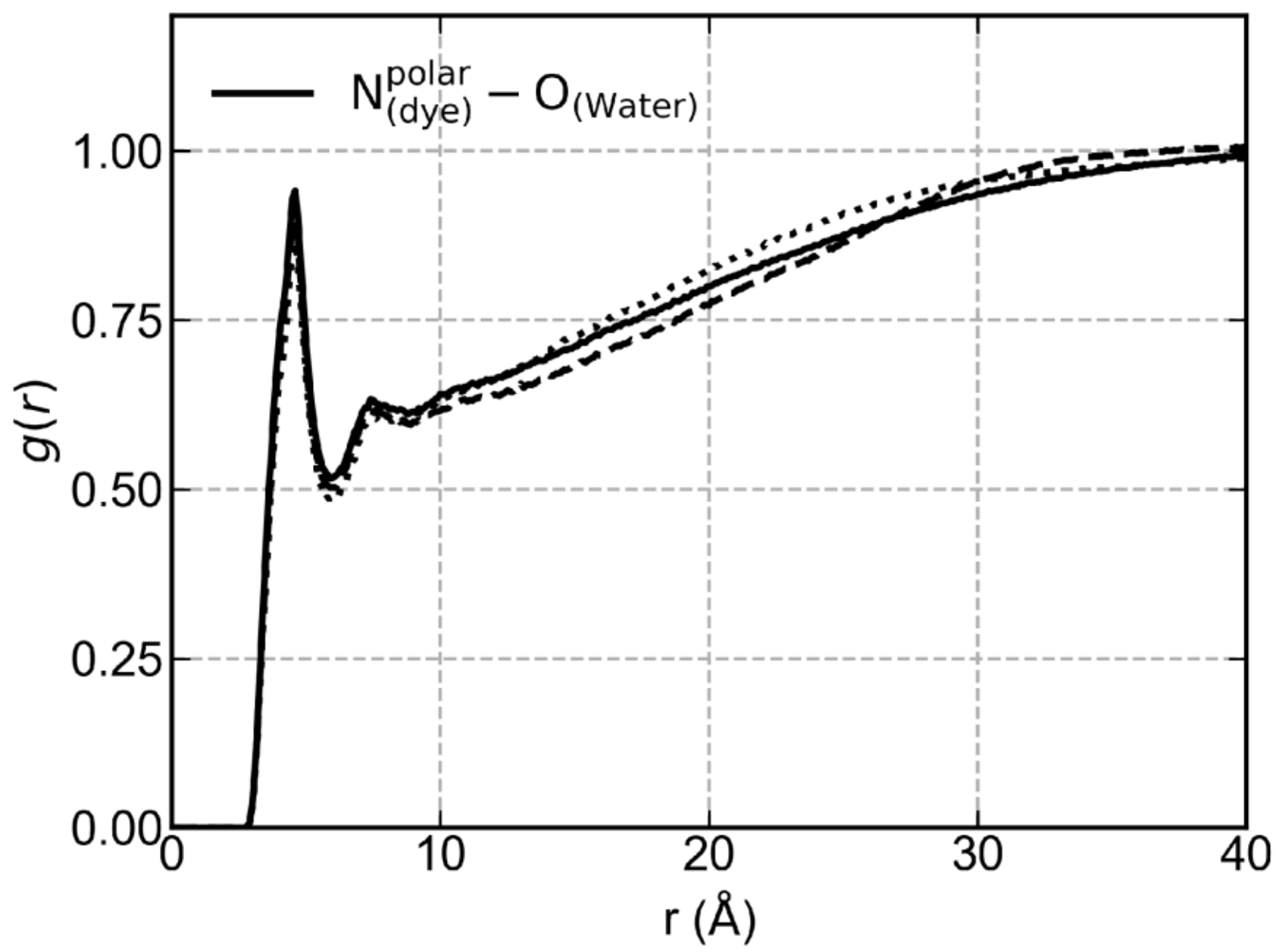

Figure S-71. Radial distribution functions (RDFs) between dye cation and water. RDF between polar descriptor of dye and O-atom of water are shown using black lines. Solid and dashed lines represent **PSMI**/PFOS in the **PTI** and **FPTI** systems, respectively, and dotted lines represent **DSMI**/PFOS in the **CTI** system. RDFs were computed from simulations performed at a **PTI**:PFOS:dye ratio of 16:64:2.

7.4 Snapshots

Figures S-72 to S-85 show snapshots of the final configuration obtained from the longest trajectory (shown in black in Figures S-64 to S-66) for the various host:PFAS:dye combinations. In each case, all concentrations are given in units of mM (corresponding to the same number of molecules for simulations with 56K water molecules). **XTI** macrocycles and dye molecules are shown as spheres (using purple or orange and green for their respective C atoms), while PFOS/PFBS anions are shown in wireframe form (using yellow and red for S and O atoms, respectively). Water molecules are omitted for clarity.

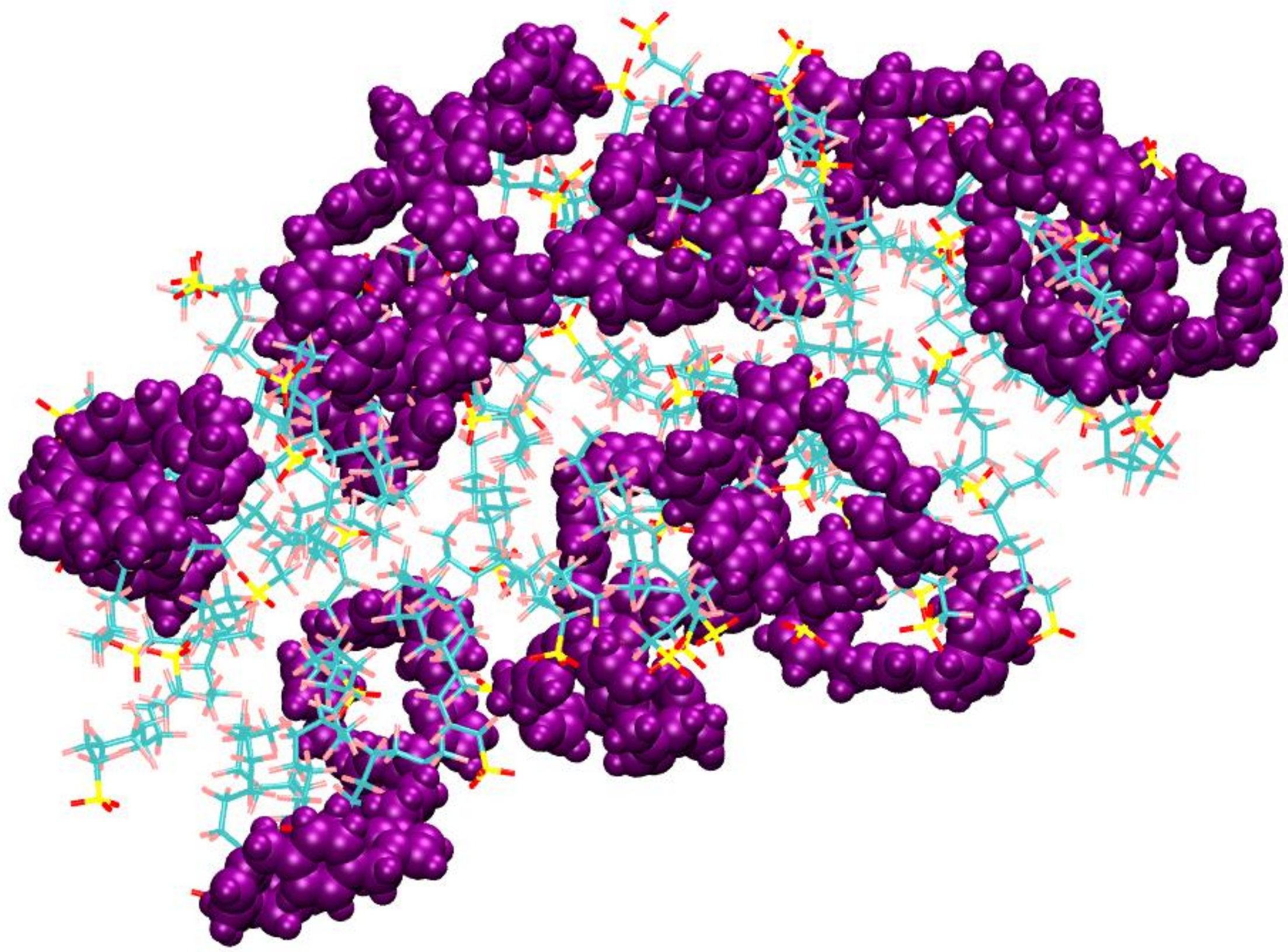

Figure S-72. Snapshot of the final configuration for **PTI**:PFOS (16:64).

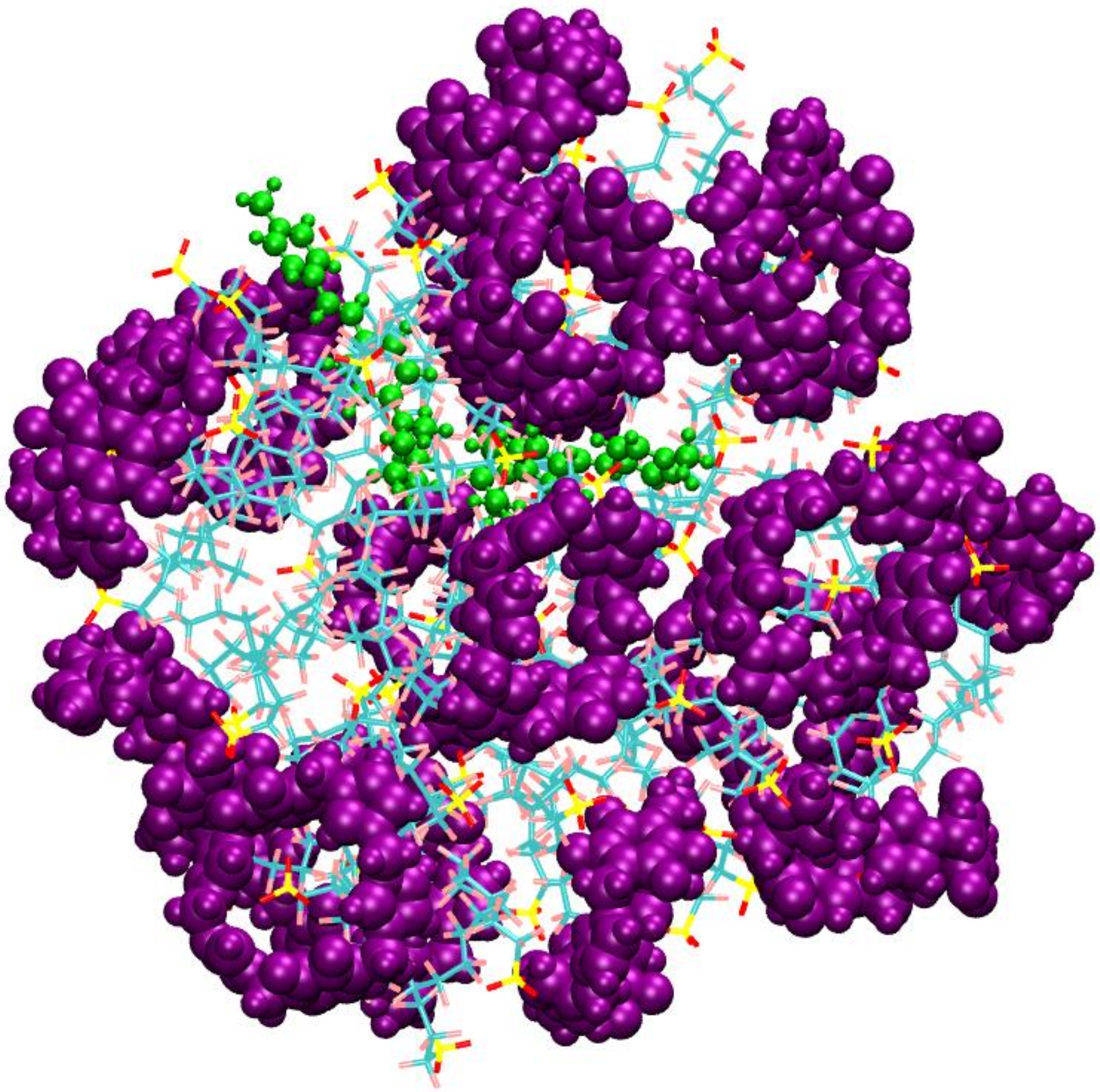

Figure S-73. Snapshot of the final configuration for **FPTI**:PFOS:**PSMI** (16:64:2).

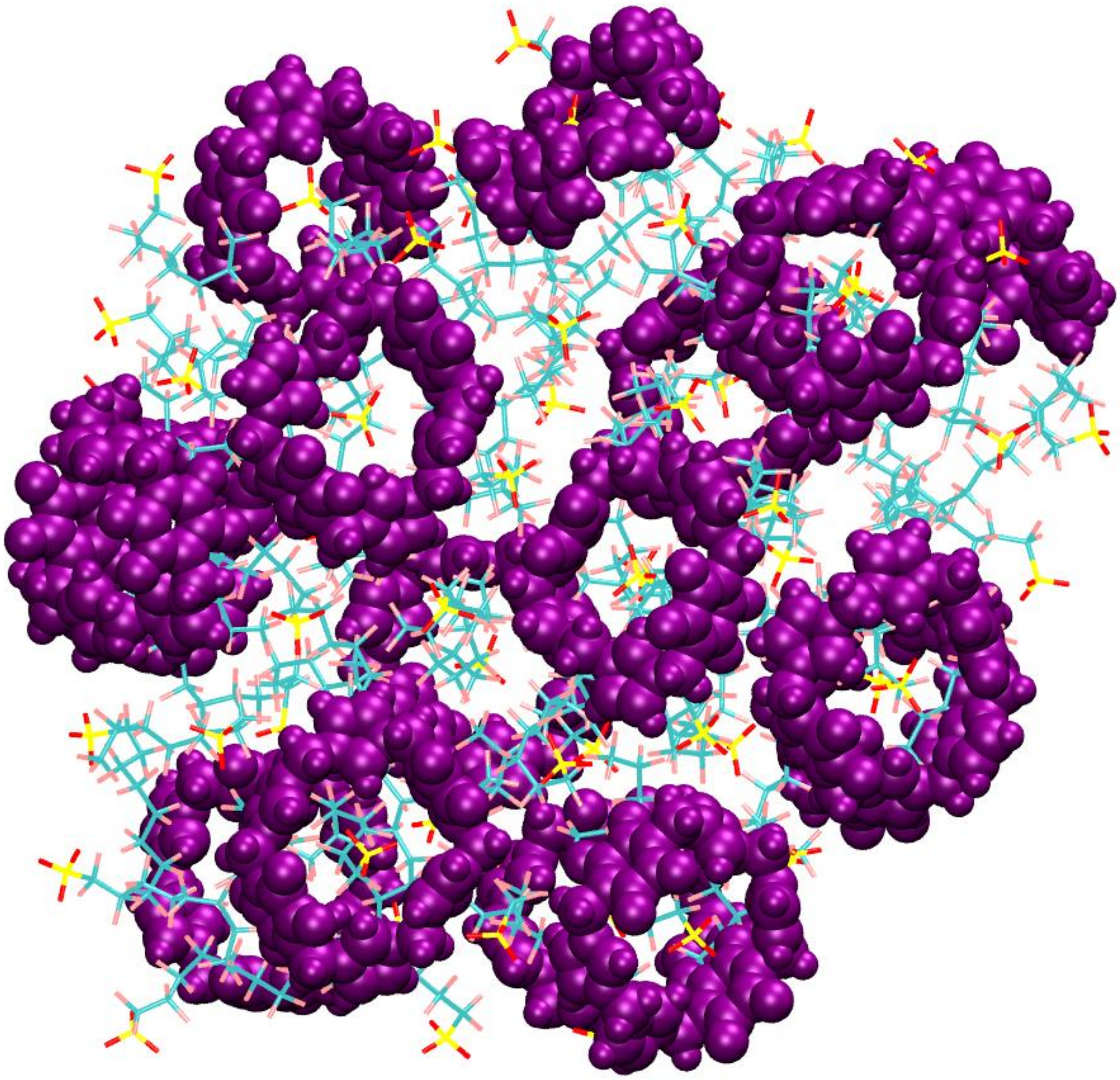

Figure S-74. Snapshot of the final configuration for **FPTI**:PFOS (16:64).

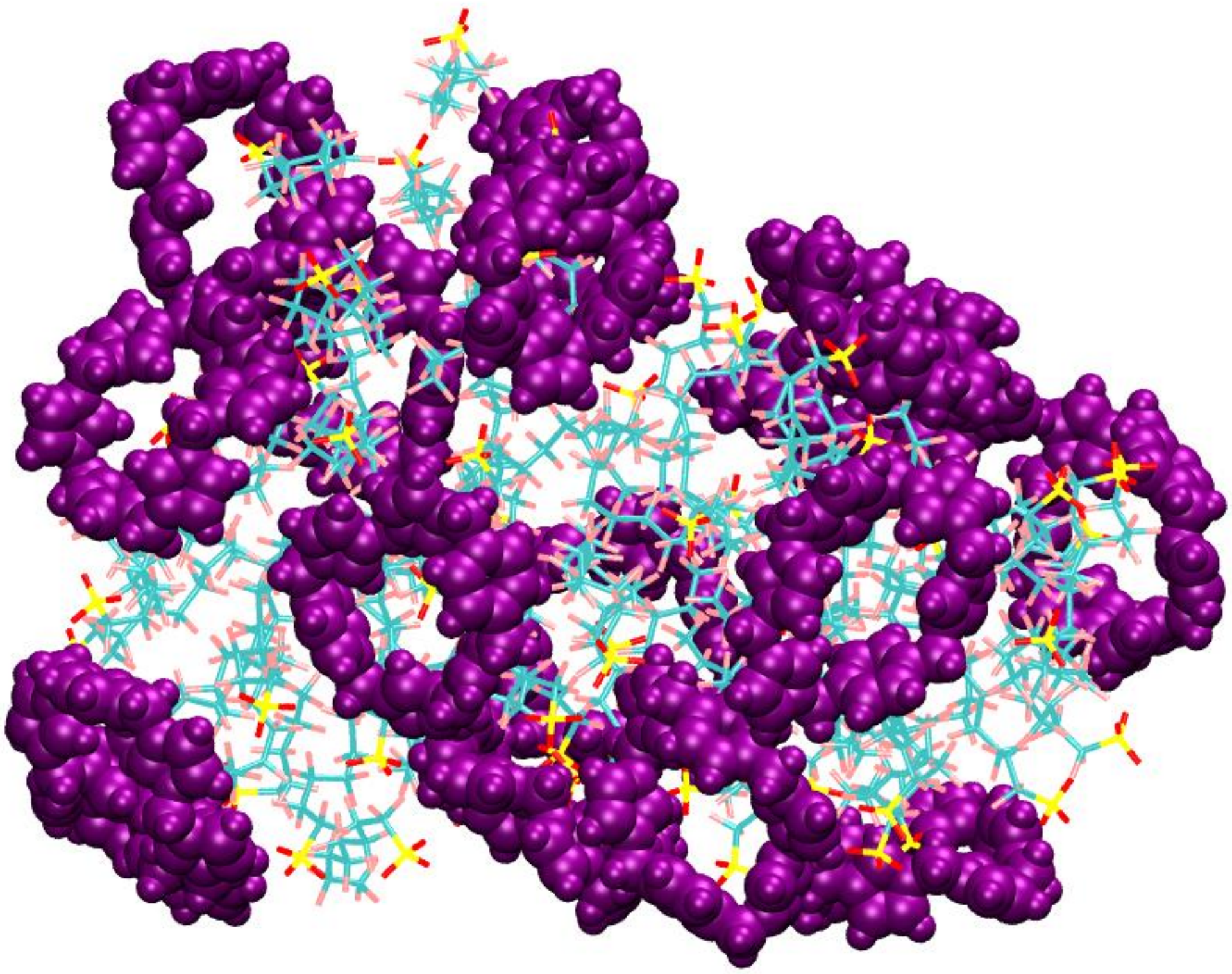

Figure S-75. Snapshot of the final configuration **PTI**:PFOS-large (16 receptors:64 PFOS at 8 mM and 32 mM).

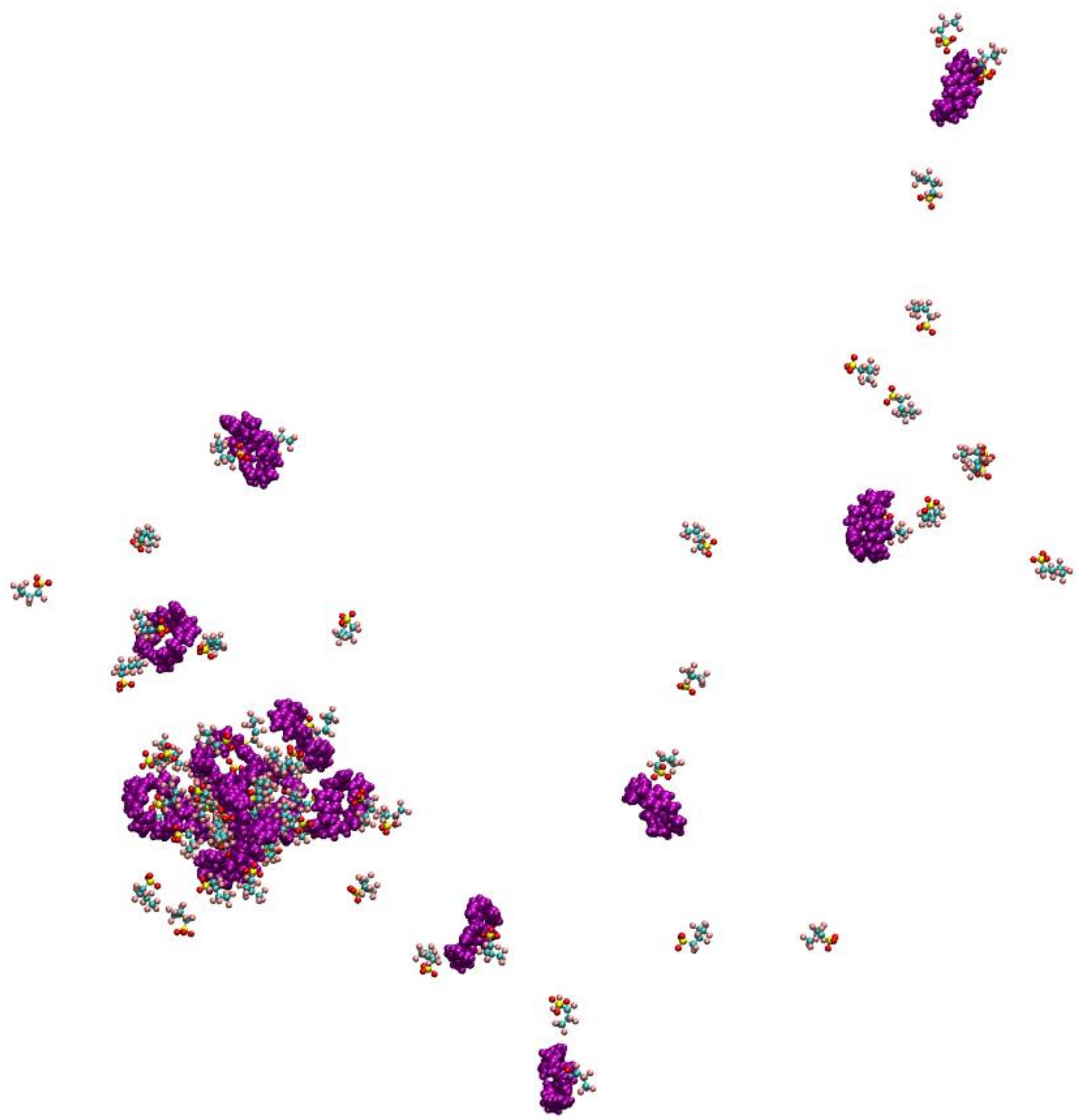

Figure S-76. Snapshot of the final configuration for **PTI**:PFBS (16:64).

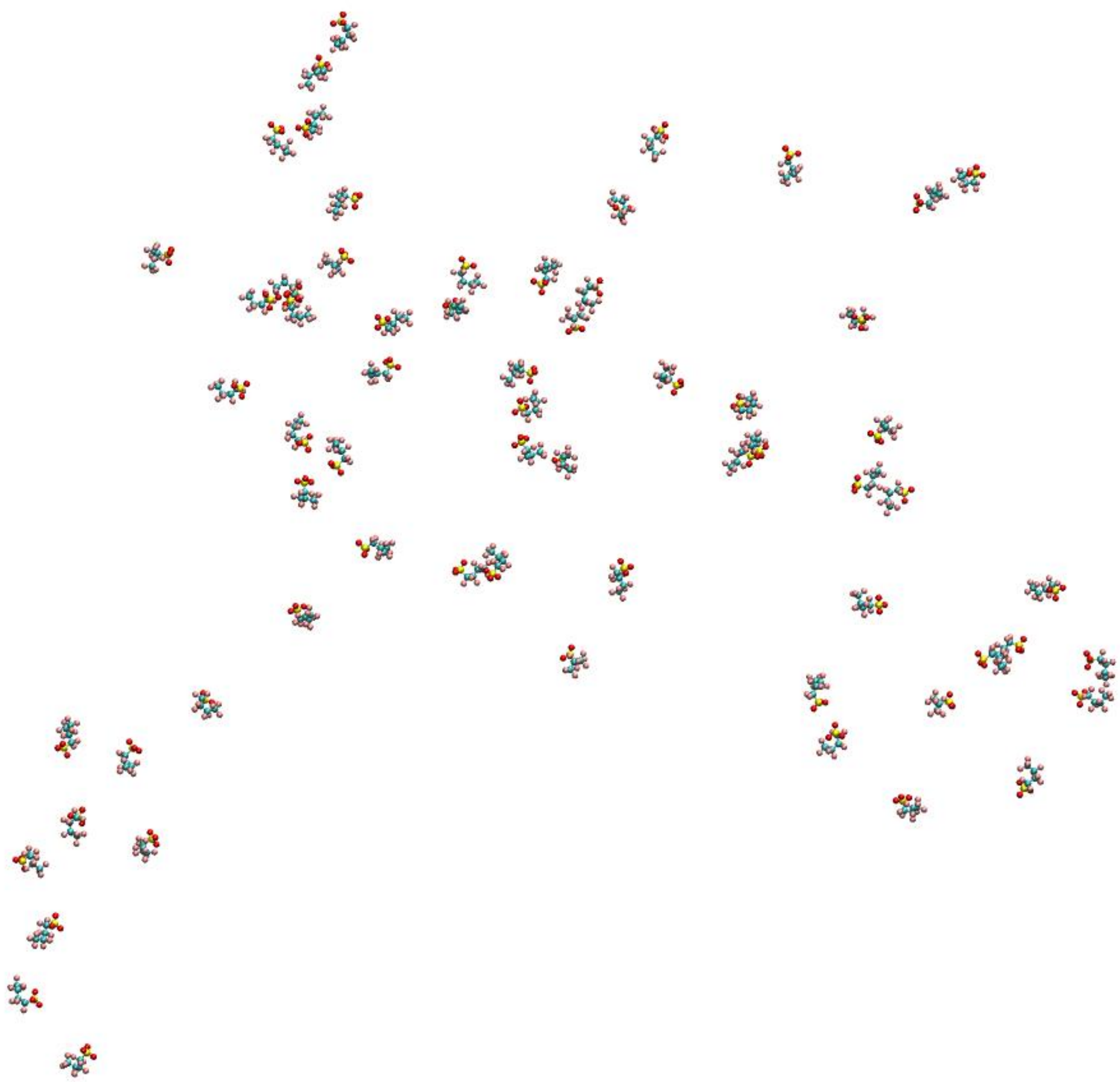

Figure S-77. Snapshot of the final configuration for **Na**PFBS.

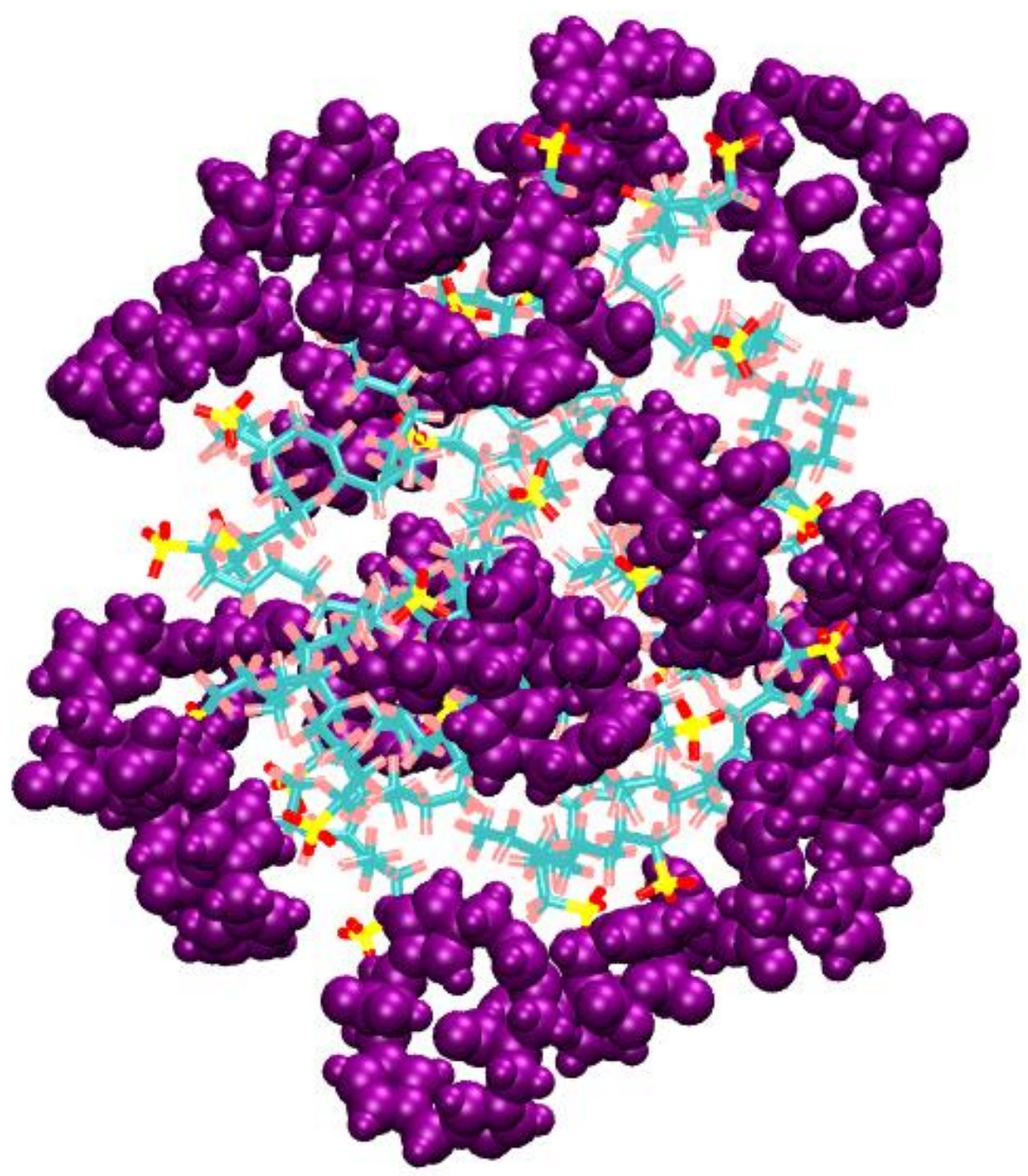

Figure S-78. Snapshot of the final configuration for **CTI**:PFOS (16:32).

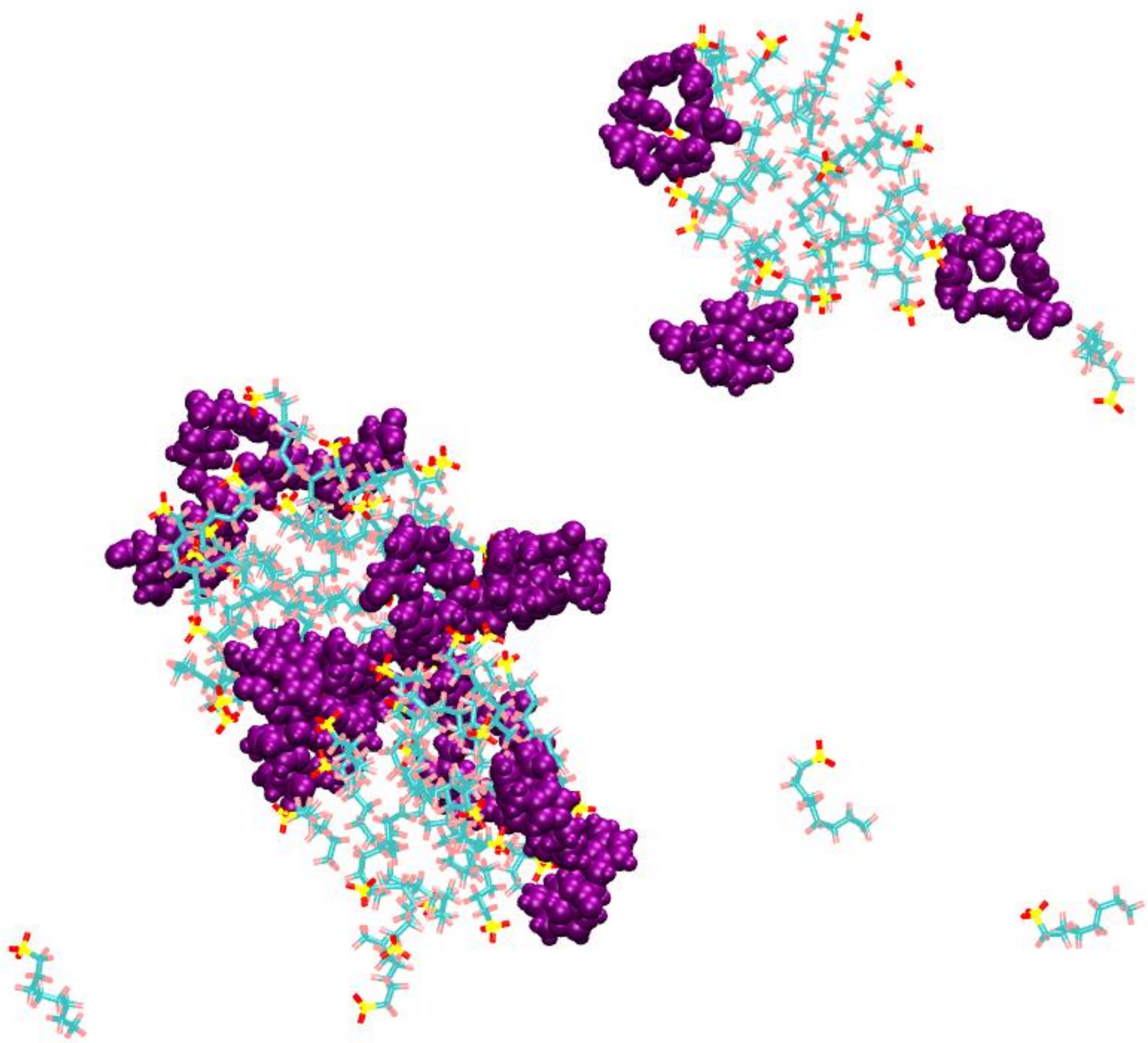

Figure S-79. Snapshot of the final configuration for **CTI**:PFOS-supra (16:64).

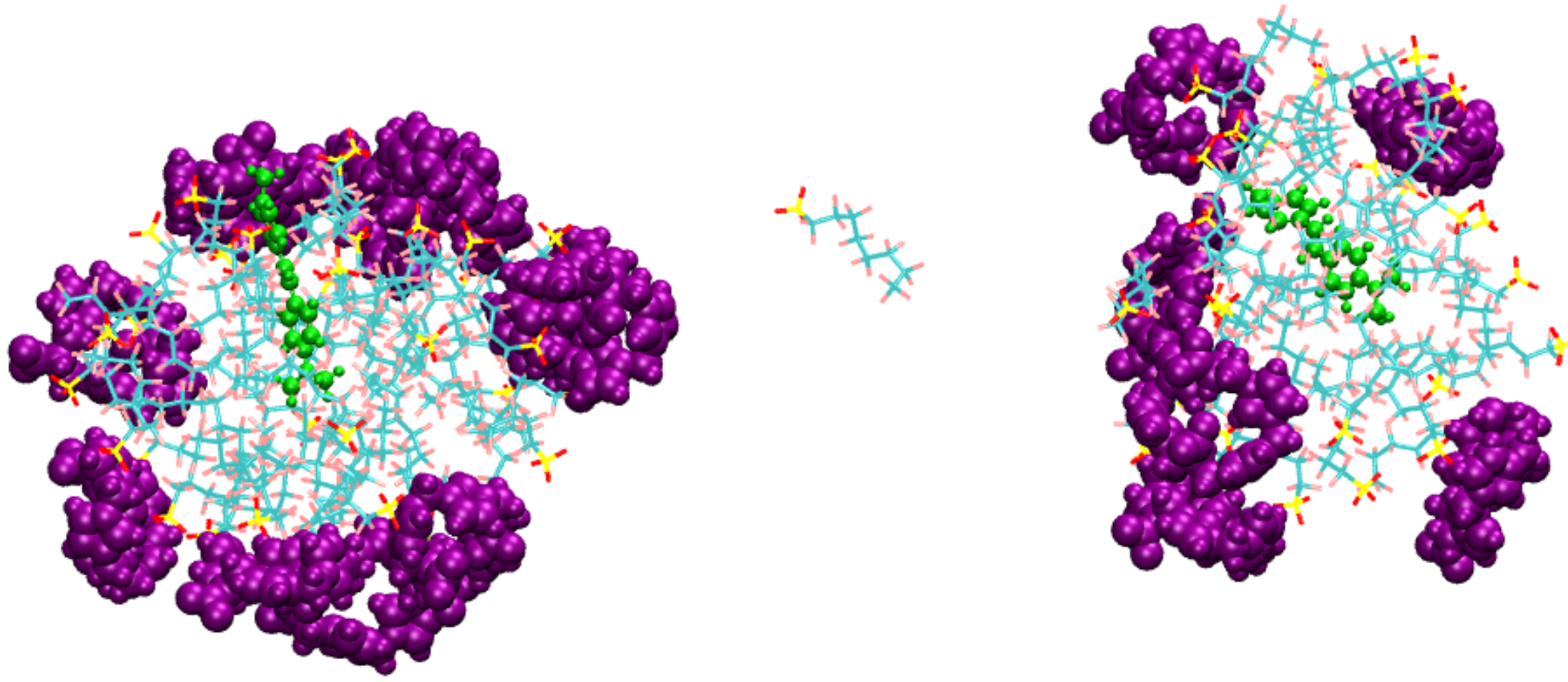

Figure S-80. Snapshot of the final configuration for **CTI**:PFOS:**DSMI** (16:64:2).

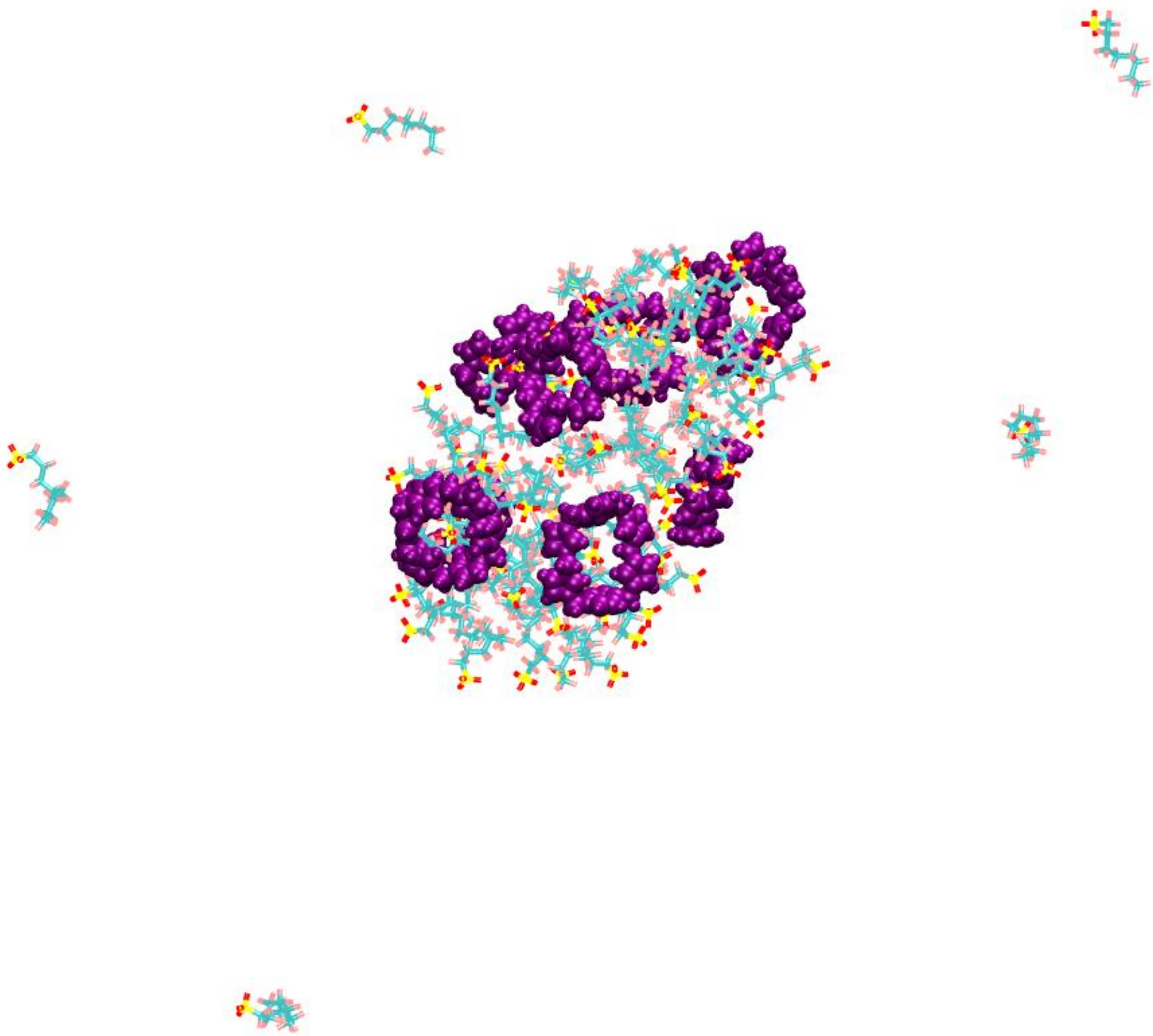

Figure S-81. Snapshot of the final configuration for **PTI**:PFOS-supra (8:64).

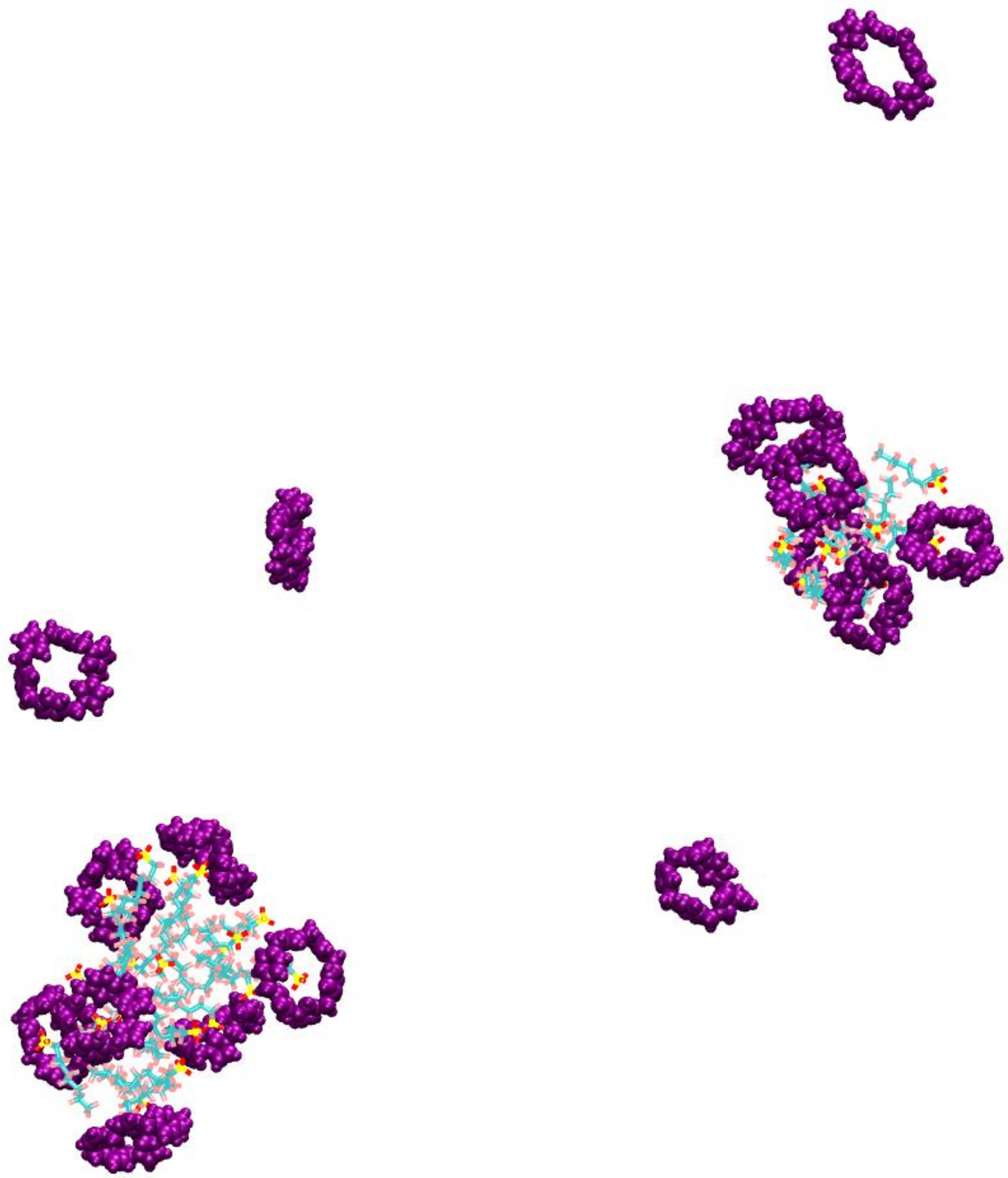

Figure S-82. Snapshot of the final configuration for **PTI**:PFOS-sub (16:32).

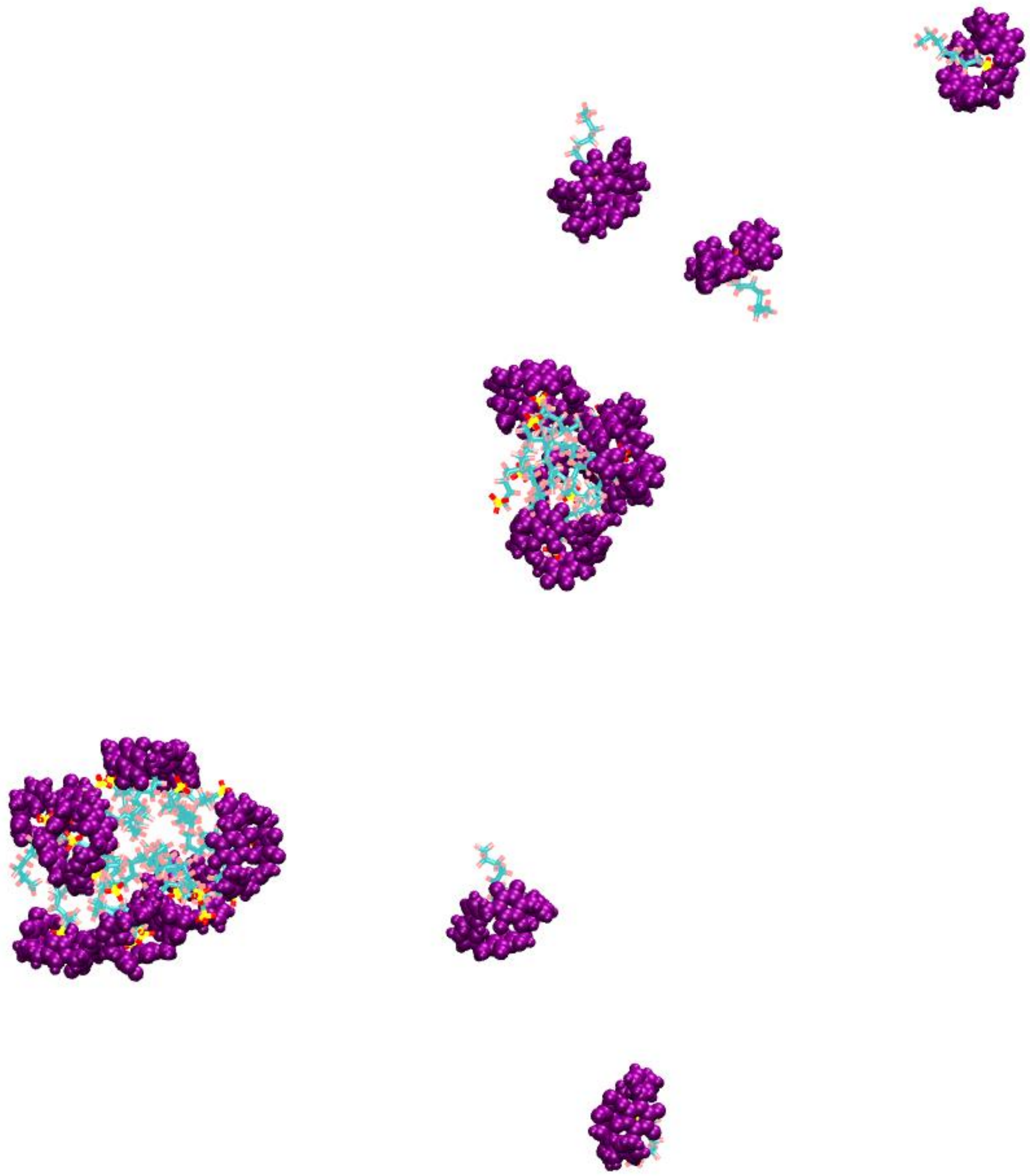

Figure S-83. Snapshot of the final configuration for **FPTI**:PFOS-sub (16:32).

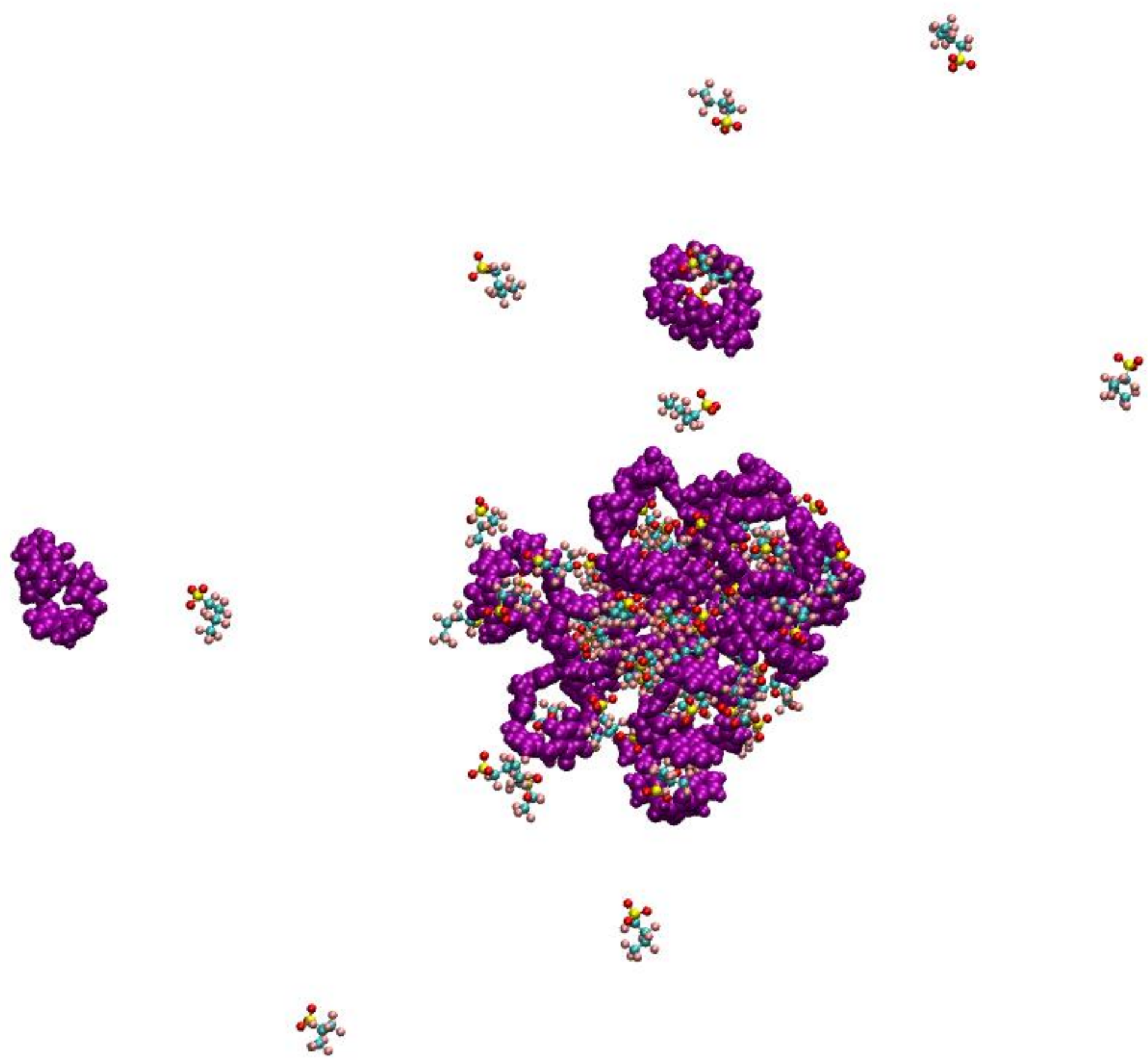

Figure S-84. Snapshot of the final configuration for **FPTI**:PFBS (16:64).

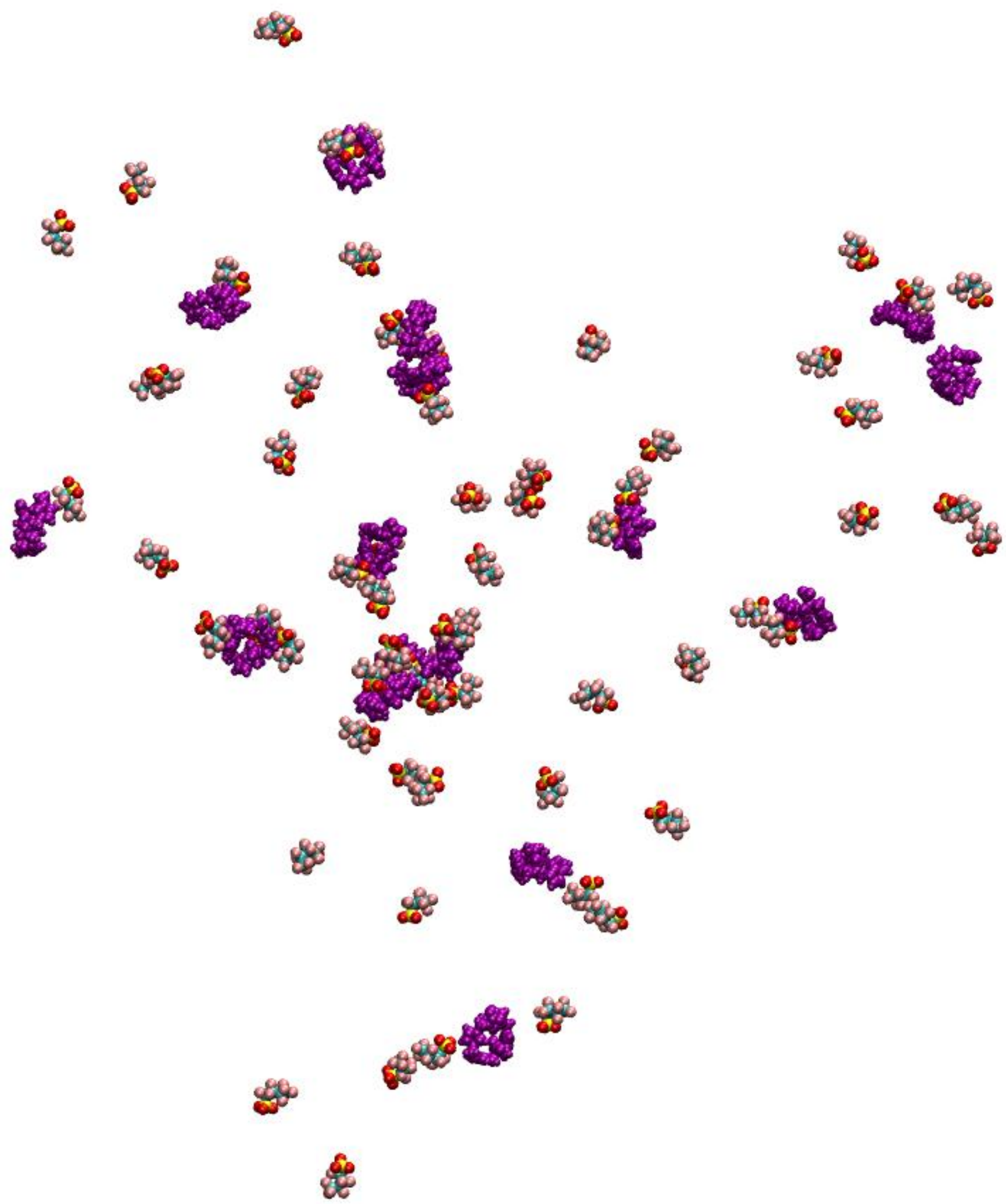

Figure S-85. Snapshot of the final configuration **CTI**:PFBS-supra (16:64)-supra.

8. References

1) Y. Liu, M. Mettry, A. D. Gill, L. Perez, W. Zhong, R. J. Hooley, Selective heavy element sensing with a simple host–guest fluorescent array. *Anal. Chem.* 2017, **89**, 11113–11121.

2) A. D. Gill, B. L. Hickey, S. Wang, M. Xue, W. Zhong, R. J. Hooley, Sensing of citrulline modifications in histone peptides by deep cavitand hosts. *Chem. Commun.* 2019, **55**, 13259–13262.

3) Z. Fu, J. Lin, C. Li, W. Yan, T. Wu, Cuprous iodide pseudopolymorphs based on imidazole ligand and their luminescence thermochromism. *Cryst. Growth Des.* 2016, **16**, 2322–2327.

4) H. Okamoto, T. Kozai, Z. Okabayaki, T. Shinmyozu, H. Ota, K. Amimoto, K. Satake, Synthesis, structure, and photoreactions of fluorinated 2,11-diaza[3_2]paracyclophane: Photochemical formation of cage-diene type benzene dimer. *J. Phys. Org. Chem.* 2017, **30**, e3726.

5) A. C. Garcia, R. Lian, P. Fasihianifard, L. He, R. R. Julian, V. Carta, C. A. Chang, R. J. Hooley, Cationic imidazolium macrocycles enable array-based, site-selective optical detection of peptide phosphorylation. *Org. Biomol. Chem.* 2026, **24**, 4731–4738.

6) N. Saigul, F. Korkmaz, İ. Kurultak, A New Artificial Urine Protocol to Better Imitate Human Urine. *Sci. Rep.* 2019, **9**, 20159–20170.

7) SAINT, V8.30A, (2012), Bruker Analytical X-Ray Systems, Madison, WI.

8) SADABS, 2.03, (2016), Bruker Analytical X-Ray Systems, Madison, WI.

9) G. M. Sheldrick, A Short History of SHELX. *Acta Cryst.* 2008, **A64**, 112–122.

10) G. M. Sheldrick, SHELXT-Integrated Space-Group and Crystal-Structure Determination. *Acta Cryst.* 2015, **A71**, 3–8.

11) O. V. Dolomanov, L. J. Bourhis, R. J. Gildea, J. A. K. Howard, H. Puschmann, OLEX2: a complete structure solution, refinement and analysis program. *J. Appl. Crystallogr.* 2009, **42**, 339–341.

12) W. Kohn, L. J. Sham, Self-Consistent Equations Including Exchange and Correlation Effects. *Phys. Rev.* 1965, **140**, A1133–A1138.

13) Y. Zhao, D. G. Truhlar, The M06 Suite of Density Functionals for Main Group Thermochemistry, Thermochemical Kinetics, Noncovalent Interactions, Excited States, and Transition Elements: Two New Functionals and Systematic Testing of Four M06-Class Functionals and 12 Other Functionals. *Theor. Chem. Acc.* 2008, **120**, 215–241.

14) F. Weigend, R. Ahlrichs, Balanced Basis Sets of Split Valence, Triple Zeta Valence and Quadruple Zeta Valence Quality for H to Rn: Design and Assessment of Accuracy. *Phys. Chem. Chem. Phys.* 2005, **7**, 3297–3305.

15) M. Cossi, V. Barone, B. Mennucci, J. Tomasi, Ab Initio Study of Ionic Solutions by a Polarizable Continuum Dielectric Model *Chem. Phys. Lett.* 1998, **286**, 253–260.

16) J. Wang, R. M. Wolf, J. W. Caldwell, P. A. Kollman, D. A. Case, Development and Testing of a General Amber Force Field. *J. Comput. Chem.* 2004, **25**, 1157–1174.

17) D. A. Case, H. M. Aktulga, K. Belfon, D. S. Cerutti, G. A. Cisneros, V. W. D. Cruzeiro, N. Forouzesh, T. J. Giese, A. W. Götz, H. Gohlke, S. Izadi, K. Kasavajhala, M. C. Kaymak, E. King, T. Kurtzman, T.-S. Lee, P. Li, J. Li, T. Luchko, R. Luo, M. Manathunga, M. R. Machado, H. M. Nguyen, K. A. O'Hearn, A. V. Onufriev, F. Pan, S. Pantano, R. Qi, A. Rahnamoun, A. Risheh, S. Schoot-Verdugo, A. Shajan, J. Swails, J. Wang, H. Wei, X. Wu, Y. Wu.; S. Zhang, S. Zhao, Q. Zhu, T. E. Cheatham III, D. R. Roe, A. Roitberg, C. Simmerling, D. M. York, M. C. Nagan, AmberTools. *J. Chem. Inf. Model.* 2023, **63**, 6183–6191.

18) D. A. Case, D. S. Cerutti, V. W. D. Cruzeiro, T. A. Darden, R. E. Duke, M. Ghazimirsaeed, G. M. Giambasu, T. J. Giese, A. W. Götz, J. A. Harris, K. Kasavajhala, T.-S. Lee, Z. Li, C. Lin, J. Liu, Y. Miao, R. Salomon-Ferrrer, J. Shen, R. Snyder, J. Swails, R. C. Walker, J. Wang, X. Wu, J. Zeng, T. E. Cheatham III, D. R. Roe, A. Roitberg, C. Simmerling, D. M. York, M. C. Nagan, K. M. Merz Jr. Recent Developments in Amber Biomolecular Simulations. *J. Chem. Inf. Model.* 2025, **65**, 7835–7843.

19) M. R. Shirts, C. Klein, J. M. Swails, J. Yin, M. K. Gilson, D. L. Mobley, D. A. Case, E. D. Zhong, Lessons Learned from Comparing Molecular Dynamics Engines on the SAMPL5 Dataset. *J. Comput.-Aided Mol. Des.* 2017, **31**, 147–161.

20) A. V. Marenich, S. V. Jerome, C. J. Cramer, D. G. Truhlar, Charge Model 5: An Extension of Hirshfeld Population Analysis for the Accurate Description of Molecular Interactions in Gaseous and Condensed Phases. *J. Chem. Theory Comput.* 2012, **8**, 527–541.

21) G. D. Barbosa, F. W. Tavares, A. Striolo, Molecular Interactions of Perfluorinated and Branched Fluorine-Free Surfactants at Interfaces: Insights from a New Reliable Force Field. *J. Chem. Theory Comput.* 2024, **20**, 7300–7314.

22) P. Mark, L. Nilsson, Structure and Dynamics of the TIP3P, SPC, and SPC/E Water Models at 298 K. *J. Phys. Chem. A* 2001, **105**, 9954–9960.

23) B. R. Brooks, C. L. Brooks, III, A. D. MacKerell Jr., L. Nilsson, R. J. Petrella, B. Roux, Y. Won, G. Archontis, C. Bartels, S. Boresch, A. Caflisch, L. Caves, Q. Cui, A. R. Dinner, M. Feig, S. Fischer, J. Gao, M. Hodoscek, W. Im, K. Kuczera, T. Lazaridis, J. Ma., V. Ovchinnikov, E. Paci, R. W. Pastor, C. B. Post, J. Z. Pu, M. Schaeffer, B. Tidor, R. M. Venable, H. L. Woodcock, X. Wu. W. Yang, D. M. York, M. Karplus CHARMM: The Biomolecular Simulation Program. *J. Comput. Chem.* 2009, **30**, 1545–1614.

24) T. Darden, D. York, L. Pedersen, Particle Mesh Ewald: An N log(N) Method for Ewald Sums in Large Systems. *J. Phys. Chem. A* 1993, **98**, 10089–10092.

25) S. Páll, M. J. Abraham, C. Kutzner, B. Hess, E. Lindahl Tackling Exascale Software Challenges in Molecular Dynamics Simulations with GROMACS. In International Conference on Exascale Applications and Software *Springer*, 2014, 3–27.

26) P. Bauer, B. Hess, E. Lindahl, GROMACS 2022 Source Code. *Zenodo*, 2022.

27) B. Hess, H. Bekker, H. J. C. Berendsen, J. G. E. M. Fraaije, LINCS: A Linear Constraint Solver for Molecular Simulations. *J. Comput. Chem.* 1997, **18**, 1463–1472.

28) S. Páll, B. Hess, A Flexible Algorithm for Calculating Pair Interactions on SIMD Architectures. *Comput. Phys. Commun.* 2013, **184**, 2641–2650.

29) G. Bussi, D. Donadio, M. Marrinello, Canonical Sampling Through Velocity Rescaling. *J. Chem. Phys.* 2007, **126**, 014101–014107.

30) M. Parrinello, A. Rahman, Polymorphic Transitions in Single Crystals: A New Molecular Dynamics Method. *J. Appl. Phys.* 1981, **52**, 7182–7190.
